# MELK inhibition disrupts actin cytoskeleton and broadly restricts human coronavirus infections

**DOI:** 10.1038/s41467-026-72615-1

**Published:** 2026-05-06

**Authors:** Kuai Yu, Qiaorui Yao, Dong Wang, Qingtao Hu, Dandan Li, Fang Li, Fenghua Chen, Jingyi Su, Ting Huang, Qing Zhang, Zishuo Lin, Wei Ran, Yiliang Wang, Yuzheng Zhou, Shuai Wen, Yuting Lin, Yaming Jiu, Jingxian Zhao, Jincun Zhao

**Affiliations:** 1 State Key Laboratory of Respiratory Disease, National Clinical Research Centre for Respiratory Disease, National Centre for Respiratory Medicine, Guangzhou Institute of Respiratory Health, The First Affiliated Hospital of Guangzhou Medical University, Guangzhou Medical University, Guangzhou, China; 2 Guangzhou National Laboratory, Guangzhou, China; 3 GMU-GIBH Joint School of Life Sciences, The Guangdong-Hong Kong-Macao Joint Laboratory for Cell Fate Regulation and Diseases, Guangzhou Medical University, Guangzhou, China; 4 Proteomics and Metabolomics Core Facility, Guangzhou National Laboratory, Guangzhou, China; 5 School of Basic Medical Sciences, Guangzhou Medical University, Guangzhou, China; 6 Institute for Hepatology, National Clinical Research Center for Infectious Disease, Shenzhen Third People’s Hospital, The Second Affiliated Hospital, School of Medicine, Southern University of Science and Technology, Shenzhen, China; 7 Unit of Cell Biology and Imaging Study of Pathogen Host Interaction, Key Laboratory of Molecular Virology and Immunology, Shanghai Institute of Immunity and Infection, Chinese Academy of Sciences, Shanghai, China; 8 University of Chinese Academy of Sciences, Beijing, China; 9 Shanghai Institute for Advanced Immunochemical Studies, School of Life Science and Technology, Shanghai Tech University, Shanghai, China

**Keywords:** Virus-host interactions, Actin

## Abstract

Protein kinases regulate numerous critical biological processes in eukaryotic cells and are important targets for drug development. However, the common functional protein kinases and corresponding inhibitors with broad-spectrum therapeutic potential for human coronavirus infections remain largely unknown. By integrating global phosphoproteomics and high-content screening, we identify maternal embryonic leucine zipper kinase (MELK) as a common kinase required for the infections of multiple human coronaviruses currently circulating in the population. Inhibition of MELK activity by OTSSP167, or genetic depletion of its expression, exhibits broad antiviral effects in cells, human airway organoids as well as in mice under a prophylactic setting. Intriguingly, super-resolution imaging reveals that MELK colocalizes with the cellular actin cytoskeleton that is required for viral infection. Subsequently, live-cell imaging demonstrates that OTSSP167 treatment disrupts the dynamics of actin cytoskeleton. Mechanistic analysis reveals that MELK directly phosphorylates a key actin-depolymerizing protein, cofilin-1 at S3 and T70, thereby suppressing its actin-severing activity. Inhibiting MELK activity or expression activates cofilin-1 and disrupts actin filament formation, thereby impeding multiple steps of viral life cycle. Collectively, our study reveals a common regulation of coronavirus infections by MELK through modulation of actin cytoskeleton, and the broad antiviral effect of OTSSP167 as a novel actin cytoskeleton modulator.

## Abstract (web summary)

Human coronavirus infections broadly require host kinase MELK, which stabilizes the actin cytoskeleton by phosphorylating cofilin-1. Consequently, MELK inhibition by OTSSP167 exhibits broad-spectrum antiviral activity against diverse strains.

## Introduction

Protein kinases are a large superfamily responsible for protein phosphorylation^1^. Protein phosphorylation represents one of the most pervasive and critical post-translational modifications (PTMs), serving as a molecular switch to control diverse protein functions such as activity, turnover, interaction, conformation, localization, and crosstalk with other PTMs^2,3^. Over two-thirds of human proteins can be phosphorylated, regulating numerous critical cellular processes^2,4^. Owing to aberrant activity and expression of kinases associated with various disorders including cancer and inflammatory diseases, the kinase family has become one of the most important drug targets^4–6^. Until 2023, the Food and Drug Administration (FDA) had approved 80 kinase inhibitors, mostly for cancer treatment, but none for viral infections^7^. Currently, host kinases that play conserved regulatory roles across diverse human coronaviruses remain largely unidentified.

Coronaviruses are a highly diverse family of enveloped positive-sense single-stranded RNA viruses that can cause mild to severe respiratory infections in humans^8,9^. Seven human coronaviruses have been identified, including severe acute respiratory syndrome coronavirus (SARS-CoV), Middle East respiratory syndrome coronavirus (MERS-CoV), severe acute respiratory syndrome coronavirus 2 (SARS-CoV-2), HCoV-229E, HCoV-NL63, HCoV-OC43, and HCoV-HKU1^8,9^. Since 2002, three zoonotic highly pathogenic coronaviruses, including SARS-CoV, MERS-CoV, and SARS-CoV-2, have caused fatal respiratory illnesses and outbreaks in humans^10^. The frequent adaptive mutation and recombination characteristics of coronaviruses increase the possibility of interspecies transmission and evasion of treatment, especially vaccines and antibodies^8–10^. Therefore, broad-spectrum antiviral strategies are important for controlling the current and potential emergence of coronavirus epidemics. Global phosphoproteomics has been widely utilized to elucidate host signaling perturbations in various models, such as SARS-CoV-2-infected Vero E6 (African green monkey kidney)^11^, Caco-2 (human colon epithelial)^12^, and primary human nasal epithelial cells (HNE)^13^, as well as SARS-CoV-2/SARS-CoV comparisons in A549-ACE2 (human lung carcinoma cells transduced with ACE2)^14^. These studies identified significant viral regulation of growth factor receptor (GFR) and mitogen-activated protein kinase (MAPK) signaling pathways, cell cycle, and cytoskeleton. However, the simultaneous global phosphoproteomic profiling of multiple coronaviruses to reveal broader regulatory mechanisms is currently lacking. Whether certain kinases broadly regulate human coronavirus infections and whether their corresponding inhibitors possess broad-spectrum antiviral properties remains unclear.

The actin cytoskeleton is essential for cell mechanics and has increasingly been implicated in the mediation of cell signaling^15^. The actin cytoskeleton is highly dynamic through simultaneous polymerization and depolymerization, reflecting the transition between monomeric globular actin (G-actin) and polymeric filamentous actin (actin filaments, F-actin)^16^. Actin-binding proteins account for every aspect of these dynamics, such as cofilin-1 catalyzing the severing of F-actin and profilin-1 promoting and stabilizing F-actin formation^17,18^.

Viruses have evolved to exploit and reorganize cellular actin cytoskeleton at every stage of their lifecycle, including entry, replication, assembly, and egress^16,19,20^. For example, human immunodeficiency virus (HIV) remodels the actin cytoskeleton to induce receptor clustering for entry^21^. Real-time imaging visualizes SARS-CoV-2 virus-like particles (VLPs) boosting and utilizing filopodia to enter cells^22^. Later during infection, F-actin rearrangements induced by SARS-CoV-2 infection are important for particle production in lung cells^23^. Similarly, SARS-CoV-2 infection triggers the reprogramming of microvilli for egress in primary nasal epithelial organoids^24^. Therefore, regulation of the actin cytoskeleton rationally affects every stage of the viral life cycle^16,25,26^. While the relationship between the cellular actin cytoskeleton and viral infection has been well documented, the key regulators and mechanisms remain largely unclear.

Here, we identify maternal embryonic leucine zipper kinase (MELK) as a common kinase regulating multiple human coronavirus infections, and also reveal its critical role in cellular actin cytoskeleton. MELK directly phosphorylates cofilin-1 at S3 and T70, thereby suppressing its severance activity of actin cytoskeleton. Inhibition of MELK activates cofilin-1, leading to the severance and disturbance of the actin cytoskeleton, which exhibits broad antiviral effects against human coronaviruses. These findings unveil a common regulation of coronavirus infections by MELK through the modulation of actin cytoskeleton. Targeting MELK potentially provides new avenues for antiviral strategy development.

## Results

### MELK is a common kinase required for multiple human coronavirus infections

**Fig. 1:**
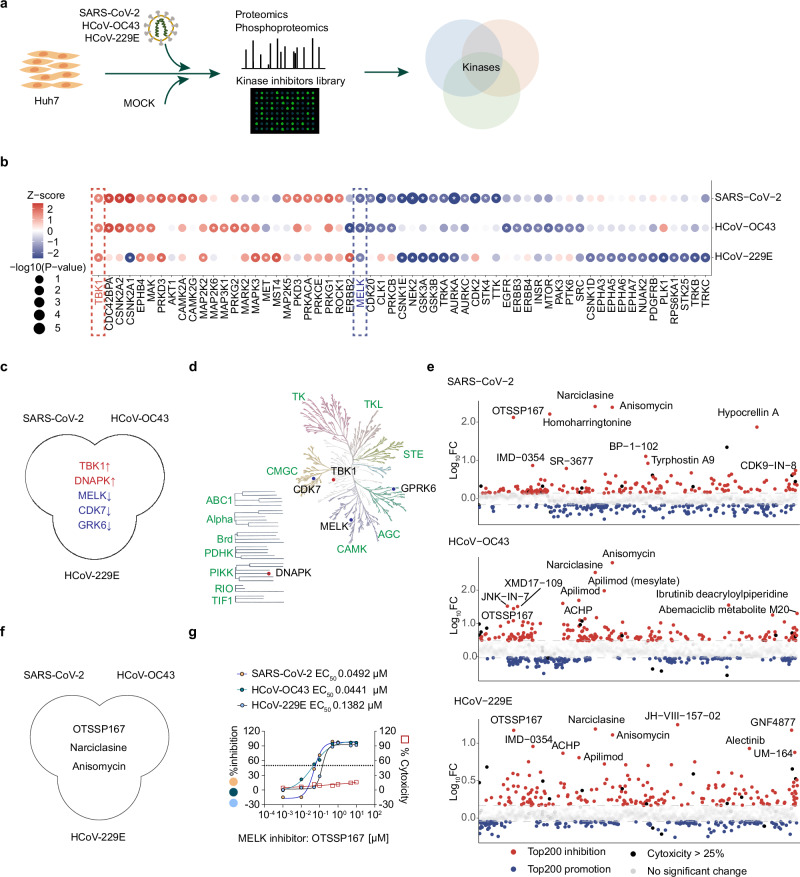
MELK is a common kinase involving multiple human coronavirus infections. **a** Workflow schema for the identification and screening of common kinases. Huh7 cells were infected with SARS-CoV-2 (MOI = 1), HCoV-229E (MOI = 0.1) or HCoV-OC43 (MOI = 1), respectively. For proteomics and phosphoproteomics analysis, cells were harvested at 24 h post-infections. All samples were analyzed using mass spectrometry with a data-independent analysis (DIA) approach to measure changes in both protein abundance and phosphorylation level upon infections. For kinase inhibitor library screening, the infection rate was measured at 24 h post-infections using the Celigo Image Cytometer with DAPI and N protein staining, and the cytotoxicity of the compounds in Huh7 cells was determined by the CCK8 assay. The results of phosphoproteomics and screening were combined to analyze the common functional kinases upon the three coronavirus infections. **b** Kinase substrate enrichment analysis (KSEA) of changes in kinase activities upon three coronavirus infections. Kinases with statistically significant change in activity were marked with an asterisk (*). Source data are provided as a Source Data file. **c** Venn diagram showing the kinases with common changes in activity across the three viral infections. Upregulated (red, ↑) and downregulated (blue, ↓) kinases are shown. Both KSEA and PTM-SEA consistently identified the suppression of MELK. The specific identification methods for the other kinases are as follows: KSEA identified TBK1; motif-centric analysis identified GRK6; and PTM-SEA identified DNA-PK and CDK7. **d** The three identified candidate kinases were mapped to the KinMap. http://www.kinhub.org/kinmap/index.html. Upregulated (red) and downregulated (blue) kinases are shown. **e** The results of kinase inhibitor library high-content screening. Red dots represent the top 200 compounds with inhibitory functions against viral infection, with the top 10 compounds annotated. Blue dots represent the top 200 compounds promoting viral infection. Black dots indicate compounds with strong cytotoxicity, over 25%. Source data are provided as a Source Data file. **f** Venn diagram showing the kinases inhibitors with suppressive functions against all three coronavirus infections, OTSSP167, Narciclasine and Anisomycin. **g** Percent inhibition of OTSSP167 against SARS-CoV-2, HCoV-229E and HCoV-OC43 infections and cytotoxicity in Huh7 cells. Huh7 cells were infected with SARS-CoV-2 (MOI = 1), HCoV-229E (MOI = 0.1) or HCoV-OC43 (MOI = 1), respectively, in the presence of a range of concentrations for 24 h. The infection rate was measured using IFA with N protein staining, and the cytotoxicity of the compounds was determined by the CCK8 assay. Graphs depict the percentage inhibition of viral infection (left *Y*-axis), percentage cytotoxicity (right Y-axis) and the concentration causing a 50% reduction in replication (EC_50_). Data are presented as mean ± SEM of *n* = 3 independent biological replicates. Source data are provided as a Source Data file. See also Supplementary Figs. 1 and 2.

To comprehensively identify common functional protein kinases involved in human coronavirus infections, we integrated two strategies: global phosphoproteomics and high-content screening of kinase inhibitors (Fig. 1a). Common changes in the activity of certain kinases suggest a conserved association with viral infections. Here, we utilized proteomics and phosphoproteomics to reveal global changes in protein phosphorylation during coronavirus infections and employed state-of-the-art bioinformatics approaches to identify common changes in kinase activity in response to the three human coronaviruses, including SARS-CoV-2, HCoV-229E, and HCoV-OC43.

To exclude variations arising from different cellular backgrounds in phosphoproteomics, we aimed to identify a cell line susceptible to all three coronaviruses. However, currently available lung-derived cells do not simultaneously support the efficient replication of SARS-CoV-2, HCoV-229E, and HCoV-OC43. Therefore, we adopted the strategy from a pan-coronavirus genome-wide CRISPR screen and selected Huh7 cells, which are susceptible to all three coronaviruses^27,28^. We infected Huh7 cells with SARS-CoV-2, HCoV-229E, or HCoV-OC43, respectively, and subsequently harvested the cells after 24 h. A data-independent acquisition (DIA) approach was used to analyze changes in both global protein abundance and phosphorylation. Quality control demonstrated high consistency and quantitative reproducibility of omics data (Supplementary Fig. 1a–d). In total, high-quality quantification of 7593 proteins and 21,761 phosphorylation sites (localization cutoff >0.9) was achieved (Supplementary Fig. 1e and Supplementary Data 1). Subsequently, we evaluated the contribution of protein abundance to changes in phosphorylation levels. No significant changes in protein abundance were observed for the majority of significantly altered phosphorylation sites (Supplementary Fig. 1f), indicating that phosphorylation signaling represents an independent host response to coronavirus infections, largely uncoupled from changes in protein expression. The protein abundance (Supplementary Fig. 1g) and phosphorylation levels (Supplementary Fig. 1h) that were significantly altered upon the three human coronavirus infections are displayed using a heatmap and Venn diagram. The common changes in protein abundance and phosphorylation sites suggested their potential conserved association with viral infections. Next, we analyzed the common functions in biological processes of proteins exhibiting significant changes in abundance (Supplementary Fig. 1i) or phosphorylation (Supplementary Fig. 1j) using gene ontology enrichment. Notably, proteins exhibiting significantly altered phosphorylation levels were highly enriched in functional categories such as RNA splicing, histone modification, and actin filament organization (Supplementary Fig. 1j).

Numerous phosphorylation events have been extensively studied, and the kinases and their specific substrates are well annotated^2,29^. To infer kinase activities from the phosphorylation of their substrates, we performed Kinase Substrate Enrichment Analysis (KSEA). Notably, KSEA demonstrated that the innate immune regulator, TANK-binding kinase 1 (TBK1), was consistently activated (positive Z-score), whereas MELK exhibited conserved suppression (negative *Z*-score) (Fig. 1b, Supplementary Data 2). To ensure robust inference, we complemented KSEA with orthogonal approaches. Motif-centric analysis specifically identified the conserved suppression of G protein-coupled receptor kinase 6 (GRK6) (Supplementary Fig. 2a). Furthermore, PTM-SEA identified the consistent activation of DNA-dependent protein kinase (DNA-PK) and the shared suppression of cyclin-dependent kinase 7 (CDK7) and MELK (Supplementary Fig. 2b). Overall, these inferred kinase activity results revealed a conserved regulatory landscape across the three viral infections (Fig. 1c). These kinases were mapped to KinMap^30^ (Fig. 1d). Notably, our findings align well with previous reports. Our observation of MELK suppression is consistent with the global phosphorylation landscape of SARS-CoV-2 infection reported by Bouhaddou et al.^11^, where MELK activity was downregulated at 8, 12, and 24 h post-infection. Similarly, our finding regarding CDK7 is corroborated by Wu et al.^13^. Their dataset reveals a consistent trend of suppressed CDK7 activity at multiple time points (6, 24, 34, and 48 h) following SARS-CoV-2 infection in human airway epithelial cells. TBK1 is a well-studied central kinase in innate immunity^31^. The activation of TBK1 coincides with an induced innate immune response following coronavirus infections^32,33^, which was confirmed by examining the activated form of TBK1 (phosphorylated TBK1 at serine residue 172, p-TBK1-S172) in response to three human coronavirus infections (Supplementary Fig. 2c). These immunoblotting findings are consistent with the phosphoproteomics results, verifying the activation of TBK1 across three coronavirus infections. As expected, treatment with TBK1 inhibitors increased the infection rates of all three human coronaviruses (Supplementary Fig. 2d), without affecting cell viability (Supplementary Fig. 2e).

Furthermore, high-content and high-throughput kinase inhibitor screening for multiple human coronaviruses is also a powerful strategy for discovering both common functional kinases and broad-spectrum kinase inhibitors. A compound screening library containing 1805 kinase inhibitors was simultaneously evaluated through its effect on the infection rate of SARS-CoV-2, HCoV-229E, and HCoV-OC43 (Fig. 1e). Huh7 cells were treated with DMSO or diluted compounds at a working concentration of 1 μM simultaneously with virus or MOCK infection. After 24 h, cell viability was assessed using a Cell Counting Kit-8 (CCK8) assay, and the infection rate was determined through high-content analysis with an indirect immunofluorescence assay (IFA) (Fig. 1e). The qualified candidates were considered based on high efficiency and low toxicity. To identify candidate kinase inhibitors that could significantly regulate three coronavirus infections, we selected top ten kinase inhibitors with inhibitory or promoting effects based on the SARS-CoV-2, HCoV-229E, and HCoV-OC43 screening results, then organized in a Venn diagram. The results demonstrated that three kinase inhibitors, OTSSP167, Narciclasine, and Anisomycin, inhibited all three virus infections, while none was observed to enhance all their infections (Fig. 1f). Interestingly, OTSSP167, which has been tested in clinical trials for cancer treatment^34^, is an inhibitor of MELK. The concentrations of OTSSP167 causing a 50% reduction in viral replication (EC_50_) are 0.0492 μM against SARS-CoV-2, 0.1382 μM against HCoV-229E, and 0.0441 μM against HCoV-OC43, respectively (Fig. 1g). MELK, a Ser/Thr protein kinase, is highly expressed in various tumors associated with poor prognosis and has been extensively studied as an anti-tumor therapeutic target^35–37^. Currently, its role in the coronavirus life cycle remains unknown. The suppression of MELK activity suggests a conserved association with human coronavirus infections.

Collectively, the results of phosphoproteomic analysis and high-content screening indicated that MELK is a common kinase broadly required for human coronavirus infections.

### MELK inhibition blocks multiple human coronavirus infections

**Fig. 2:**
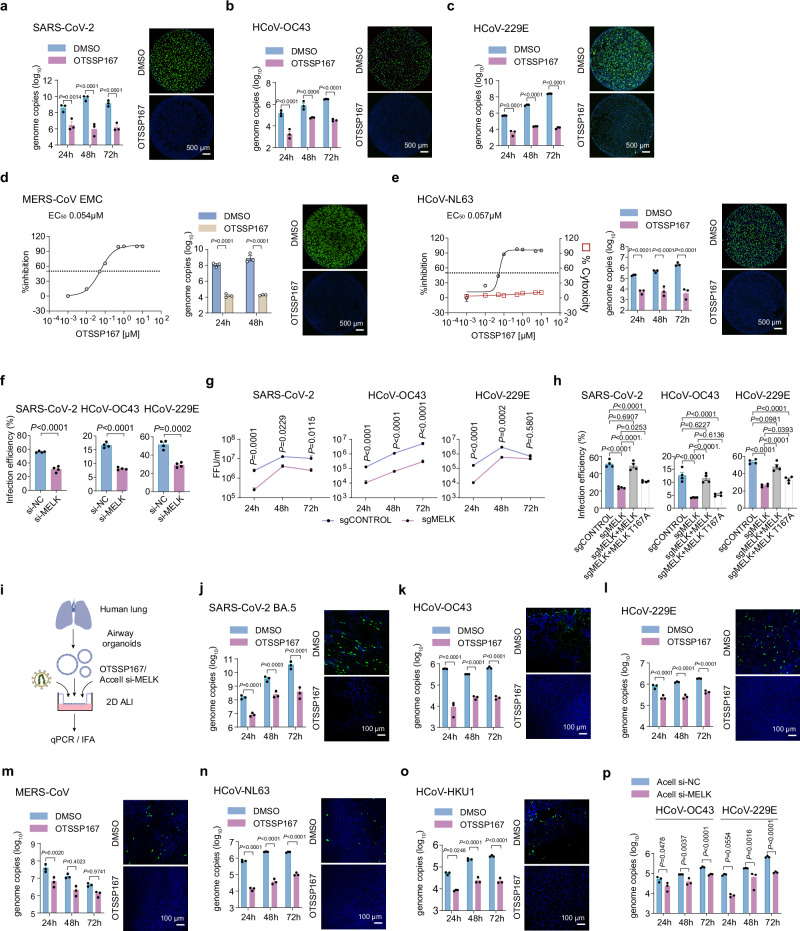
MELK inhibition blocks multiple human coronavirus infections. **a**–**e** OTSSP167 inhibited the infections of SARS-CoV-2, HCoV-229E and HCoV-OC43. **a**–**d** Huh7 or **e** Huh7-ACE2 cells were infected with SARS-CoV-2 (MOI = 1), HCoV-229E (MOI = 0.1), HCoV-OC43 (MOI = 1), MERS-CoV (MOI = 0.1), and HCoV-NL63 (MOI = 1), respectively, in the presence of **a**–**c** OTSSP167 (0.2 μM) or **d**, **e** a range of concentrations. Virus gene copy number in supernatant was quantified by qPCR of the N gene at 24, 48, and 72 hpi. Viral infections were visualized using IFA with N protein staining at 24 hpi. Images were representative of three independent experiments. Source data are provided as a Source Data file. **f**–**h** Decreasing the expression of MELK blocked the infections of SARS-CoV-2, HCoV-229E, and HCoV-OC43. The expression of MELK was decreased with **f** siRNA transfection or **g**, **h** sgRNA contained lentivirus transduction. Rescue experiments were performed using both wild-type and kinase-dead (T167A) MELK. The infection rate was measured as in Fig. 1a. Viral titers of supernatants were determined by focus-forming assay (FFA) at the indicated time points. Source data are provided as a Source Data file. **i**–**p** Antiviral efficacy of MELK inhibition against multiple human coronavirus infections in 2D human proximal airway organoids. **j**–**o** MELK activity was inhibited using OTSSP167 (0.2 μM). **p** MELK expression was decreased using the indicated Accel siRNA. Viral titers of supernatants from the apical chambers of the 2D proximal airway organoids were detected by real-time PCR. Viral infections were visualized using IFA with N protein staining at 48 hpi. Images were representative of three independent experiments. Source data are provided as a Source Data file. Data presented in **a**–**e**, **g**, **j**–**p** are mean ± SEM of *n* = 3 independent biological replicates. Statistical significance was determined using two-way ANOVA followed by two-sided Sidak’s multiple comparisons test. Exact *p* values are indicated in the figure. Data presented in **f** are mean ± SEM of *n* = 4 independent biological replicates. Statistical significance was determined using two-tailed Student’s *t* test. Exact *p* values are indicated in the figure. Data presented in **h** are mean ± SEM of *n* = 4 independent biological replicates. Statistical significance was determined using one-way ANOVA followed by two-sided Dunnett’s multiple comparisons test. Exact *p* values are indicated in the figure. See also Supplementary Figs. 3–5.

To further investigate the antiviral effects of MELK inhibition, we initially assessed the impact of OTSSP167 on the replication and infection of three human coronaviruses. Huh7 cells were infected with SARS-CoV-2 (Fig. 2a), HCoV-OC43 (Fig. 2b), or HCoV-229E (Fig. 2c), respectively, in the presence of OTSSP167 or DMSO as a control. The released virus in the supernatant was detected by qPCR at 24, 48, and 72 h post-infection (hpi), and the infected cells were analyzed by IFA at 24 hpi. The results demonstrated that OTSSP167 treatment significantly inhibited viral replication and infection (Fig. 2a–c). Given the diverse sequence mutations and infection characteristics of SARS-CoV-2 variants of concern (VOCs), we evaluated the effect of OTSSP167 on various SARS-CoV-2 variants, including Alpha, Beta, Delta, BA.1, BA.2.3, BA.5, XBB.1.5, XBB.1.16, EG.5, and JN.1 (Supplementary Fig. 3a–k and Supplementary Table 1). The results from EC_50_, qPCR, and IFA indicated OTSSP167 exhibited obvious antiviral activity against VOCs comparable to that observed with WT. Additionally, OTSSP167 also exhibited significant antiviral activity against MERS-CoV (EMC/2012, GD01, Nigeria) (Fig. 2d and Supplementary Fig. 4a, b) and HCoV-NL63 (Fig. 2e), indicating its broad-spectrum anti-coronavirus efficacy. To evaluate the effect of MELK expression level on viral infection, we employed siRNA knockdown or Cas9 knockout to decrease the expression of MELK (Supplementary Fig. 4c, d). The results demonstrated a significant decrease in viral replication in cells with decreased MELK expression compared to control cells (Fig. 2f–h). Additionally, we performed rescue experiments using both wild-type and kinase-dead (T167A) MELK^38^. The results showed that only the wild-type MELK, but not the kinase-dead (T167A) mutant, could restore the infection phenotype (Fig. 2h), indicating that decreased expression of MELK inhibits coronavirus infections.

Next, we assessed the effect of MELK inhibition using an ex vivo human proximal airway organoid model (Fig. 2i), which comprises major types of human airway epithelial cells and is widely used for studying human respiratory viral pathogenesis. OTSSP167 and Accell-siRNA were respectively used to inhibit activity and expression of MELK in human proximal airway organoids (Fig. 2i and Supplementary Fig. 4e). Consistent with our previous findings, OTSSP167 significantly reduced the replication and N protein expression of six human coronaviruses, including SARS-CoV-2 (Fig. 2j), HCoV-OC43 (Fig. 2k), HCoV-229E (Fig. 2l), MERS-CoV (Fig. 2m), HCoV-NL63 (Fig. 2n) and HCoV-HKU1 (Fig. 2o). Collectively, these findings demonstrated that OTSSP167 exhibits broad-spectrum inhibition of coronavirus replication in both cultured cells and organoids, without affecting cell viability (Supplementary Fig. 4f–i). Similarly, decreasing the expression of MELK inhibited coronavirus infections in organoids (Fig. 2p). In addition to OTSSP167, we evaluated the effects of two other MELK inhibitors, MELK-IN-1 and MELK-8a, on viral infections. The results indicated that both MELK-IN-1 and MELK-8a exhibited antiviral activity against SARS-CoV-2, HCoV-OC43, and HCoV-229E (Supplementary Fig. 5a, b). However, their EC_50_ values were significantly higher than those of OTSSP167. Furthermore, the inhibitory effect of OTSSP167 on viral replication was markedly attenuated in sgMELK-transduced cells (Supplementary Fig. 5c, d). To further validate the direct engagement of OTSSP167 with MELK, we performed the Cellular Thermal Shift Assay (CETSA) in both Huh7 cells and human airway organoids. The results revealed that OTSSP167 treatment caused a significant thermal destabilization of MELK compared to the vehicle control. This ligand-induced destabilization provided evidence of the direct interaction between OTSSP167 and MELK in the cellular environment. Interestingly, a recent study revealed that the binding of certain inhibitors can induce the destabilization of target kinases, leading to their subsequent degradation^39^. Consistent with this phenomenon, time-course analysis revealed that OTSSP167 treatment triggered MELK degradation in cells (Supplementary Fig. 5e–g). These findings suggested that the antiviral activity of OTSSP167 was dependent on MELK.

Collectively, these results demonstrated that inhibiting both MELK activity and expression can effectively block multiple human coronavirus infections.

### MELK inhibitor impairs the replication of SARS-CoV-2 and HCoV-OC43 in vivo

**Fig. 3:**
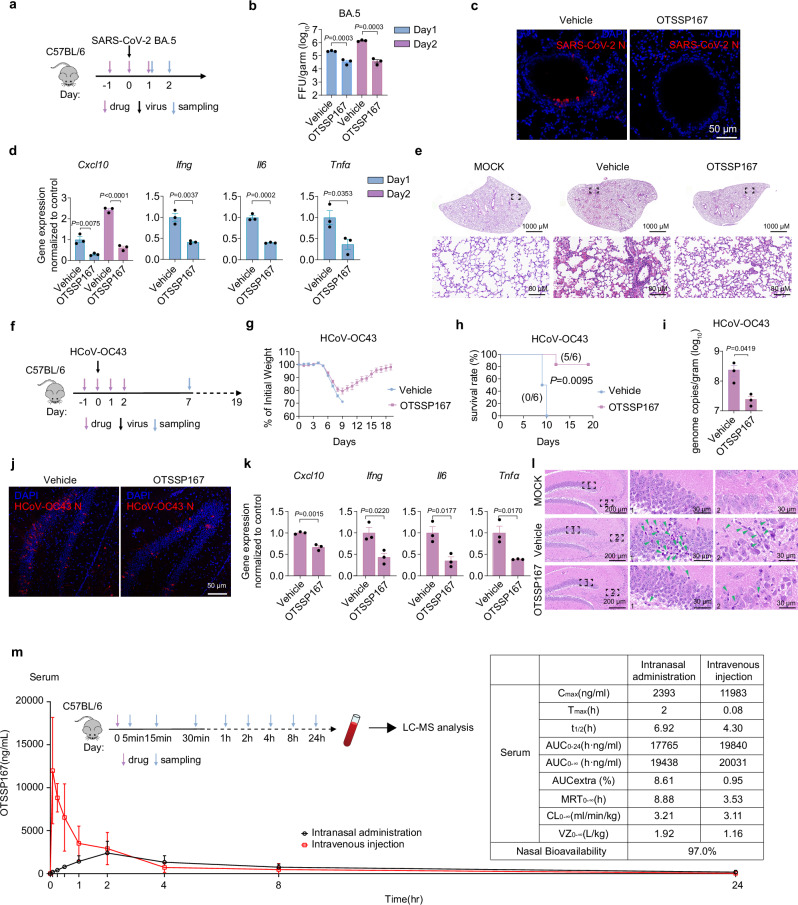
MELK inhibitor impairs the replication of SARS-CoV-2 and HCoV-OC43 in vivo. **a** Six-week-old C57BL/6 mice were intranasally infected with 1 × 10^5^ FFU omicron BA.5 and treated with intranasal administration of DMSO or OTSSP167 (3.75 mg/kg/day) for 3 days. Lungs of infected mice were collected on 1 dpi and 2 dpi. **b** The viral titers in lungs were determined by FFA. Source data are provided as a Source Data file. **c** Viral N protein expression in lung with or without OTSSP167 treatment was detected by immunofluorescence. Images were representative of three independent experiments. **d** The infected mice were sacrificed at 1 dpi and 2 dpi, and the expression of pro-inflammatory cytokines in lungs was quantified by qPCR. Source data are provided as a Source Data file. **e** Representative images of haematoxylin and eosin (H&E)-stained lungs. Images were representative of three independent experiments. **f** Six-week-old C57BL/6 mice were intranasally infected with 2 × 10^4^ FFU OC43 and treated with intranasal administration of DMSO or OTSSP167 (3.75 mg/kg/day) from day −1 to day 2. Brains of infected mice were collected at 7 dpi for viral burden and inflammation response determination. **g** Body weight and **h** survival of the infected mice were monitored for 19 days. Source data are provided as a Source Data file. **i** Infected mice were sacrificed at 7 dpi, and viral gene copies in brain were quantified by qPCR. Source data are provided as a Source Data file. **j** Viral N protein expression in brain, with or without OTSSP167 treatment, was detected by immunofluorescence at 7 dpi. Images were representative of three independent experiments. **k** The expressions of pro-inflammatory cytokines in brain were quantified by qPCR at 7 dpi. Source data are provided as a Source Data file. **l** Representative images of haematoxylin and eosin (H&E)-stained brains. Images were representative of three independent experiments. **m** Pharmacokinetic profiles of OTSSP167 in mice. Serum concentration-time curves of OTSSP167 in C57BL/6 mice following intranasal (black line) or intravenous (red line) administration. Blood samples were collected at the indicated time points (5 min to 24 h) and analyzed by LC-MS. The table (right) summarizes key pharmacokinetic parameters, maximum concentration (Cmax), time to peak (Tmax), elimination half-life (*t*_1/2_), area under the curve (AUC_0-24_ and AUC_0-∞_), mean residence time (MRT_0-∞_), clearance (CL_0-∞_), and volume of distribution (VZ_0-∞_). The absolute bioavailability of intranasal administration was determined to be 97.0%. Source data are provided as a Source Data file. Data presented in **b**, **d**, **i**, **k** are mean ± SEM of *n* = 3 independent biological replicates. Statistical significance was determined using a two-tailed Student’s *t* test. Exact *p* values are indicated in the figure. Data presented in **h** are survival curves of *n* = 6 mice per group. Statistical significance was determined using Log-rank (Mantel-Cox) test. Exact *p* values are indicated in the figure. Data presented in **m** are mean ± SD of *n* = 3 independent biological replicates. See also Supplementary Fig. 6.

To determine the antiviral potency of OTSSP167 in vivo, we employed a non-lethal SARS-CoV-2 BA.5 infection mouse model and a lethal HCoV-OC43 infection mouse model^40^. Mice were infected with 1 × 10^5^ FFU of BA.5. Intranasal (i.n.) administration of OTSSP167 (3.75 mg/kg) was applied, with the first dose given at −1 dpi and once daily for 3 consecutive days (Fig. 3a). The uninfected mice treated in parallel with the same dose for 5 days exhibited no significant body weight loss or overt toxicity (Supplementary Fig. 6a, b). In lung tissues, MELK inhibition with OTSSP167 significantly reduced viral titers (Fig. 3b), N protein expression (Fig. 3c), virus-induced pro-inflammatory cytokines and chemokines (Fig. 3d). Expectedly, OTSSP167-treated mice exhibited improved lung histology (Fig. 3e).

Next, we evaluated the anti-HCoV-OC43 activity of OTSSP167 in vivo. OTSSP167 (3.75 mg/kg, i.n.) was administered daily starting from −1 dpi (1 day prior to viral challenge) through 2 dpi, with mice being challenged with 2 × 10⁴ FFU of HCoV-OC43 (neurotropic) at 0 dpi (Fig. 3f). OTSSP167 substantially attenuated body weight loss in OC43 infected mice (Fig. 3g). All vehicle-treated mice died on or before 9 dpi, whereas OTSSP167 treatment resulted in higher survival rate (83.3% vs. 0%, *p* = 0.0095) (Fig. 3h). In mouse brain tissues, MELK inhibition with OTSSP167 significantly reduced the expression of HCoV-OC43 N gene (Fig. 3i), N protein (Fig. 3j), virus-induced pro-inflammatory cytokines and chemokines (Fig. 3k). The attenuated virus replication and expression of pro-inflammatory markers were accompanied by fewer histological changes. Brains from HCoV-OC43-infected mice in the vehicle treatment group showed severe progressive degeneration of neurons in hippocampal layers (Fig. 3l, middle). By contrast, neuron damage was greatly ameliorated in the brains of OTSSP167-treated mice (Fig. 3l, bottom). Additionally, to evaluate the therapeutic potential of OTSSP167, we initiated treatment at 12 or 24 h post-infection. The results showed that post-infection treatment also significantly inhibited the replication of both SARS-CoV-2 BA.5 and HCoV-OC43 in vivo and improved survival rates to a certain extent (Supplementary Fig. 6c–h). However, the therapeutic efficacy was less potent compared to prophylactic administration. This suggests that, similar to many anti-coronavirus agents^41,42^, OTSSP167 exerts its optimal antiviral effects when administered prophylactically or early. Taken together, OTSSP167 effectively protected mice from HCoV-OC43 and SARS-CoV-2 infections.

To evaluate the translational potential, we further analyzed the pharmacokinetics of OTSSP167 in C57BL/6 mice serum following intranasal (i.n.) administration, using intravenous (i.v.) injection as a reference. The LC-MS analysis demonstrated that intranasal delivery resulted in excellent systemic absorption, with an absolute bioavailability of 97.0%. Notably, intranasal administration exhibited a sustained-release profile with a longer half-life (6.92 h) compared to the intravenous route (4.30 h). These results demonstrated that intranasal administration of OTSSP167 effectively achieves robust and sustained systemic drug exposure (Fig. 3m).

### MELK inhibition disrupts cellular actin cytoskeleton

**Fig. 4:**
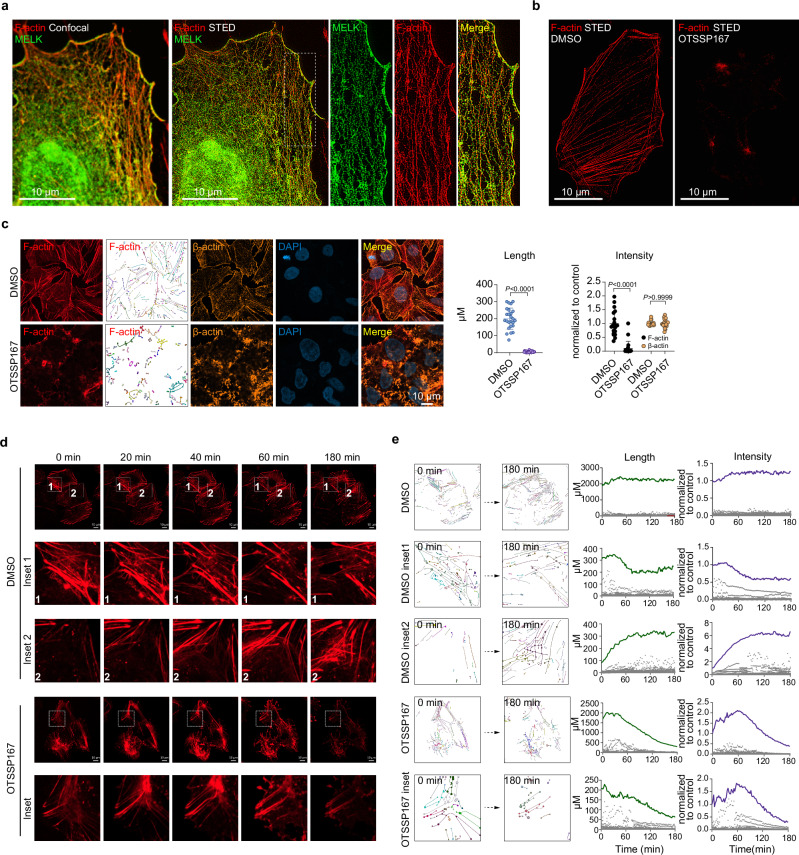
MELK inhibition disrupts actin cytoskeleton. **a** MELK colocalized with F-actin. Representative super-resolution stimulated-emission-depletion (STED) microscopy images showed colocalization of MELK (green) with F-actin (STAR RED phalloidin, red) in Huh7 cells. Images were representative of three independent experiments. **b**, **c** MELK inhibition in activity with OTSSP167 induced depolymerization of F-actin. **b** Representative STED microscopy images showed F-actin in DMSO-treated and OTSSP167-treated cells. **c** Representative immunostaining of G-actin and F-actin in DMSO-treated and OTSSP167-treated cells. Cells were fixed and immunostained with **b** STAR RED phalloidin or **c** AF647-phalloidin and anti-β-actin antibodies. **c** The F-actin length and the intensities of F-actin and G-actin in individual cells were quantified using Arivis Vision4D software. Images were representative of three independent experiments. Source data are provided as a Source Data file. **d**, **e** OTSSP167 impaired actin cytoskeleton dynamics. **d** Live-cell imaging using fluorogenic probe SiR-actin visualized the changes in actin cytoskeleton dynamics in DMSO-treated and OTSSP167-treated cells. **e** The F-actin length and the intensities of F-actin and G-actin in individual cells were quantified using Arivis Vision4D software. Source data are provided as a Source Data file. Data presented in **c** are mean ± SD of length (*n* = 25) and Intensity (*n* = 20) from three independent biological replicates. Statistical significance was determined using a two-tailed Student’s *t* test. Exact *p* values are indicated in the figure. See also Supplementary Fig. 7.

To investigate the underlying mechanism by which MELK inhibition impedes coronavirus infections, we first examined the subcellular localization of MELK, a critical determinant of protein function in cellular signaling^43^. Interestingly, in addition to its reported membrane^44,45^ and nuclear localization^46,47^, the super-resolution stimulated-emission-depletion (STED) microscopy images (Fig. 4a) and confocal microscopy images (Supplementary Fig. 7a, b) revealed partial colocalization of MELK with the actin cytoskeleton. Considering the critical role of F-actin reconfiguration and reorganization in various stages of the viral life cycle^16,19,20^ and its association with MELK, we hypothesized that inhibiting MELK may induce F-actin remodeling, thereby impeding coronavirus infections. To test this hypothesis, we examined the impact of inhibiting MELK activity with OTSSP167 on F-actin. STED imaging revealed a significant reconfiguration of F-actin following OTSSP167 treatment for 3 h (Fig. 4b). We then used Arivis Vision 4D software to analyze and quantify the length and fluorescence intensity of F-actin. Treatment with OTSSP167 for 3 h significantly decreased both the length and fluorescence intensity of F-actin, while β-actin (comprising both G-actin and F-actin) exhibited aggregation without a concurrent decrease in fluorescence intensity (Fig. 4c), indicating that inhibiting MELK activity with OTSSP167 induced depolymerization of F-actin. Next, we examined whether reducing MELK expression levels affected F-actin organization. Consistently, the reduction of MELK expression also induced F-actin reorganization and markedly reduced the length and fluorescence intensity of F-actin (Supplementary Fig. 7c–f). Additionally, using a biochemical fractionation assay of actin^48^, we quantified the G/F-actin ratios to assess the state of actin polymerization under OTSSP167 treatment. Consistent with our F-actin fluorescence imaging results, quantitative analysis of the fractionated lysates revealed that the F-actin pellet fraction was significantly reduced 4 h after OTSSP167 treatment, and then partially recovered after 12 h (Supplementary Fig. 7g, h). These findings indicated that inhibiting MELK, both in its activity and expression, induces the depolymerization of F-actin.

The dynamic nature of F-actin, which requires continuous polymerization and depolymerization, underlies its function in host cell processes and virus infection^16^. To investigate the effect of MELK inhibition on actin filament dynamics, we applied live-cell imaging using fluorogenic probe SiR-actin^49^. In DMSO-treated cells (Fig. 4d, e and Supplementary Movie 1), F-actin networks exhibited simultaneous polymerization and depolymerization with appropriate dynamics during the 4 h observation period. The length and fluorescence intensity of F-actin were quantified using Arivis Vision 4D and remained relatively stable (Fig. 4d, e and Supplementary Movie 2). In DMSO inset1, F-actin exhibited a gradual decrease in length and fluorescence intensity over time (Fig. 4d, e and Supplementary Movies 3, 4), while there was a gradual increase in DMSO inset2 (Fig. 4d, e and Supplementary Movies 5, 6). In contrast, F-actin in cells treated with OTSSP167 exhibited rapid aggregation followed by quick depolymerization (Fig. 4d, e and Supplementary Movies 7, 8). The decline rate of length and fluorescence intensity of F-actin was faster than that of DMSO inset1 (Fig. 4d, e and Supplementary Movies 9, 10).

Overall, MELK inhibition significantly disrupted the dynamics of F-actin by inducing depolymerization. Given the co-localization between the protein kinase MELK and F-actin, we reasoned that MELK might phosphorylate actin or certain actin-binding proteins to remodel actin filaments.

### MELK directly phosphorylates actin-depolymerizing protein cofilin-1

**Fig. 5:**
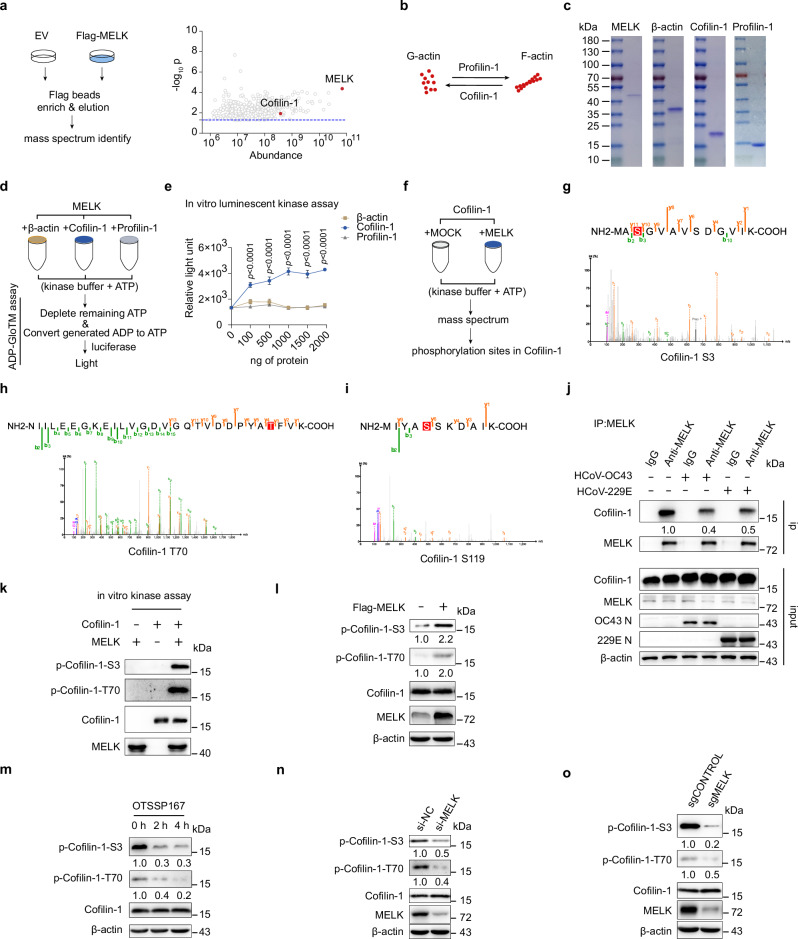
MELK directly phosphorylates actin-depolymerizing protein cofilin-1. **a** Identification of cofilin-1 associated with MELK by IP-MS. HEK293T cells were transfected with EV or FLAG-MELK expression plasmid. After 48 h, cell lysates were incubated with anti-FLAG-M2 antibody-conjugated agarose for coimmunoprecipitation. Bound proteins were eluted with 3×FLAG peptide and identified by mass spectrometry. Source data are provided as a Source Data file. **b** Schematic diagram illustrating the relationship and functions between cofilin-1 and profilin-1. **c** Expression and purification of MELK, β-actin and actin-binding proteins (cofilin-1 and profilin-1) from *E. coli*. Images were representative of three independent experiments. **d** Schematic diagram of in vitro luminescent kinase assay. **e** In vitro luminescent kinase assay with recombinant MELK (100 ng) and increasing amounts of β-actin, cofilin-1 and profilin-1. Phosphorylation of the substrate was monitored through ADP produced during the kinase reaction. Source data are provided as a Source Data file. **f** Identification of phosphorylation sites on cofilin-1 modified by MELK. The spectra of the modified peptides were identified in samples containing both MELK and cofilin-1, but not in samples containing solely cofilin-1, showing modifications at **g** S3, **h** T70, and **i** S119. **j** Viral infection decreased the interaction between MELK and cofilin-1. Huh7 cells were infected with HCoV-OC43 or HCoV-229E for 24 h. The interaction between MELK and cofilin-1 was examined by coimmunoprecipitation. The gray values were quantified using ImageJ. Images were representative of three independent experiments. Source data are provided as a Source Data file. **k** In vitro kinase assay with recombinant MELK (100 ng) and cofilin-1 (1000 ng). The phosphorylation of cofilin-1 at S3 and T70 was determined by western blot using the indicated antibodies. We generated an antibody specifically detecting cofilin-1 phosphorylation at T70. Images were representative of three independent experiments. Source data are provided as a Source Data file. **l** Overexpression of MELK enhanced the phosphorylation level of cofilin-1 at S3 and T70. Huh7 cells were transfected with EV or FLAG-MELK expression plasmid. After 24 h, the phosphorylation of cofilin-1 at S3 and T70 was determined by western blot using the indicated antibodies. The gray values were quantified using ImageJ and normalized to cofilin-1. Images were representative of three independent experiments. Source data are provided as a Source Data file. **m**–**o** MELK inhibition in both activity and expression decreased the phosphorylation level of cofilin-1 at S3 and T70. The phosphorylation of cofilin-1 at S3 and T70 was detected in **m** OTSSP167-treated, **n** siMELK-transfected and **o** sgMELK-transduced cells with indicated antibodies. The gray values as shown in (**j**). Images were representative of three independent experiments. Source data are provided as a Source Data file. Data presented in **e** are mean ± SEM of *n* = 5 independent biological replicates. Statistical significance was determined using two-way ANOVA followed by two-sided Dunnett’s multiple comparisons test (versus the 0 ng control group for each protein). Exact *p* values for Cofilin-1 at all tested concentrations are indicated in the figure. β-actin and Profilin-1 showed no significant differences at all concentrations. For β-actin, the exact *p* values versus 0 ng control were 0.1760 (100 ng), 0.1985 (500 ng), >0.9999 (1000 ng), >0.9999 (1500 ng), and 0.8204 (2000 ng). For Profilin-1, the exact *p* values versus 0 ng control were 0.9958 (100 ng), 0.7716 (500 ng), 0.9998 (1000 ng), 0.9997 (1500 ng), and 0.9949 (2000 ng). See also Supplementary Fig. 8.

To determine whether MELK is associated with certain actin-binding proteins, we performed an Immunoprecipitation-Mass Spectrometry (IP-MS) assay (Fig. 5a). Among the associated proteins, a key actin-binding protein, cofilin-1^18^, was identified (Fig. 5a). The primary function of cofilin-1 is to sever F-actin and catalyze the transition from F-actin to G-actin, whereas another important actin-binding protein, profilin-1, counteracts this effect^17,50^ (Fig. 5b). Phosphorylation plays critical roles in the functions of these two actin-binding proteins^51^, and many phosphorylation sites have been identified in actin^52^. To investigate whether MELK regulates the actin cytoskeleton through phosphorylating actin or these two actin-binding proteins, we employed recombinant MELK, actin, cofilin-1, and profilin-1 proteins, which were expressed and purified from prokaryotic expression systems (Fig. 5c). MELK was incubated individually with β-actin, cofilin-1, and profilin-1 in a kinase buffer supplemented with ATP, then the generated ADP was quantified to evaluate the phosphorylation reaction between the kinase and substrate (Fig. 5d). In the reaction tube containing MELK and cofilin-1, the generation of ADP exhibited a proportional increase with the levels of cofilin-1 protein (Fig. 5e). In contrast, the other two reactions did not show any significant changes (Fig. 5e). The results demonstrated that MELK could directly phosphorylate cofilin-1. To further identify the modified site, mass spectrometry was employed to analyze the proteins following the in vitro kinase reaction (Fig. 5f). Phosphorylation of cofilin-1 at serine 3 (S3) (Fig. 5g), threonine 70 (T70) (Fig. 5h), and serine 119 (S119) (Fig. 5i) was identified after modification by MELK. The interaction between endogenous MELK and cofilin-1 was subsequently validated using co-immunoprecipitation assays (Fig. 5j). Furthermore, their interaction was found to be attenuated upon viral infection, consistent with our findings that viral infections suppressed MELK activity. It has been established that S3 phosphorylation inhibits cofilin-1 activity by preventing binding to and severing F-actin^53,54^, while the functions of cofilin-1 phosphorylation at T70 and S119 remained unknown. We generated an antibody specifically targeting T70-phosphorylated cofilin-1. Using this antibody along with a commercially available S3-phosphorylated cofilin-1 antibody, we confirmed that MELK could phosphorylate cofilin-1 at S3 and T70 both in vitro (Fig. 5k) and in cells (Fig. 5l). Subsequently, both inhibiting MELK activity with OTSSP167 (Fig. 5m) and decreasing MELK expression (Fig. 5n, o) significantly reduced the level of S3 and T70 phosphorylated cofilin-1. The peptide competition assays confirmed that the antibody signal against both the phosphorylated peptide and the protein was specifically abolished by the phosphorylated peptide but not the non-phosphorylated counterpart, validating its high specificity for the phosphorylated epitope (Supplementary Fig. 8a–c). To validate the site specificity in a cellular context, we transfected cells with plasmids expressing Flag-tagged wild-type (WT) or T70A mutant cofilin-1. Subsequently, we enriched the cofilin-1 proteins via immunoprecipitation (IP) using anti-Flag beads. The immunoblotting results indicated the pT70 signal was detected in WT cofilin-1 but was lost in the T70A mutant (Supplementary Fig. 8d). To further validate the existence of cofilin-1 T70 phosphorylation in cells, we prepared three groups of cell samples for liquid chromatography–tandem mass spectrometry (LC-MS/MS) identification, including MOCK control, MELK overexpression, and MELK overexpression treated with OTSSP167. Based on the relative abundance revealed by extracted ion chromatograms (XIC), the T70 phosphorylation of cofilin-1 was markedly elevated in the MELK-overexpressing group. Conversely, OTSSP167 treatment effectively suppressed this phosphorylation signal (Supplementary Fig. 8e, f).

Collectively, these results suggested that MELK may remodel the actin cytoskeleton by phosphorylating cofilin-1.

### MELK inhibition induces cofilin-1 to sever actin filaments

**Fig. 6:**
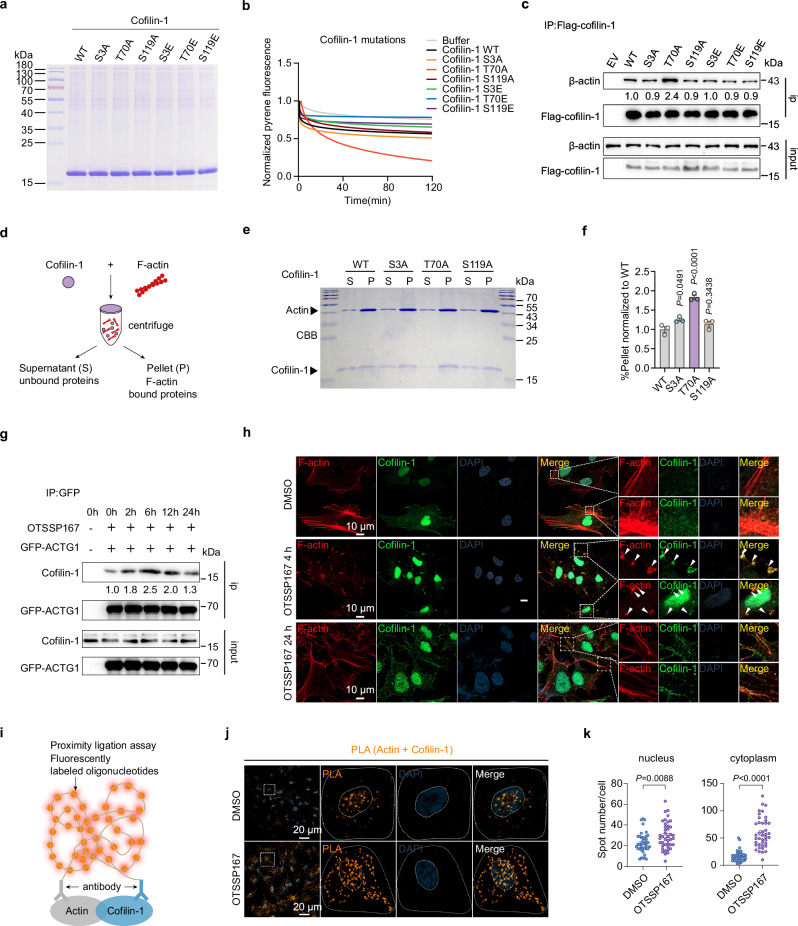
MELK inhibition induces cofilin-1 to sever actin filaments. **a** Expression and purification of WT, S3A, S3E, T70A, T70E, S119A and S119E cofilin-1 from HEK293 cells. Images were representative of three independent experiments. **b** The severance activity of cofilin-1 WT and mutants was examined using an in vitro pyrene-actin depolymerization assay. The depolymerization of F-actin was monitored by fluorescence intensity using a microplate reader. **c** The T70A mutation enhanced the interaction between cofilin-1 and actin. HEK293T cells were transfected with the indicated expression plasmids for 36 h. The interaction between cofilin-1 and actin was examined by coimmunoprecipitation. The gray values were quantified using ImageJ. Images were representative of three independent experiments. Source data are provided as a Source Data file. **d**–**f** The T70A mutation of cofilin-1 enhanced its binding to F-actin in vitro. **d** Diagram for actin co-sedimentation assay. **e** Cofilin-1 WT or mutants were incubated with F-actin for 30 min at room temperature, then were ultracentrifuged at 120,000 × *g* for 30 min. The supernatants (S) and pellets (P) were separately detected with SDS-PAGE and CBB (Coomassie Brilliant Blue) staining. **f** Quantitative analysis of (**e**). % Pellet was calculated based on the ratio of cofilin-1 density in P over that in P + S, which was then normalized to cofilin-1 WT. Images were representative of three independent experiments. Source data are provided as a Source Data file. **g** OTSSP167 enhanced the interaction between cofilin-1 and actin. The interaction between cofilin-1 and actin was examined by coimmunoprecipitation. The gray values were quantified using ImageJ. Images were representative of three independent experiments. Source data are provided as a Source Data file. **h**–**k** OTSSP167 induced colocalization between cofilin-1 and actin in situ. **h** Representative confocal microscopy images showed colocalization of cofilin-1 (green) with F-actin (AF647-phalloidin, red) in DMSO or OTSSP167-treated Huh7 cells. Nuclei were stained with DAPI (blue). **i** Diagram for proximity ligation assay (PLA) assay. **j** Colocalization between cofilin-1 and actin was quantified by PLA using the indicated antibodies. Orange immunofluorescent dots appeared when actin interacts with cofilin-1. **k** The number of orange fluorescent dots in each cell treated with DMSO or OTSSP167 was enumerated. Each dot represents an individual cell, with PLA signals quantified separately within the cytoplasm and nucleus. Images were representative of three independent experiments. Source data are provided as a Source Data file. Data presented in **f** are mean ± SEM of *n* = 3 independent biological replicates. Statistical significance was determined using one-way ANOVA followed by two-sided Dunnett’s multiple comparisons test. Exact *p* values are indicated in the figure. Data presented in **k** are mean ± SD. Each dot represents an individual cell. *n* = 40 cells for DMSO and *n* = 41 cells for OTSSP167, pooled from 3 independent biological replicates. Statistical significance was determined using two-tailed Student’s *t* test. Exact *p* values are indicated in the figure. See also Supplementary Fig. 9.

To determine the roles of cofilin-1 phosphorylation sites modified by MELK, we examined the effects of recombinant cofilin-1 proteins with corresponding site mutants (to Alanine (A): a nonphosphorylatable form; to Glutamic acid (E): a pseudophosphorylated form) on actin depolymerization in vitro (Fig. 6a). A pyrene-labeled actin fluorescence assay^55^ was used to evaluate the severing ability of these recombinant cofilin-1 proteins, including wild-type (WT) (Supplementary Fig. 9a), S3A and S3E (Supplementary Fig. 9b), T70A and T70E (Supplementary Fig. 9c), S119A and S119E (Supplementary Fig. 9d). The results showed that WT and three nonphosphorylatable form mutants (S3A, T70A and S119A) increased F-actin severing in a dose-dependent manner. In contrast, all three pseudophosphorylated form mutants (S3E, T70E and S119E) recombinant proteins exhibited restrained severance activity. Compared with WT, cofilin-1 T70A mutant exhibited the highest severance activity, followed by S3A, while S119A showed no obvious difference in activity (Fig. 6b). The phenotype of the cofilin-1 S3A mutant was consistent with previous studies^53,54^. Using a coimmunoprecipitation assay to detect the interaction between actin and cofilin-1 mutants, we found that the T70A mutant exhibited a stronger interaction with actin compared to the WT and other mutants (Fig. 6c). Furthermore, the impact of phosphorylation at S3, T70, and S119 on the ability of cofilin-1 to bind F-actin was investigated using the actin co-sedimentation assay. This well-established assay utilizes ultracentrifugation to sediment F-actin and its binding partners. F-actin was incubated with recombinant WT or mutant cofilin-1 proteins prior to centrifugation. The cofilin-1 proteins in the pellet and supernatant represented the F-actin-bound and unbound fractions, respectively (Fig. 6d). It was observed that the S3A and T70A mutants enhanced cofilin-1’s binding to F-actin, with the T70A mutant exhibiting stronger binding (Fig. 6e, f). These results demonstrated that the phosphorylation of cofilin-1 at S3 and T70 by MELK suppresses the severance activity of cofilin-1, suggesting that MELK inhibition could activate cofilin-1 to sever F-actin.

We subsequently investigated the impact of MELK inhibition on the association between cofilin-1 and actin. The coimmunoprecipitation assay demonstrated that OTSSP167 treatment enhanced the interaction between cofilin-1 and actin (Fig. 6g). Confocal microscopy revealed that after 4 h of OTSSP167 treatment, cytoplasmic cofilin-1 formed aggregates and exhibited distinct co-localization with F-actin (Fig. 6h). Compared to 4 h, F-actin exhibited partial recovery in aggregation at 24 h post-treatment, with reduced colocalization with cofilin-1. This may be important in preventing significant cytotoxicity. In addition, we conducted a proximity ligation assay (PLA) (Fig. 6i) to detect the changes in the association between cofilin-1 and actin following OTSSP167 treatment. In the PLA assay, orange immunofluorescent dots appear when cofilin-1 interacts with actin (Fig. 6j). Changes in the localization and abundance of these dots reflected binding dynamics between actin and cofilin-1. Enumerating the number of orange immunofluorescent dots in the cytoplasm and nucleus from the DMSO and OTSSP167 groups demonstrated that MELK inhibition by OTSSP167 significantly enhanced cofilin-1 binding to actin (Fig. 6k). STED imaging revealed that viral infections significantly induced F-actin rearrangement (Supplementary Fig. 9e), potentially resulting from reduced MELK activity caused by the infections. Additionally, viral infections enhanced the association between cofilin-1 and actin (Supplementary Fig. 9f, g), consistent with the effects observed following MELK inhibition with OTSSP167.

Collectively, these results demonstrated that the inhibition of MELK activates cofilin-1 and facilitates its binding to and severing F-actin.

### MELK inhibition disrupts both clathrin-dependent endocytosis and virus–membrane fusion, thereby blocking viral entry

**Fig. 7:**
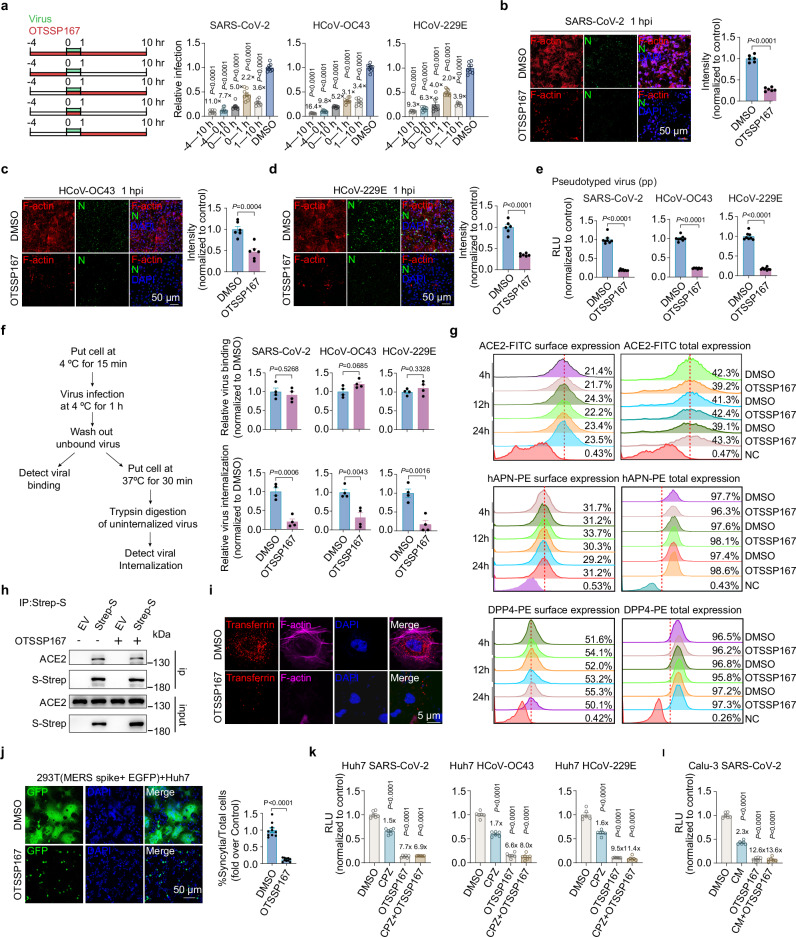
MELK inhibition disrupts both clathrin-dependent endocytosis and virus–membrane fusion, thereby blocking viral entry. **a** Time-of-addition assay. HCoV-229E, HCoV-OC43, and SARS-CoV-2 infected Huh7 cells were incubated with OTSSP167 at the time points indicated. Infection efficiency at 24 h post infection was quantified by immunostaining N protein and normalized to the DMSO-treated cells. Source data are provided as a Source Data file. **b**–**d** OTSSP167 inhibited viral entry. Representative images showing viral entry with or without OTSSP167 (0.2 μM) pre-treatment for 2 h. Huh7 cells were fixed at 1 h post infection and stained with N protein of **b** SARS-CoV-2, **c** HCoV-OC43 and **d** HCoV-229E. Quantified relative fluorescence intensity of N was normalized to DAPI and DMSO. Images were representative of three independent experiments. Source data are provided as a Source Data file. **e** Quantification of viral entry efficiency in Huh7 cells with or without OTSSP167 treatment inoculated with pseudoviruses bearing spike proteins from SARS-CoV-2. Source data are provided as a Source Data file. **f** Virus binding and internalization in DMSO-treated or OTSSP167-treated Huh7 cells. The amount of viral RNA in DMSO-treated cells was normalized to 1 (*n*  =  4). Source data are provided as a Source Data file. **g** OTSSP167 had no effect on the expression of viral receptors. The surface and total expressions of receptor ACE2, hAPN and DPP4 were detected with flow cytometry after DMSO or OTSSP167 treatment for indicated times. **h** OTSSP167 had no effect on the interaction between SARS-CoV-2 spike and ACE2. The interaction between SARS-CoV-2 spike and ACE2 was examined by coimmunoprecipitation. Images were representative of three independent experiments. Source data are provided as a Source Data file. **i** OTSSP167 inhibited the uptake of transferrin-AF555 via clathrin-mediated endocytosis. Representative images showed the human transferrin Alexa Fluor 555 entering cells with or without OTSSP167 pre-treatment for 2 h. Images were representative of three independent experiments. **j** OTSSP167 blocked virus-membrane fusion. HEK293T cells were co-transfected with MERS-CoV S and pEGFP for 24 h, then mixed with Huh7 cells for membrane–membrane fusion. After 12 h, the formation of syncytium was observed using a confocal microscope. Images were representative of three independent experiments. Source data are provided as a Source Data file. The effect of OTSSP167 combined with **k** chlorpromazine (CPZ) or **l** camostat mesylate (CM) against viral entry. CPZ:10 μM, CM: 25 μM, OTSSP167: 0.2 μM. Cells were treated with the indicated compounds 3 h before infection with pseudoviruses. Source data are provided as a Source Data file. Data presented in **a** are mean ± SEM of *n* = 8, and in **k** and **l** are mean ± SEM of *n* = 6 independent biological replicates. Statistical significance was determined using one-way ANOVA followed by two-sided Dunnett’s multiple comparisons test. Exact *p* values are indicated in the figure. Data presented in **b**–**e** are mean ± SEM of *n* = 6, in **f** are mean ± SEM of *n* = 4, and in **j** are mean ± SEM of *n* = 10 independent biological replicates. Statistical significance was determined using two-tailed Student’s *t* test. Exact *p* values are indicated in the figure. See also Supplementary Figs. 10–15.

Next, we investigated the impact of remodeled actin filaments following MELK inhibition on the viral life cycle. A time-of-drug addition assay in a single infectious cycle was used to evaluate the antiviral effect of OTSSP167 in both Huh7 (Fig. 7a) and Calu-3 cells (Supplementary Fig. 10a). We found OTSSP167 significantly blocked both entry and post-entry steps of three human coronaviruses. Notably, treatment before viral inoculation exhibited a more significant inhibitory effect (Fig. 7a and Supplementary Fig. 10a). Therefore, we speculated that MELK inhibition may interfere with the coronavirus entry stage. We then used IFA to visualize virions entering cells at 1 h post infection. Consistent with our speculation, the quantity of viral particles, including SARS-CoV-2 (Fig. 7b), HCoV-OC43 (Fig. 7c) and HCoV-229E (Fig. 7d), in OTSSP167-treated cells was significantly lower than that in control cells. To confirm this function, we challenged both Huh7 and Calu-3 cells with replication-incompetent pseudoviruses, expressing SARS-CoV-2, HCoV-OC43 or HCoV-229E spike proteins. Consistently, the infections of three pseudoviruses were significantly inhibited after OTSSP167 treatment (Fig. 7e and Supplementary Fig. 10b), or decreasing expression of MELK (Supplementary Fig. 10c–e). Furthermore, we found that OTSSP167 treatment had no effect on viral binding, but impaired viral internalization (Fig. 7f). This suggests that MELK inhibition may not affect the receptor expression or the interaction between the virus and its receptor, but instead impact subsequent stages of viral entry. Indeed, inhibiting MELK activity and expression had no effect on the surface or total expression of viral receptors (Fig. 7g and Supplementary Fig. 11a–d), or the virus-receptor interaction (Fig. 7h and Supplementary Fig. 11e). These results indicated MELK inhibition blocked viral entry mainly by impairing viral internalization. Clathrin-mediated endocytosis and membrane fusion are the two primary pathways for coronaviruses to enter cells^56,57^. Notably, the actin cytoskeleton is required for the processes of clathrin-mediated endocytosis and membrane morphology^58^. Then we found that the uptake of human transferrin-AF555 via clathrin-mediated endocytosis was significantly decreased in OTSSP167-treated cells (Fig. 7i). To examine the effect of MELK inhibition on endocytic pit dynamics, we performed live-cell Total Internal Reflection Fluorescence (TIRF) Microscopy to track the lifetime distribution of GFP-clathrin-coated pits (CCPs) and assessed cargo uptake efficiency using fluorescent transferrin. The lifecycle of a productive CCP, including initiation, maturation, and uncoating, is typically completed within 50–100 s^59,60^. Consistent with this range, CCPs in our control cells exhibited a mean lifetime of 83.5 s. However, OTSSP167 caused an extension of the mean lifetime to 243.2 s, consistent with the formation of ‘stalled’ pits observed upon actin cytoskeleton inhibition^61^ (Supplementary Fig. 12a). Additionally, we used live-cell imaging to examine transferrin uptake dynamics with AF555-transferrin. The results revealed that both OTSSP167 treatment and MELK knockdown significantly impaired the uptake efficiency of transferrin, demonstrating a functional block in clathrin-mediated endocytosis (Supplementary Fig. 12b, c). To evaluate whether MELK inhibition blocked membrane fusion, we performed a syncytium assay in which HEK293T cells co-expressing MERS-CoV spike and EGFP were co-cultured with Huh7 and Calu-3 cells. The results showed that spike-mediated cell-cell fusion was significantly blocked in OTSSP167-treated cells (Fig. 7j and Supplementary Fig. 12d–f). Then we used AP2 inhibitor, chlorpromazine (CPZ)^62^, and TMPRSS2 inhibitor, camostat mesylate (CM)^63^ to block clathrin-dependent endocytosis and virus–membrane fusion, respectively. We observed that inhibiting a single entry pathway, followed by OTSSP167 treatment, would further suppress viral entry (Fig. 7k, l). Since OTSSP167 alone already suppresses viral entry to extremely low levels, the co-treatment with CPZ or CM showed no further additive effect. These results indicated MELK inhibition disrupts both clathrin-mediated endocytosis and membrane fusion, thereby blocking viral entry.

Given the reported role of MELK in cell-cycle^64^, we analyzed cell cycle distribution using PI staining. The results showed that OTSSP167 had no significant effect on the cell cycle at 6 h or 12 h post-treatment. Consistent with a previous study^64^, G2/M arrest was observed after 24 h of treatment. Coronaviruses typically complete a replication cycle within 12 h. Our time-of-addition and pseudovirus entry assays demonstrated obvious inhibition at the early entry stage, suggesting that the antiviral effect of OTSSP167 occurs before the cell cycle perturbation (Supplementary Fig. 13a, b). Despite this early antiviral effect, our phosphoproteomic profiling was performed at 24 h, suggesting that some of the captured phosphorylation changes may reflect cell cycle perturbation associated with viral infection. We also examined the effect of OTSSP167 on innate immunity, including p-TBK1 levels and the transcription of IFNβ and ISG56. The results showed that OTSSP167 had no significant impact on these markers, indicating that its antiviral activity is independent of the innate immune signaling response (Supplementary Fig. 13c–e). Our study found MELK phosphorylates cofilin-1 to suppress the depolymerization of F-actin that is required for viral infection. Therefore, we examined the effect of cofilin-1 on viral entry. Given its roles in actin cytoskeleton dynamics, both knockdown (Supplementary Fig. 14a) and overexpression (Supplementary Fig. 14b, c) of cofilin-1 could block viral entry. Notably, cofilin-1 S3A and T70A mutants exhibited a stronger inhibitory effect. Additionally, we performed rescue experiments by co-expressing MELK with either wild-type (WT) cofilin-1 or its mutants (S3A, T70A) and assessed viral entry using three pseudoviruses (SARS-CoV-2, HCoV-229E, and HCoV-OC43). The results demonstrated that the phosphodeficient mutants (S3A, T70A) effectively rescued the enhanced viral entry caused by MELK overexpression (Supplementary Fig. 14d). Since LIM-kinases (LIMKs) are known to phosphorylate cofilin-1 at S3, we also evaluated the antiviral effect of the LIMK inhibitor R10015^65^. Our data indicated that R10015 is capable of inhibiting SARS-CoV-2 infection, but significant efficacy was observed only at concentrations greater than 10 μM (Supplementary Fig. 14e, f). Since inhibiting MELK leads to the dephosphorylation of multiple sites (S3/T70), we postulate that this induces a greater degree of cofilin-1 activation compared to LIMK inhibition, thereby causing a more significant disruption of actin-dependent viral entry. The actin cytoskeleton assembles specialized cellular structures, such as filopodia and microvilli, which are essential for multiple stages of the viral life cycle, including viral release^16,24^. Scanning electron microscopy observations revealed that OTSSP167 treatment markedly decreased the number of filopodia in cells (Supplementary Fig. 15a), and reduced the number of microvilli without affecting the abundance of cilia in proximal airway organoids (Supplementary Fig. 15b–d), indicating OTSSP167 may interfere with the later stage of the viral cycle by reducing these structures. Further, we utilized a virus-like particles (VLPs) packaging system to detect viral egress. The results showed that the VLPs released into the supernatant were significantly reduced following OTSSP167 treatment (Supplementary Fig. 15e). These findings suggested that MELK inhibition may involve multiple stages of the viral life cycle, which is consistent with the broad functions of the actin cytoskeleton in the viral life cycle^19^. Indeed, subversion of the actin cytoskeleton dynamics with Cytochalasin D and Jasplakinolide obviously blocks the infection of various viruses^25,26^ (Supplementary Fig. 15f, g), and the EC_50_ values of OTSSP167 were lower than those of these compounds.

Collectively, the suppression of actin dynamics by MELK inhibition hinders multiple stages of the viral life cycle, particularly exhibiting strong antiviral effects at the early entry stage.

## Discussion

The 518 protein kinases constitute about 1.7% of all human genes, and serve as central regulators of signal transduction in eukaryotic cells through protein phosphorylation^1^. Aberrant phosphorylation results in various disorders^5,6^. Accordingly, kinase inhibitors represent a class of promising therapeutic agents, many of which have been approved by the FDA^7^. To date, a Janus kinase inhibitor, Baricitinib, has been granted an FDA Emergency Use Authorization (EUA) for COVID-19 primarily by reducing inflammation^66,67^. However, no kinase inhibitors with antiviral capabilities have been approved for clinical treatment. For broad regulation of coronavirus infections, both common kinases and corresponding inhibitors remain unidentified. The identification of these regulators can significantly contribute to controlling current and future coronavirus epidemics.

MELK is a Ser/Thr protein kinase and belongs to the Snfl/AMPK family of serine/threonine kinase. The expression of MELK is higher in cancer cells and tissues compared to their normal counterparts, making it an attractive therapeutic target for managing cancers such as prostate cancer, hepatocellular carcinoma, ovarian cancer, and breast cancer^35–37^. The role of MELK in coronavirus infections remains unknown. The Bouhaddou study identified kinase CK2 (CSNK2A1/2) activation during SARS-CoV-2 infection^11^. Our findings verified the CK2 upregulation during SARS-CoV-2 infection, and expanded its upregulation in HCoV-OC43 infections (Fig. 1b). Nevertheless, our data revealed that there is no significant change of CK2 activity observed during HCoV-229E infection, indicating its specificity to certain viruses. In contrast, we discovered that MELK activity underwent common changes in response to SARS-CoV-2, HCoV-OC43 and HCoV-229E infections, and that MELK inhibitor OTSSP167 exhibits broad-spectrum antiviral activity against six currently circulating human coronaviruses. Interestingly, we also found that infections upregulate TBK1, a canonical innate immune kinase that protects host cells from viral invasion by triggering the expression of interferons (IFNs) and interferon-stimulated genes (ISGs). Similar to the TBK1 response, we propose that the regulation of MELK during viral infection represents a host defense strategy to resist viral invasion. Therefore, inhibition of MELK mimics and amplifies this defense mechanism. Because of the requirement for simultaneous susceptibility to all three viruses and the lack of specific p-MELK antibodies, the absence of phosphoproteomic validation in a respiratory epithelial cell line remains a limitation.

Actin cytoskeleton is strongly associated with viral infection. Our work identified a previously unknown method of actin cytoskeleton disturbance: MELK inhibition. Viral infection reconfigures and reorganizes actin cytoskeleton to optimize viral replication and virion production, and disruption of actin cytoskeleton affects every stage of viral life cycle^16,19,20^. This structure could be used for the translocation of virions to facilitate their movement towards entry sites on cell surface, including vesicular stomatitis virus (VSV), human papilloma virus (HPV), vaccinia virus (VV), HIV and SARS-CoV-2^16,21,22^. In addition, SARS-CoV-2 infection induces rearrangements of F-actin nanostructures in host pulmonary cells^23^ and microvillar reprogramming in primary nasal epithelial organoids^24^ to promote viral production and egress. Logically, small-molecule compounds that disrupt the actin cytoskeleton exhibit antiviral activity. Indeed, subversion of the actin cytoskeleton dynamics with Cytochalasin D and Jasplakinolide obviously blocks the infection of HCoV-OC43^25^ and SARS-CoV-2^26^. We firstly identified MELK inhibitor OTSSP167 as a powerful regulator of the actin cytoskeleton against multiple coronavirus infections. Noteworthily, some small-molecule drugs targeting cellular cytoskeleton have received FDA approval for clinical use in antiviral and anticancer therapies. For instance, Sabizabulin, an oral disruptor of microtubule, has been granted an FDA Fast Track designation for COVID-19^68^; Imatinib and Lapatinib, two regulators of actin filament polymerization, have been approved for cancer treatment^69,70^. Additionally, OTSSP167 has been tested in clinical trials, including phase 1 study of solid metastatic tumors and phase 2 open trials in leukemia^34^. OTSSP167 and other clinical-stage kinase inhibitors were primarily developed for cancer therapies via systemic administration. Importantly, in these oncology trials, systemic use of OTSSP167 has been associated with dose-limiting hematologic toxicities, such as thrombocytopenia and neutropenia. However, since human coronaviruses primarily target the nose, airways, and lungs in vivo, intranasal delivery allows the drug to act directly on virus-infected cells, thereby minimizing potential side effects. Indeed, inhalation is already a clinically utilized route for several respiratory antivirals. Furthermore, our pharmacokinetic data demonstrate that intranasal delivery of OTSSP167 in mice achieves a high absolute bioavailability of 97.0%. Therefore, we postulate that systemic dosing would require significantly higher overall doses to reach effective antiviral concentrations in the lungs, thereby potentially increasing the risk of adverse effects. Addressing the translational gap requires optimizing precise delivery to target cells to boost efficacy while limiting adverse effects.

The stronger phenotype observed with OTSSP167 treatment compared to MELK KD/KO is likely due to compensatory effects and delivery efficiency. OTSSP167 inhibits kinase activity upon binding and acts very rapidly (within minutes to tens of minutes). In contrast, siRNA usually needs 48–72 h to take effect, and the continuous inhibition of MELK expression by CRISPR/Cas9 is prone to induce cellular compensatory mechanisms. Additionally, the efficiency of small molecules entering cells is typically higher than that of siRNA transfection. While the scaffold-distinct MELK inhibitors MELK-8a and MELK-IN-1 effectively suppress viral infection, their EC_50_ values against viral infection are notably higher than those of OTSSP167. This discrepancy may be attributed to differences in their pharmacological properties, as their in vitro IC_50_ values for inhibiting MELK activity are also higher than those of OTSSP167. Additionally, differences in cellular permeability may also contribute to this gap. Importantly, OTSSP167 exhibits known polypharmacology and has been reported to inhibit several other host kinases, such as Aurora B and BUB1^71^. Therefore, to conclusively exclude potential off-target contributions to OTSSP167’s antiviral efficacy and to minimize potential side effects, the development of highly specific MELK-targeted inhibitors is needed.

Actin cytoskeleton has been increasingly recognized as a key mediator of host defenses, such as B-cell mediated adaptive immunity and RIG-I-like receptor (RLR)-mediated innate immunity^15,72^. Our work revealed that the inhibition of MELK impeded viral infections by disturbing the actin cytoskeleton. Interestingly, MELK activity was suppressed in response to multiple human coronaviruses, suggesting it may act as a cellular self-defense mechanism against viral infection through regulation of the actin cytoskeleton.

Cofilin-1 is a potent actin-binding protein that stimulates the severance and depolymerization of F-actin, which mediates development, tissue homeostasis, and disease, including various cancers^73,74^. Phosphorylation plays a crucial role in the activity of cofilin-1. Cofilin-1 is reportedly inactivated by phosphorylation at S3, which is modified by LIM-kinases (LIMKs) and TESK1/2^75,76^. Consistent with this, our results showed that the LIMK inhibitor R10015 could inhibit SARS-CoV-2 entry. Therefore, we speculated that TESK1/2 might also play a role in regulating viral infection. However, other key phosphorylation sites and corresponding kinases that regulate cofilin-1 function largely remain unknown. Here, we identified that MELK directly phosphorylates cofilin-1 at S3, T70 and S119. Interestingly, compared to the WT, S3A and S119A mutants, cofilin-1 T70A mutant exhibits the strongest activity in the severance of F-actin. The identification of T70 as another crucial phosphorylation site of cofilin-1 indicates that MELK may exert its functions by regulating cofilin-1 activity. Whether MELK promotes tumorigenesis by modifying cofilin-1, and whether the phosphorylation of cofilin-1 at T70 matters in diseases, especially tumors, require further investigation. The absence of pT70 in our global phosphoproteomic dataset is likely attributable to ion suppression, wherein signals from low-abundance peptides are easily eclipsed by their high-abundance counterparts. Importantly, the PhosphoSitePlus database documents several independent publications that have successfully identified endogenous cofilin-1 T70 phosphorylation, strongly supporting its physiological relevance.

In summary (Supplementary Fig. 16), we identified MELK as a common functional kinase required for multiple human coronavirus infections and unveiled a critical and previously unknown function on regulation of the actin cytoskeleton. MELK inhibition broadly impedes human coronavirus infections through disrupting the actin cytoskeleton. Notably, MELK inhibitor OTSSP167 holds great clinical promise as an effective regulator of actin cytoskeleton. Our study provides unique insights into virus-host interactions and facilitates the development of antiviral design.

## Methods

### Ethical compliance statement

All animal experiments in this study were performed in accordance with all relevant ethical regulations. The infection study protocols were reviewed and approved by the Laboratory Animal Ethics Committee of the First Affiliated Hospital of Guangzhou Medical University (Approval Nos. 2021468 and 2022038). The pharmacokinetic (PK) study was additionally reviewed and approved by the Institutional Animal Care and Use Committee (IACUC) of Shenzhen Lingfu Top Biotechnology Co., Ltd. (Approval No. TOP-1PZ-GM251203). To eliminate potential variability in susceptibility associated with sex, 6-week-old male C57BL/6 mice were utilized in this study. Mice were group-housed in IVC cages in an SPF facility. The room environment was controlled with a 12/12-h light/dark cycle, an ambient temperature of 22 ± 2 °C, and a relative humidity of 40–70%. This study involving human lung tissues was reviewed and approved by the Ethics Review Committee of the First Affiliated Hospital of Guangzhou Medical University (approval number: ES-2023-193-02). All participants provided written informed consent prior to sample collection, and all samples were anonymized before analysis.

### Cells and viruses

Huh7, HEK293T, HRT-18, Vero E6, Calu-3, and Vero 81 cells were purchased from American Type Culture Collection (ATCC). Huh7, Huh7-ACE2, HEK293T, and Vero E6 cells were cultured in Dulbecco’s modified Eagle’s medium (DMEM, Gibco, Grand Island, NY) supplemented with 10% fetal bovine serum (FBS) and penicillin/streptomycin (P/S) (100 U/mL, Gibco, Grand Island, NY). Huh7-ACE2 cells were cultured in DMEM with 10% FBS, puromycin (1 μg/ml), and penicillin-streptomycin. HRT-18 cells were cultured in Roswell Park Memorial Institute 1640 (RPMI 1640, Gibco, Grand Island, NY) supplemented with 10% FBS and penicillin/streptomycin (100 U/mL). Calu-3 cells were cultured in minimum essential medium (MEM, Gibco) supplemented with 20% FBS, 0.1 mM MEM non-essential amino acid solution (MEAA, Gibco), and penicillin/streptomycin (100 U/mL). Cell lines were authenticated and screened regularly for mycoplasma contamination using PCR-based assays, and all results were negative.

The MERS-CoV strains (EMC2012, GD01, and Nigeria) were propagated in Vero 81 and preserved in Biosafety Level 3 (BSL-3) Laboratory. The SARS-CoV-2 variants were propagated in Vero E6 and preserved in Biosafety Level 3 (BSL-3) Laboratory. All the infection experiments involving MERS-CoV and SARS-CoV-2 were performed at BSL-3 in Guangzhou Customs Inspection and Quarantine Technology Center. HCoV-229E was propagated in Huh7 cells. HCoV-OC43 was propagated in HRT-18 cells. HCoV-NL63 was propagated in Huh7-ACE2 cells. HCoV-HKU1 was propagated in human proximal airway organoids. All the infection experiments involving HCoV-229E, HCoV-OC43, HCoV-NL63, and HCoV-HKU1 were performed at BSL-2 in Guangzhou Medical University.

For viral infection assays, cells were inoculated with the virus for 1 h at 37 °C. Subsequently, the viral supernatant was removed and replaced with fresh medium. To evaluate the EC_50_ and viral replication, compounds were added concurrently with the virus. After 1 h of inoculation, the mixture was removed, and cells were washed and cultured in fresh medium. For the time-of-addition experiment, cells were treated with OTSSP167 during the indicated time, then the compound was washed away and replaced with fresh medium. In the pseudovirus entry assay, cells were pre-treated with the compounds for 1 h. The drug was removed by washing prior to the inoculation with pseudoviruses, and luciferase activity was measured at 24 h post-infection.

### Reagents and antibodies

Mouse anti-Flag antibody M2-conjugated agarose (catalog no. A2220) and the Duolink In Situ–Fluorescence kit for PLA (catalog no. DUO92008, DUO92002, DUO92004) were purchased from Sigma-Aldrich. Steady-Glo luciferase assay (catalog no. E2520) and ADP-Glo™ Kinase Assay (catalog no. V6930) were obtained from Promega. SiR-actin (catalog no. CY-SC001) and the Actin Polymerization Biochem Kit (catalog no. BK003) were purchased from Cytoskeleton. Alexa Fluor™ 647 phalloidin (catalog no. A22287) was purchased from Invitrogen.

Rabbit antibodies against MERS-CoV N protein (catalog no. 40068-RP02), SARS-CoV-2 N protein (catalog no. 40143-R004), HCoV-229E N protein (catalog no. 40640-T62), HCoV-OC43 N protein (catalog no. 40643-T62), HCoV-NL63 N protein (catalog no. 40641-T62), and HCoV-HKU1 N protein (catalog no. 40642-T62) were obtained from Sino Biological. Rabbit antibodies against human p-TBK1 Ser172 (catalog no. 5483), TBK1 (catalog no. 3504), Flag (catalog no. 14793S), β-actin (catalog no. 4970S), Cofilin-1 (catalog no. 5175S), and p-Cofilin-1 S3 (catalog no. 3313S) were obtained from Cell Signaling Technology. Rabbit antibodies against MELK (catalog no. ab273015) were obtained from Abcam. Mouse antibodies against β-actin (catalog no. 3700S) were obtained from Cell Signaling Technology. We generated a custom polyclonal antibody against the pT70 epitope of cofilin-1. Rabbits were immunized with the phosphopeptide C-TVDDPYA(pT)FVKM. The obtained serum underwent specific affinity purification followed by negative selection against the non-phosphorylated peptide (C-TVDDPYATFVKM) to eliminate non-specific binding. The antibody was purified by cross-adsorbing against the non-phosphorylated peptide to remove non-specific binders. Enzyme-Linked Immunosorbent Assay (ELISA) validation subsequently confirmed that the purified antibody specifically recognizes the phosphorylated epitope. Primary antibodies were used at a 1:1000 dilution for Western Blot (WB) and a 1:100 dilution for immunofluorescence (IFA) assays.

### Proteomics and phosphoproteomics

Huh7 cells were infected with SARS-CoV-2, HCoV-229E, HCoV-OC43 for 24 h, respectively, or with MOCK. An appropriate amount of protein from each sample was pooled into a combined sample for the Spectral Library. Twenty micrograms of protein from each original sample were analyzed with SDS-PAGE electrophoresis to assess the consistency between samples. All samples, including the pooled sample, were digested with trypsin. DTT was added to a final concentration of 20 mM, incubated at 30 °C for 2 h. IAA was added to a final concentration of 25 mM, shaken at 600 rpm for 1 min, and incubated in the dark at room temperature for 30 min. NH_4_HCO_3_ buffer (50 mM) was added to dilute the urea concentration below 1.5 M. Then, 40 µL of NH_4_HCO_3_ buffer (1:100 ratio of Lys-C) was added, shaken at 600 rpm for 1 min, and incubated at 37 °C for 4 h. Trypsin was added at a 1:50 ratio and incubated at 37 °C for 16 h. The pooled sample was desalted using a C18 cartridge, followed by freeze-drying and reconstitution with the High Select™ Fe-NTA Phosphopeptide Enrichment Kit (Thermo Scientific, A32992). Peptide concentration was measured using OD280 and reconstituted in 0.1% FA. The pooled sample was fractionated using the HPRP method, and all fractions were collected. Each fraction was freeze-dried, reconstituted in 10 µL of 0.1% FA, and the peptide concentration was measured using OD280. Two micrograms of peptides from each fraction were mixed with iRT (Biognosys Ki-3002) standard peptides and analyzed by DDA (90 min, each fraction, voltage 1500 V, ion source temperature 180 °C, drying gas 3 L/min, PASEF cycles: 8, TIMS accumulation time: 100 ms, TIMS range: 0.75–1.35, MS and MS/MS range: 300–1700 Da, dynamic exclusion time: 24 s). DDA data were directly imported into Spectronaut to construct the Spectral Library (enzyme: trypsin, max missed cleavage sites: 1). Identified proteins must pass the filtering criteria: FDR < 1%, phosphorylation localization probability cutoff >0.9. All downstream analyses (including kinase activity inference) utilized only Class I sites (>0.9 localization probability). For the detection samples, desalting was performed using a C18 cartridge, followed by freeze-drying and reconstitution with the High Select™ Fe-NTA Phosphopeptide Enrichment Kit (Thermo Scientific, A32992). Peptide concentration was measured using OD280. Enriched phosphopeptides were reconstituted in 0.1% FA. A total of 400 ng of peptides from each sample were mixed with iRT (Biognosys Ki-3002) standard peptides and analyzed with DIA (90 min). Chromatographic separation was performed using a nanoElute HPLC system with nanoflow rates (solution A: 0.1% formic acid in water and solution B: 0.1% formic acid in acetonitrile). Separated samples were analyzed using a timsTOF mass spectrometer (Bruker) in DIA-PASEF mode (positive ions, MS and MS/MS scan range: 300–1700 m/z. MS2 data acquisition uses DIA-PASEF mode with 8 TIMS scan windows, each with an accumulation time of 100 ms). All DIA data were analyzed using the established library and Spectronaut software, with the same database as used for library construction (retention time prediction type: dynamic iRT, MS2 level interference correction: enabled, cross-run normalization: enabled). All results must pass the filtering criteria: Q value cutoff of 0.01. Changes in kinase activity were estimated by Kinase-Substrate Enrichment Analysis (KSEA) by measuring and averaging the amounts of the identified substrates^77^.

### Kinase inhibitors library screening

The kinase inhibitors library containing 1805 compounds was purchased from MedChemExpress. All compounds were diluted with DMEM to a 1 μM working concentration. Huh7 cells were seeded into 96-well plates (655090, Greiner) at a density of 3.5 × 10^4^ cells/well. After 12 h, each well was treated with either 1 μM of inhibitor drug or DMSO control, concurrently with respective infections (SARS-CoV-2: MOI = 1, HCoV-229E: MOI = 0.1, HCoV-OC43: MOI = 1). The specific MOI for each virus was optimized based on its distinct replication kinetics in Huh7 cells. After 12 h, cells were fixed with 4% paraformaldehyde for IFA. Viral-infected cells were visualized by staining viral N proteins with indicated antibodies. All cells were stained with nuclear dye DAPI. The images of the whole well were captured and analyzed for infection rate with a Celigo Image Cytometer. The effect of the inhibitor on viral infection was reflected by the fold change in infection rate compared to the DMSO control.

### Cell viability assay

Cytotoxicity was assessed using the Cell Counting Kit-8 (CCK8) assay (Beyotime), according to the manufacturer’s instructions. The assay was conducted in uninfected Huh7 cells with the same compound dilutions and concurrently with the viral replication assay. All assays were performed in biologically independent triplicates.

### Human proximal airway organoids

The human proximal airway organoids were established using biopsy samples of normal human lung tissues obtained from patients undergoing surgical operations at the First Affiliated Hospital of Guangzhou Medical University. All donors had been provided with written consent, which was approved by the Institutional Review Board of the First Affiliated Hospital of Guangzhou Medical University. Human proximal airway organoids were embedded in 3D Matrigel and maintained in an expansion medium. After being mechanically sheared into fragments and seeded in transwell inserts until reaching approximately 90% cell confluence, organoids were induced to differentiate by replenishing with differentiation medium for 14 days at 37 °C in a humidified incubator with 5% CO_2_^78^.

### siRNA transfection in human proximal airway organoids

Accell-siRNA SMARTPool Human Non-Targeting Control Pool (D-001910-10-50) and Accell Human MELK (9833) siRNA–SMARTpool (E-004029-00-0005) were obtained from Dharmacon/Horizon Discovery. siRNAs were dissolved to a concentration of 100 mM using siRNA buffer (Dharmacon, B-002000-UB-100). Passive transfection was carried out by introducing the Accell-siRNA to a final concentration of 1 mM into transwell for 2 weeks. This siRNA concentration was maintained by supplementing with fresh siRNA during each medium change^24^.

### CRISPR-Cas9-mediated genome editing

The guide RNAs (sgRNAs) targeting human MELK were designed using an online design tool (CRISPick). After annealing, the double-stranded oligos were inserted into the lentiCRISPR v2 vector after cleavage by BsmBI. For lentivirus production, the constructed plasmids were co-transfected with packaging plasmids (psPAX2 and pMD2.G) into HEK293T cells using Lipofectamine 2000. Viral supernatants were collected at 48 and 72 h post-transfection and filtered. Huh7 cells were infected with the lentiviral supernatants in the presence of polybrene and selected using puromycin (1 μg/ml) starting at 48 h post-infection. After one week of selection, the knockout efficiency was verified by Western blot with an antibody against MELK. The sgRNA sequences are listed in Supplementary Table 2.

### RNA isolation and quantitative PCR

Total RNA from cells or tissues was extracted using TRIzol Reagent (Invitrogen, 15596-026) in accordance with the manufacturer’s protocol. The viral RNA from cell supernatant was isolated using EasyPure^®^ Simple Viral DNA/RNA Kit (Transgen, ER211-01) according to the manufacturer’s protocol. For viral genome copy detection, real-time PCR was performed using the EZProbe One Step qRT-PCR Probe Kit (ROX2 plus). For cellular gene expression detection, mRNA was reverse-transcribed to cDNA using PrimeScript RT reagent Kit with genomic DNA Eraser (Takara Biomedical Technology catalog no. RR047A), and qPCR was performed using 2 × Color SYBR Green qPCR Master Mix (ROX2 plus) (EZB, A0012-R2). All qPCR reactions were conducted using the QuantStudio 6 Flex Real-Time PCR System. All the primer sequences are listed in Supplementary Table 2.

### Co-immunoprecipitation (Co-IP)

The cDNA encoding the full-length SARS-CoV-2 Spike (S) protein (Wuhan-Hu-1 strain) was codon-optimized and cloned into the pcDNA3.1 vector with a Strep-tag fused to the C-terminus. HEK293T cells were transfected with the S-Strep plasmid using Lipofectamine 2000. After 48 h, cells were harvested and lysed in lysis buffer [50 mM Tris-HCl (pH 7.4), 150 mM NaCl, 0.5% Triton X-100, and protease inhibitor cocktail] for 30 min at 4 °C. The lysates were clarified by centrifugation at 13,000 rpm for 15 min at 4 °C. The supernatants were incubated with Streptavidin Agarose beads (Beyotime, P2159) for 4 h at 4 °C to obtain the S-Strep protein. The beads were washed three times with lysis buffer to remove unbound proteins. Subsequently, the S-Strep-bound beads were incubated with lysates from Huh7 cells (containing endogenous ACE2) in the presence of 1 μM OTSSP167 or DMSO (vehicle control) overnight at 4 °C with rotation^79^. The beads were then washed five times with lysis buffer. Bound proteins were eluted by boiling in SDS loading buffer for 10 min and analyzed by immunoblotting with antibodies against ACE2 and Strep.

### Focus forming assay (FFA)

Cells were seeded in 96-well plates at a density of 3.5 × 10^4^ cells/well: Vero E6 cells for SARS-CoV-2, HRT-18 cells for HCoV-OC43, Huh7 cells for HCoV-229E. After 12 h, virus cultures or tissue homogenates were serially diluted and used to inoculate cells at 37 °C for 1 h. The inocula were then removed before adding 100 μl medium containing 1.6% (SARS-CoV-2) or 1.2% (HCoV-OC43 and HCoV-229E) carboxymethylcellulose per well. After 24 h, cells were fixed with 4% paraformaldehyde and permeabilized with 0.2% Triton X-100. Cells were then incubated with indicated antibodies against viral N proteins at 37 °C for 1 h, followed by an HRP-labeled goat anti-rabbit secondary antibody (Jackson ImmunoResearch Laboratories) at 37 °C for 1 h. The foci were visualized using TrueBlue Peroxidase Substrate (KPL, Gaithersburg, MD), and enumerated with an ELISPOT reader (Cellular Technology Ltd., Cleveland, OH). Viral titers were calculated as FFU per ml or per gram.

### Immunofluorescence and histology

To observe the viral infection or subcellular localization in cells or organoids, Huh7 cells were seeded in 96-well plates (655090, Greiner) or on coverslips in 24-well plates, and human proximal airway organoids were seeded in transwell inserts. After infection or compounds treatments, the cells or organoids were fixed with 4% paraformaldehyde for 15 min at room temperature, and then permeabilized with 0.2% Triton X-100 in phosphate-buffered saline (PBS) for 10 min at room temperature, followed by blocking with goat serum (Cwbio CW0130S) for 30 min at room temperature. Cells or organoids were then incubated with indicated primary antibodies in phosphate-buffered saline, 0.1% Tween 20 (PBST) overnight at 4 °C, followed by incubation with indicated Alexa Fluor secondary antibodies and DAPI. Cells in 96-well plates were imaged using the Celigo Image Cytometer. Cells on coverslips were imaged using facility line (Abberior) super-resolution stimulated-emission-depletion (STED) microscopy and Zeiss LSM 980 confocal microscope. For Abberior STED imaging, excitation was performed with a 640 nm pulsed laser (20.3% power). Super-resolution was achieved using a 775 nm STED depletion laser at 10% power. The fluorescence signal was detected by avalanche photodiodes (APDs) in the spectral range of 650–755 nm. To reject confocal background, time-gated detection was employed with a gate start of 0.75 ns and a width of 8 ns. The viral infection in organoids was imaged using a Zeiss LSM 980 confocal microscope. The microvilli in organoids were imaged using Carl Zeiss Elyra 7 with Lattice SIM Super Resolution confocal microscope. The infected mice tissues were fixed in 10% formalin, embedded in paraffin and sectioned at 5 μm. Antigen retrieval was performed using antigen unmasking solution. The samples were incubated with indicated primary antibodies in PBST overnight at 4 °C, followed by incubation with indicated Alexa Fluor secondary antibodies and DAPI. Images were captured with Zeiss LSM 980 confocal microscope.

### Recombinant proteins

Recombinant human MELK (D3-V330) (catalog no. HY-P701811), cofilin-1 (M1-L166) (catalog no. HY-P75682), and β-actin (D2-F375) (catalog no. HY-P7453), expressed in *E. coli*, were purchased from MedChemExpress. Profilin-1 recombinant protein (catalog no. PL7211) was obtained from ECM Biosciences. Human cDNAs encoding cofilin-1 WT and mutants were cloned into PTT5-His vector. The plasmids were then transformed into HEK293 cells, and the recombinant proteins were purified with HisPur Ni–nitrilotriacetic acid resin.

### In vitro kinase assays

For the in vitro luminescent kinase assay, recombinant activated MELK (100 ng) was incubated with recombinant cofilin-1, β-actin, and profilin-1 proteins (100 ng, 500 ng, 1000 ng, 1500 ng, and 2000 ng) for 60 min at 30 °C in the kinase buffer (40 mM Tris,7.5; 20 mM MgCl2; 0.1 mg/ml BSA; 50 μM DTT; 50 μM ATP). The kinase reaction was measured using an ADP-Glo™ Kinase Assay (Promega). The remaining ATP after the completion of the kinase reaction was then depleted, followed by the conversion of ADP to ATP. The newly synthesized ATP was quantified using a luciferase/luciferin reaction. For mass spectrometry analysis or western blot, the protein samples were performed peptide extraction or SDS-PAGE electrophoresis, respectively.

### Live-cell imaging

For live-cell imaging, 2 × 10^4^ Huh7 cells were seeded into a 35 mm glass-bottomed dish (MatTek Corporation, Ashland, USA). After 12 h, cells were incubated with SIR-actin (100 nM, Cytoskeleton) for 4 h at 37 °C with 5% CO_2_ to visualize F-actin, followed by OTSSP167 (0.2 μM) or DMSO treatment. Live-cell imaging was performed using a Zeiss LSM 980 laser scanning confocal microscope equipped with an Axio Observer 7 inverted stand. Cells were imaged using a Plan-Apochromat 63×/1.40 NA Oil DIC objective. Fluorescence images (Cy5 channel) were acquired using a 639 nm laser (0.4% power) with a pinhole set to 1.00 AU (66 μm). Images were captured at a resolution of 2048 × 2048 pixels with a pixel size of 0.066 μm and an 8-bit depth. Time-lapse sequences were recorded for a total duration of 3 h, with images acquired every 2 min. The scan speed was set to 6 (pixel dwell time: 1.02 μs) with unidirectional scanning mode. All live cell imaging data were further analyzed by Arivis Vision 4D for quantification of F-actin length and intensity. Preprocessing: to improve signal-to-noise ratio, raw images in the Cy5 channel were processed using a Discrete Gaussian filter with a diameter of 0.512 μm for denoising. Punctate structure detection: structures were identified using the Shape Detection operation. Objects were defined as bright spheres with a diameter range of >0.07 μm. Filamentous structures were reconstructed using the Neurite Tracer module based on the Probabilistic Reconstructor algorithm. The specific segmentation parameters were set as follows: Branch diameter: 0.132–0.986 μm, Seeding parameters: tubularity local threshold of 0.1000 with a seed filter of 80%. Post-processing: To reduce artifacts, terminal sections with a length less than 0.263 μm were excluded.

To analyze clathrin-mediated endocytosis dynamics, Huh7 cells expressing GFP-Clathrin were treated with DMSO or OTSSP167 (0.2 µM) for 4 h. Live-cell imaging of CCP lifetimes was performed on a Carl Zeiss Elyra 7 system in TIRF mode at 37 °C with 5% CO_2_. For cargo uptake assays, treated cells were incubated with Alexa Fluor 555-conjugated transferrin and imaged using a Zeiss LSM 980 confocal microscope. All image data were processed using Fiji (ImageJ) to quantify the mean lifetime of surface CCPs and the intracellular fluorescence intensity of internalized transferrin, respectively.

### F-actin co-sedimentation assay

F-actin co-sedimentation assay was performed as described previously, with minor modifications^53^. Briefly, 2 mg/ml actin from rabbit muscle (BK003, Cytoskeleton) was polymerized for 60 min at room temperature in 10× polymerization buffer containing 0.2 M Tris-HCl (pH 7.5), 1 M KCl, 20 mM MgCl2, and 1 mM dithiothreitol. Purified cofilin-1 protein (5 μg of either wild type or mutants) was incubated with 10 μg of F-actin in 200 μl of 10 mM Tris-HCl (pH 8.0) for 30 min at room temperature. Then, the mixtures were ultracentrifuged at 120,000 × *g* at 4 °C for 30 min. The supernatants and pellets were separately subjected to SDS-PAGE and stained with Coomassie brilliant blue (CBB).

### Proximity ligation assay (PLA)

PLAs were used to examine the interaction of cofilin-1 and actin using the Duolink In Situ Fluorescence kit (Sigma-Aldrich, DUO92008, DUO92002, and DUO92004) according to the manufacturer’s protocol. In Fig. 6, Huh7 cells were cultured on coverslips in 24-well plates with OTSSP167 (0.2 μM) or DMSO treatment for 3 h. Cells were fixed and permeabilized as described in the “Immunofluorescence and histology” section. The cells were then blocked with Duolink blocking buffer in a preheated humidity chamber for 30 min at 37 °C and incubated with indicated primary antibodies (Rabbit anti-cofilin-1, 1:500; Mouse anti-β-actin, 1:500) in antibody diluent overnight at 4 °C, followed by incubation with probe anti-rabbit PLUS and probe anti-mouse MINUS for 60 min at 37 °C. PLA probes in close proximity would form a closed circle in ligation-ligase solution for 30 min at 37 °C, and the fluorescent signal was amplified in amplification solution for 100 min at 37 °C. The fluorescent spots were imaged with Zeiss LSM 980 confocal microscope.

### Pseudotyped virus

SARS-CoV-2, HCoV-OC43 and HCoV-229E pseudotyped viruses were generated using vesicular stomatitis virus (VSV) pseudotyped system. The pcDNA3.1 vector carrying SARS-CoV-2 S, HCoV-OC43 S and HCoV-229E S were transfected into HEK293T cells for 24 h. Cells were then infected with rVSV-ΔG-luc, and supernatant containing pseudotyped virus was harvested 24 h post infection. Huh7 cells were seeded into 96-well plates. After 12 h, cells were incubated with SARS-CoV-2, HCoV-OC43 and HCoV-229E pseudotyped viruses, respectively, for 24 h. Luciferase activity was measured using the steady-Glo luciferase assay system (Promega).

### Virus-like particles (VLPs)

The SARS-CoV-2 S (spike), E (envelope), M (membrane), and N (nucleocapsid) genes were cloned into human expression plasmids. HEK293T cells were co-transfected with these plasmids for 48 h. Following transfection, virus-like particles (VLPs) released into the culture supernatants were collected and analyzed by western blot using indicated antibodies.

### Reporting summary

Further information on research design is available in the Nature Portfolio Reporting Summary linked to this article.

## Supplementary information

Supplementary Information

Description Of Additional Supplementary File

Supplementary Data 1

Supplementary Data 2

Supplementary Movie 1

Supplementary Movie 2

Supplementary Movie 3

Supplementary Movie 4

Supplementary Movie 5

Supplementary Movie 6

Supplementary Movie 7

Supplementary Movie 8

Supplementary Movie 9

Supplementary Movie 10

Reporting summary

Transparent Peer Review file

## Source data

Source data

## Data Availability

The mass spectrometry proteomics data generated in this study have been deposited in the ProteomeXchange Consortium via the PRIDE partner repository under accession codes PXD057194 (Global proteomics), PXD057224 (Global phosphoproteomics), PXD057095 (IP-MS), and PXD074164 (Cofilin-1 T70 phosphorylation). The processed proteomics data generated in this study are provided in the Supplementary Information/Source Data file. All other data supporting the results of this manuscript are available in the article or its Supplementary Information. Source data for all figures are provided with this paper. Source data are provided with this paper.
