# Supplementary material for: MELK inhibition disrupts actin cytoskeleton and broadly restricts human coronavirus infections
Source: Nat Commun. 2026 May 6;17:6098. doi: 10.1038/s41467-026-72615-1 (PMC13358123; doi:10.1038/s41467-026-72615-1)

MELK inhibition disrupts actin cytoskeleton and broadly restricts human coronavirus infections

Kuai Yu[1#], Qiaorui Yao[1,2#], Dong Wang[1,3#], Qingtao Hu[1,3#], Dandan Li[1#], Fang Li[1], Fenghua Chen[1], Jingyi Su[1], Ting Huang[1], Qing Zhang[4], Zishuo Lin[5], Wei Ran[1], Yiliang Wang[1], Yuzheng Zhou[6], Shuai Wen[1], Yuting Lin[1], Yaming Jiu[7,8]*, Jingxian Zhao[1,2]*, Jincun Zhao[1,2,6,9]*

1. State Key Laboratory of Respiratory Disease, National Clinical Research Centre for Respiratory Disease, National Centre for Respiratory Medicine, Guangzhou Institute of Respiratory Health, the First Affiliated Hospital of Guangzhou Medical University, Guangzhou Medical University, Guangzhou, Guangdong Province 510182, China.
2. Guangzhou National Laboratory, No. 9 XingDaoHuanBei Road, Guangzhou International Bio Island, Guangzhou, Guangdong Province 510005, China.
3. GMU-GIBH Joint School of Life Sciences, The Guangdong-Hong Kong-Macao Joint Laboratory for Cell Fate Regulation and Diseases, Guangzhou Medical University, Guangzhou, Guangdong Province 510182, China.
4. Proteomics and Metabolomics Core Facility, Guangzhou National Laboratory, No. 9 XingDaoHuanBei Road, Guangzhou International Bio Island, Guangzhou, Guangdong, 510005, China.
5. School of Basic Medical Sciences, Guangzhou Medical University Guangzhou, Guangdong, 511436, China.
6. Institute for Hepatology, National Clinical Research Center for Infectious Disease, Shenzhen Third People's Hospital, The Second Affiliated Hospital, School of Medicine, Southern University of Science and Technology, Shenzhen, Guangdong, 518112, China.
7. Unit of Cell Biology and Imaging Study of Pathogen Host Interaction, Key Laboratory of Molecular Virology and Immunology, Shanghai Institute of Immunity and Infection, Chinese Academy of Sciences, Shanghai, 200031, China.
8. University of Chinese Academy of Sciences, Yuquan Road No. 19(A),

Shijingshan District, Beijing, 100049, China.
9. Shanghai Institute for Advanced Immunochemical Studies, School of Life Science and Technology, Shanghai Tech University, Shanghai, 201210, China.
* Corresponding author
E-mail address:
zhaojincun@gird.cn (Jincun Zhao)
zhaojingxian@gird.cn (Jingxian Zhao)
ymjiu@siii.cas.cn (Yaming Jiu)
# Kuai Yu, Qiaorui Yao, Dong Wang, Qingtao Hu and Dandan Li contributed equally to this work.

Supplementary Fig.1. Quality control and Gene Ontology enrichment of proteomics and phosphoproteomics

Supplementary Fig. 2. TBK1 is activated during coronavirus infections

Supplementary Fig. 3. MELK inhibition blocks the infection of SARS-CoV-2 VOCs

Supplementary Fig. 4. Broad restriction of various MERS-CoV strains by MELK inhibition and cytotoxicity analysis of OTSSP167

Supplementary Fig. 5. Antiviral activity of other MELK inhibitors and confirmation of OTSSP167 target engagement

Supplementary Fig. 6. Evaluation of the therapeutic potential of OTSSP167

Supplementary Fig. 7. Inhibition of MELK induces the depolymerization of F-actin

Supplementary Fig. 8. Validation of the p-Cofilin-1-T70 antibody and cofilin-1 T70 phosphorylation

Supplementary Fig. 9. Characterization of Cofilin-1 depolymerization activity and its association with actin upon viral infection

Supplementary Fig. 10. Inhibition of MELK suppresses coronavirus infections in both Calu-3 and Huh7 cells

Supplementary Fig. 11. MELK inhibition does not affect viral receptor expression or Spike-ACE2 interaction

Supplementary Fig. 12. MELK inhibition impairs clathrin-mediated endocytosis and spike-mediated membrane fusion

Supplementary Fig. 13. The antiviral activity of OTSSP167 is independent of cell cycle perturbation and innate immune responses

Supplementary Fig. 14. Cofilin-1 regulates the entry of coronaviruses

Supplementary Fig. 15. OTSSP167 reduced the release of virus-like particles

Supplementary Fig. 16. Model

Supplementary Table 1. EC50 and Selectivity Indices

Supplementary Table 2. Primer or oligo sequences (5 - 3) used in this study

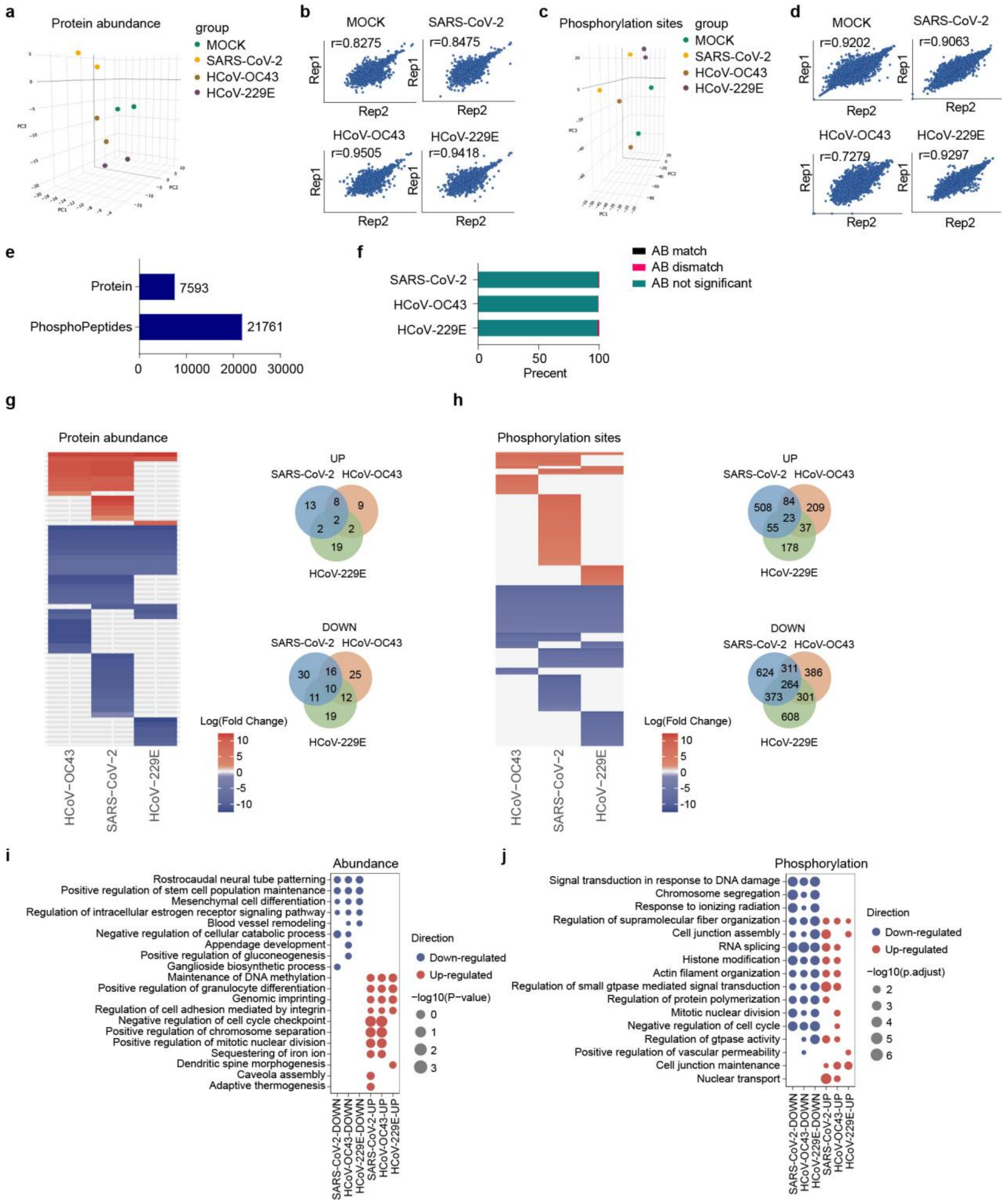

a
Protein abundance
group
MOCK
SARS-CoV-2
HCoV-OC43
HCoV-229E
b
MOCK
SARS-CoV-2
r=0.8275
r=0.8475
HCoV-OC43
HCoV-229E
r=0.9505
r=0.9418
Rep1
Rep2
c
Phosphorylation sites
d
r=0.9202
r=0.9063
r=0.7279
r=0.9297
e
Protein
7593
PhosphoPeptides
21761
0
10000
20000
30000
f
AB match
AB dismatch
AB not significant
0
50
100
Precent
g
Protein abundance
UP
DOWN
Log(Fold Change)
h
Phosphorylation sites
i
Abundance
Rostrocaudal neural tube patterning
Positive regulation of stem cell population maintenance
Mesenchymal cell differentiation
Regulation of intracellular estrogen receptor signaling pathway
Blood vessel remodeling
Negative regulation of cellular catabolic process
Appendage development
Positive regulation of gluconeogenesis
Ganglioside biosynthetic process
Maintenance of DNA methylation
Positive regulation of granulocyte differentiation
Genomic imprinting
Regulation of cell adhesion mediated by integrin
Negative regulation of cell cycle checkpoint
Positive regulation of chromosome separation
Positive regulation of mitotic nuclear division
Sequestering of iron ion
Dendritic spine morphogenesis
Caveola assembly
Adaptive thermogenesis
Direction
Down-regulated
Up-regulated
−log10(P−value)
SARS-CoV-2-DOWN
HCoV-OC43-DOWN
HCoV-229E-DOWN
SARS-CoV-2-UP
HCoV-OC43-UP
HCoV-229E-UP
j
Phosphorylation
Signal transduction in response to DNA damage
Chromosome segregation
Response to ionizing radiation
Regulation of supramolecular fiber organization
Cell junction assembly
RNA splicing
Histone modification
Actin filament organization
Regulation of small gtpase mediated signal transduction
Regulation of protein polymerization
Mitotic nuclear division
Negative regulation of cell cycle
Regulation of gtpase activity
Positive regulation of vascular permeability
Cell junction maintenance
Nuclear transport
−log10(p.adjust)

Supplementary Fig. 1. Quality control and Gene Ontology enrichment enrichments of proteomics and phosphoproteomics

a, c, Principal-component analysis (PCA) of protein abundance (a) and phosphorylation sites (c).

b, d, Pearson correlation analysis between replicates of proteomics (b) and phosphoproteomics (d).

e, The number of detected quantifiable proteins and phosphorylation sites (>0.9 localization probability).

f, Percent of significantly regulated phosphorylation sites with same direction (AB match) or opposite direction (AB mismatch) of significant or insignificant (AB not significant) changes in protein abundance.

g, h, All analyses utilized the limma R package with empirical Bayes moderation, which incorporates all biological replicate measurements in linear models and stabilizes variance estimates across features. For changes in protein abundance and phosphorylated sites, p-values were adjusted using the Benjamini-Hochberg false discovery rate (FDR) method, with FDR < 0.05 as the significance threshold. Heat map showed the proteins with significant changes in abundance (g) and phosphorylation levels (h) upon infection, and the related number was presented with Venn diagram. In the heatmaps, each row on the y-axis represents an individual differentially expressed protein (g) or significantly changed phosphorylation site (h).

i, j, Gene ontology enrichment analysis of proteins with significant changes in abundance (i) and phosphorylation levels (j). P-values were calculated using the empirical Bayes method in limma (abundance), and adjusted for multiple testing using the Benjamini-Hochberg procedure (FDR) (phosphorylation levels).

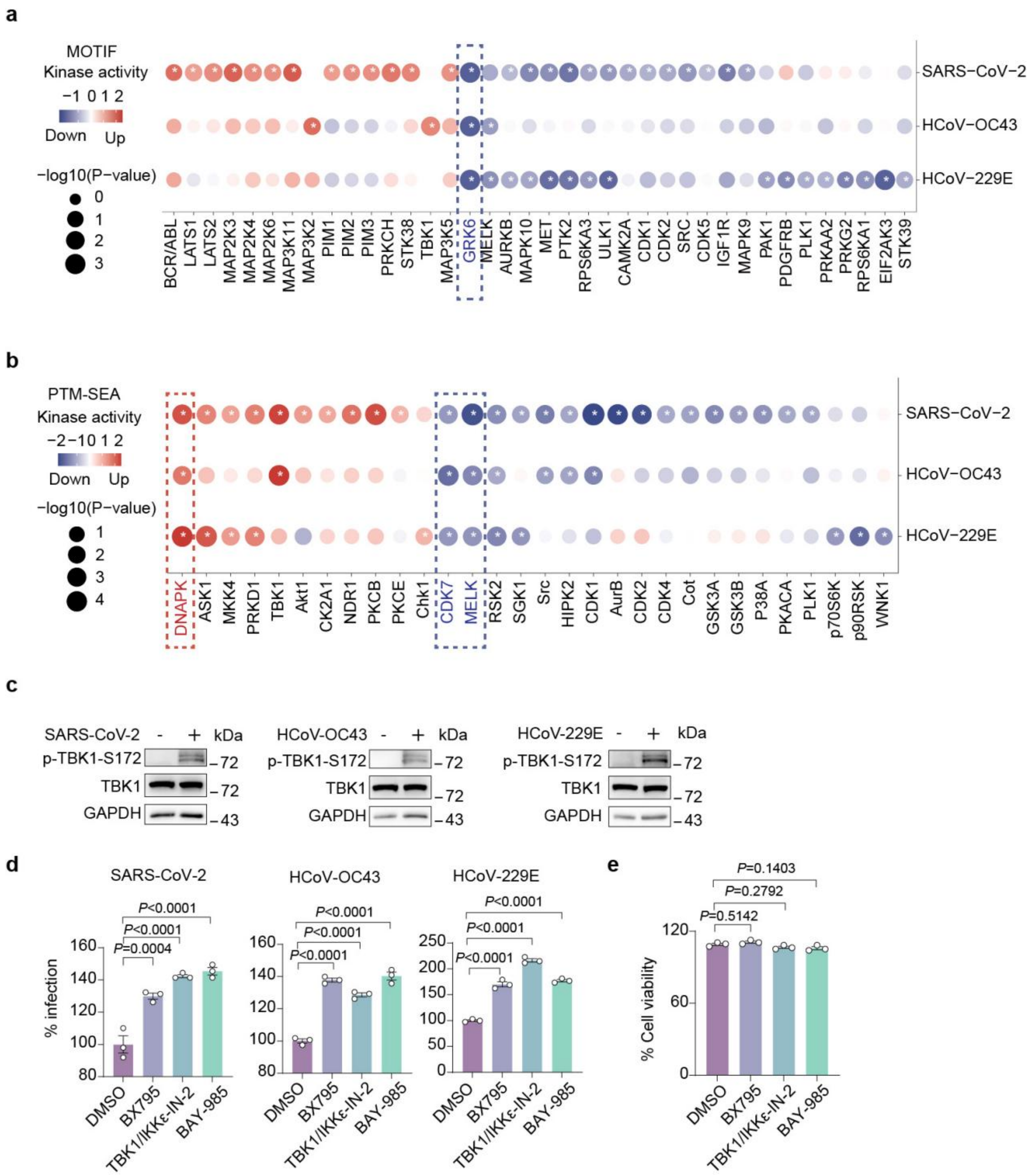
a
MOTIF
Kinase activity
−1 0 1 2
Down Up
−log10(P−value)
0
1
2
3
SARS−CoV−2
HCoV−OC43
HCoV−229E
BCR/ABL
LATS1
LATS2
MAP2K3
MAP2K4
MAP2K6
MAP3K11
MAP3K2
PIM1
PIM2
PIM3
PRKCH
STK38
TBK1
MAP3K5
GRK6
MELK
AURKB
MAPK10
MET
PTK2
RPS6KA3
ULK1
CAMK2A
CDK1
CDK2
SRC
CDK5
IGF1R
MAPK9
PAK1
PDGFRB
PLK1
PRKAA2
PRKG2
RPS6KA1
EIF2AK3
STK39
b
PTM-SEA
Kinase activity
−2−10 1 2
Down Up
−log10(P−value)
1
2
3
4
SARS−CoV−2
HCoV−OC43
HCoV−229E
DNAPK
ASK1
MKK4
PRKD1
TBK1
Akt1
CK2A1
NDR1
PKCB
PKCE
Chk1
CDK7
MELK
RSK2
SGK1
Src
HIPK2
CDK1
AurB
CDK2
CDK4
Cot
GSK3A
GSK3B
P38A
PKACA
PLK1
p70S6K
p90RSK
WNK1
c
SARS-CoV-2 - + kDa
p-TBK1-S172 72
TBK1 72
GAPDH 43
HCoV-OC43 - + kDa
p-TBK1-S172 72
TBK1 72
GAPDH 43
HCoV-229E - + kDa
p-TBK1-S172 72
TBK1 72
GAPDH 43
d
SARS-CoV-2
P<0.0001
P<0.0001
P=0.0004
% infection
80 100 120 140 160
HCoV-OC43
P<0.0001
P<0.0001
P<0.0001
80 100 120 140 160
HCoV-229E
P<0.0001
P<0.0001
P<0.0001
0 50 100 150 200 250
DMSO
BX795
TBK1/IKKε-IN-2
BAY-985
e
P=0.1403
P=0.2792
P=0.5142
% Cell viability
0 60 120
DMSO
BX795
TBK1/IKKε-IN-2
BAY-985

Supplementary Fig. 2. TBK1 is activated during coronavirus infections

a, **b**, Kinase activity inference across SARS-CoV-2, HCoV-OC43, and HCoV-229E infections using orthogonal approaches: (a) Motif-centric analysis performed with the PhosR package[80], and (b) PTM-SEA analysis performed with ssGSEA2[81]. The color of the circles represents the normalized kinase activity (Red: upregulated; Blue: downregulated), and the size corresponds to the significance level (-log10 P-value). Dashed boxes highlight kinases with conserved regulatory patterns across the three coronaviruses, including the downregulation of MELK, GRK6, and CDK7, and the upregulation of DNAPK. P-values were calculated using the empirical Bayes method in limma. Source data are provided as a SourceData file.

c, Virus infections activated TBK1. Huh7 cells were infected with HCoV-229E, HCoV-OC43 and SARS-CoV-2 for 24 hours respectively. Activated p-TBK1 levels were measured by SDS-PAGE. Images were representative of three independent experiments. Source data are provided as a SourceData file.

d, e, TBK1 inhibitors promoted the infection of HCoV-229E, HCoV-OC43 and SARS-CoV-2. Huh7 cells were infected with SARS-CoV-2 (MOI=1.0), HCoV-229E (MOI=0.1) and HCoV-OC43 (MOI=1), respectively, in the presence of BX795 (1 μM), TBK1/IKKε-IN-2 (0.5 μM) and BAY-985 (1 μM) for 24 hours. (d) The infection rate was measured using IFA with N protein staining, and (e) the cytotoxicity of the compounds was determined by the CCK8 assay. Source data are provided as a SourceData file.

Data presented in (d) and (e) are mean ± SEM of *n* = 3 independent biological replicates. Statistical significance was determined using one-way ANOVA followed by two-sided Dunnett's multiple comparisons test. Exact P values are indicated in the figure.

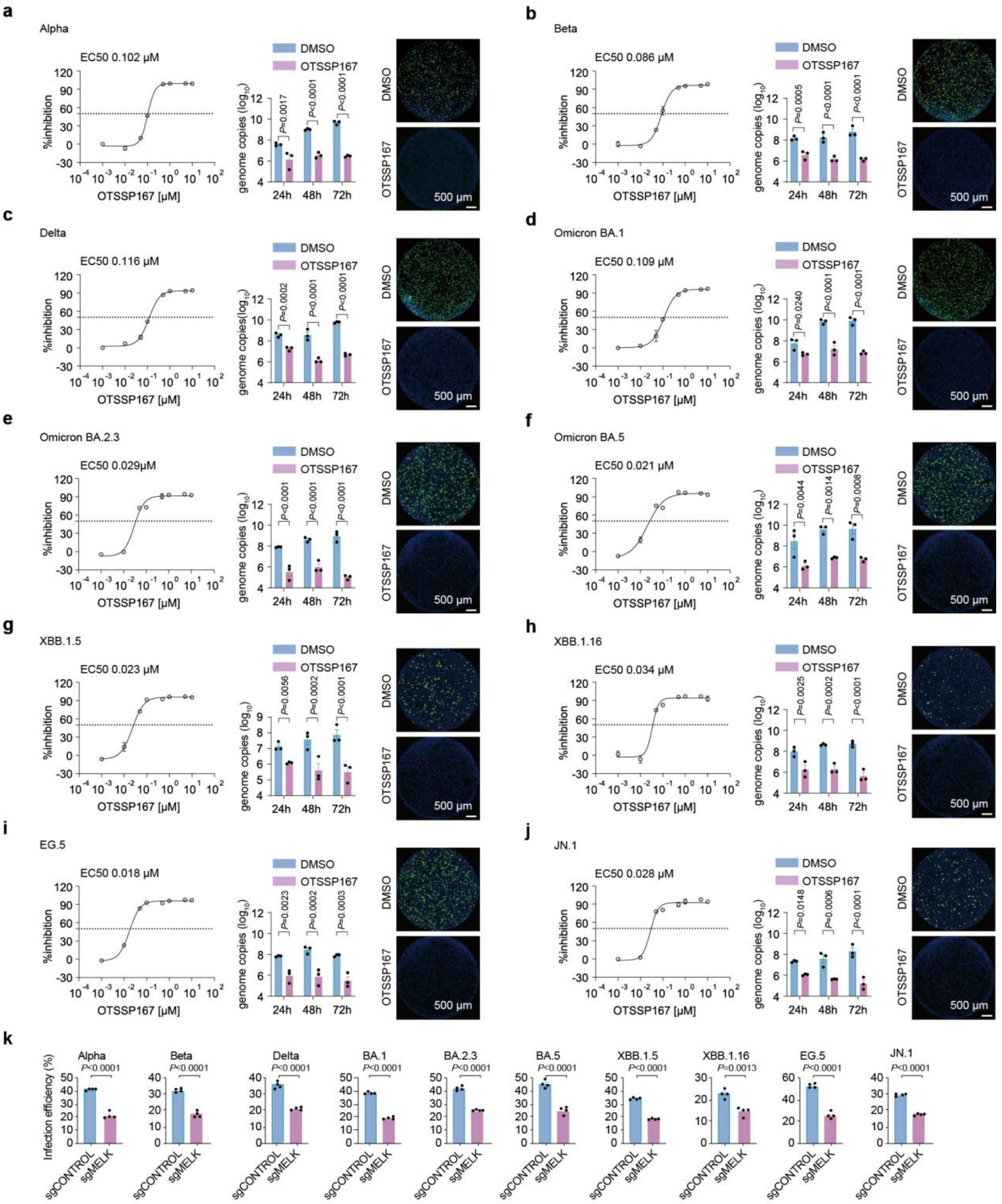

a
Alpha
EC50 0.102 µM
%inhibition
OTSSP167 [µM]
DMSO
OTSSP167
genome copies (log10)
P=0.0017
P<0.0001
P<0.0001
24h
48h
72h
500 µm
b
Beta
EC50 0.086 µM
P=0.0005
P<0.0001
P<0.0001
c
Delta
EC50 0.116 µM
P=0.0002
P<0.0001
P<0.0001
d
Omicron BA.1
EC50 0.109 µM
P=0.0240
P<0.0001
P<0.0001
e
Omicron BA.2.3
EC50 0.029µM
P<0.0001
P<0.0001
P<0.0001
f
Omicron BA.5
EC50 0.021 µM
P=0.0044
P=0.0014
P=0.0008
g
XBB.1.5
EC50 0.023 µM
P=0.0056
P=0.0002
P<0.0001
h
XBB.1.16
EC50 0.034 µM
P=0.0025
P=0.0002
P<0.0001
i
EG.5
EC50 0.018 µM
P=0.0023
P=0.0002
P=0.0003
j
JN.1
EC50 0.028 µM
P=0.0148
P=0.0006
P<0.0001
k
Infection efficiency (%)
Alpha P<0.0001
Beta P<0.0001
Delta P<0.0001
BA.1 P<0.0001
BA.2.3 P<0.0001
BA.5 P<0.0001
XBB.1.5 P<0.0001
XBB.1.16 P=0.0013
EG.5 P<0.0001
JN.1 P<0.0001
sgCONTROL
sgMELK

Supplementary Fig. 3. MELK inhibition blocks the infection of SARS-CoV-2 VOCs

a-j, OTSSP167 inhibited the infections of SARS-CoV-2 VOCs. As in Fig.2d. Source data are provided as a SourceData file.

k, Decreasing MELK expression inhibited the infections of SARS-CoV-2 VOCs. As in Fig.2h. Source data are provided as a SourceData file.

Data presented in (a) to (j) are mean ± SEM of n = 3 independent biological replicates. Statistical significance was determined using two-way ANOVA followed by two-sided Sidaks multiple comparisons test. Exact P values are indicated in the figure.

Data presented in (k) are mean ± SEM of n = 4 independent biological replicates. Statistical significance was determined using two-tailed Student's t-test. Exact P values are indicated in the figure.

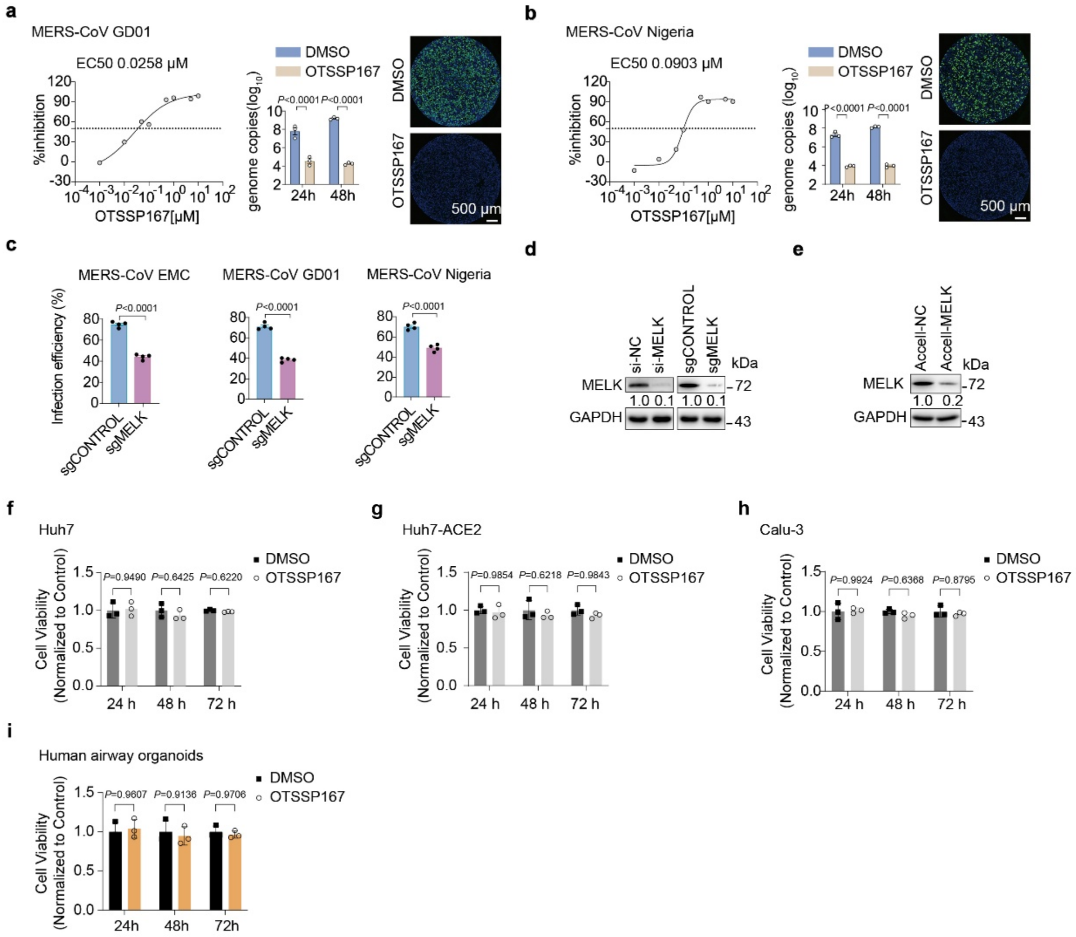
a
MERS-CoV GD01
EC50 0.0258 μM
%inhibition
OTSSP167[μM]
DMSO
OTSSP167
genome copies(log10)
P<0.0001
24h
48h
500 μm
b
MERS-CoV Nigeria
EC50 0.0903 μM
c
MERS-CoV EMC
MERS-CoV GD01
MERS-CoV Nigeria
Infection efficiency (%)
sgCONTROL
sgMELK
d
si-NC
si-MELK
kDa
MELK
GAPDH
1.0 0.1 1.0 0.1
-72
-43
e
Accell-NC
Accell-MELK
1.0 0.2
f
Huh7
Cell Viability (Normalized to Control)
P=0.9490
P=0.6425
P=0.6220
24 h
48 h
72 h
g
Huh7-ACE2
P=0.9854
P=0.6218
P=0.9843
h
Calu-3
P=0.9924
P=0.6368
P=0.8795
i
Human airway organoids
P=0.9607
P=0.9136
P=0.9706
24h
48h
72h

Supplementary Fig. 4. Broad restriction of various MERS-CoV strains by MELK inhibition and cytotoxicity analysis of OTSSP167

a, b, OTSSP167 inhibited the infections of MERS-CoV GD01 and MERS-CoV Nigeria. As in Fig.2d. Images were representative of three independent experiments. Source data are provided as a SourceData file.

c, Decreasing MELK expression inhibited the infections of various MERS-CoV strains. As in Fig.2h. Source data are provided as a SourceData file.

d, e, Immunoblotting analysis of the MELK expression after (**d**) siRNA transfection and sgRNA lentivirus transduction in Huh7 cells, and (**e**) Accell-siRNA transfection in human proximal airway organoids. The gray values were quantified using ImageJ and normalized to GAPDH. Images were representative of three independent experiments. Source data are provided as a SourceData file.

f-i, Cell viability assays were performed to assess the potential cytotoxicity of OTSSP167(0.2 μM) in (f) Huh7, (g) Huh7-ACE2, (h) Calu-3 cells, and (i) human airway organoids. Cells were treated with OTSSP167 or DMSO for the indicated time points (24, 48, and 72 h). Viability was quantified using the CCK-8 assay for cell lines (f‒h) and the CellTiter-Glo ATP assay for organoids (i). Results are normalized to the DMSO-treated control at each time point. Source data are provided as a SourceData file.

Data presented in (a), (b) and (f) to (i) are mean ± SEM of n = 3 independent biological replicates. Statistical significance was determined using two-way ANOVA followed by two-sided Sidaks multiple comparisons test. Exact P values are indicated in the figure.

Data presented in (c) are mean ± SEM of n = 4 independent biological replicates. Statistical significance was determined using two-tailed Student's t-test. Exact P values are indicated in the figure.

a

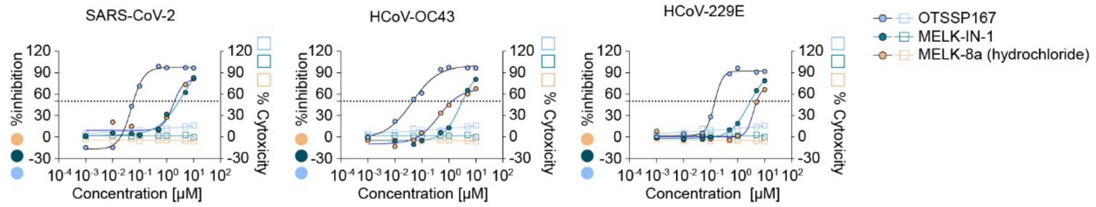

b

	OTSSP167 IC50 0.41nM	MELK-8a IC50 2nM	MELK-IN-1 IC50 3nM
SARS-CoV-2 EC50 (μM)	0.0492	1.684	3.956
HCoV-OC43 EC50 (μM)	0.0441	1.089	2.730
HCoV-229E EC50 (μM)	0.1382	3.920	2.631

c **d**

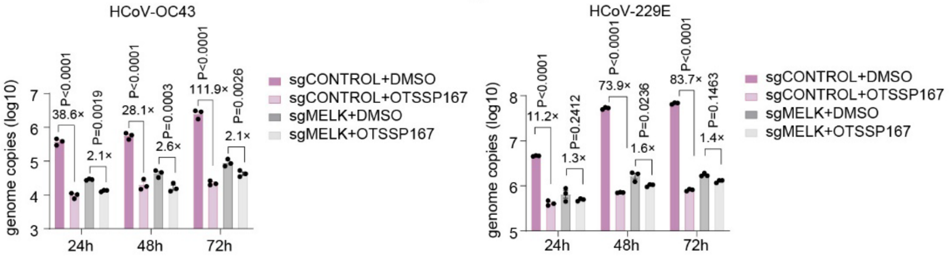

e

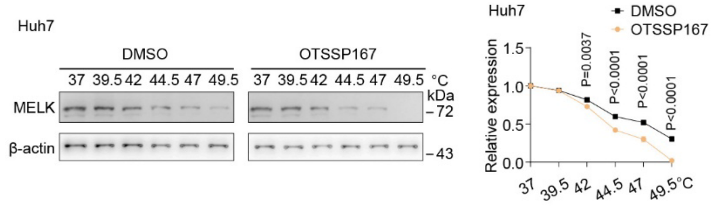

f

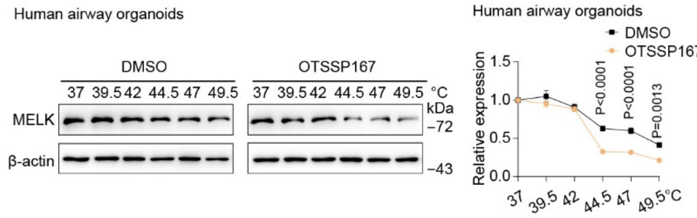

g

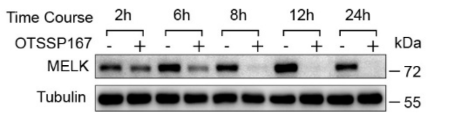

Supplementary Fig. 5. Antiviral activity of other MELK inhibitors and confirmation of OTSSP167 target engagement

a, b, Percent inhibition of (a) MELK-IN-1 and (b) MELK-8a against SARS-CoV-2, HCoV-OC43 and HCoV-229E infections and cytotoxicity in Huh7 cells. As in Fig.1g. Source data are provided as a SourceData file.

c, d, The effect of OTSSP167 on viral infections in sgCONTROL- or sgMELK-transduced cells. Virus gene copy number in the supernatant was quantified by qPCR of the N gene at 24, 48 and 72 hpi. As in Fig.2a. Source data are provided as a SourceData file.

e, f, Cellular thermal shift assay (CETSA) determined the target engagement of MELK by OTSSP167 in (e) Huh7 cells and (f) human airway organoids. Cells were incubated with DMSO or OTSSP167 (0.2 μM) for 0.5 h, harvested, and subsequently subjected to heat treatment at the indicated temperatures for 3 min. Left: Soluble MELK protein levels were analyzed by Western blot. β-actin served as the loading control. Right: Melt curves displaying the quantification of MELK relative protein abundance plotted against temperature. Images were representative of three independent experiments. Source data are provided as a SourceData file.

g, Time-course Western Blot analysis of MELK in Huh7 cells treated with OTSSP167 or DMSO. Tubulin served as the loading control. Images were representative of three independent experiments. Source data are provided as a SourceData file.

Data presented in (a) are mean ± SEM of n = 3 independent biological replicates.

Data presented in (c) to (f) are mean ± SEM of n = 3 independent biological replicates. Statistical significance was determined using two-way ANOVA followed by two-sided Sidaks multiple comparisons test. Exact P values are indicated in the figure.

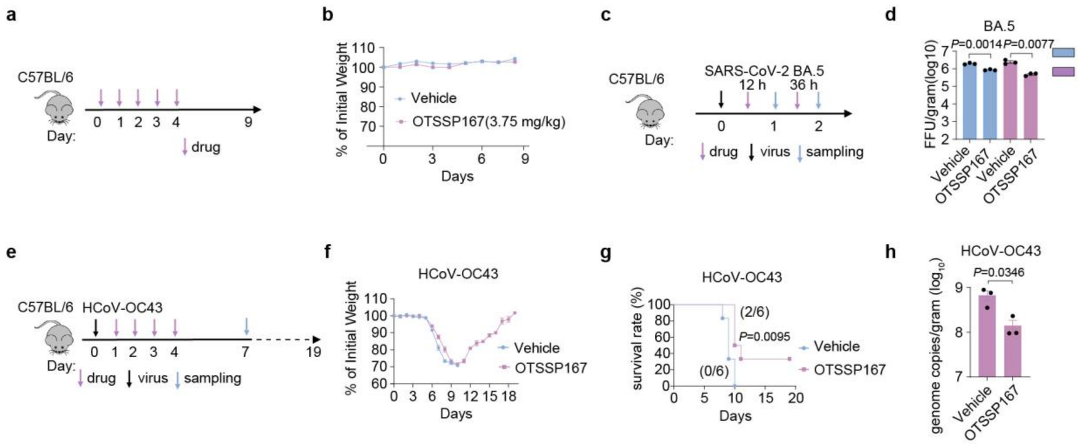
a
C57BL/6
Day:
0 1 2 3 4 9
drug
b
% of Initial Weight
Days
Vehicle
OTSSP167(3.75 mg/kg)
c
C57BL/6
SARS-CoV-2 BA.5
12 h
36 h
Day:
0 1 2
drug
virus
sampling
d
BA.5
FFU/gram(log10)
P=0.0014
P=0.0077
Day1
Day2
Vehicle
OTSSP167
Vehicle
OTSSP167
e
C57BL/6
HCoV-OC43
Day:
0 1 2 3 4 7 19
drug
virus
sampling
f
HCoV-OC43
% of Initial Weight
Days
Vehicle
OTSSP167
g
HCoV-OC43
survival rate (%)
Days
(2/6)
P=0.0095
(0/6)
Vehicle
OTSSP167
h
HCoV-OC43
genome copies/gram (log10)
P=0.0346
Vehicle
OTSSP167

Supplementary Fig. 6. Evaluation of the therapeutic potential of OTSSP167

a,b, Six-week-old C57BL/6 mice were used for an intranasal tolerability study of OTSSP167. OTSSP167 (3.75 mg/kg) was administered daily for 5 consecutive days. (a) Schematic of intranasal tolerability study in C57BL/6 mice. (b) Body weight of the mice was monitored for 8 days. Source data are provided as a SourceData file.

c,d, Six-week-old C57BL/6 mice were intranasally infected with 1×10^5 FFU SARS-CoV-2 BA.5 and treated with intranasal administration of DMSO or OTSSP167 (3.75 mg/kg/day) at 12 hpi and 36 hpi. Lungs of infected mice were collected on 1 dpi and 2 dpi. (c) Schematic of therapeutic administration in SARS-CoV-2 BA.5-infected C57BL/6 mice. (d)The viral titers in lungs were determined by FFA. Source data are provided as a SourceData file.

e-h, Six-week-old C57BL/6 mice were intranasally infected with 2×10^4 FFU HCoV-OC43 and treated intranasally with DMSO or OTSSP167 (3.75 mg/kg/day) from 1 dpi for 4 days. Brains of infected mice were collected at 7 dpi. (e) Schematic of therapeutic administration in HCoV-OC43-infected C57BL/6 mice. (f) Body weight and (g) survival of the infected mice were monitored for 19 days. (h) Infected mice were sacrificed at 7 dpi, and viral gene copies in brain were quantified by qPCR. Data presented in (d) and (h) are mean $\pm$ SEM of n = 3 independent biological replicates. Statistical significance was determined using two-tailed Student s t-test. Exact P values are indicated in the figure. Source data are provided as a SourceData file.

Data presented in (g) are survival curves of n = 6 mice per group. Statistical significance was determined using Log-rank (Mantel-Cox) test. Exact P values are indicated in the figure.

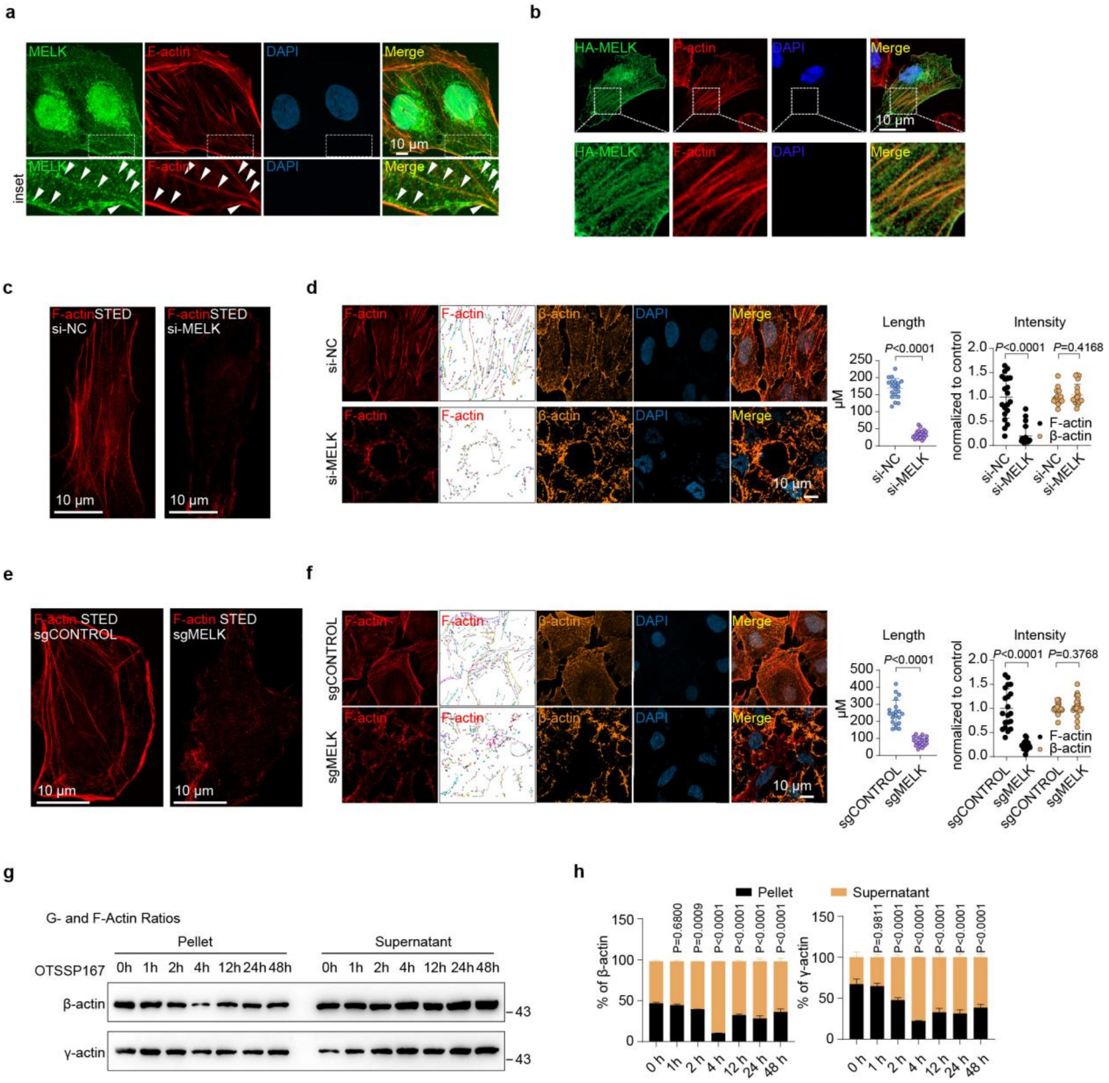
a
MELK
F-actin
DAPI
Merge
10 μm
inset
b
HA-MELK
F-actin
DAPI
Merge
10 μm
c
F-actin STED
si-NC
si-MELK
10 μm
d
si-NC
si-MELK
β-actin
Length
P<0.0001
μM
Intensity
P=0.4168
normalized to control
e
sgCONTROL
sgMELK
f
P=0.3768
g
G- and F-Actin Ratios
Pellet
Supernatant
OTSSP167
0h 1h 2h 4h 12h 24h 48h
γ-actin
43
h
% of β-actin
% of γ-actin
P=0.6800
P=0.0009
P=0.9811
0 h 1 h 2 h 4 h 12 h 24 h 48 h

Supplementary Fig. 7. Inhibition of MELK induces the depolymerization of F-actin

a, b, MELK colocalized with F-actin. Representative confocal microscopy images showed colocalization of (a) MELK or (b) overexpressed HA-MELK (green) with F-actin (AF647-phalloidin, red) in Huh7 cells. Nuclei (DAPI), blue. Images were representative of three independent experiments.

c-f, MELK inhibition in expression induced depolymerization of F-actin. Representative STED microscopy images showed F-actin (STAR RED phalloidin) in (c) si-MELK-transfected and (e) sgMELK-transduced cells. Representative immunostaining of G-actin and F-actin in (d) si-MELK-transfected and (f) sgMELK-transduced cells. Cells were fixed and immunostained with AF647-phalloidin and anti-β-actin antibodies. The F-actin length and the intensities of F-actin and G-actin in individual cells were quantified using Arivis Vision4D software. Images were representative of three independent experiments. Source data are provided as a SourceData file.

g, Biochemical fractionation analysis of G-actin and F-actin ratios. Huh7 cells were treated with OTSSP167 for 0, 1, 2, 4, 12, 24, and 48 hours. F-actin (Pellet) and G-actin (Supernatant) fractions were separated by high-speed centrifugation (66000g, 1h), and the distribution of β-actin and γ-actin was detected by Western blot. Images were representative of three independent experiments. Source data are provided as a SourceData file.

h, Quantitative analysis of the relative levels of β-actin and γ-actin with imageJ as shown in (h). Source data are provided as a SourceData file.

Data presented in (d) and (f) are mean ± SD of n =20 from three independent biological replicates. Statistical significance was determined using two-tailed Students t -test. Exact P values are indicated in the figure.

Data presented in (h) are mean ± SEM of n = 3 independent biological replicates. Statistical significance was determined using two-way ANOVA followed by two-sided Sidaks multiple comparisons test. Exact P values are indicated in the figure.

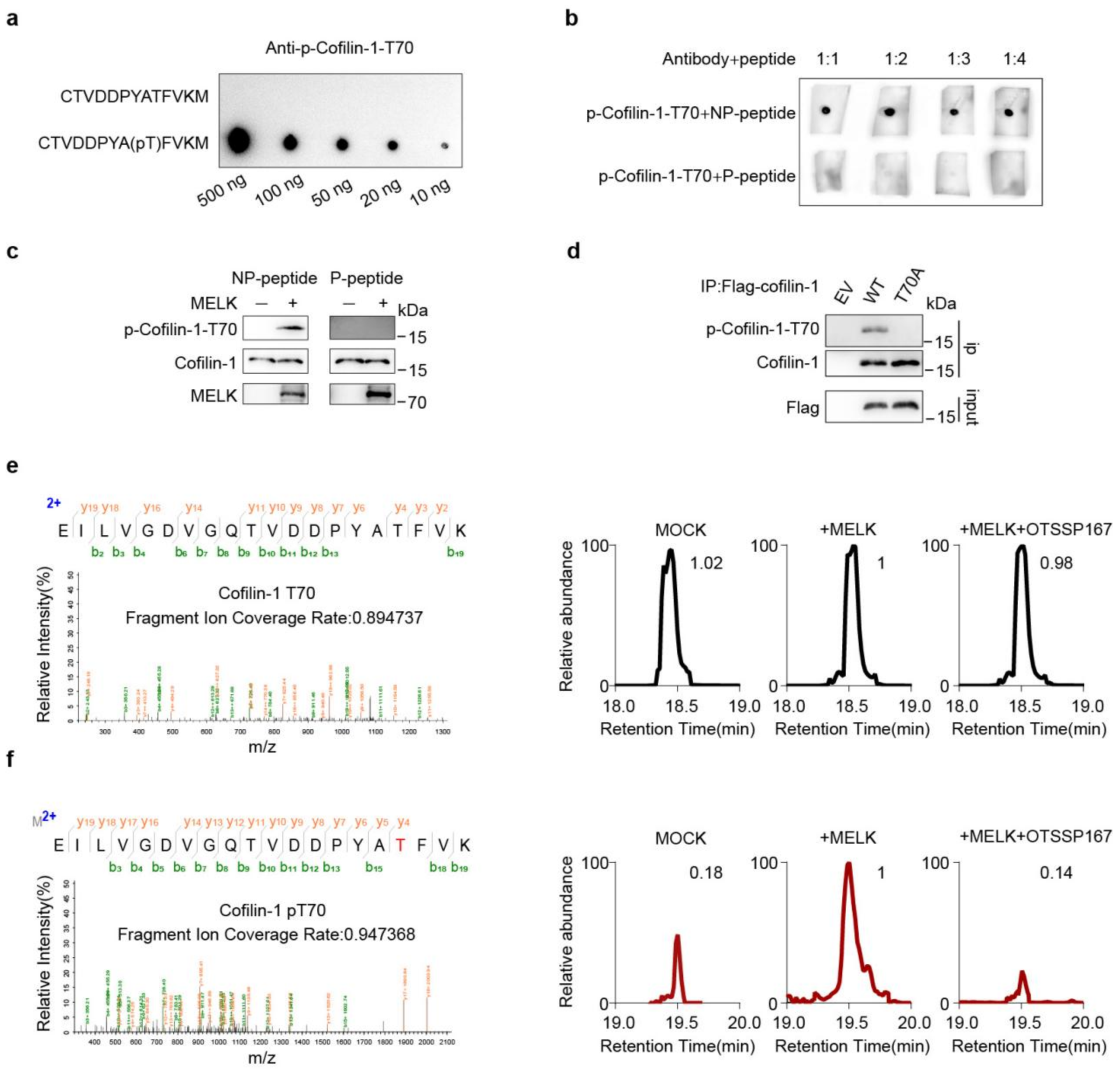
a
Anti-p-Cofilin-1-T70
CTVDDPYATFVKM
CTVDDPYA(pT)FVKM
500 ng
100 ng
50 ng
20 ng
10 ng
b
Antibody+peptide
1:1
1:2
1:3
1:4
p-Cofilin-1-T70+NP-peptide
p-Cofilin-1-T70+P-peptide
c
NP-peptide
P-peptide
MELK
kDa
p-Cofilin-1-T70
Cofilin-1
MELK
15
15
70
d
IP:Flag-cofilin-1
EV
WT
T70A
kDa
p-Cofilin-1-T70
Cofilin-1
Flag
ip
input
e
EILVGDVGQTVDDPYATFVK
Cofilin-1 T70
Fragment Ion Coverage Rate:0.894737
Relative Intensity(%)
m/z
MOCK
+MELK
+MELK+OTSSP167
1.02
1
0.98
Relative abundance
Retention Time(min)
f
EILVGDVGQTVDDPYATFVK
Cofilin-1 pT70
Fragment Ion Coverage Rate:0.947368
Relative Intensity(%)
m/z
MOCK
+MELK
+MELK+OTSSP167
0.18
1
0.14
Relative abundance
Retention Time(min)

Supplementary Fig. 8. Validation of the p-Cofilin-1-T70 antibody and Cofilin-1 T70 phosphorylation

a, Evaluation of antibody specificity using dot blot assay. Indicated amounts of non-phosphorylated (NP) or phosphorylated (P) peptides corresponding to the Cofilin-1 T70 site were spotted onto the membrane and immunoblotted with the generated p-Cofilin-1-T70 antibody. Images were representative of three independent experiments. Source data are provided as a SourceData file.

b,c, Peptide competition assay to validate the specificity of p-Cofilin-1-T70 antibody. b, The antibody was pre-incubated with phosphorylated Cofilin-1 T70 peptide (P-peptide) or non-phosphorylated Cofilin-1 T70 peptide (NP-peptide) at mass ratios of 1:1, 1:2, 1:3, and 1:4(antibody:peptide), then used to probe phosphorylated peptide spots. Each blot representing a separate membrane strip for the indicated condition. c, In vitro kinase assay validation. Recombinant Cofilin-1 was incubated with or without recombinant MELK in the presence of ATP. The reaction mixtures were immunoblotted with the antibody pre-incubated with NP- or P-peptides (1:1 mass ratio). Images were representative of three independent experiments. Source data are provided as a SourceData file.

d, HEK293T cells were transfected with Flag-tagged wild-type (WT) or T70A mutant Cofilin-1 for 24 hours. Flag-Cofilin-1 protein was immunoprecipitated with anti-Flag beads and immunoblotted with the generated anti-p-Cofilin-1-T70 antibody. Images were representative of three independent experiments. Source data are provided as a SourceData file.

e, f, Identification of Cofilin-1 T70 phosphorylation by LC-MS/MS analysis. HEK293T cells were transfected with MELK or empty vector for 24 h, followed by treatment with OTSSP167 (0.2 μM) or DMSO for 4 hours. Endogenous Cofilin-1 was enriched by immunoprecipitation using an anti-Cofilin-1 antibody and subjected to LC-MS/MS identification. Left panels: Annotated MS/MS spectra of the (e) non-phosphorylated and (f) T70-phosphorylated peptide (EILVGDVGQTVDDPYATFVK) identified from the enriched samples. The phosphorylation site was localized to Threonine 70 with high confidence (probability

score > 0.98). Right panels: Extracted ion chromatograms (XIC) displaying the relative abundance of the indicated peptides. Note that the pT70 signal (f) is markedly enriched by MELK overexpression but abolished by OTSSP167 treatment. Source data are provided as a SourceData file.

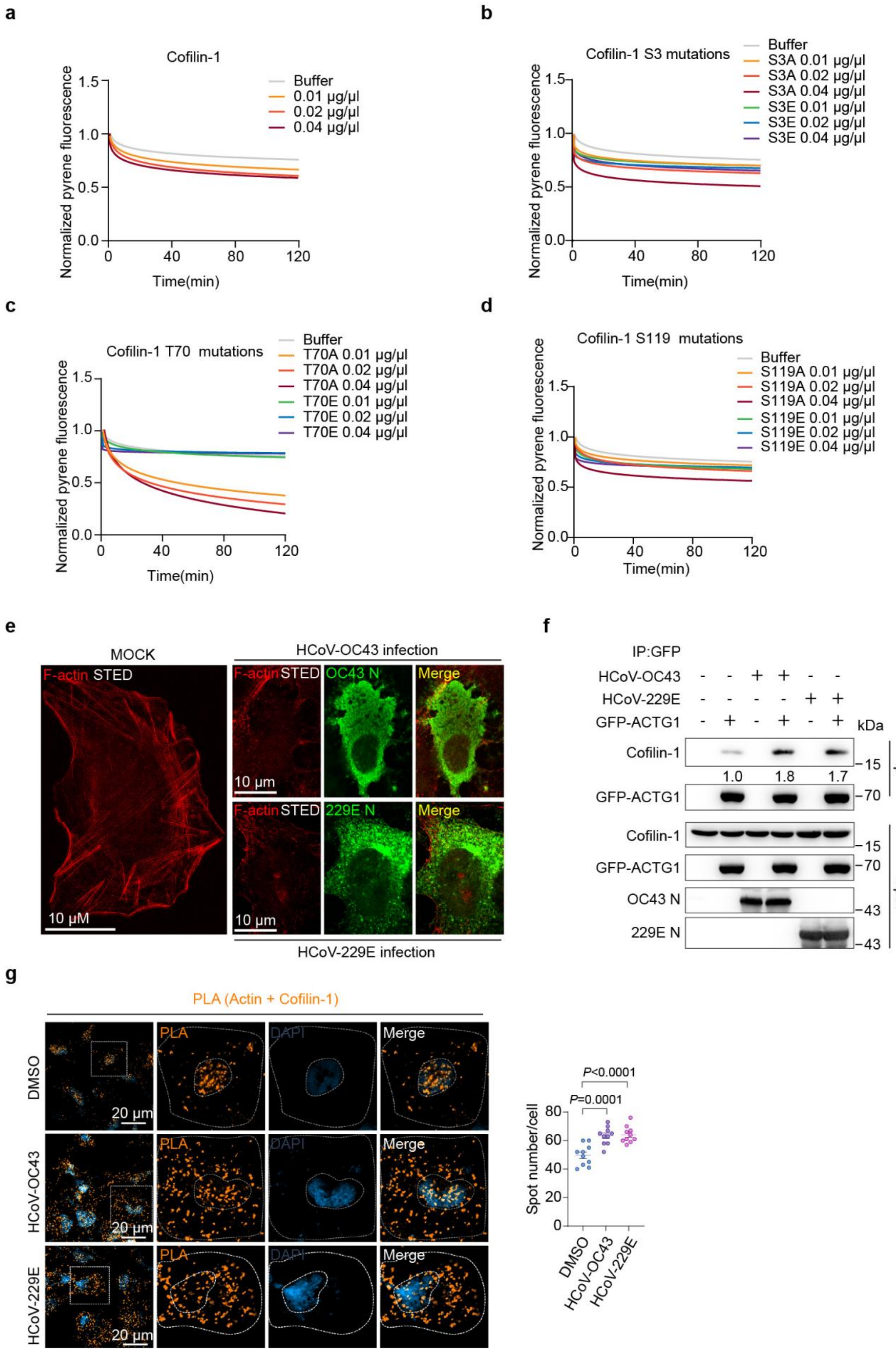

a
Cofilin-1
Buffer
0.01 μg/μl
0.02 μg/μl
0.04 μg/μl
Normalized pyrene fluorescence
Time(min)
b
Cofilin-1 S3 mutations
Buffer
S3A 0.01 μg/μl
S3A 0.02 μg/μl
S3A 0.04 μg/μl
S3E 0.01 μg/μl
S3E 0.02 μg/μl
S3E 0.04 μg/μl
c
Cofilin-1 T70 mutations
T70A 0.01 μg/μl
T70A 0.02 μg/μl
T70A 0.04 μg/μl
T70E 0.01 μg/μl
T70E 0.02 μg/μl
T70E 0.04 μg/μl
d
Cofilin-1 S119 mutations
S119A 0.01 μg/μl
S119A 0.02 μg/μl
S119A 0.04 μg/μl
S119E 0.01 μg/μl
S119E 0.02 μg/μl
S119E 0.04 μg/μl
e
MOCK
F-actin STED
10 μM
HCoV-OC43 infection
OC43 N
Merge
10 μm
229E N
HCoV-229E infection
f
IP:GFP
HCoV-OC43
HCoV-229E
GFP-ACTG1
kDa
Cofilin-1
1.0 1.8 1.7
ip
input
OC43 N
229E N
g
PLA (Actin + Cofilin-1)
DMSO
HCoV-OC43
HCoV-229E
PLA
DAPI
Merge
20 μm
Spot number/cell
P<0.0001
P=0.0001

Supplementary Fig. 9. Characterization of Cofilin-1 depolymerization activity and its association with actin upon viral infection

a-d, The severance activity of cofilin-1 WT and mutants was examined in an *in vitro* pyrene-actin depolymerization assay. All proteins were tested at three increasing doses. The depolymerization of F-actin was monitored by fluorescence intensity using a microplate reader.

e, Viral infections induced rearrangement of F-actin. Representative STED microscopy images showed F-actin (STAR RED phalloidin) in MOCK, HCoV-OC43 and HCoV-229E infected (24 h) cells. Infected cells were stained with anti-viral N antibody (green). Images were representative of three independent experiments.

f, Viral infections enhanced the interaction between cofilin-1 and actin. The interaction between cofilin-1 and actin was examined by coimmunoprecipitation. The gray values were quantified using ImageJ. Images were representative of three independent experiments. Source data are provided as a SourceData file.

g, Viral infections induced colocalization between cofilin-1 and actin in situ. Colocalization between cofilin-1 and actin was quantified by PLA using the indicated antibodies. Orange immunofluorescent dots appear when actin interacts with cofilin-1. The number of orange fluorescent dots in each cell treated with MOCK or viral infections was enumerated. Images were representative of three independent experiments. Source data are provided as a SourceData file.

Data presented in (g) are mean ± SD of $n = 10$ from three independent biological replicates. Statistical significance was determined using one-way ANOVA followed by two-sided Dunnett's multiple comparisons test. Exact P values are indicated in the figure.

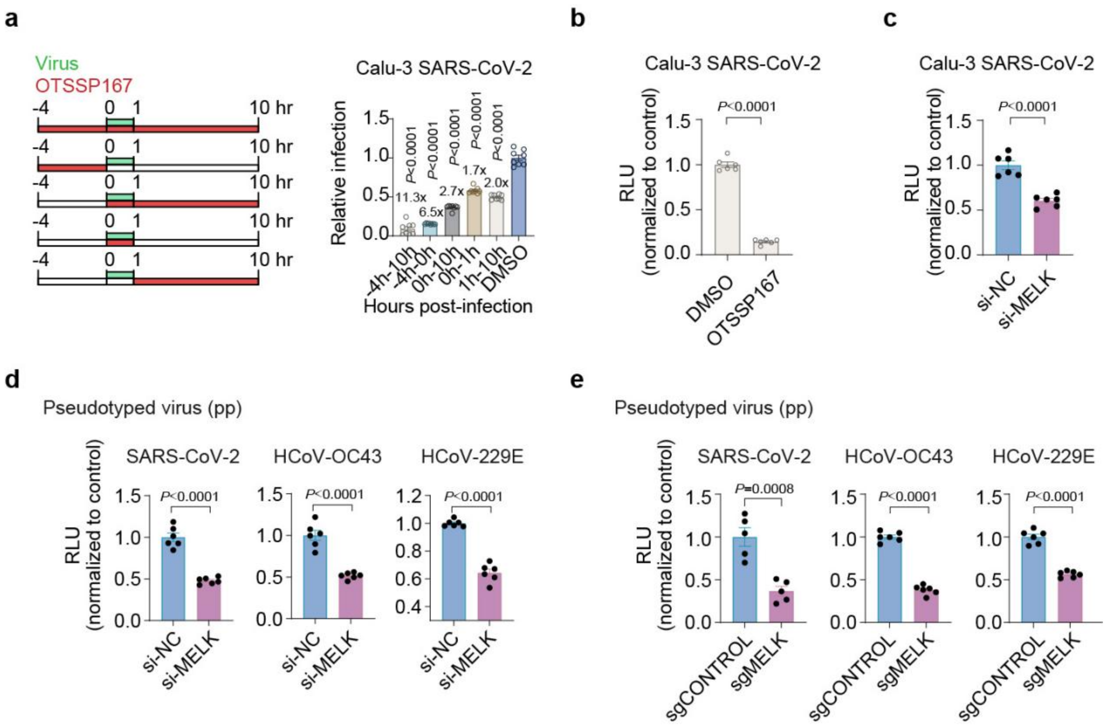
a
Virus
OTSSP167
-4 0 1 10 hr
Calu-3 SARS-CoV-2
Relative infection
Hours post-infection
-4h-10h
-4h-0h
0h-10h
0h-1h
1h-10h
DMSO
11.3x
6.5x
2.7x
1.7x
2.0x
P<0.0001
b
Calu-3 SARS-CoV-2
RLU (normalized to control)
P<0.0001
DMSO
OTSSP167
c
Calu-3 SARS-CoV-2
RLU (normalized to control)
P<0.0001
si-NC
si-MELK
d
Pseudotyped virus (pp)
SARS-CoV-2
HCoV-OC43
HCoV-229E
RLU (normalized to control)
P<0.0001
si-NC
si-MELK
e
Pseudotyped virus (pp)
SARS-CoV-2
HCoV-OC43
HCoV-229E
RLU (normalized to control)
P=0.0008
P<0.0001
sgCONTROL
sgMELK

Supplementary Fig. 10. Inhibition of MELK suppresses coronavirus infections in both Calu-3 and Huh7 cells

a, Time-of-addition assay. Calu-3 cells were incubated with OTSSP167(0.2 μM) at the time points indicated. Infection efficiency at 24 hours post infection was quantified by immunostaining N protein and normalized to the DMSO-treated cells. Source data are provided as a SourceData file.

b, Quantification of viral entry efficiency in Calu-3 cells with or without OTSSP167 (0.2 μM) treatment inoculated with pseudoviruses bearing spike proteins from SARS-CoV-2. Viral entry efficiency was measured by normalized RLU. Source data are provided as a SourceData file.

c, Quantification of viral entry efficiency in siMELK-transfected Calu-3 cells inoculated with pseudoviruses bearing spike proteins from SARS-CoV-2. Viral entry efficiency was measured by normalized RLU. Source data are provided as a SourceData file.

d, e, Quantification of viral entry efficiency in (d) siMELK-transfected and (e) sgMELK-transduced Huh7 cells inoculated with pseudoviruses bearing spike proteins from SARS-CoV-2, HCoV-OC43 and HCoV-229E. Source data are provided as a SourceData file.

Data presented in (a) are mean ± SEM of n=8 independent biological replicates. Statistical significance was determined using one-way ANOVA followed by two-sided Dunnett's multiple comparisons test. Exact P values are indicated in the figure.

Data presented in (b) to (d) are mean ± SEM of n = 6, in (e) are mean ± SEM of SARS-CoV-2 (n = 5) and in (j) are mean of HCoV-OC43 and HCoV-229E (n = 6) ± SEM from n = 3 independent biological replicates. Statistical significance was determined using two-tailed Student s t-test. Exact P values are indicated in the figure.

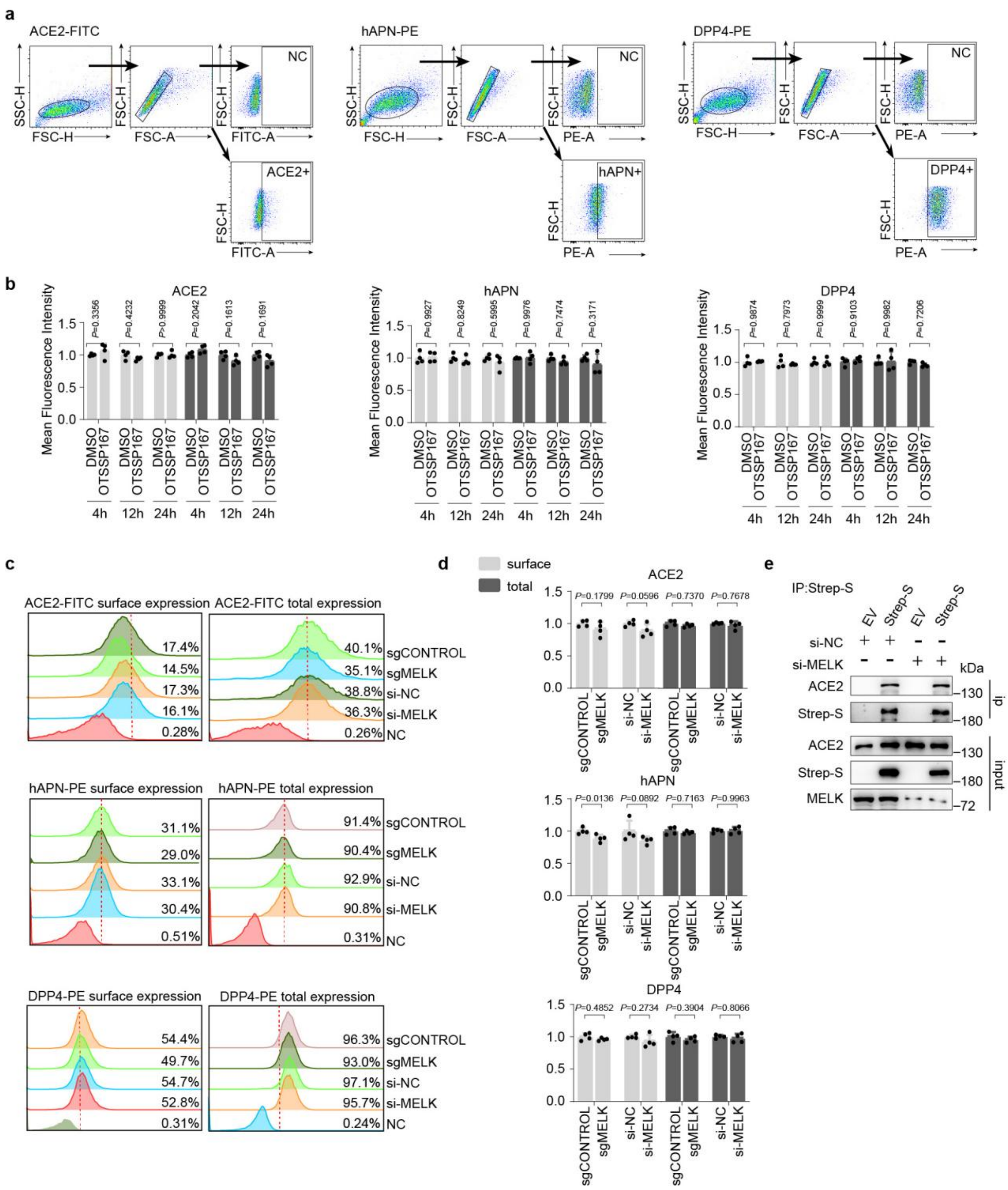
a
ACE2-FITC
hAPN-PE
DPP4-PE
SSC-H
FSC-H
FSC-A
FITC-A
PE-A
NC
ACE2+
hAPN+
DPP4+
b
ACE2
hAPN
DPP4
Mean Fluorescence Intensity
DMSO
OTSSP167
4h
12h
24h
c
ACE2-FITC surface expression
ACE2-FITC total expression
hAPN-PE surface expression
hAPN-PE total expression
DPP4-PE surface expression
DPP4-PE total expression
sgCONTROL
sgMELK
si-NC
si-MELK
NC
d
surface
total
ACE2
hAPN
DPP4
e
IP:Strep-S
EV
Strep-S
si-NC
si-MELK
kDa
ip
input
MELK

Supplementary Fig. 11. MELK inhibition does not affect viral receptor Expression or Spike-ACE2 interaction

a, Representative flow cytometry gating strategies for all flow cytometry assays in Figure 7g and Supplementary Fig 11b-d. Single-cell populations were gated sequentially by forward scatter-height vs side scatter-height (FSC-H vs SSC-H) and forward scatter-height vs forward scatter-area (FSC-H vs FSC-A), followed by discrimination of negative (NC) and positive (ACE2+, hAPN+, DPP4+) populations for ACE2, hAPN, and DPP4. Images were representative of three independent experiments.

b, Quantification of receptor expression. Mean fluorescence intensity (MFI) of surface and total ACE2, hAPN, and DPP4 at different time points (4, 12, 24 h) with OTSSP167 treated Huh7 Cells. Source data are provided as a SourceData file.

c, d, Decreased expression of MELK had no effect on the expression of viral receptors. The surface and total expressions of receptor ACE2, hAPN and DPP4 were detected with flow cytometry in sg/siMELK transduced Huh7 Cells. Source data are provided as a SourceData file.

e, Decreased expression of MELK had no effect on the interaction between SARS-CoV-2 spike and ACE2. HEK293T cells were co-transfected with indicated siRNA and plasmid for 48 h. The interaction between SARS-CoV-2 spike and ACE2 was examined by coimmunoprecipitation. Images were representative of three independent experiments. Source data are provided as a SourceData file.

Data presented in (b) and (d) are mean ± SEM of n = 4 independent biological replicates. Statistical significance was determined using two-way ANOVA followed by two-sided Sidaks multiple comparisons test. Exact P values are indicated in the figure.

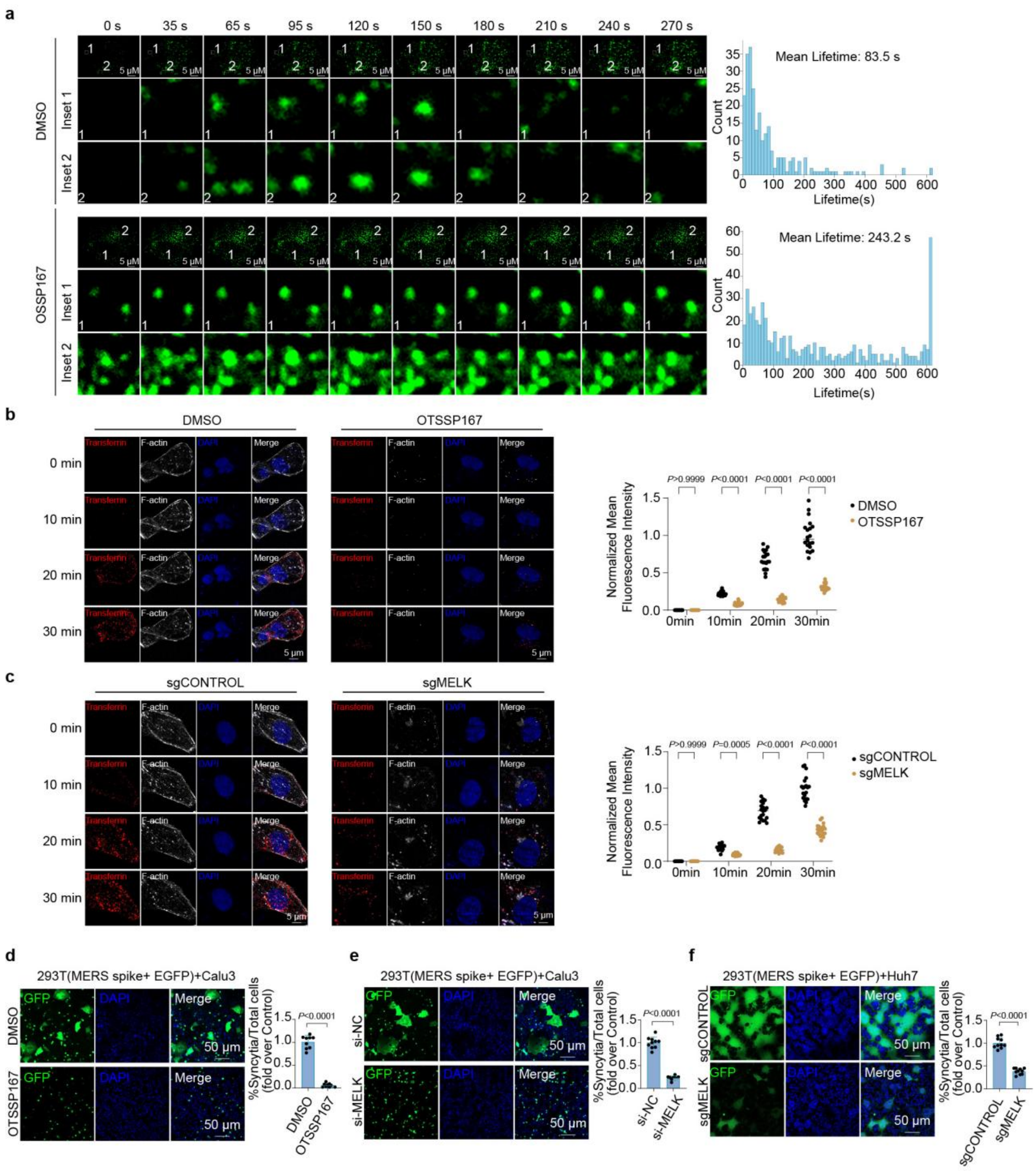

a
0 s
35 s
65 s
95 s
120 s
150 s
180 s
210 s
240 s
270 s
DMSO
Inset 1
Inset 2
5 μM
Mean Lifetime: 83.5 s
Count
Lifetime(s)
OSSP167
Mean Lifetime: 243.2 s
b
DMSO
OTSSP167
0 min
10 min
20 min
30 min
Transferrin
F-actin
DAPI
Merge
5 μm
Normalized Mean Fluorescence Intensity
P>0.9999
P<0.0001
DMSO
OTSSP167
0min
10min
20min
30min
c
sgCONTROL
sgMELK
P=0.0005
d
293T(MERS spike+ EGFP)+Calu3
GFP
50 μm
%Syncytia/Total cells (fold over Control)
e
si-NC
si-MELK
f
293T(MERS spike+ EGFP)+Huh7

Supplementary Fig. 12. MELK inhibition impairs clathrin-mediated endocytosis and spike-mediated membrane fusion

a, Analysis of clathrin-coated pit (CCP) dynamics using live-cell TIRF microscopy. Huh7 cells expressing GFP-Clathrin were treated with DMSO or OTSSP167(0.2 μM, 4 hours). Left: Time-lapse images of representative CCPs. Right: Histograms displaying the distribution of CCP lifetimes. The mean lifetime is indicated (83.5 s for DMSO vs 243.2 s for OTSSP167). Images were representative of three independent experiments. Source data are provided as a SourceData file.

b, c, Live-cell imaging analysis of transferrin uptake dynamics. (b) Huh7 cells treated with OTSSP167(vs DMSO) or (c) transduced with sgMELK (vs sgCONTROL) were incubated with Transferrin-AF555. The dynamic accumulation of internalized transferrin was monitored by live-cell confocal microscopy over a 30-min time course. Transferrin (red), F-actin (Sir-actin, gray), Nucleus (Hoechst, blue). The fluorescence intensity of intracellular transferrin was quantified at indicated time points to evaluate the differences in uptake efficiency (right panels). Images were representative of three independent experiments. Source data are provided as a SourceData file.

d-f, Spike-mediated membrane fusion assay. HEK293T cells were co-transfected with MERS-CoV S and pEGFP plasmids for 24 hours. These effector cells were then co-cultured with Calu-3 target cells that had been treated with (d) OTSSP167 (0.2 μM, 4 hours), (e) siMELK, or (f) subjected to MELK-knockout via sgMELK. After 12 hours, syncytia formation was observed via confocal microscopy and quantified as the percentage of fused cells relative to the control. Images were representative of three independent experiments. Source data are provided as a SourceData file.

Data presented in (b) and (c) are mean ± SEM of n = 20 independent biological replicates. Statistical significance was determined using two-way ANOVA followed by two-sided Sidaks multiple comparisons test. Exact P values are indicated in the figure.

Data presented in (d) are mean ± SEM of n = 9, in (e) and (f) are mean ± SEM of n = 10 independent biological replicates. Statistical significance was determined using

two-tailed Student’s t-test. Exact P values are indicated in the figure.

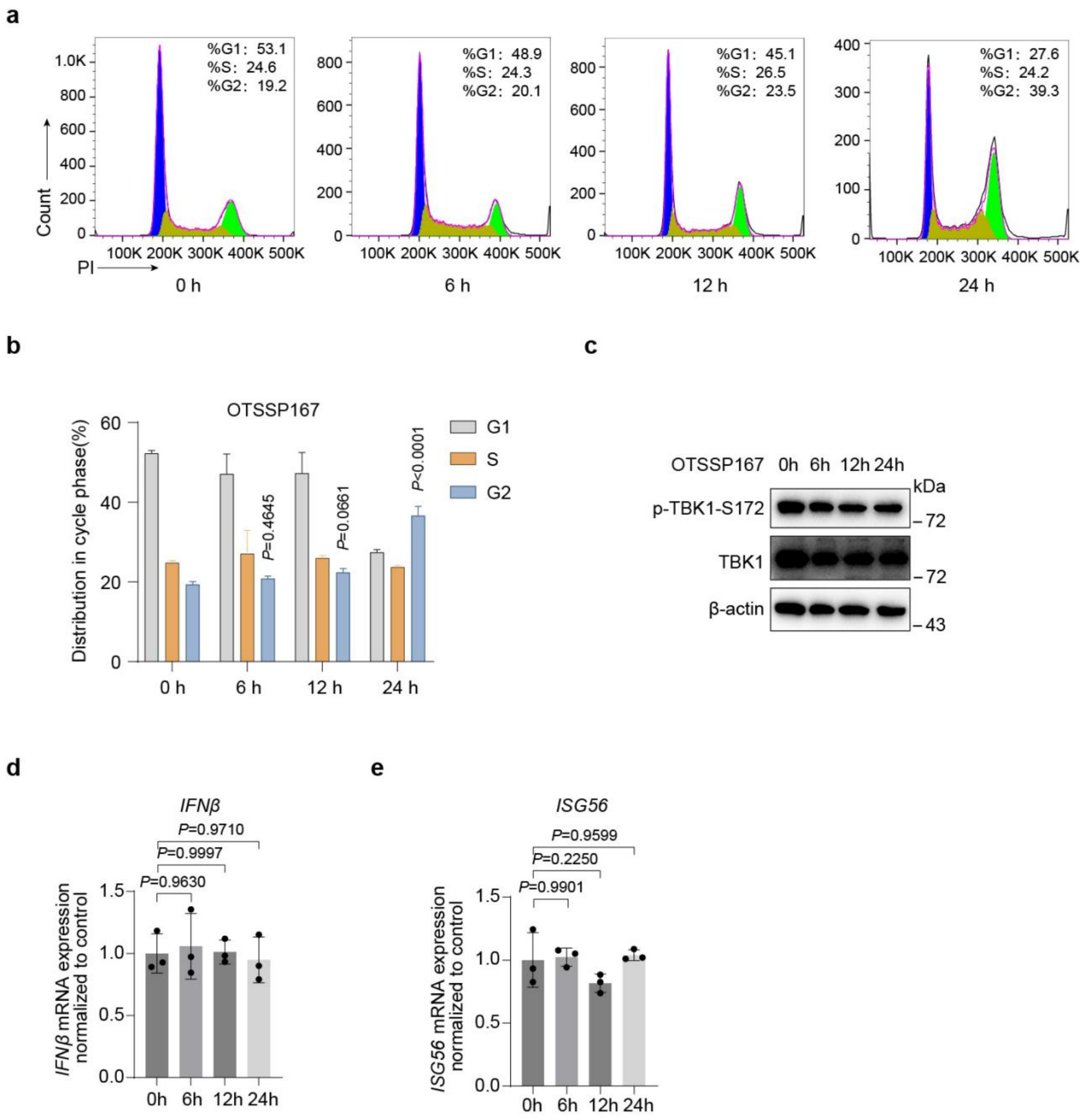
a
%G1: 53.1
%S: 24.6
%G2: 19.2
Count
PI
0 h
%G1: 48.9
%S: 24.3
%G2: 20.1
6 h
%G1: 45.1
%S: 26.5
%G2: 23.5
12 h
%G1: 27.6
%S: 24.2
%G2: 39.3
24 h
b
OTSSP167
Distribution in cycle phase(%)
G1
S
G2
P=0.4645
P=0.0661
P<0.0001
0 h
6 h
12 h
24 h
c
OTSSP167 0h 6h 12h 24h
kDa
p-TBK1-S172
TBK1
β-actin
72
72
43
d
IFNβ
IFNβ mRNA expression normalized to control
P=0.9710
P=0.9997
P=0.9630
0h 6h 12h 24h
e
ISG56
ISG56 mRNA expression normalized to control
P=0.9599
P=0.2250
P=0.9901
0h 6h 12h 24h

Supplementary Fig. 13. The antiviral activity of OTSSP167 is independent of cell cycle perturbation and innate immune responses

a, Representative flow cytometry plots show cell cycle distribution (proportions of G1, S, and G2/M phases) at different time points (0, 6, 12, 24 h) following OTSSP167 (0.2 μM) treatment for indicated time, as analyzed by PI staining. Images were representative of three independent experiments. Source data are provided as a SourceData file.

b, Quantification of cell cycle distribution in (a). Source data are provided as a SourceData file.

c, Western blot of phosphorylated TBK1 (p-TBK1-S172) and total TBK1 protein levels at different time points following OTSSP167 treatment in Huh7 cells. β-actin served as the loading control. Images were representative of three independent experiments. Source data are provided as a SourceData file.

d, e, (d) IFNβ and (e) ISG56 mRNA expression levels at different time points following OTSSP167 treatment in Huh7 cells, measured by qPCR and normalized to the 0 h control. Reference gene: GAPDH. Images were representative of three independent experiments. Source data are provided as a SourceData file.

Data presented in (b), (d) and (e) are mean ± SEM of n = 3 independent biological replicates. Statistical significance was determined using one-way ANOVA followed by two-sided Dunnett's multiple comparisons test. Exact P values are indicated in the figure.

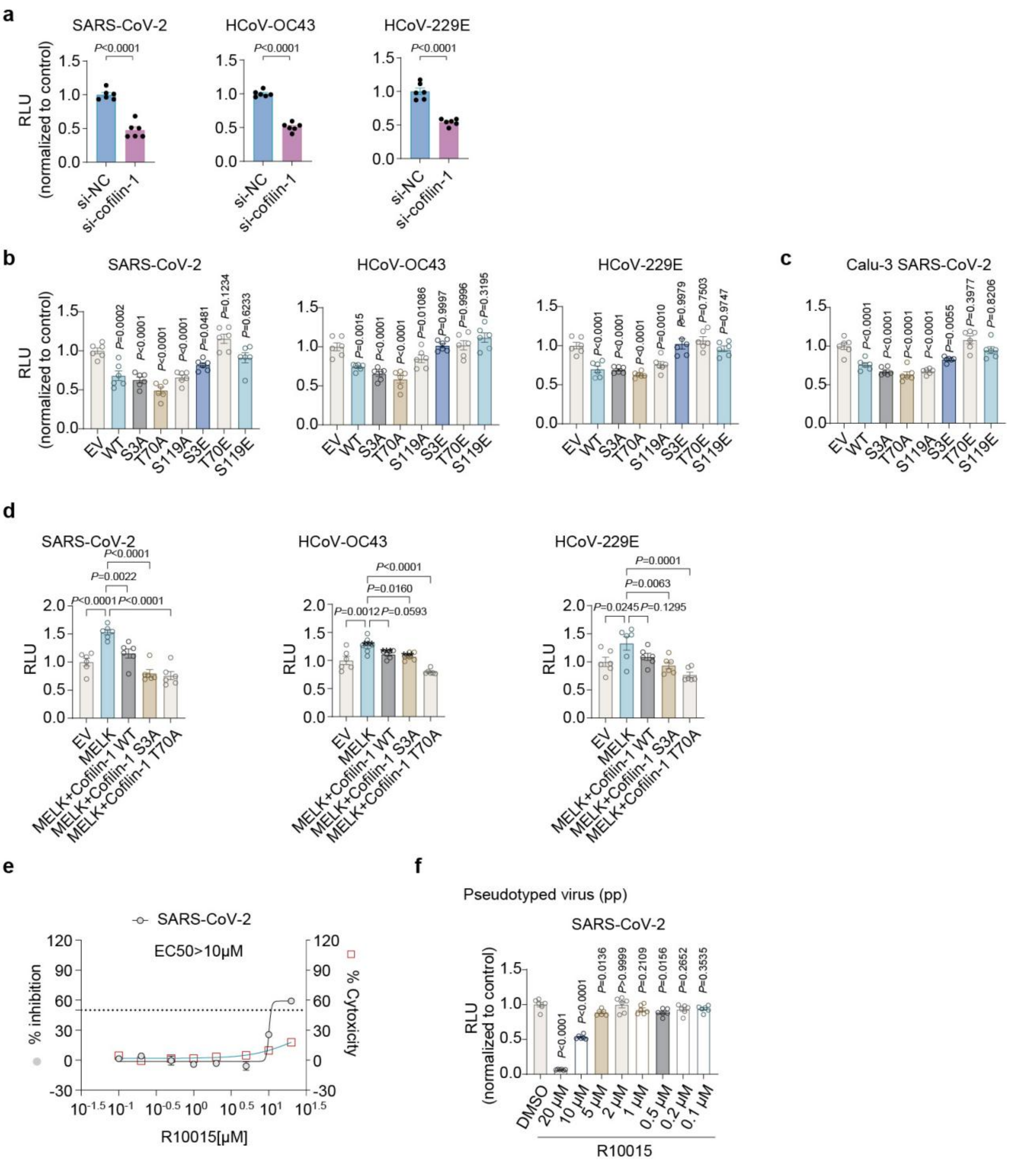
a
SARS-CoV-2
HCoV-OC43
HCoV-229E
P<0.0001
RLU (normalized to control)
si-NC
si-cofilin-1
b
c
Calu-3 SARS-CoV-2
EV
WT
S3A
T70A
S119A
S3E
T70E
S119E
d
RLU
MELK
MELK+Cofilin-1 WT
MELK+Cofilin-1 S3A
MELK+Cofilin-1 T70A
e
f
Pseudotyped virus (pp)
% inhibition
% Cytoxicity
EC50>10μM
R10015[μM]
DMSO
20 μM
10 μM
5 μM
2 μM
1 μM
0.5 μM
0.2 μM
0.1 μM
R10015

Supplementary Fig. 14. Cofilin-1 regulates the entry of coronaviruses

a, b, Quantification of viral entry efficiency in cofilin-1 knockdown(a) and (b) overexpression Huh7 cells that were inoculated with pseudoviruses bearing spike proteins from SARS-CoV-2, HCoV-229E and HCoV-OC43. Source data are provided as a SourceData file.

c, Quantification of viral entry efficiency in cofilin-1 overexpression Calu-3 cells that were inoculated with pseudoviruses bearing spike proteins from SARS-CoV-2. Viral entry efficiency was measured by normalized RLU. Source data are provided as a SourceData file.

d, Huh7 cells were co-transfected with MELK and Flag-tagged cofilin-1 WT or phosphodeficient mutants (S3A, T70A). Cells were subsequently infected with pseudoviruses bearing spike proteins of SARS-CoV-2, HCoV-OC43, or HCoV-229E. Viral entry efficiency was measured by normalized RLU. Source data are provided as a SourceData file.

e, Percent inhibition of R10015 against SARS-CoV-2 infections and cytotoxicity in Calu-3 cells. Calu-3 cells were infected with SARS-CoV-2 (MOI=1.0) in the presence of a range of concentrations for 24 hours. The infection rate was measured using IFA with N protein staining, and the cytotoxicity of the compounds was determined by the CCK8 assay. Graphs depict the percentage inhibition of viral infection (left Y-axis), percentage cytotoxicity (right Y-axis) and the concentration causing a 50% reduction in replication (EC50). Source data are provided as a SourceData file.

f, Quantification of viral entry efficiency in Calu-3 cells treated with a range of R10015 concentrations and inoculated with pseudoviruses bearing spike proteins from SARS-CoV-2. Viral entry efficiency was measured by normalized relative luciferase units (RLU). Source data are provided as a SourceData file.

Data presented in (a) are mean ± SEM of n = 6 independent biological replicates. Statistical significance was determined using two-tailed Student's t-test. Exact P values are indicated in the figure.

Data presented in (b) to (f) are mean ± SEM of n = 6 independent biological replicates. Statistical significance was determined using one-way ANOVA followed

by two-sided Dunnett's multiple comparisons test. Exact P values are indicated in the figure.

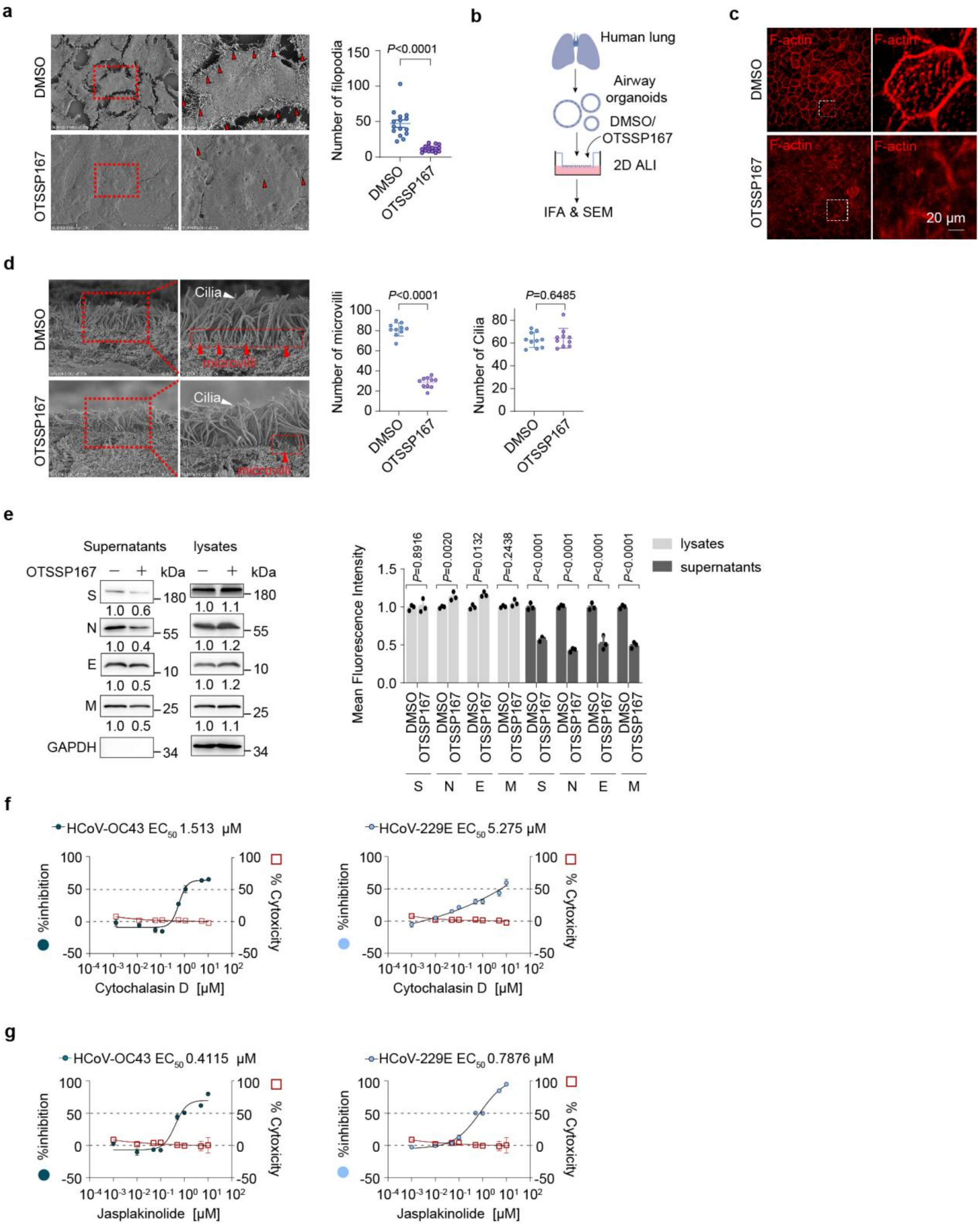
a
DMSO
OTSSP167
Number of filopodia
P<0.0001
150
100
50
0
DMSO
OTSSP167
b
Human lung
Airway organoids
DMSO/ OTSSP167
2D ALI
IFA & SEM
c
DMSO
OTSSP167
F-actin
20 μm
d
Cilia
microvilli
Number of microvilli
P<0.0001
Number of Cilia
P=0.6485
e
Supernatants
lysates
OTSSP167
kDa
S
N
E
M
GAPDH
180
55
10
25
34
1.0 0.6
1.0 0.4
1.0 0.5
1.0 0.5
1.0 1.1
1.0 1.2
1.0 1.2
1.0 1.1
Mean Fluorescence Intensity
P=0.8916
P=0.0020
P=0.0132
P=0.2438
P<0.0001
P<0.0001
P<0.0001
P<0.0001
lysates
supernatants
f
HCoV-OC43 EC50 1.513 μM
HCoV-229E EC50 5.275 μM
%inhibition
% Cytoxicity
Cytochalasin D [μM]
g
HCoV-OC43 EC50 0.4115 μM
HCoV-229E EC50 0.7876 μM
Jasplakinolide [μM]

Supplementary Fig. 15. OTSSP167 reduced the release of virus-like particles

a, OTSSP167 reduced the number of filopodia. Representative SEM images of filopodia in cells with or without OTSSP167 (0.2 μM) treatment for 3 hours. Images were representative of three independent experiments. Source data are provided as a SourceData file.

b-d, OTSSP167 reduced the number of microvilli without affecting the abundance of cilia in human proximal airway organoids. (b) The impact of OTSSP167 on microvilli in human proximal airway organoids was observed through (c) IFA and (d) SEM. In the right magnified SEM images (d), red dashed boxes indicate the microvilli regions, and red arrows point to individual microvilli. Images were representative of three independent experiments. Source data are provided as a SourceData file.

e, Effects of OTSSP167 on intracellular and extracellular assembly of VLPs. Left, Western blot of S, N, E, and M protein levels in cell supernatants and lysates after DMSO (-) or OTSSP167 (+) treatment. GAPDH served as the loading control. The gray values were quantified using ImageJ and normalized to the DMSO group (set to 1.0). Right, Quantification of fluorescence intensity from the Western blot bands, comparing intracellular (lysates, gray bars) and extracellular (supernatants, dark gray bars) S, N, E, and M protein levels. The gray values were quantified using ImageJ. Images were representative of three independent experiments. Source data are provided as a SourceData file.

f, g, Percent inhibition of (f) Cytochalasin D and (g) Jasplakinolide against SARS-CoV-2, HCoV-229E and HCoV-OC43 infections and cytotoxicity in Huh7 cells. As in Fig.1g. Source data are provided as a SourceData file.

Data presented in (a) are mean ± SD of n = 15, in (d) are mean ± SD of n = 10 from three independent biological replicates. Statistical significance was determined using two-tailed Students t -test. Exact P values are indicated in the figure.

Data presented in (e) are mean ± SEM of n = 3 independent biological replicates. Statistical significance was determined using two-way ANOVA followed by two-sided Sidaks multiple comparisons test. Exact P values are indicated in the figure.

Data presented in (f) and (g) are mean ± SEM of n = 3 independent biological replicates.

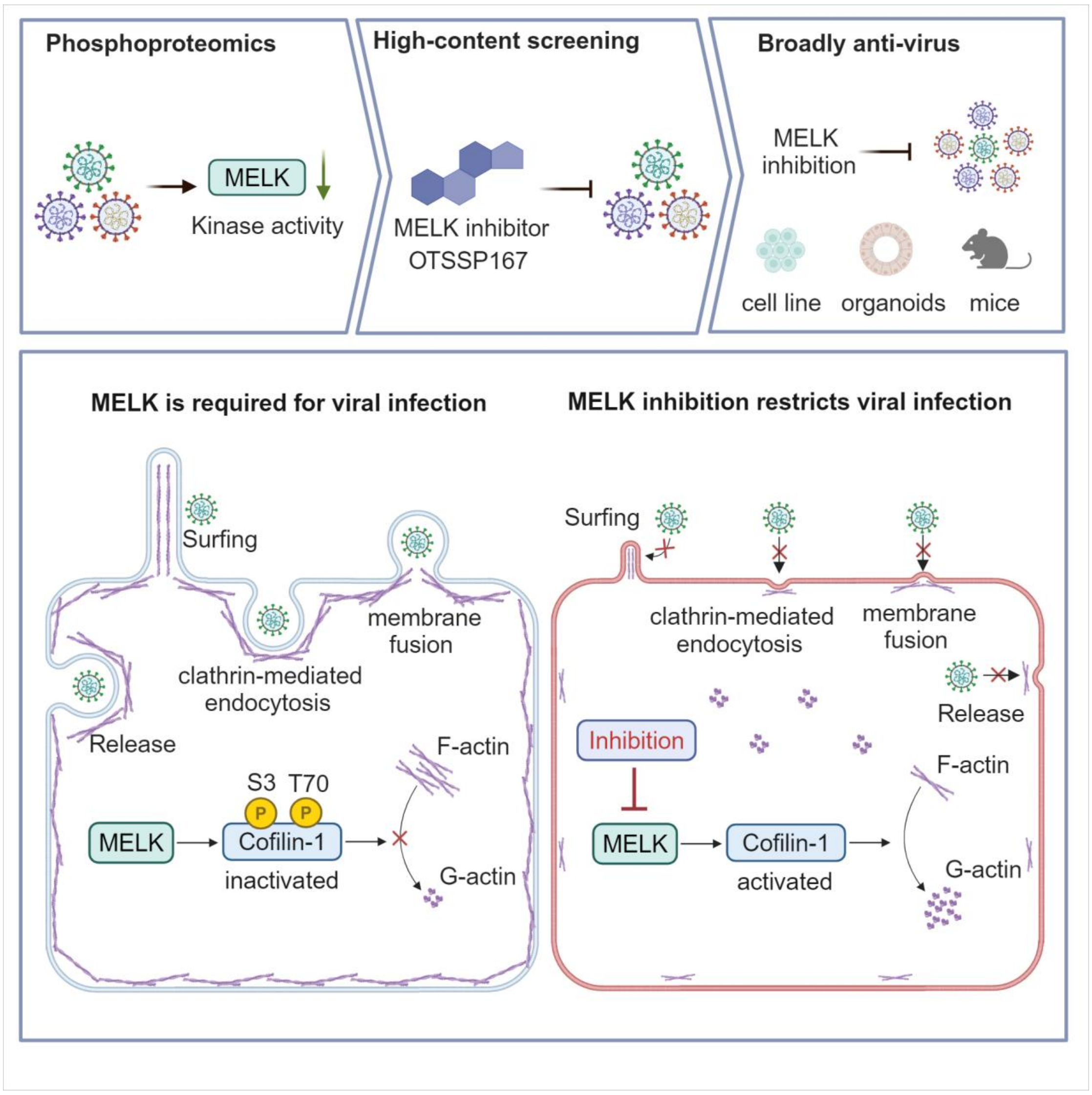
Phosphoproteomics
MELK
Kinase activity
High-content screening
MELK inhibitor
OTSSP167
Broadly anti-virus
MELK
inhibition
cell line
organoids
mice
MELK is required for viral infection
Surfing
membrane
fusion
clathrin-mediated
endocytosis
Release
F-actin
S3
T70
P
P
MELK
Cofilin-1
inactivated
G-actin
MELK inhibition restricts viral infection
Surfing
clathrin-mediated
endocytosis
membrane
fusion
Release
Inhibition
F-actin
MELK
Cofilin-1
activated
G-actin

Supplementary Fig. 16. Model

The infections of human coronavirus commonly require host protein kinase MELK, which maintains the polymerization of actin cytoskeleton by directly phosphorylating cofilin-1. Therefore, MELK inhibitor OTSSP167 exhibitsbroad-spectrum antiviral activity against diverse human coronaviruses. Created in BioRender. Kuai, Y. (2026) https://BioRender.com/jq0m3mp.

Supplementary Table 1

	Variant(Huh7)	EC50 (uM)	CC50 (uM)	Selectivity Index (SI)
OTSSP167	SARS-CoV-2 (WT)	0.0492	>10	>203.3
	SARS-CoV-2 (Alpha)	0.102	>10	>98
	SARS-CoV-2 (Beta)	0.086	>10	>116.3
	SARS-CoV-2 (Delta)	0.116	>10	>86.2
	SARS-CoV-2 (Omicron BA.1)	0.109	>10	>91.7
	SARS-CoV-2 (Omicron BA.2.3)	0.029	>10	>344.8
	SARS-CoV-2 (Omicron BA.5）	0.021	>10	>476.2
	SARS-CoV-2 (XBB.1.5)	0.023	>10	>434.8
	SARS-CoV-2 (XBB.1.16)	0.034	>10	>294.1
	SARS-CoV-2 (EG.5)	0.018	>10	>555.6
	SARS-CoV-2 (JN.1)	0.028	>10	>357.1
	HCoV-229E	0.1382	>10	>72.4
	HCoV-NL63(Huh7-ACE2)	0.057	>10	>175.4
	HCoV-OC43	0.0441	>10	>226.8
	MERS-CoV (EMC/2012)	0.054	>10	>185.2
	MERS-CoV (Nigeria)	0.0903	>10	>110.7
	MERS-CoV (GD01)	0.0258	>10	>387.6
MELK-IN-1	SARS-CoV-2	3.956	>10	>2.5
	HCoV-OC43	2.730	>10	>3.7
	HCoV-229E	2.631	>10	>3.8
MELK-8a	SARS-CoV-2	1.684	>10	>5.9
	HCoV-OC43	1.089	>10	>9.2
	HCoV-229E	3.920	>10	>2.6
Cytochalasin D	HCoV-OC43	1.513	>10	>6.6
	HCoV-229E	5.275	>10	>1.9
Jasplakinolide	HCoV-OC43	0.4115	>10	>24.3
	HCoV-229E	0.7876	>10	>12.70
R10015	SARS-CoV-2	>10	>10	-

Supplementary Table 2

	Primers
SARS-CoV-2-Forward	CACATTGGCACCCGCAATC
SARS-CoV-2-probe	FAM-ACTTCCTCAAGGAACAACATTGCCA-BBQ
SARS-CoV-2-Reverse	GAGGAACGAGAAGAGGCTTG
HCoV-OC43-Forward	CGATGAGGCTATTCCGACTAGGT
HCoV-OC43-probe	FAM-TCCGCCTGGCACGGTACTCCCT-BHQ1
HCoV-OC43-Reverse	CCTTCCTGAGCCTTCAATATAGTAACC
HCoV-229E-Forward	CGCAAGAATTCAGAACCAGAG
HCoV-229E-probe	FAM-CCACACTTCAATCAAAAGCTCCCAAATG-BHQ1
HCoV-229E-Reverse	GGGAGTCAGGTTCTTCAACAA
MERS-CoV-Forward	GCAACGCGCGATTCAGTT
MERS-CoV-probe	FAM-CTCTTCACATAATCGCCCCGAGCTCG-BHQ1
MERS-CoV-Reverse	GCCTCTACACGGGACCCATA
HCoV-NL63-Forward	AGGACCTTAAATTCAGACAACGTTCT
HCoV-NL63-probe	FAM-TAACAGTTTTAGCACCTTCCTTAGCAACCCAA ACA-BHQ1
HCoV-NL63-Reverse	GATTACGTTTGCGATTAC CAAGACT
HCoV-HKU1-Forward	GGTTGGGATTATCCTAAATGTGA
HCoV-HKU1-Reverse	CCATCATCACTCAAAATCATCATA
M-GAPDH-F	AGGTCGGTGTGAACGGATTTG
M-GAPDH-R	TGTAGACCATGTAGTTGAGGTCA
M-TNFα-F	ATAGCTCCCAGAAAAGCAAGC
M-TNFα-R	CACCCCGAAGTTCAGTAGACA
M-IL6 -F	TGGAGTCACAGAAGGAGTGGCTAAG
M-IL6 -R	TCTGACCACAGTGAGGAATGTCCAC
M-CXCL10-F	CCAAGTGCTGCCGTCATTTTC
M-CXCL10-R	GGCTCGCAGGGATGATTTCAA
si-MELK	ACTGGAGAGATGGTAGCTATA
sg-MELK-1	caccGTTGCTTATGTGCACAGCCA
sg-MELK-2	caccGCTCACCAGGAGCAAAAGGA
H-GAPDH-F	CGAGATCCCTCCAAAATCAA
H-GAPDH-R	TTCACACCCATGACGAACAT
H-ISG56-F	ACGGCTGCCTAATTTACAGC
H-ISG56-R	AGTGGCTGATATCTGGGTGC

Supplementary Fig. 2c

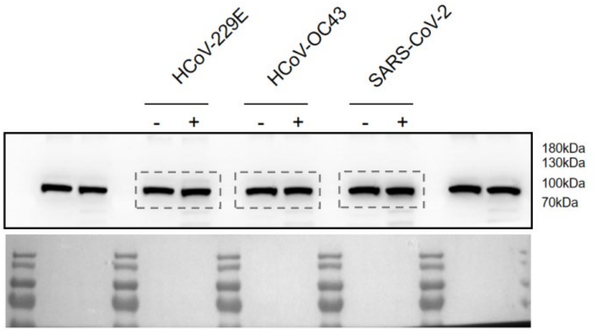

TBK1 Antibody

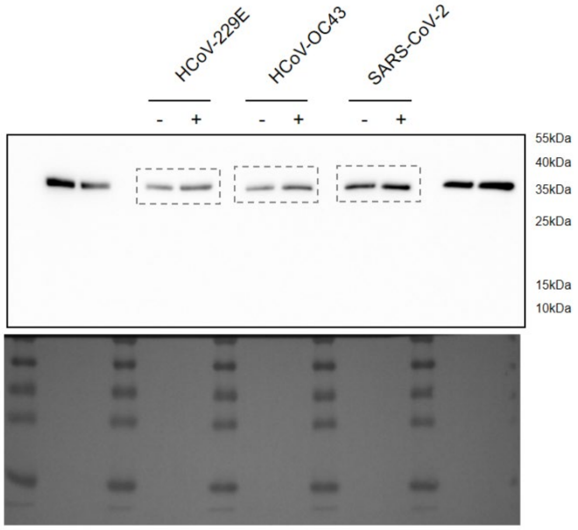

GAPDH Antibody

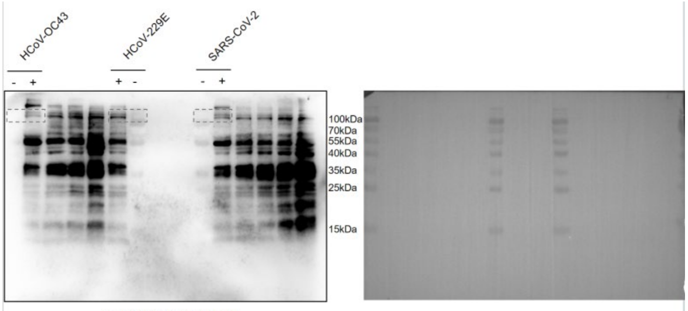

p-TBK1-S172 Antibody

Supplementary Fig. 4d

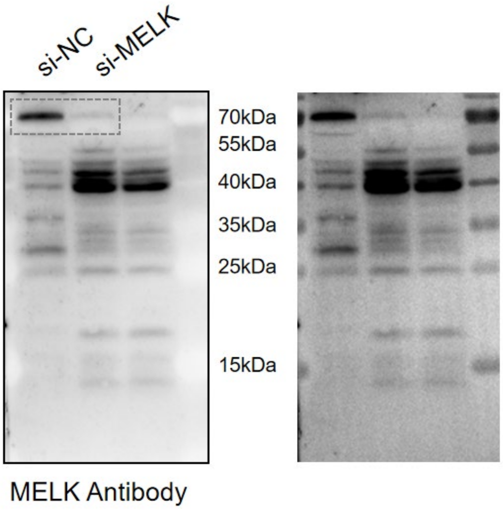

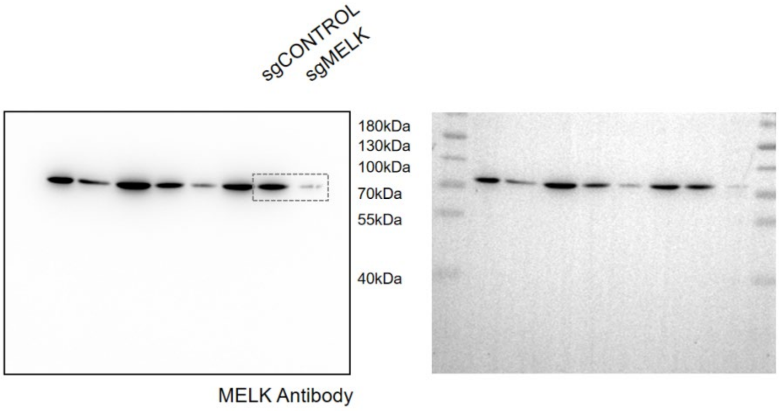

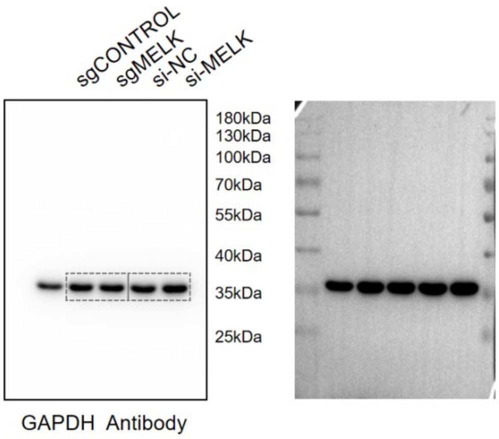

Supplementary Fig. 4e

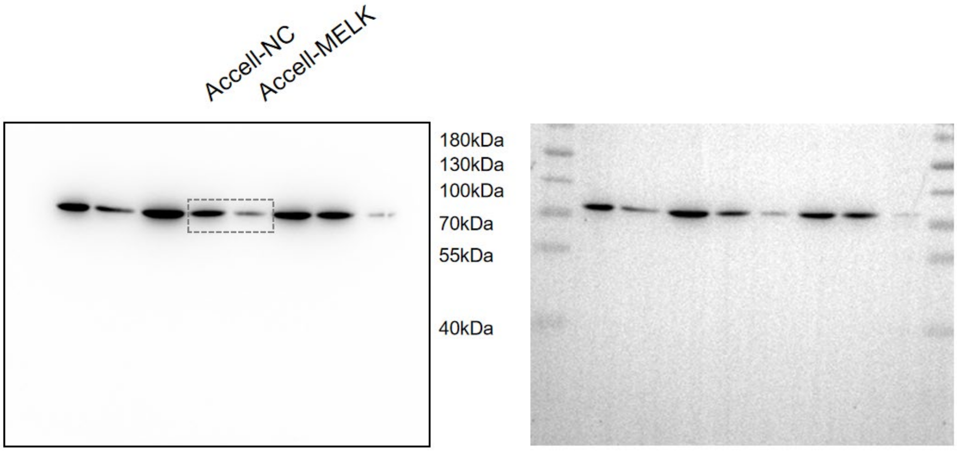

MELK Antibody

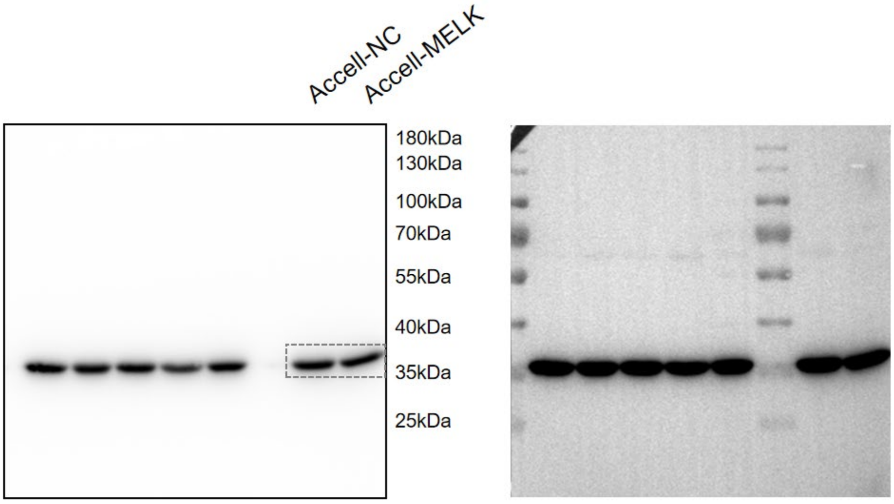

GAPDH Antibody

Supplementary Fig. 5e

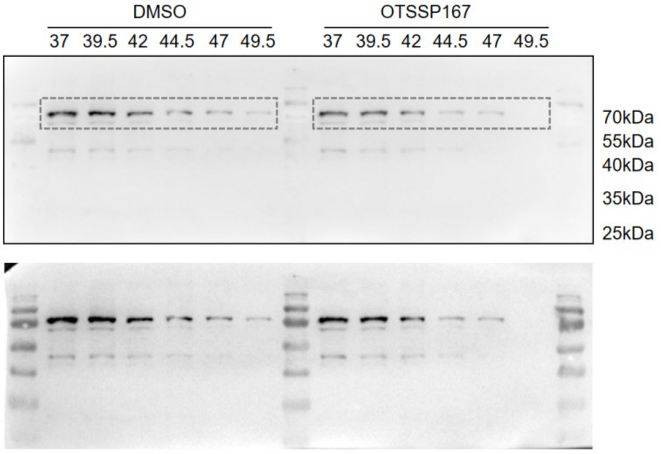

MELK Antibody

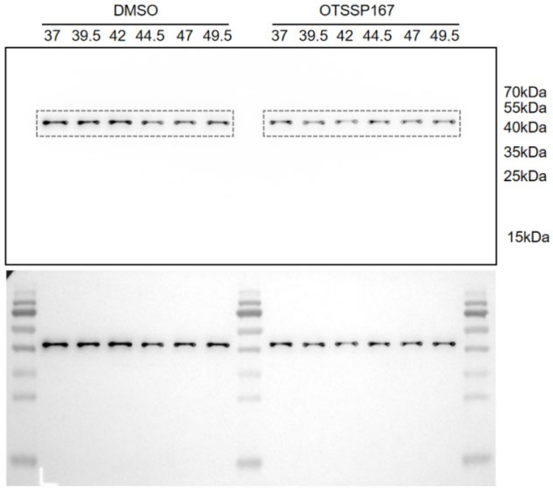

β-actin Antibody

Supplementary Fig. 5f

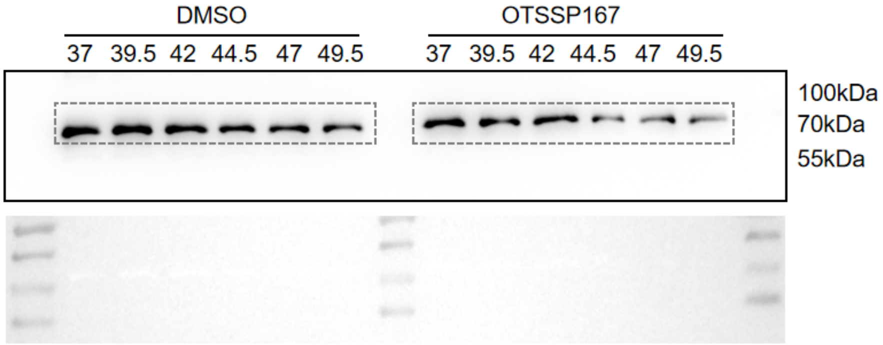

MELK Antibody

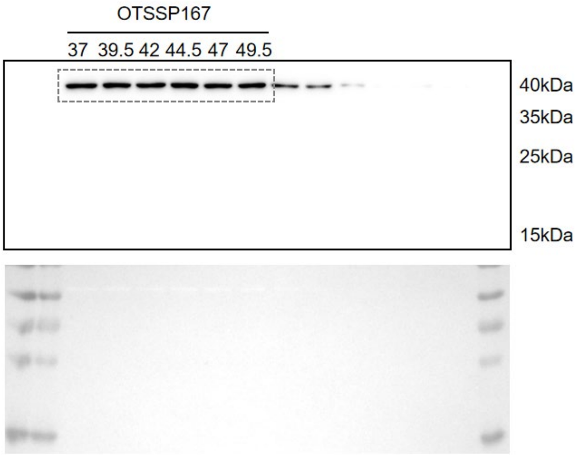

β-actin Antibody

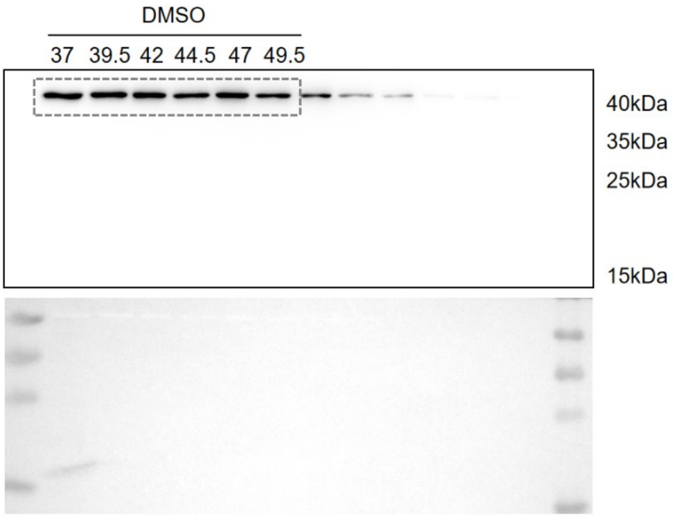

β-actin Antibody

Supplementary Fig. 5g

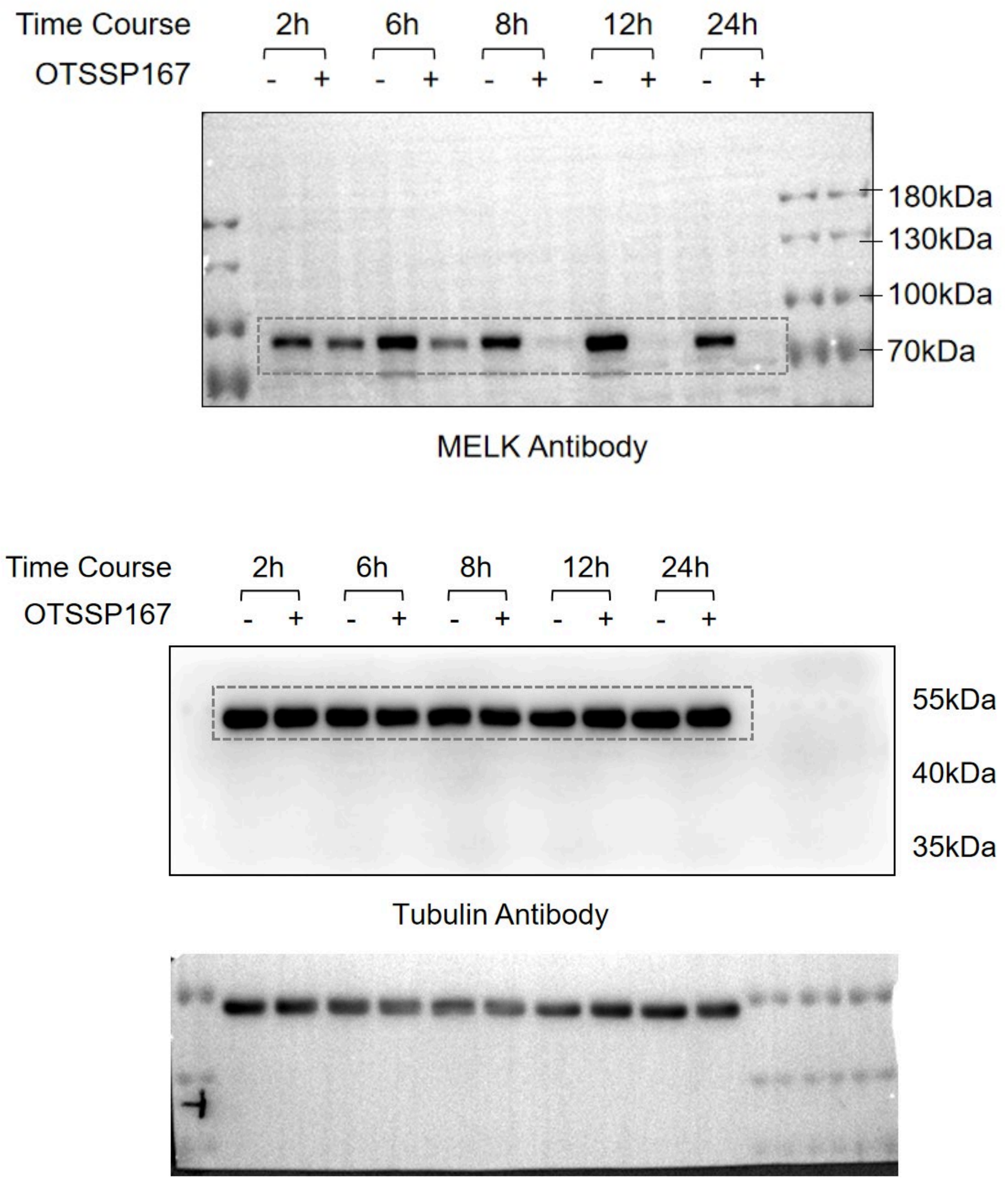

Supplementary Fig. 7g

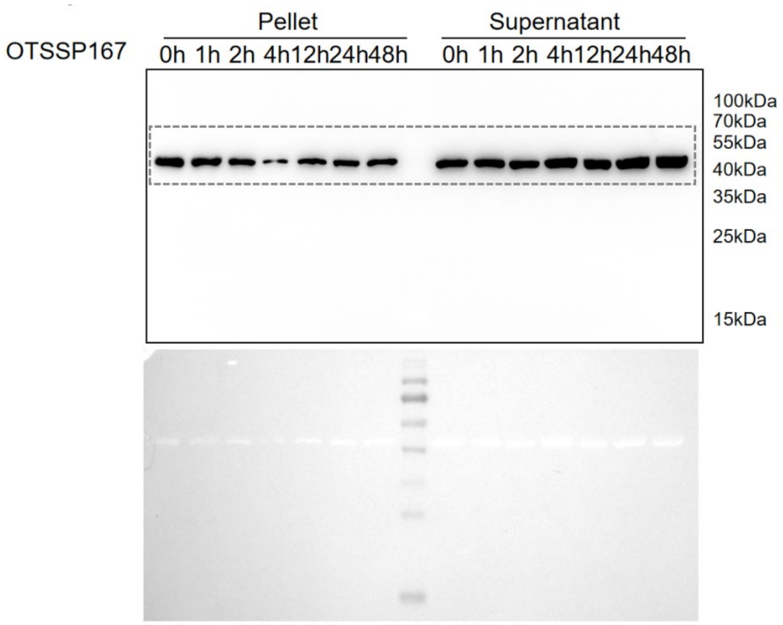

β-actin Antibody

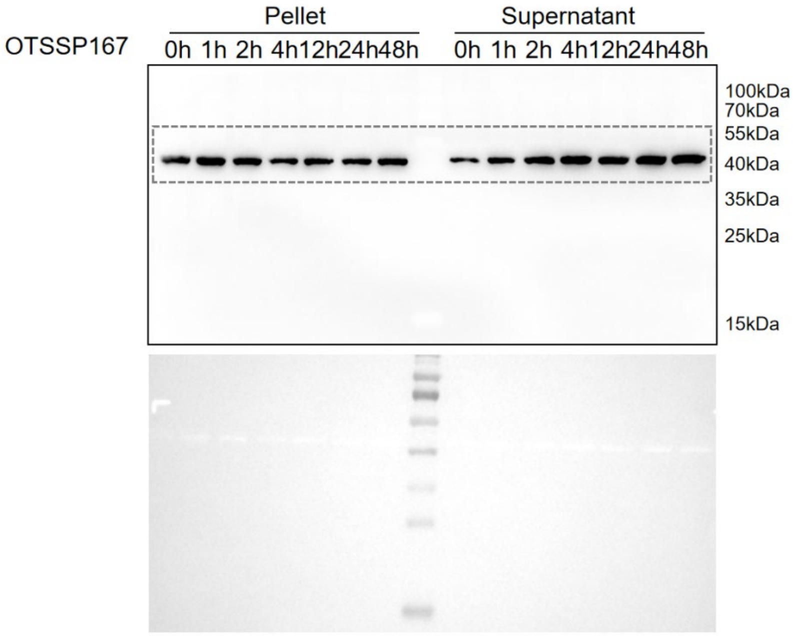

γ-actin Antibody

Supplementary Fig. 8a

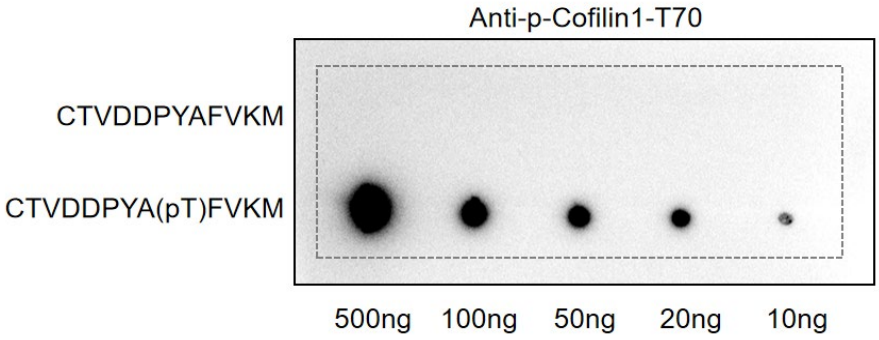

Supplementary Fig. 8b

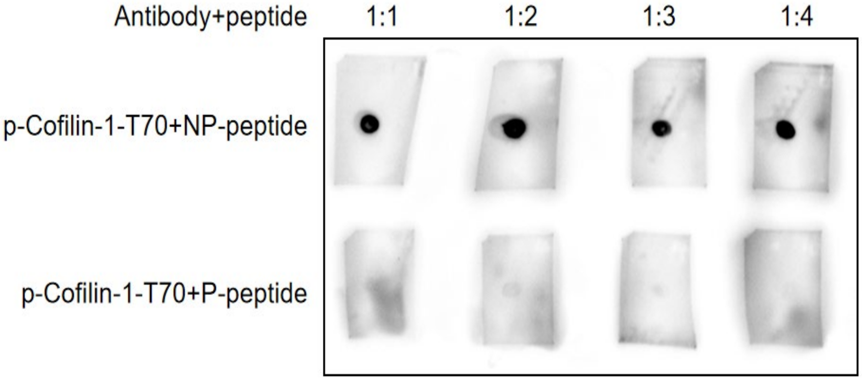

Supplementary Fig. 8c

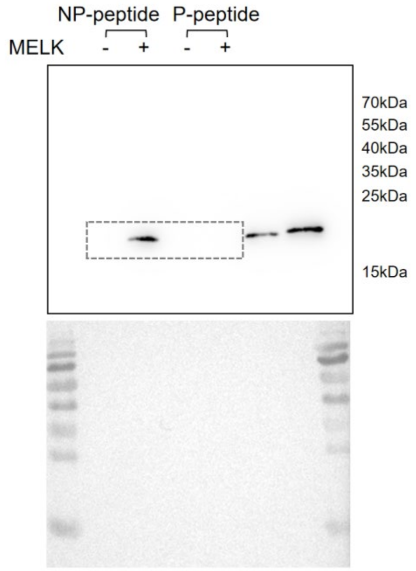

p-Cofilin1-T70 Antibody

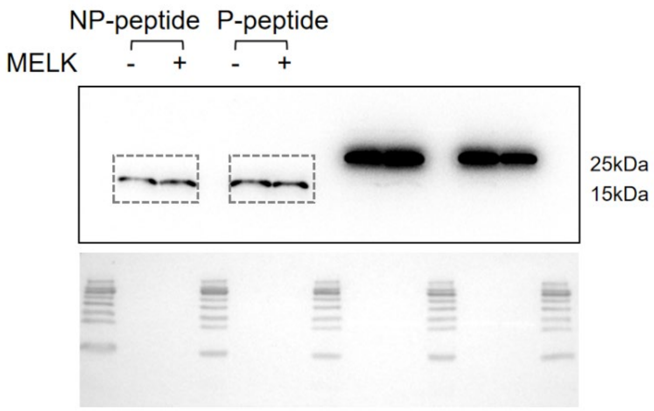

Cofilin-1 Antibody

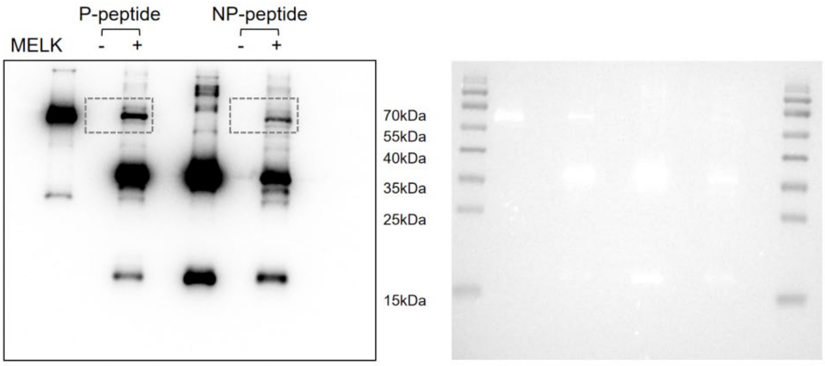

MELK Antibody

Supplementary Fig. 8d

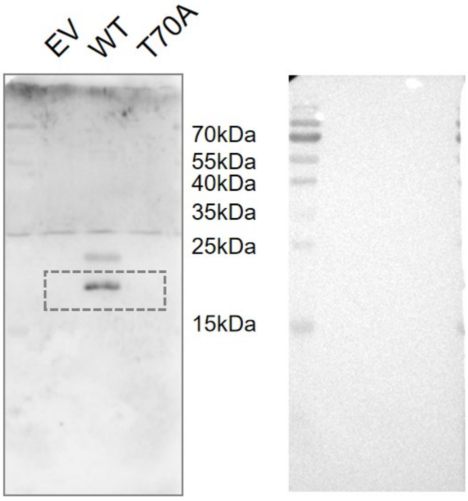

p-Cofilin1-T70
Antibody

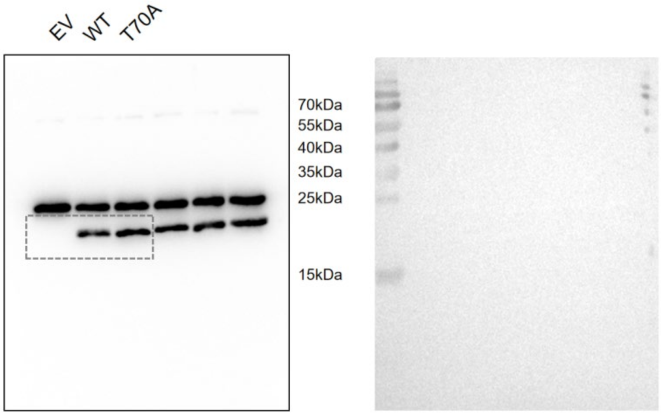

Cofilin1 Antibody

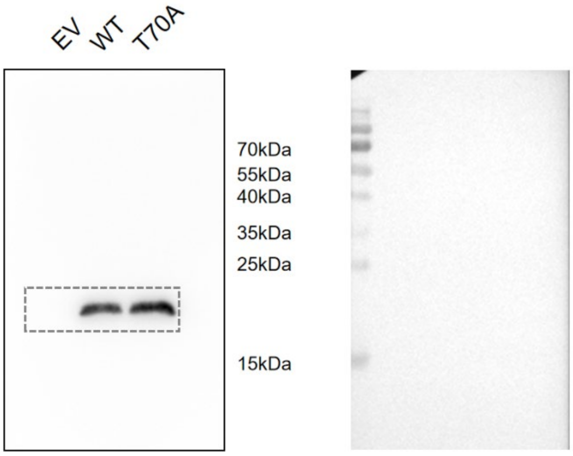

Flag Antibody

Supplementary Fig. 9f

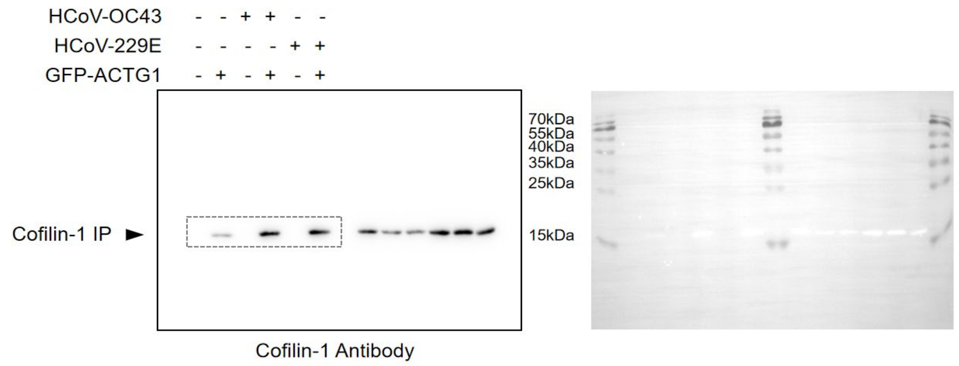

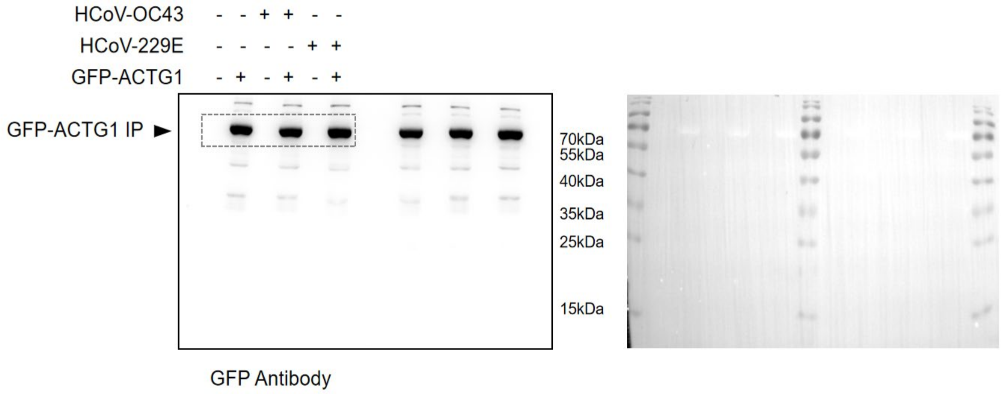

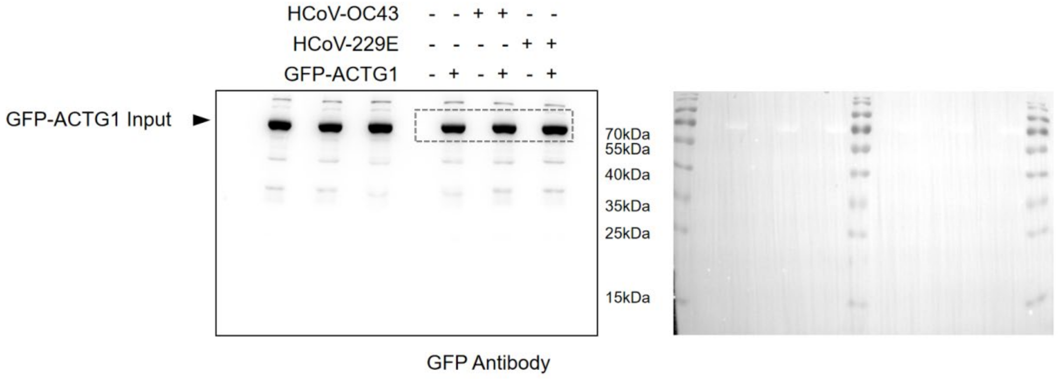

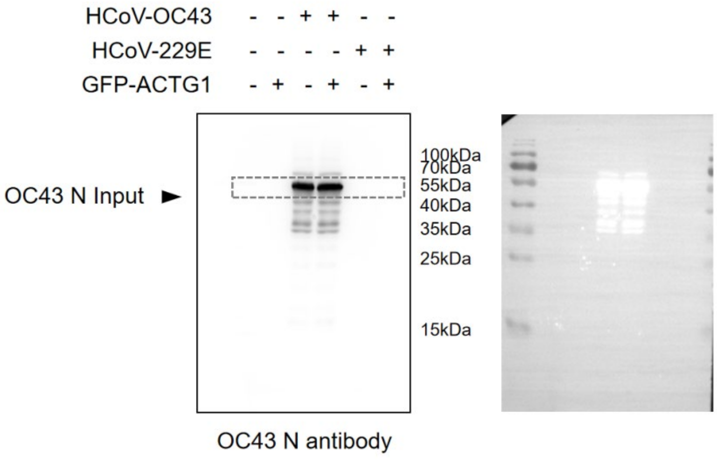
HCoV-OC43 - - + + - -
HCoV-229E - - - - + +
GFP-ACTG1 - + - + - +
OC43 N Input
100kDa
70kDa
55kDa
40kDa
35kDa
25kDa
15kDa
OC43 N antibody

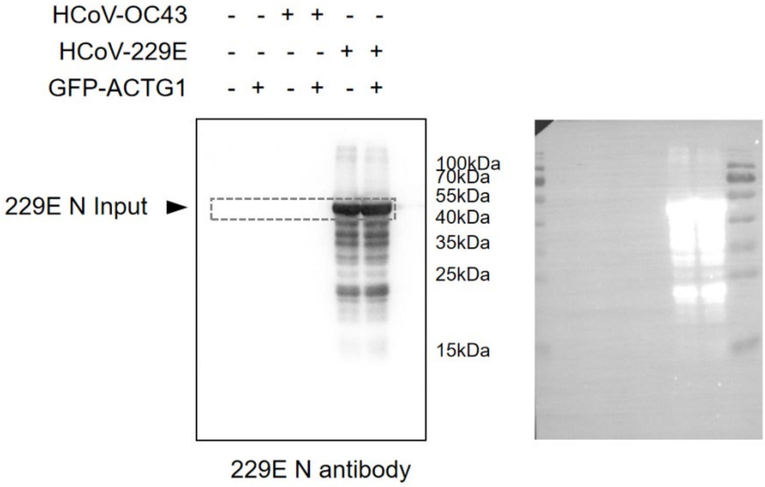
HCoV-OC43 - - + + - -
HCoV-229E - - - - + +
GFP-ACTG1 - + - + - +
229E N Input
100kDa
70kDa
55kDa
40kDa
35kDa
25kDa
15kDa
229E N antibody

Supplementary Fig. 11e

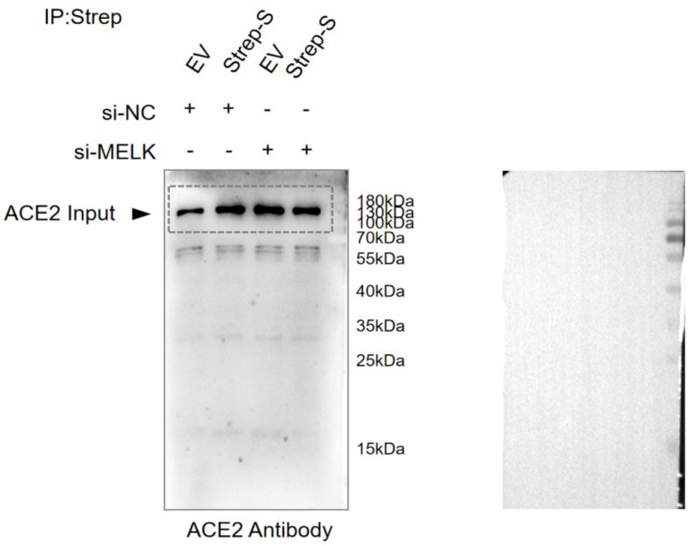

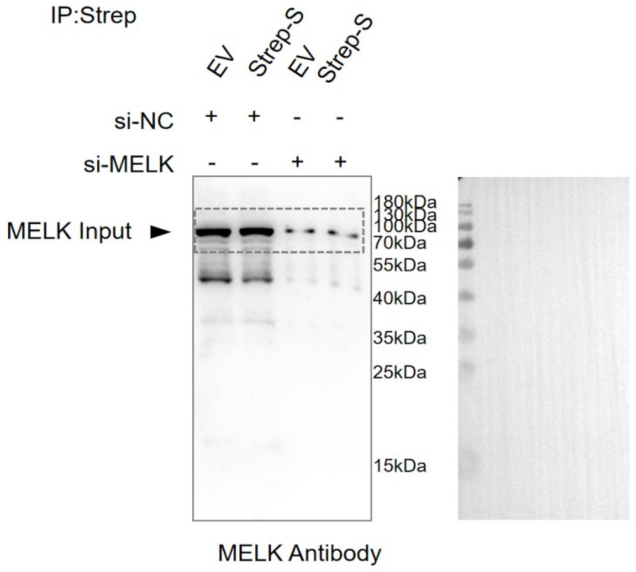

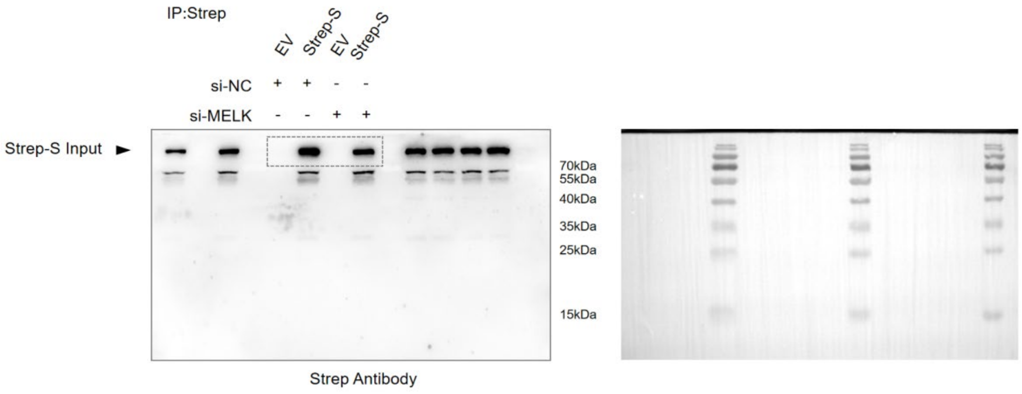

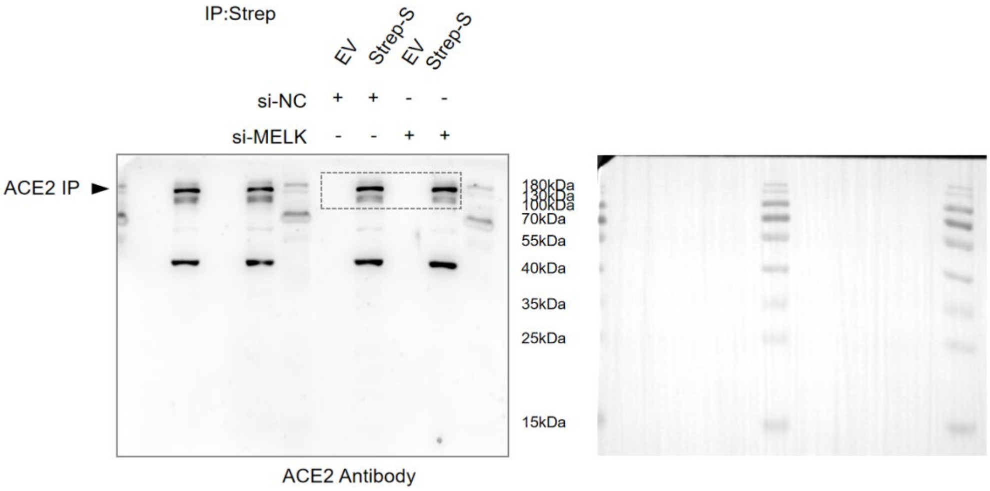
IP:Strep
EV
Strep-S
EV
Strep-S
si-NC + + - -
si-MELK - - + +
ACE2 IP
180kDa
130kDa
100kDa
70kDa
55kDa
40kDa
35kDa
25kDa
15kDa
ACE2 Antibody

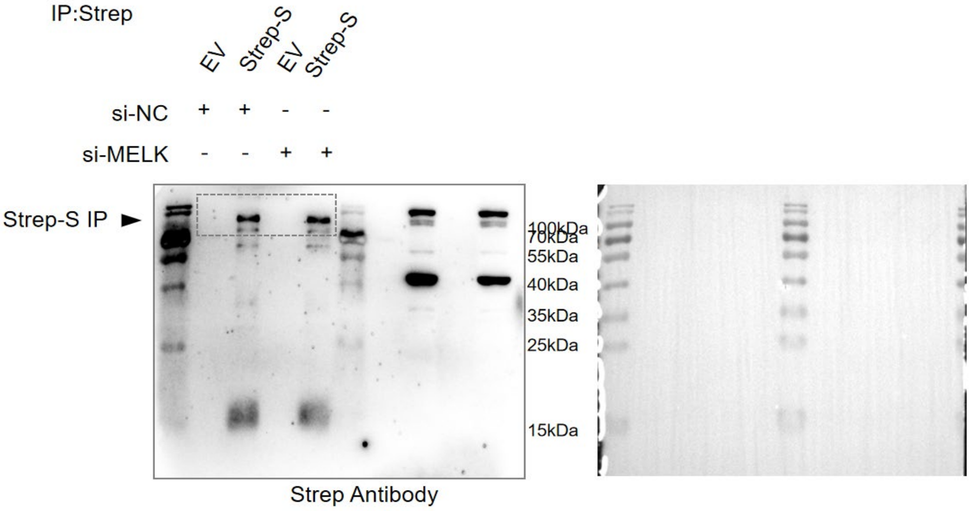
IP:Strep
EV
Strep-S
EV
Strep-S
si-NC + + - -
si-MELK - - + +
Strep-S IP
100kDa
70kDa
55kDa
40kDa
35kDa
25kDa
15kDa
Strep Antibody

Supplementary Fig. 13c

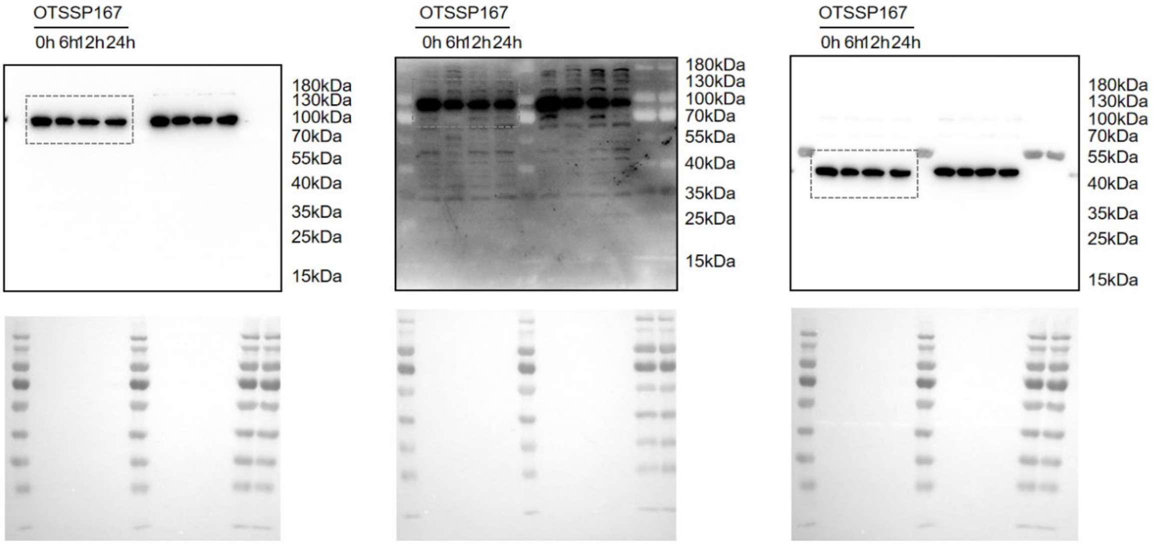

p-TBK1 S172 Antibody   TBK1 Antibody   β-actin Antibody

Supplementary Fig. 15e

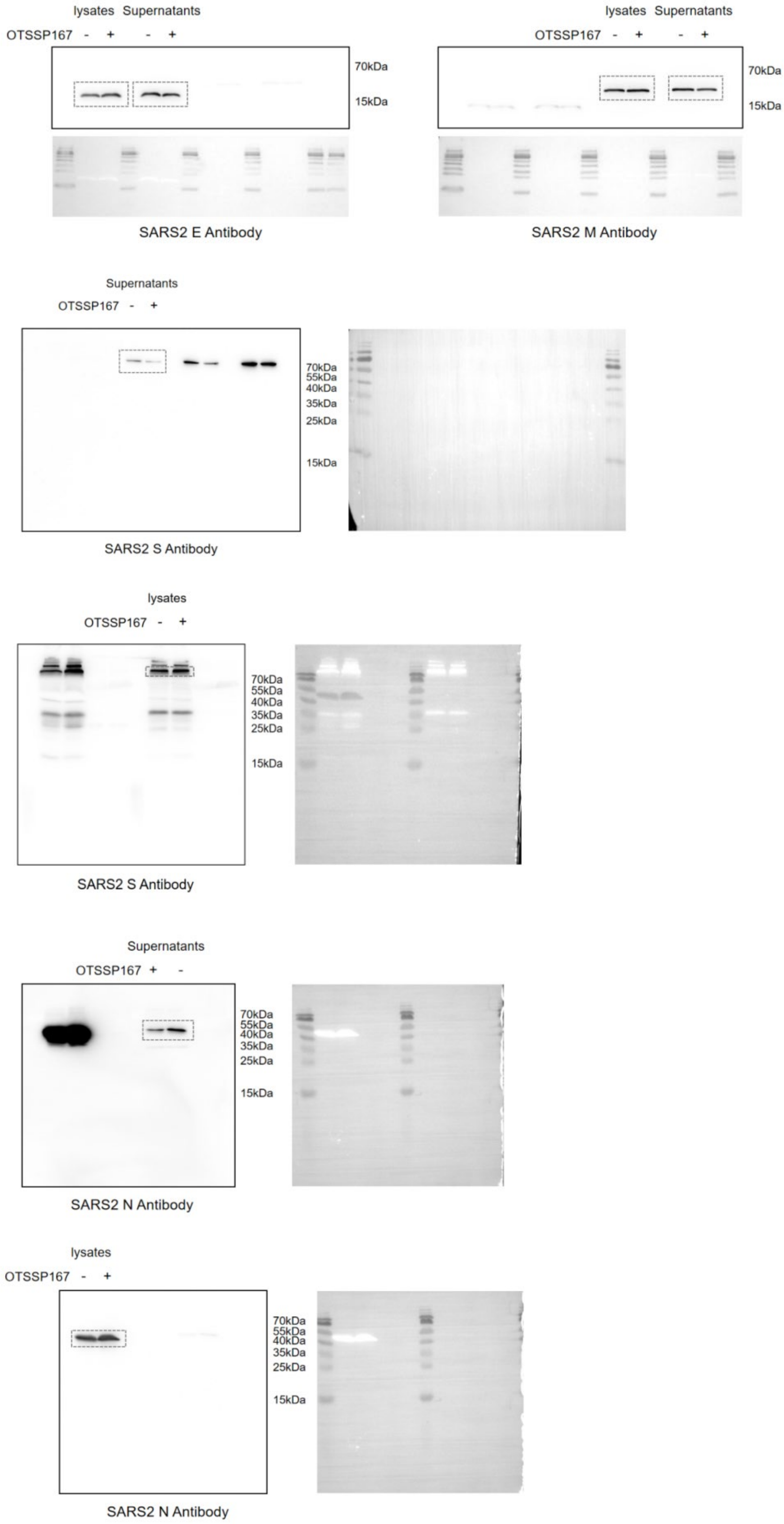

Supplement: Supplementary file 1 — Supplementary Information [file 41467_2026_72615_MOESM1_ESM.pdf]
