# Supplementary material for: MELK inhibition disrupts actin cytoskeleton and broadly restricts human coronavirus infections
Source: Nat Commun. 2026 May 6;17:6098. doi: 10.1038/s41467-026-72615-1 (PMC13358123; doi:10.1038/s41467-026-72615-1)
Supplement: Supplementary file 2 — Description Of Additional Supplementary File [file 41467_2026_72615_MOESM2_ESM.pdf]

Description of Additional supplementary files

Supplementary Movie 1

Title: DMSO treatment Description: This movie captures the live-cell imaging of actin filament dynamics in Huh7 cells treated with DMSO (vehicle control).

Supplementary Movie 2

Title: DMSO treatment (rendering) Description: This movie demonstrates the quantification and reconstruction of F-actin networks in DMSO-treated Huh7 cells using arivis Vision 4D software.

Supplementary Movie 3

Title: DMSO treatment inset1 Description: This movie shows a magnified view (inset 1) of F-actin dynamics in a DMSO-treated Huh7 cell.

Supplementary Movie 4

Title: DMSO treatment inset1 (rendering) Description: This movie demonstrates the quantification and reconstruction of F-actin networks in a magnified view (inset 1) of DMSO-treated Huh7 cells using arivis Vision 4D software.

Supplementary Movie 5

Title: DMSO treatment inset2 Description: This movie shows a magnified view (inset 2) of F-actin dynamics in a DMSO-treated Huh7 cell.

Supplementary Movie 6

Title: DMSO treatment inset2 (rendering) Description: This movie demonstrates the quantification and reconstruction of F-actin networks in a magnified view (inset 2) of DMSO-treated Huh7 cells using arivis Vision 4D software.

Supplementary Movie 7

Title: OTSSP167 treatment Description: This movie captures the live-cell imaging of actin filament dynamics in Huh7 cells treated with OTSSP167.

Supplementary Movie 8

Title: OTSSP167 treatment (rendering) Description: This movie demonstrates the quantification and reconstruction of F-actin networks in OTSSP167- treated Huh7 cells using arivis Vision 4D software.

Supplementary Movie 9

Title: OTSSP167 treatment inset Description: This movie shows a magnified view (inset 1) of F-actin dynamics in a OTSSP167 -treated Huh7 cell.

Supplementary Movie 10

Title: OTSSP167 treatment inset (rendering) Description: This movie demonstrates the quantification and reconstruction of F-actin networks in a magnified view (inset) of OTSSP167 -treated Huh7 cells using arivis Vision 4D software.

Supplementary Data 1

Title: Protein abundance and phosphorylation sites Description: This dataset contains the high-quality quantification of 7,593 proteins and 21,761 phosphorylation sites (localization probability > 0.9) identified in Huh7 cells infected with SARS-CoV-2, HCoV-229E, or HCoV-OC43, analyzed via dataindependent acquisition (DIA) mass spectrometry.

Supplementary Data 2

Title: Kinase substrate enrichment analysis (KSEA) Description: This dataset provides the inferred kinase activity changes across infections by three human coronaviruses (SARS-CoV-2, HCoV-229E, and HCoVOC43). Kinase activities were estimated by measuring and averaging the phosphorylation levels of their known substrates, providing Z-scores and significance values for kinases.
