# Supplementary material for: MELK inhibition disrupts actin cytoskeleton and broadly restricts human coronavirus infections
Source: Nat Commun. 2026 May 6;17:6098. doi: 10.1038/s41467-026-72615-1 (PMC13358123; doi:10.1038/s41467-026-72615-1)
Supplement: Supplementary file 15 — Reporting summary [file 41467_2026_72615_MOESM15_ESM.pdf]

Corresponding author(s): Jincun Zhao
Jingxian Zhao
Yaming Jiu

Last updated by author(s): Apr 4, 2026

Reporting Summary

Nature Portfolio wishes to improve the reproducibility of the work that we publish. This form provides structure for consistency and transparency in reporting. For further information on Nature Portfolio policies, see our Editorial Policies and the Editorial Policy Checklist.

Statistics

For all statistical analyses, confirm that the following items are present in the figure legend, table legend, main text, or Methods section.

n/a	Confirmed	
☐	☒	The exact sample size (*n*) for each experimental group/condition, given as a discrete number and unit of measurement
☐	☒	A statement on whether measurements were taken from distinct samples or whether the same sample was measured repeatedly
☐	☒	The statistical test(s) used AND whether they are one- or two-sided<br>*Only common tests should be described solely by name; describe more complex techniques in the Methods section.*
☒	☐	A description of all covariates tested
☐	☒	A description of any assumptions or corrections, such as tests of normality and adjustment for multiple comparisons
☐	☒	A full description of the statistical parameters including central tendency (e.g. means) or other basic estimates (e.g. regression coefficient) AND variation (e.g. standard deviation) or associated estimates of uncertainty (e.g. confidence intervals)
☐	☒	For null hypothesis testing, the test statistic (e.g. F, t, r) with confidence intervals, effect sizes, degrees of freedom and P value noted<br>*Give P values as exact values whenever suitable.*
☒	☐	For Bayesian analysis, information on the choice of priors and Markov chain Monte Carlo settings
☒	☐	For hierarchical and complex designs, identification of the appropriate level for tests and full reporting of outcomes
☐	☒	Estimates of effect sizes (e.g. Cohen's *d*, Pearson's *r*), indicating how they were calculated

Our web collection on statistics for biologists contains articles on many of the points above.

Software and code

Policy information about availability of computer code

Data collection	Proteomics and phosphoproteomics data were collected with timsTOF mass spectrometer (Bruker). Viral infection rate was measured with Celigo Image Cytometer (Nexcelom Bioscience). Viral genome copies and cellular gene expression were measured by QuantStudio 6 (ABI). The foci in focus forming assay were enumerated with ELISPOT reader (Cellular Technology Ltd. Cleveland, OH). Immunofluorescence, histology, live-cell imaging and proximity ligation assay were acquired using Zeiss LSM 980 confocal microscope. Cells on coverslips were imaged using facility line (abberior) super-resolution simulated-emission-depletion (STED) microscopy and Zeiss LSM 980 confocal microscope.TIRF Microscopy: Carl Zeiss Elyra 7 system in TIRF mode.
Data analysis	Proteomics and phosphoproteomics data were analyzed with Spectronaut software(V19). The F-actin length and the intensity of F-actin and G-actin were quantified using arivis Vision 4D software(3.4). Statistical analyses were performed with GraphPad PRISM (Versions 9).The gray values were quantified using ImageJ software. sgRNA Design: CRISPick (Broad Institute, online tool)

For manuscripts utilizing custom algorithms or software that are central to the research but not yet described in published literature, software must be made available to editors and reviewers. We strongly encourage code deposition in a community repository (e.g. GitHub). See the Nature Portfolio guidelines for submitting code & software for further information.

Data

Policy information about availability of data

All manuscripts must include a data availability statement. This statement should provide the following information, where applicable:

- Accession codes, unique identifiers, or web links for publicly available datasets
- A description of any restrictions on data availability
- For clinical datasets or third party data, please ensure that the statement adheres to our policy

The mass spectrometry proteomics data generated in this study have been deposited in the ProteomeXchange Consortium via the PRIDE partner repository under accession codes PXD057194(Global proteomics) [https://www.ebi.ac.uk/pride/archive/projects/PXD057194], PXD057224(Global phosphoproteomics) [https://www.ebi.ac.uk/pride/archive/projects/PXD057224], PXD057095 (IP-MS) [https://www.ebi.ac.uk/pride/archive/projects/PXD057095], and PXD074164 (Cofilin-1 T70 phosphorylation) [https://www.ebi.ac.uk/pride/archive/projects/PXD074164]. The processed proteomics data generated in this study are provided in the Supplementary Information/Source Data file. All other data supporting the results of this manuscript are available in the article or its Supplementary Information. Source data for all figures are provided with this paper.

Research involving human participants, their data, or biological material

Policy information about studies with human participants or human data. See also policy information about sex, gender (identity/presentation), and sexual orientation and race, ethnicity and racism.

Reporting on sex and gender	Participants were not excluded based on sex or gender.
Reporting on race, ethnicity, or other socially relevant groupings	No socially constructed or socially relevant categorization variables were used in this manuscript.
Population characteristics	Human lung tissues were collected from adult donors at the First Affiliated Hospital of Guangzhou Medical University during surgical procedures for non-infectious and non-malignant conditions. We utilized only those tissue segments verified as histologically and macroscopically normal for subsequent organoid generation. The study group encompassed both male and female patients, and no personally identifiable data were recorded. All individuals provided written informed consent.
Recruitment	Residual normal lung tissues were collected from consenting patients undergoing clinically indicated thoracic surgery. No active recruitment was conducted beyond the standard clinical workflow.
Ethics oversight	This study involving human lung tissues was reviewed and approved by the Ethics Review Committee of the First Affiliated Hospital of Guangzhou Medical University (approval number: ES-2023-193-02). All participants provided written informed consent prior to sample collection, and all samples were anonymized before analysis.

Note that full information on the approval of the study protocol must also be provided in the manuscript.

Field-specific reporting

Please select the one below that is the best fit for your research. If you are not sure, read the appropriate sections before making your selection.

☒ Life sciences ☐ Behavioural & social sciences ☐ Ecological, evolutionary & environmental sciences

For a reference copy of the document with all sections, see nature.com/documents/nr-reporting-summary-flat.pdf

Life sciences study design

All studies must disclose on these points even when the disclosure is negative.

Sample size	No statistical method was used to predetermine the sample size for our experiments, but our sample sizes are similar to or larger than those reported in previous publications in the field. At least three replicates were included in the subgroup comparison. And no statistical tool was used to predetermine sample sizes.<br>In Fig. 3, for SARS-CoV-2 BA.5 infection, six-week-old male C57BL/6 mice were administered intranasally with vehicle or OTSSP167 (3.75 mg/kg). Viral titers, pro-inflammatory cytokines, and histological analysis in lungs were determined using n=3 mice per group. For HCoV-OC43 infection, six-week-old male C57BL/6 mice were used for survival curves (n=6 mice per group) , while viral gene copies, cytokines, and histology in brains were evaluated using n=3 mice per group.
Data exclusions	No data has been excluded for statistical analysis.
Replication	All attempts at replication were successful. Replications for each experiment are detailed in the respective figure legends.
Randomization	For animal studies, mice of the same age and sex were randomly assigned to different experimental groups. For cell-based assays and omics samples, treatments were assigned to parallel cultures randomly to minimize experimental bias.
Blinding	For histological assessments and animal survival monitoring, investigators were blinded to group allocation.

Reporting for specific materials, systems and methods

We require information from authors about some types of materials, experimental systems and methods used in many studies. Here, indicate whether each material, system or method listed is relevant to your study. If you are not sure if a list item applies to your research, read the appropriate section before selecting a response.

Materials & experimental systems

n/a	Involved in the study
☐	☒ Antibodies
☐	☒ Eukaryotic cell lines
☒	☐ Palaeontology and archaeology
☐	☒ Animals and other organisms
☒	☐ Clinical data
☒	☐ Dual use research of concern
☒	☐ Plants

Methods

n/a	Involved in the study
☒	☐ ChIP-seq
☒	☐ Flow cytometry
☒	☐ MRI-based neuroimaging

Antibodies

Antibodies used

Rabbit antibodies against MERS-CoV N protein (catalog no. 40068-RP02), SARS-CoV-2 N protein (catalog no. 40143-R004), HCoV-229E N protein (catalog no. 40640-T62), HCoV-OC43 N protein (catalog no. 40643-T62), HCoV-NL63 N protein (catalog no. 40641-T62), HCoV-HKU1 N protein (catalog no. 40642-T62) were obtained from Sino Biological. Rabbit antibodies against human p-TBK1 Ser172 (catalog no. 5483), TBK1 (catalog no. 3504), Flag (catalog no. 14793S), β-actin (catalog no. 4970S), Cofilin-1 (catalog no. 5175S), p-Cofilin-1 S3 (catalog no. 3313S) were obtained from Cell Signaling Technology. Rabbit antibodies against MELK (catalog no. ab273015) was obtained from Abcam. Mouse antibodies against β-actin (catalog no. 3700S) was obtained from Cell Signaling Technology.Primary antibodies were used at a 1:1000 dilution for Western Blot (WB) and a 1:100 dilution for immunofluorescence (IFA) assays .

Validation

Commercially available antibodies (e.g., from Sino Biological, CST, and Abcam) were validated by the manufacturers for the specified applications as indicated on their official websites. For our custom p-Cofilin-1 T70 antibody, rabbits were immunized with the phosphopeptide C-TVDDPYA(pT)FVKM. The obtained serum underwent specific affinity purification followed by negative selection against the non-phosphorylated peptide (C-TVDDPYATFVKM) to eliminate non-specific binding. The antibody was purified by cross-adsorbing against the non-phosphorylated peptide to remove non-specific binders. Validation with phospho-peptide competition assay.

Eukaryotic cell lines

Policy information about cell lines and Sex and Gender in Research

Cell line source(s)

Vero E6: ATCC (CRL-1586),Vero 81: ATCC (CCL-81),Huh7: (JCRB0403),HEK293T: ATCC (CRL-3216),Calu-3: ATCC (HTB-55),HRT-18: ATCC (CCL-244),Huh7-hACE2: In-house constructed from Huh7

Authentication

All cell lines were frequently checked for cellular morphologies, growth rates and functions. All cell lines purchased from ATCC (Vero E6, Vero 81, HEK293T, HRT-18, and Calu-3) and JCRB (Huh7) were authenticated using STR profiling prior to purchase.Huh7-hACE2 line, constructed from the Huh7 line, was authenticated by confirming stable hACE2 expression via Western blot and flow cytometry.

Mycoplasma contamination

All cell lines used were tested (by PCR) and were mycoplasma free.

Commonly misidentified lines
(See ICLAC register)

No commonly misidentified lines were used in this study.

Animals and other research organisms

Policy information about studies involving animals; ARRIVE guidelines recommended for reporting animal research, and Sex and Gender in Research

Laboratory animals

For SARS-CoV-2 and HCoV-OC43 infections, six-week-old WT C57BL/6 mice were purchased from GemPharmatech Co., Ltd. (Jiangsu, China).

Wild animals

No wild animals were used in the study.

Reporting on sex

Male WT C57BL/6 mice were used in this study.

Field-collected samples

No field collected samples were used in the study.

Ethics oversight

All animal experiments in this study were performed in accordance with all relevant ethical regulations. The infection study protocols

Ethics oversight	were reviewed and approved by the Laboratory Animal Ethics Committee of the First Affiliated Hospital of Guangzhou Medical University (Approval Nos. 2021468 and 2022038). The pharmacokinetic (PK) study was additionally reviewed and approved by the Institutional Animal Care and Use Committee (IACUC) of Shenzhen Lingfu Top Biotechnology Co., Ltd. (Approval No. TOP-1PZ-GM251203).To eliminate potential variability in susceptibility associated with sex, 6 week-old male C57BL/6 mice were utilized in this study. Mice were group-housed in IVC cages in an SPF facility. The room environment was controlled with a 12/12-hour light/dark cycle, an ambient temperature of 22±2°C, and a relative humidity of 40–70%. This study involving human lung tissues was reviewed and approved by the Ethics Review Committee of the First Affiliated Hospital of Guangzhou Medical University (approval number: ES-2023-193-02). All participants provided written informed consent prior to sample collection, and all samples were anonymized before analysis.

Note that full information on the approval of the study protocol must also be provided in the manuscript.

Plants

Seed stocks	It is not relevant to our study.
Novel plant genotypes	It is not relevant to our study.
Authentication	It is not relevant to our study.
