# Supplementary material for: MELK inhibition disrupts actin cytoskeleton and broadly restricts human coronavirus infections
Source: Nat Commun. 2026 May 6;17:6098. doi: 10.1038/s41467-026-72615-1 (PMC13358123; doi:10.1038/s41467-026-72615-1)
Supplement: Supplementary file 16 — Transparent Peer Review file [file 41467_2026_72615_MOESM16_ESM.pdf]

nature portfolio

Peer Review File

MELK inhibition disrupts actin cytoskeleton and broadly restricts human coronavirus infections

Corresponding Author: Professor Jincun Zhao

This file contains all reviewer reports in order by version, followed by all author rebuttals in order by version.

Version 0:

Reviewer comments:

Reviewer #1

(Remarks to the Author)
The manuscript by Yu and co-authors has performed phosphor-proteomics in the setting of coronavirus infections. They have identified MELK kinase as a major target that can be used to inhibit coronavirus infection. MELK inhibitors have also been tested and shown to be effective both in vitro and in vivo to inhibit coronavirus infections in mouse models. The study is extensive, and the discovery of MELK as a new cofilin-1 kinase is quite novel (S3, T70, and S119 phosphorylation et al.). Given that the MELK inhibitor OTSSP167 has been tested in clinical trials for cancer treatment, the drug could also be tested for treating coronavirus infection in the future. In general, the studies are extensive, and the data are largely high quality, and conclusions are solid. The reviewer has only minor comments.

1) Given that LIMK has been considered as a major kinase for cofilin S3 phosphorylation , and LIMK inhibitors such as R10015 have been shown to block the infection of multiple viruses such as HIV and HSV-1 (PMCID: PMC5469273), would LIMK inhibitors also block SARS-CoV-2? Nevertheless, the LIMK inhibitor is not effective in blocking viral entry through endocytosis (e.g VSV-G-mediated entry), whereas the MELK inhibitor is effective in blocking SARS-CoV-2 entry. A discussion of these differences would be appreciated.

2) The Methods section has no detailed description of the in vitro virus infection experiments. How long the cells were infected? Was the drug kept during infection, after infection or washed away after infection? Actin and cofilin inhibitors can affect both viral entry and assembly, a simple time of addition experiment would be able to clarify major viral steps that are inhibited the most.

Reviewer #2

(Remarks to the Author)
This study by Yu et al., combines quantitative phosphoproteomics with a 1,805-compound kinase-inhibitor screen in Huh7 cells infected with SARS-CoV-2, HCoV-229E, and HCoV-OC43. Kinase-substrate enrichment points to reduced MELK activity across infections, and MELK inhibitor OTSSP167 is identified as a broad antiviral with low sub-µM EC50s. The authors support MELK's role via si/sgRNA and rescue by MELK over-expression, extend findings to human proximal airway organoids (six coronaviruses tested), and evaluate intranasal OTSSP167 in mouse models (with SARS-CoV-2 BA.5; HCoV-OC43). Mechanistically, MELK co-localises with F-actin, and OTSSP167 or MELK knockdown rapidly reduces F-actin, disrupts clathrin-dependent endocytosis and spike-mediated fusion, and lowers VLP egress. IP-MS plus in vitro kinase assays suggest MELK phosphorylates cofilin-1 at S3 and T70 (and S119), supported by a new anti-pT70 antibody; non-phosphorylatable cofilin mutants enhance severing and restrict entry.
Overall the dataset in this article is broad, the imaging persuasive, and the multi-system validation (cells-to-organoids-to-mice) is a strength. That said, target specificity, phospho-analysis rigor, and a few key cell-bio controls need tightening before this fully meets Nature Communications standards.

Major comments

1. OTSSP167 specificity and possible off-targets. Regarding OTSSP167 specificity and potential off-targets, the compound's known promiscuity means the current data only partially support MELK as the principal on-target mechanism. To strengthen

the causal link to MELK catalytic activity, the study should include a genetic rescue in which wild-type, but not kinase-dead, MELK restores the infection phenotype; if feasible, a gatekeeper mutant that is compatible with an orthogonal inhibitor would add a decisive chemical–genetic layer. In parallel, the authors should test at least one additional, scaffold-distinct MELK inhibitor and show that it reproduces the cellular phenotypes and preserves the EC50 rank-order across assays. Finally, direct evidence of drug–target engagement in cells (CETSA/TPP or NanoBRET) should be provided in both Huh7 cells and airway organoids to demonstrate on-target binding under the experimental conditions used.

2. Cofilin-1 phosphorylation & antibody validation. The cofilin-1 phosphorylation narrative—especially the assignment of T70—is central but currently rests heavily on Western blot data with a newly generated pT70 antibody. This claim requires full antibody validation, including phospho-peptide competition blots and demonstration that the signal is lost in a T70A cellular context. Orthogonal mass spectrometry should complement the immunochemical evidence, with site-localized spectra and confidence metrics, and ideally an in-cell phosphoproteomic comparison under OTSSP167 treatment or MELK knockdown. In short, both the reagent characterization and the site assignment need to be tightened to match the strength of the mechanistic conclusion.

3. Phosphoproteomics data analysis QC. The DIA phosphoproteomics appears technically solid (7,593 proteins; 27,079 phosphopeptides), but the downstream inference of kinase activity needs added rigor. Either raise the site-localization cutoff to a more conservative threshold (≥0.9 for pivotal findings) or justify why 0.75 is sufficient in this context; report site-level FDR and replicate-wise statistics with appropriate multiple-testing correction; and complement KSEA with motif-centric or PTM-SEA analyses to mitigate annotation bias. For transparency and reuse, deposit the raw DDA/DIA files, spectral library, and Spectronaut outputs to PRIDE with complete metadata.

4. Cell-type & timing. Phosphoproteomics is only in Huh7 at a single 24h timepoint with mixed MOIs, which likely represents mid/late infection. A limited time-course (e.g., 1–6–24 h) and/or verification of MELK/cofilin sites in airway-relevant cells (Calu3/Calu-3, HBEC ALI, or your organoids) would help seperate entry vs post-entry signaling. The authors should compare or discuss their data on the light of previously published SARS-CoV-2 phosphoproteomics articles

5. Entry vs egress. Time-of-addition suggests both. The authors show reduced transferrin uptake, blocked fusion, fewer filopodia/microvilli, and less VLP release. Please quantify G/F-actin ratios, please analyse endocytic pit dynamics if feasible, and include sgMELK in transferrin/fusion assays to show phenotypes arise from MELK loss rather than just the compound.

6. Cofilin epistasis in cells. In-vitro severing is convincing; mutants restrict pseudovirus entry. Test cellular epistasis: can cofilin-1 T70A or S3A (or double) rescue entry with MELK over-expression? Does a phospho-mimetic cofilin blunt OTSSP167's antiviral effect? This would lock in the MELK→cofilin axis.

7. In vivo pharmacology. Mouse dosing is prophylactic (−1 dpi). Include a therapeutic arm (+12–24 hpi), basic tolerability/toxicity at 3.75 mg/kg intranasal in uninfected mice, and any PK/PD or target-engagement readout (even surrogate) to support translational relevance.

8. Cell-cycle & innate immunity. MELK is cell-cycle regulated; both infection and OTSSP167 can shift cycling and secondarily impact actin. Control for cell-cycle distribution (PI/EdU/FUCCI) and discuss. Also check innate signaling (pTBK1, ISGs), as indirect antiviral effects are possible. It's important to rule out these confounders.

9. Receptor-level controls. Flow analysis shows ACE2/hAPN/DPP4 unchanged and spike–ACE2 co-IP intact under drug. Please add gating strategies, replicate counts, and stats; test fusion/entry in TMPRSS2-high Calu-3 to broaden relevance.

10. Quantification & stats. For PLA counts, filament metrics, syncytia, etc., indicate blinding, normalise to cell area, and prefer ANOVA + post-hoc over multiple t-tests. Report n, exact p, effect sizes, and define biological vs technical replicates clearly.

Minor comments

- AA residue naming. "Tyrosine 70 (T70)" should be threonine 70.

- MOI/timepoint heterogeneity. Please justify per-virus MOIs (e.g., 1.0 vs 0.1) and single 24h phospho timepoint; discuss impact on KSEA comparability.

-EC50/CC50/SI. Provide selectivity indices across viruses and cell types, a compact table would do.

- pT70 antibody. Add immunogen sequence/epitope context and validation panels to Supplement.

-Microscopy methods. State acquisition settings, randomisation/blinding, and segmentation parameters (e.g., arivis Vision 4D) to ensure reproducibility.

-Figure typos. Clean minor labels (e.g., "OTSS167" missing "P"; repeated "BA.5").

-VLP interpretation. Also show cell-associated S/E/M/N to exclude expression/assembly artifacts; quantify intra- vs extracellular.

-Organoid health. Include viability/TEER or ciliation metrics under OTSSP167 (reduced microvilli may reflect general epithelium stress).

-Data & code. Please provide PRIDE accession(s) and share analysis outputs/code, include uncropped blots and imaging source data as per journal policy.

Reviewer #3

(Remarks to the Author)
Summary:
The manuscript begins with a large-scale kinase inhibitor screen to identify compounds that perturb coronavirus infection, which showed that the MELK inhibitor OTSSP167 inhibits infection by the coronaviruses SARS-CoV-2, OC43, and 229E. The study also performs a complimentary phosphoproteomic analysis of Huh7 cells infected by SARS-CoV-2, OC43, or 229E, quantifying protein abundance and phosphoproteomic alterations following infection. By applying KSEA analysis to the phosphoproteomic data, they determine that MELK activity is reduced upon infection by all three coronaviruses. MELK small molecule inhibition, knockdown, and knockout are utilized to demonstrate broad requirement for MELK function for efficient replication of coronaviruses in cell culture, notably performing these experiments in both cancer-derived cells and primary human proximal airway organoids. Pretreatment with MELK inhibitor also reduces infection of mice with SARS-CoV-2 or OC43. The authors apply immunofluorescence microscopy to show co-localization of MELK with actin fibers, utilize AP-MS to identify an MELK interaction with cofilin, and demonstrate that MELK phosphorylates cofilin S3, T70 and S119. They demonstrate that cofilin T70A binds actin more strongly, and has augmented actin depolymerization activity. MELK inhibition appears to impact both viral entry and later stages of the virus lifecycle.

This study validates that MELK is required for optimal virus replication by multiple coronaviruses, including both low and high pathogenicity coronaviruses, in cell culture and in-vivo. It also establishes biochemical details of MELK-mediated cofilin phosphorylation, and studies the impact of MELK inhibition on the cytoskeleton. I predict these findings will have a moderate impact on the fields of virology and cell biology, and will likely stimulate interest in testing MELK inhibitors on other viruses.

My enthusiasm is moderated by some issues with the manuscript:
1) The manuscript has numerous spelling and grammatical errors, and some of the supplemental data figures are not properly labeled.
2) The literature review in the introduction is insufficient.
3) Reporting of the proteomics data is insufficient, please see notes below.
4) The choice of using hepatocyte cells for drug screening and proteomic studies needs to be addressed in the text, since the liver is not the primary site of infection.
5) The materials and methods section is incomplete.

Comments on text:
-The introduction includes insufficient literature review regarding other phosphoproteomic analyses studying coronaviruses. Please add citations and a synopsis of other mass spectrometry-based global phosphoproteomic analyses on coronavirus infection published in peer reviewed journals.
101: “Currently the role of kinases in human coronavirus infections remains largely unknown”. This seems overstated, please revise to be more precise.
145: “These findings unveil a previously undiscovered mechanism of virus-host interaction, potentially providing new avenues for antiviral strategy development.” Please revise to be more precise.

-The study’s initial drug screening and proteomics datasets utilize Huh7 cells. Since the primary site of infection of these coronaviruses is the lungs, the authors should more clearly articulate why they chose to use Huh7 cultured hepatocytes for these initial studies.

-I have concerns regarding reporting of the proteomics data. The proteomics studies were only performed in duplicate – this is not a deal-breaker but is regrettable, since it limits the statistical power of the analysis. Furthermore, the number of phosphopeptides reported is quite high for a timsTofPro system (>27,000), and the materials and methods do not list the localization score cutoff specified in Spectronaut – the localization score cutoff should be specified, and if no filter is currently implemented, the authors should re-analyze the data and report the number of localized phosphosites. The annotations in the supplemental data file also includes residues such as M, Q, and N which are not canonically phosphorylated – clarity regarding this data is needed. In addition, I see no mention of upload of the raw data to a repository. It is convention in the proteomics community to upload raw data to a repository such as PRIDE, as this is required for proper review and consideration of proteomics data for publication. For the revision, the mass spectrometry data should be uploaded to a repository, and reviewer credentials should be shared for evaluation. Upon acceptance of the manuscript, my understanding of Nature policy is that this raw data should be made available for public access.

-The authors should address in the text why the inhibition phenotype of MELK knockdown / knockout is frequently significantly smaller in magnitude than that of OTSSP167 treatment. Also, I was unable to find details of the knockout lentivirus in the methods section, this information needs to be included.

-For the mouse work, I’m curious if the authors also attempted to treat with OTSSP167 after exposure of the animal to virus (this experiment is not necessary for publication).

- It would be helpful to address the apparent dichotomy that (1) reduced MELK activity is associated with coronavirus infection, yet (2) MELK inhibition suppresses viral replication. Addressing this point would help the broader readership reconcile these observations. For example, MELK regulation during infection may be localization-specific, or residual MELK function during infection might remain sufficient to support replication.

-Numerous grammatical and spelling errors were seen throughout the manuscript. The manuscript needs to be proof-read before resubmission. Also please add page numbers to the manuscript.

Specific comments on figures:
-Figure 1G includes cytotoxicity data for OTSSP167, but other figures depicting similar experiments omit viability data. Cytotoxicity data should be included for small molecule inhibitor titration experiments on cultured cells.

-Figure 6b: When looking at a printout, it is difficult to tell the difference between the colors of each line in this plot.

-Figure 7h includes a Strep-S immunoprecipitation, but I cannot find details of this construct or IP protocol in the methods section. I’d like to understand how the Strep-S fusion construct is designed, and how the IP experiment was performed.

-Figure 7k and 7l - I would expect to see an OTSSP167-only treatment included in these experiments.

Extended data figure 2c and 2d: The heat map colorbars require quantitative labels. “High” and “Low” are not appropriate labels.
-Extended data figure 11: Please correct the legend.
-Extended data figure 12e: Legend indicates that the inhibition of virus egress caused by OTSSP167 treatment was quantified, but it’s unclear where this quantification data is located.

Version 1:

Reviewer comments:

Reviewer #1

(Remarks to the Author)

Reviewer #2

(Remarks to the Author)
This revised manuscript integrates global phosphoproteomics across three human coronaviruses (SARS-CoV-2, HCoV-229E, HCoV-OC43) with high-content kinase inhibitor screening (1,805 compounds) to identify MELK as a common host kinase required for coronavirus infections. The authors demonstrate that the MELK inhibitor OTSSP167 exhibits broad-spectrum antiviral activity (EC50 values 0.045–0.143 μM) in cell lines, human airway organoids, and two mouse infection models. Mechanistically, they show MELK directly phosphorylates cofilin-1 at S3 and T70, suppressing its actin-severing activity; inhibition of MELK therefore activates cofilin-1, disrupts F-actin dynamics, and impairs both clathrin-mediated endocytosis and spike-mediated membrane fusion required for viral entry.
The authors have performed a substantial body of additional experiments in revision. The rebuttal is thorough and responsive, addressing all major concerns raised by both reviewers. The manuscript is considerably strengthened and represents an important contribution linking host kinase signaling, actin cytoskeleton regulation, and broad-spectrum antiviral biology.

Remaining concerns

The authors have addressed the majority of the original reviewers' concerns satisfactorily. However, several points merit further attention or should be explicitly acknowledged as limitations.
1. OTSSP167 selectivity and off-target considerations
While the authors have substantially strengthened the case that MELK is the relevant target (kinase-dead rescue, multiple inhibitors, CETSA), OTSSP167 is known to be a multi-kinase inhibitor with significant activity against FLT3, DYRK1A, Aurora B, and other kinases. The scaffold-distinct inhibitors MELK-8a and MELK-IN-1 are welcome additions, but their EC50 values against viral infection are notably higher than OTSSP167 (the data in the rebuttal suggests a meaningful gap). This differential potency could reflect either pharmacological differences or off-target contributions to OTSSP167's antiviral efficacy. The authors should explicitly discuss this discrepancy and acknowledge OTSSP167's known polypharmacology in the Discussion. They might also consider presenting MELK-8a and MELK-IN-1 EC50 curves side-by-side with OTSSP167 for direct comparison.
2. Phosphoproteomics limited to a single cell type and timepoint
The phosphoproteomics discovery was performed exclusively in Huh7 cells at 24 h post-infection. While the authors

appropriately validated key functional findings in Calu-3 cells and airway organoids (as suggested by Reviewer #2), the kinase activity inference driving the entire study rests on a hepatocellular carcinoma cell line at a single late timepoint. The authors contextualize this well by referencing concordant MELK suppression in Bouhaddou et al. (Vero E6) and Wu et al. (HNE cells). Nonetheless, the absence of phosphoproteomic validation in a respiratory epithelial cell line remains a limitation that should be explicitly stated in the revised Discussion. Even showing that MELK phosphorylation status (e.g., p-MELK Western blot) changes upon infection in Calu-3 would help bridge this gap.

3. In vivo dosing regimen and translational framing

The animal experiments are encouraging but employ a prophylactic dosing regimen (first dose at -1 dpi). For a paper presenting a potential therapeutic avenue, the absence of a therapeutic (post-infection) dosing arm is notable. Additionally, intranasal delivery of a kinase inhibitor is non-standard for clinical translation. Given that OTSSP167 has entered clinical trials for oncology via systemic administration, the authors should discuss whether systemic dosing could achieve therapeutic lung concentrations and what the expected toxicity profile might be at antiviral-effective doses. The current Discussion mentions clinical trials but could be more explicit about the translational gap.

4. Cofilin-1 T70 phosphorylation stoichiometry and functional contribution

The pT70 antibody validation is now thorough (dot blot, peptide competition, T70A mutant, LC-MS/MS). However, the relative functional contribution of T70 versus S3 phosphorylation to the overall antiviral phenotype is not clearly resolved. Both S3A and T70A cofilin-1 mutants show enhanced antiviral activity, but the magnitudes appear similar. Quantitative comparison (e.g., double mutant S3A/T70A versus each single mutant) would clarify whether these sites act additively or redundantly. Additionally, T70 was not detected in the global phosphoproteomics of infected cells (only in the overexpression LC-MS/MS experiments), which raises the question of its stoichiometry under physiological conditions. This should be discussed.

Minor Points

1. The kinase activity inference now uses three complementary approaches (KSEA, PhosR motif-centric, PTM-SEA), which is excellent. However, the authors should clarify how concordance between methods was defined and report the overlap statistics more explicitly. For instance, how many kinases were identified by all three methods versus only one?
2. The increased phosphosite localization cutoff (>0.9 from the default >0.75) is appropriate and welcome. The authors should report how many phosphosites were retained after this stricter filtering and confirm that all downstream analyses (including kinase activity inference) use only Class I sites at the higher threshold.
3. The cell cycle analysis (Extended Data Fig. 13a-b) showing no G2/M arrest at 6 h and 12 h but arrest at 24 h is important for interpreting the antiviral mechanism. However, the phosphoproteomics itself was performed at 24 h. The authors should acknowledge that some of the phosphorylation changes captured may reflect cell cycle perturbation in addition to, or downstream of, viral infection.
4. The comparison with LIMK inhibitor R10015 is a valuable addition. The mechanistic distinction (LIMK phosphorylates only S3, MELK phosphorylates both S3 and T70) is compelling. Could the authors comment on whether other kinases reported to phosphorylate cofilin-1 (e.g., TESK1/2 at S3) might also contribute to the observed phenotype?
5. Throughout the manuscript, MOI values vary across virus types (SARS-CoV-2 MOI=0.5 or 1.0, HCoV-229E MOI=0.1, HCoV-OC43 MOI=0.5) without clear justification. While this likely reflects different viral replication kinetics, a brief explanation in the Methods would be helpful.
6. The manuscript states that OTSSP167 has been tested in clinical trials for solid tumors and leukemia but does not mention its known dose-limiting hematologic toxicity (thrombocytopenia, neutropenia). Given that this is positioned as a potential antiviral therapeutic, acknowledging these toxicity signals would provide more balanced translational perspective.

Reviewer #3

(Remarks to the Author)

The manuscript has been substantially improved, I compliment the authors on their hard work and comprehensive analyses. Most of my comments have been addressed.

The manuscript would benefit from additional proof reading for spelling and grammar, and I have highlighted some typos. Also, in the text several sentences are unclear or overstated. Cloning of the Strep-S construct is still ambiguous and this needs to be clarified.

My comments are outlined below, designated by line number or subfigure:

Line 60: “Protein kinases control nearly all biological processes in eukaryotic cells”. This overreaches a bit, please temper this statement.

66: “Inhibition of MELK activity by OTSSP167 or it’s expression…” Please rephrase for clarity.

93-94: “almost all aspects of cell life.” I suggest rephrasing.

251: “we employed siRNA or sgRNA” – I suggest rephrasing to “we employed siRNA knockdown or Cas9 knockout“

298-300: “We challenged mice with...OC43…followed by intranasal delivery of OTSSP167 at -1 dpi”. According to the figure and ‘-1 dpi’, the OTSSP167 was added before OC43 in this experiment.

305-306: “The attenuated virus replication and expression of pro-inflammatory markers resulted in less histological changes.” More conservative phrasing might use the phrase ‘…was accompanied by…’

563: “mediate most of the signal transduction in eukaryotic cells through phosphorylation”. I’d soften this statement – there are many mechanisms of signal transduction.

597: “surfing” is an unusual way to phrase this.

785: Please specify the online design tool used to design the sgRNAs.

807-820: The cloning of the Strep-S construct is still unclear to me. Does the naming order “Strep-S” indicate that the strep tag is located at the N-terminus of Spike? If this is correct, please explain how it was cloned to avoid perturbing processing

of the signal peptide at the N-terminus of Spike.

Extended Data Fig. 1 g and h – please describe y-axis for the reader.
1452: Number 11 seems out of place.
Extended Data Fig 4 f-i – please state the concentration tested.
Extended Data Fig 6d: “FFU/garm”. I think this is a typo.
Extended data Fig 15 d: It is somewhat unclear what data are represented by the plots to the right of the SEM images.

Open Access This Peer Review File is licensed under a Creative Commons Attribution 4.0 International License, which permits use, sharing, adaptation, distribution and reproduction in any medium or format, as long as you give appropriate credit to the original author(s) and the source, provide a link to the Creative Commons license, and indicate if changes were made.
In cases where reviewers are anonymous, credit should be given to 'Anonymous Referee' and the source.
The images or other third party material in this Peer Review File are included in the article’s Creative Commons license, unless indicated otherwise in a credit line to the material. If material is not included in the article’s Creative Commons license and your intended use is not permitted by statutory regulation or exceeds the permitted use, you will need to obtain permission directly from the copyright holder.
To view a copy of this license, visit https://creativecommons.org/licenses/by/4.0/

Reviewer #1 (Remarks to the Author):

The manuscript by Yu and co-authors has performed phosphor-proteomics in the setting of coronavirus infections. They have identified MELK kinase as a major target that can be used to inhibit coronavirus infection. MELK inhibitors have also been tested and shown to be effective both in vitro and in vivo to inhibit coronavirus infections in mouse models. The study is extensive, and the discovery of MELK as a new cofilin-1 kinase is quite novel (S3, T70, and S119 phosphorylation et al.). Given that the MELK inhibitor OTSSP167 has been tested in clinical trials for cancer treatment, the drug could also be tested for treating coronavirus infection in the future. In general, the studies are extensive, and the data are largely high quality, and conclusions are solid. The reviewer has only minor comments.

We sincerely appreciate the positive evaluation and constructive feedback of our work provided by the Reviewer. We have carefully addressed the specific minor comments raised by the Reviewer point-by-point below.

1) Given that LIMK has been considered as a major kinase for cofilin S3 phosphorylation , and LIMK inhibitors such as R10015 have been shown to block the infection of multiple viruses such as HIV and HSV-1 (PMCID: PMC5469273), would LIMK inhibitors also block SARS-CoV-2? Nevertheless, the LIMK inhibitor is not effective in blocking viral entry through endocytosis (e.g VSV-G-mediated entry), whereas the MELK inhibitor is effective in blocking SARS-CoV-2 entry. A discussion of these differences would be appreciated.

We thank the Reviewer for raising this important question regarding the comparison of the functional mechanisms between MELK and LIMK, as well as their respective inhibitors.
Following the Reviewer’s suggestion, we examined the effect of the LIMK inhibitor R10015 on SARS-CoV-2 infection using both live virus and pseudovirus entry assays. Our data indicated that R10015 is capable of inhibiting SARS-CoV-2 infection, but significant efficacy was observed only at concentrations greater than 10 μM. The pseudovirus data indicated that R10015 significantly suppresses the entry stage of SARS-CoV-2 (results shown below panel a, b).
Mechanistically, LIMK primarily phosphorylates Cofilin-1 at Ser3 to inhibit its actin-severing activity. In contrast, MELK, as identified in our study, phosphorylates Cofilin-1 not only at Ser3 but also at Thr70. Our mutagenesis data demonstrated that phosphorylation at Thr70 is critical for suppressing Cofilin-1 activity (in Fig.6b of our original manuscript). Therefore, inhibiting MELK leads to the dephosphorylation of multiple sites (S3/T70). We postulate that this leads to a greater degree of Cofilin-1 activation compared to LIMK

inhibition, resulting in a more significant disruption of the actin network-regulated endocytosis process required for viral entry.

We have added these new results to the revised manuscript (Extended Data Fig. 14e, f) and cited the reference (PMCID: PMC5469273), including a detailed discussion in the Discussion section (Page 19, Lines 537-544, in red).

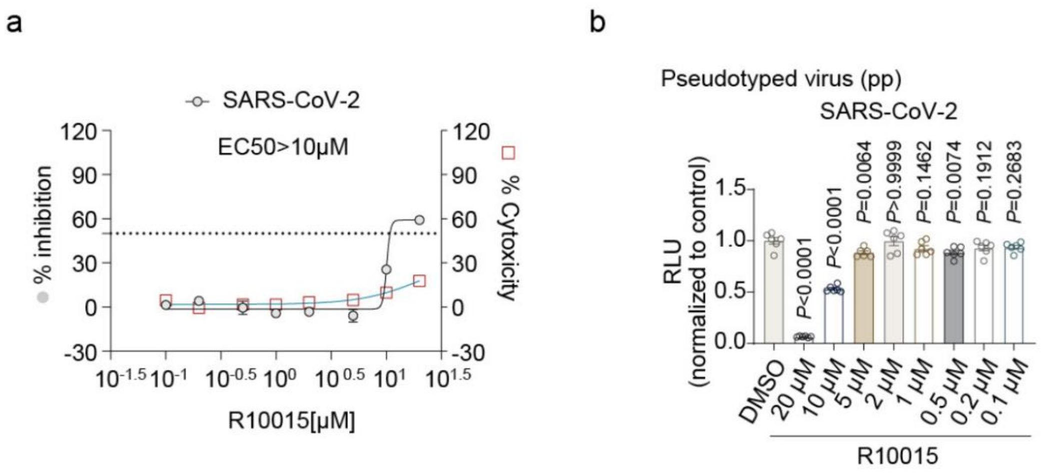

Figure legend:

a, Percent inhibition of R10015 against SARS-CoV-2 infections and cytotoxicity in Calu-3 cells. Calu-3 cells were infected with SARS-CoV-2 (MOI=1.0) in the presence of a range of concentrations for 24 hours. The infection rate was measured using IFA with N protein staining, and the cytotoxicity of the compounds was determined by the CCK8 assay. Graphs depict the percentage inhibition of viral infection (left Y-axis), percentage cytotoxicity (right Y-axis) and the concentration causing a 50% reduction in replication (EC50).

b, Quantification of viral entry efficiency in Calu-3 cells treated with a range of R10015 concentrations and inoculated with pseudoviruses bearing spike proteins from SARS-CoV-2. Viral entry efficiency was measured by normalized relative luciferase units (RLU).

Data presented in (a) and (b) are mean ± SEM of n = 3 independent biological replicates. Statistical significance was determined using one-way ANOVA followed by Dunnett's multiple comparisons test. Exact P values are indicated in the figure.

2) The Methods section has no detailed description of the in vitro virus

infection experiments. How long the cells were infected? Was the drug kept during infection, after infection or washed away after infection? Actin and cofilin inhibitors can affect both viral entry and assembly, a simple time of addition experiment would be able to clarify major viral steps that are inhibited the most.

We thank the Reviewer for this thoughtful suggestion. We apologize for the lack of detail in the original manuscript. We have now updated the Methods section to include a detailed description of the infection protocols, specifying the infection duration, drug treatment windows, and wash steps (Pages 23-24, Lines 667-675, in red).
We fully agree with the Reviewer that actin and cofilin inhibitors can affect both viral entry and assembly. Indeed, in Figure 7a of the original manuscript, we performed a time-of-addition experiment which demonstrated that OTSSP167 inhibits multiple stages of the viral life cycle, particularly exhibiting strong antiviral effects at the early entry stage, a finding that was further confirmed by our subsequent pseudovirus entry assays.

Reviewer #2 (Remarks to the Author):

This study by Yu et al., combines quantitative phosphoproteomics with a 1,805-compound kinase-inhibitor screen in Huh7 cells infected with SARS-CoV-2, HCoV-229E, and HCoV-OC43. Kinase-substrate enrichment points to reduced MELK activity across infections, and MELK inhibitor OTSSP167 is identified as a broad antiviral with low sub-μM EC50s. The authors support MELK's role via si/sgRNA and rescue by MELK over-expression, extend findings to human proximal airway organoids (six coronaviruses tested), and evaluate intranasal OTSSP167 in mouse models (with SARS-CoV-2 BA.5; HCoV-OC43). Mechanistically, MELK co-localises with F-actin, and OTSSP167 or MELK knockdown rapidly reduces F-actin, disrupts clathrin-dependent endocytosis and spike-mediated fusion, and lowers VLP egress. IP-MS plus in vitro kinase assays suggest MELK phosphorylates cofilin-1 at S3 and T70 (and S119), supported by a new anti-pT70 antibody; non-phosphorylatable cofilin mutants enhance severing and restrict entry.

Overall the dataset in this article is broad, the imaging persuasive, and the multi-system validation (cells-to-organoids-to-mice) is a strength. That said, target specificity, phospho-analysis rigor, and a few key cell-bio controls need tightening before this fully meets Nature Communications standards.

We sincerely appreciate the Reviewer's comprehensive summary and for providing valuable and constructive feedback. We have followed the Reviewer's suggestions, performed more experiments and analyses, carefully addressed the Reviewer's concerns and prepared a point-by-point response as follows.

Major comments

1. OTSSP167 specificity and possible off-targets. Regarding OTSSP167 specificity and potential off-targets, the compound's known promiscuity means the current data only partially support MELK as the principal on-target mechanism. To strengthen the causal link to MELK catalytic activity, the study should include a genetic rescue in which wild-type, but not kinase-dead, MELK restores the infection phenotype; if feasible, a gatekeeper mutant that is compatible with an orthogonal inhibitor would add a decisive chemical–genetic layer. In parallel, the authors should test at least one additional, scaffold-distinct MELK inhibitor and show that it reproduces the cellular phenotypes and preserves the EC50 rank-order across assays. Finally, direct evidence of drug–target engagement in cells (CETSA/TPP or NanoBRET) should be provided in both Huh7 cells and airway organoids to demonstrate on-target binding under the experimental conditions used.

We thank the Reviewer for raising this important question. In our original

manuscript (Fig.2h, Extended Data Fig.6a,b), we had performed wild-type MELK rescue assay and showed that the antiviral activities of scaffold-distinct inhibitors matched their rank-order of potency against MELK, supporting an on-target mechanism. We fully agree that a kinase-dead control and direct target engagement assays (such as CETSA) would significantly strengthen the conclusion regarding OTSSP167 specificity. Accordingly, we have performed the following experiments.

1. **Genetic Rescue Experiment**: Following the Reviewer's suggestion, we performed rescue experiments using both wild-type and kinase-dead (T167A) MELK (PMID: 16216881).The results showed that only the wild-type MELK, but not kinase-dead (T167A) mutant, could restore the infection phenotype (results shown below panel a). We have added these new results to the revised manuscript (Fig. 2h) (Page 9, Lines 254-256, in red).

2. **Validation with Scaffold-Distinct Inhibitors**: In our original manuscript (Extended Data Fig. 6a,b), we utilized two additional scaffold-distinct MELK inhibitors, MELK-IN-1 and MELK-8a. Their in vitro IC50 values against MELK are 3 nM and 2 nM, respectively, which are higher than those of OTSSP167 (0.41 nM). Both MELK-IN-1 and MELK-8a suppressed the infections of SARS-CoV-2, HCoV-OC43, and HCoV-229E, with EC50 values correspondingly higher than that of OTSSP167 (results shown below panel b).

3. **Cellular Thermal Shift Assay (CETSA)**: We thank the Reviewer for the suggestion. We confirmed the direct target engagement of OTSSP167 with MELK in both Huh7 cells and human airway organoids via CETSA experiments. In CETSA, ligand binding to the target protein typically alters its thermal stability (stabilization or destabilization) (PMID: 18599640).The results revealed that OTSSP167 treatment caused a significant thermal destabilization of MELK compared to the vehicle control (results shown below panel c, d).This ligand-induced destabilization provided evidence of the direct interaction between OTSSP167 and MELK in the cellular environment. Interestingly, a recent study published in Nature (PMID: 41299171) revealed that the binding of certain inhibitors can induce the destabilization of target kinases, leading to their subsequent degradation. Consistent with this phenomenon, time-course analysis revealed that OTSSP167 treatment triggered MELK degradation in cells (results shown below panel e). We have added these new results to the revised manuscript (Extended Data Fig. 5e-g) (Page 10, Lines 275-283, in red).

a

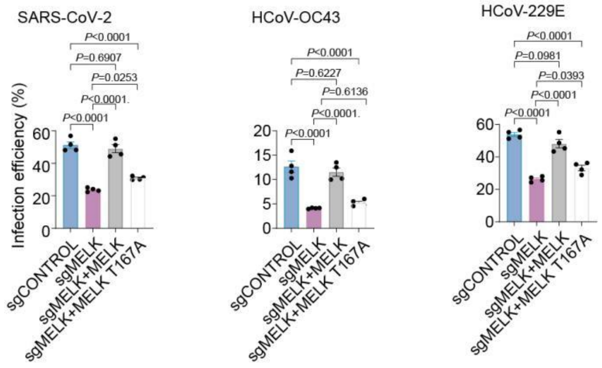

b

	OTSSP167 IC_{50} 0.41 nM	MELK-8a IC_{50} 2 nM	MELK-IN-1 IC_{50} 3 nM
SARS-CoV-2 EC_{50} (μM)	0.05	1.684	3.956
HCoV-OC43 EC_{50} (μM)	0.045	1.089	2.730
HCoV-229E EC_{50} (μM)	0.143	3.920	2.631

c

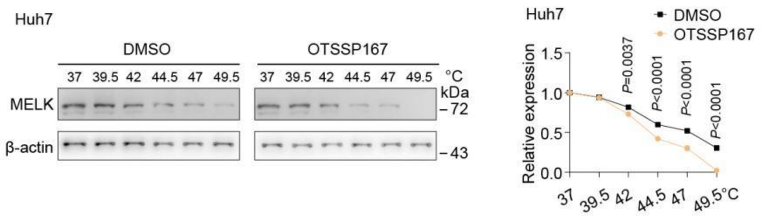

d

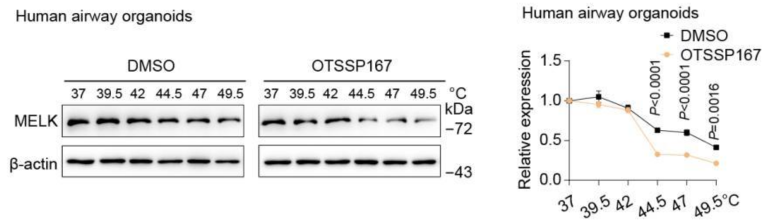

e

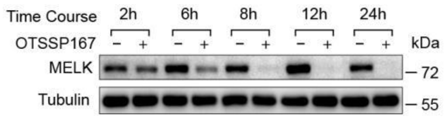

Figure legend:

a, Decreasing the expression of MELK blocked the infections of SARS-CoV-2, HCoV-OC43 and HCoV-229E. The expression of MELK was decreased with sgRNA contained lentivirus transduction. Rescue experiments were performed using both wild-type and kinase-dead(T167A) MELK. The infection rate was measured using IFA with N protein staining.

b, Comparison of the antiviral efficacy of different scaffold-distinct MELK inhibitors (OTSSP167, MELK-8a, and MELK-IN-1) against SARS-CoV-2, HCoV-OC43 and HCoV-229E, measured as half-maximal effective concentration (EC50, unit: μM).

c, d, Cellular thermal shift assay (CETSA) determined the target engagement of MELK by OTSSP167 in

(c) Huh7 cells and (d) human airway organoids. Cells were incubated with DMSO or OTSSP167 (0.2 uM) for 0.5 h, harvested, and subsequently subjected to heat treatment at the indicated temperatures for 3 min. Left: Soluble MELK protein levels were analyzed by Western blot. β-actin served as the loading control. Right: Melt curves displaying the quantification of MELK relative protein abundance plotted against temperature.

e, Time-course Western Blot analysis of MELK in Huh7 cells treated with OTSSP167 or DMSO. Tubulin served as the loading control.

Data presented in (a) are mean ± SEM of n = 4 independent biological replicates. Statistical significance was determined using one-way ANOVA followed by Dunnett's multiple comparisons test. Exact P values are indicated in the figure.

Data presented in (c) and (d) are mean ± SEM of n = 3 independent biological replicates. Statistical significance was determined using two-way ANOVA followed by Sidak's multiple comparisons test. Exact P values are indicated in the figure.

2. Cofilin-1 phosphorylation & antibody validation. The cofilin-1 phosphorylation narrative—especially the assignment of T70—is central but currently rests heavily on Western blot data with a newly generated pT70 antibody. This claim requires full antibody validation, including phospho-peptide competition blots and demonstration that the signal is lost in a T70A cellular context. Orthogonal mass spectrometry should complement the immunochemical evidence, with site-localized spectra and confidence metrics, and ideally an in-cell phosphoproteomic comparison under OTSSP167 treatment or MELK knockdown. In short, both the reagent characterization and the site assignment need to be tightened to match the strength of the mechanistic conclusion.

We thank the Reviewer for raising this critical question. Regarding the generation of the pT70 antibody, which was purified by cross-adsorbing against the non-phosphorylated peptide to remove non-specific binders. Enzyme-Linked Immunosorbent Assay (ELISA) validation subsequently confirmed that the purified antibody specifically recognizes the phosphorylated epitope. We fully agree with the Reviewer that further validation of both antibody specificity and the phosphorylation site would strengthen our conclusions. Following the Reviewer's suggestion, we have performed experiments to validate the Cofilin-1 phosphorylation and antibody specificity.

1. **Phospho-peptide competition**: Following the Reviewer's suggestion, we performed immunoblotting assays to confirm that the p-T70 antibody specifically recognizes the antigenic phosphorylated peptide (results shown below panel a). Furthermore, peptide competition assays confirmed that the antibody signal against both the phosphorylated peptide and the protein was specifically abolished by the phosphorylated peptide but not the non-phosphorylated counterpart, validating its high specificity for the phosphorylated epitope (results shown below panel b). We have added these

new results to the revised manuscript (Extended Data Fig. 8a-c) (Page 14, Lines 403-406, in red).

2. **T70A mutation validation**: To validate the site specificity in a cellular context, we transfected cells with plasmids expressing Flag-tagged wild-type (WT) or T70A mutant Cofilin-1. Subsequently, we enriched the Cofilin-1 proteins via immunoprecipitation (IP) using anti-Flag beads. The immunoblotting results indicated the pT70 signal was detected in WT Cofilin-1 but was lost in the T70A mutant (results shown below panel d**)**. We have added these new results to the revised manuscript (Extended Data Fig. 8 d) (Pages 14-15, Lines 406-411, in red).

3. **Phosphoproteomic validation**: We prepared three groups of cell samples for liquid chromatography–tandem mass spectrometry (LC-MS/MS) identification, including MOCK control, MELK overexpression, and MELK overexpression treated with OTSSP167. The results indicated that both unphosphorylated and phosphorylated peptide EILVGDVGQTVDDPYATFVK (Cofilin-1 residues 55-74) were identified. The site localization probability analysis assigned the phosphorylation to Threonine 70 (T70) with a high confidence score of 0.986. Based on the relative abundance revealed by extracted ion chromatograms (XIC), the T70 phosphorylation of Cofilin-1 was markedly elevated in the MELK-overexpressing group. Conversely, OTSSP167 treatment effectively suppressed this phosphorylation signal (results shown below panel e, f). We have added these new results to the revised manuscript (Extended Data Fig. 8e, f) (Page 15, Lines 411-417, in red).

In summary, we have rigorously validated both the specificity of our p-T70 antibody and the identity of the phosphorylation site through proteomic approaches, ensuring the solidity of our mechanistic conclusions.

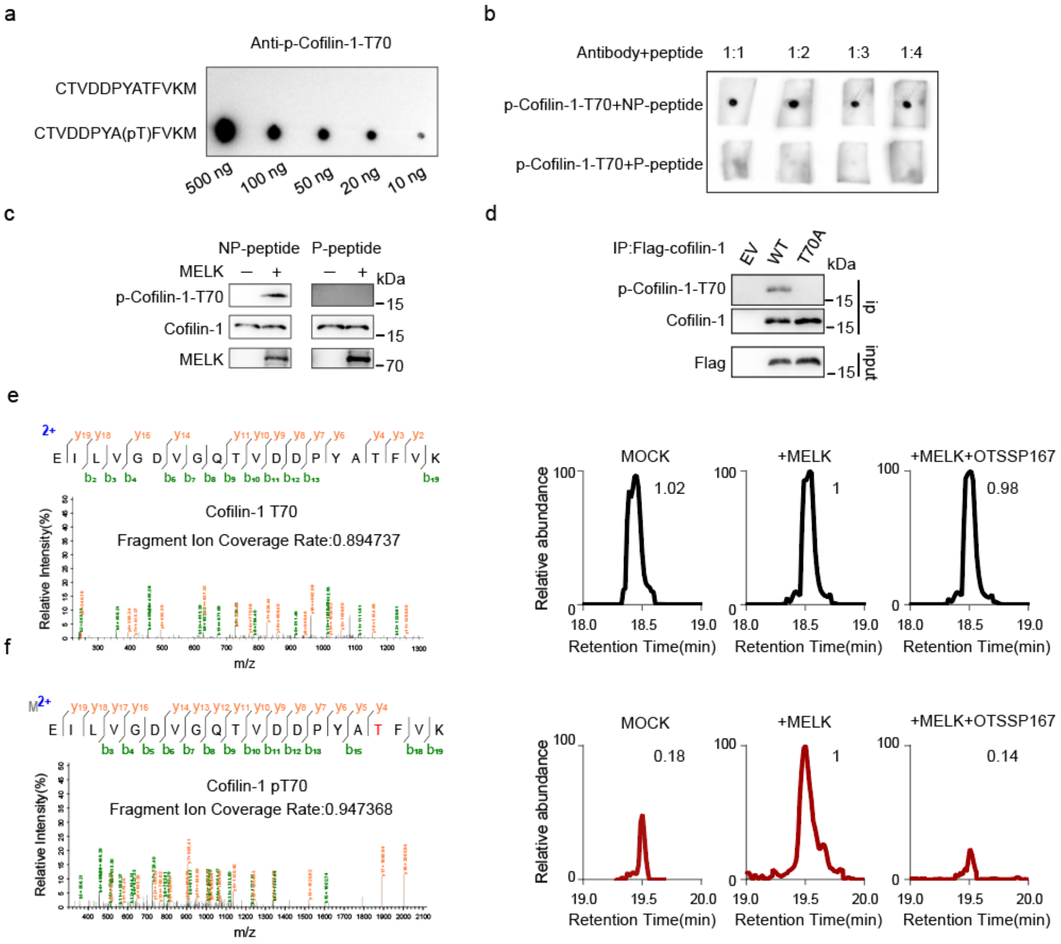

Figure legend:

a, Evaluation of antibody specificity using dot blot assay. Indicated amounts of non-phosphorylated (NP) or phosphorylated (P) peptides corresponding to the Cofilin-1 T70 site were spotted onto the membrane and immunoblotted with the generated anti-p-Cofilin-1-T70 antibody.

b,c, Peptide competition assay to validate the specificity of p-Cofilin-1-T70 antibody. b, The antibody was pre-incubated with phosphorylated Cofilin-1 T70 peptide (P-peptide) or non-phosphorylated Cofilin-1 T70 peptide (NP-peptide) at mass ratios of 1:1, 1:2, 1:3, and 1:4(antibody:peptide), then used to probe phosphorylated peptide spots. Each blot representing a separate membrane strip for the indicated condition. c, In vitro kinase assay validation. Recombinant Cofilin-1 was incubated with or without recombinant MELK in the presence of ATP. The reaction mixtures were immunoblotted with the antibody pre-incubated with NP- or P-peptides (1:1 mass ratio).

d, HEK293T cells were transfected with Flag-tagged wild-type (WT) or T70A mutant Cofilin-1 for 24 hours. Flag-Cofilin-1 protein was immunoprecipitated with anti-Flag beads and immunoblotted with the generated anti-p-Cofilin-1-T70 antibody.

e, f, Identification of Cofilin-1 T70 phosphorylation by LC-MS/MS analysis. HEK293T cells were transfected with MELK or empty vector for 24 h, followed by treatment with OTSSP167 (0.2 uM) or DMSO for 4 hours. Endogenous Cofilin-1 was enriched by immunoprecipitation using an anti-Cofilin-1 antibody and subjected to LC-MS/MS identification. Left panels: Annotated MS/MS spectra of the (e) non-phosphorylated and (f) T70-phosphorylated peptide (EILVGDVGQTVDDPYATFVK) identified from

the enriched samples. The phosphorylation site was localized to Threonine 70 with high confidence (probability score > 0.98). Right panels: Extracted ion chromatograms (XIC) displaying the relative abundance of the indicated peptides. Note that the pT70 signal (f) is markedly enriched by MELK overexpression but abolished by OTSSP167 treatment.

3. Phosphoproteomics data analysis QC. The DIA phosphoproteomics appears technically solid (7,593 proteins; 27,079 phosphopeptides), but the downstream inference of kinase activity needs added rigor. Either raise the site-localization cutoff to a more conservative threshold (≥0.9 for pivotal findings) or justify why 0.75 is sufficient in this context; report site-level FDR and replicate-wise statistics with appropriate multiple-testing correction; and complement KSEA with motif-centric or PTM-SEA analyses to mitigate annotation bias. For transparency and reuse, deposit the raw DDA/DIA files, spectral library, and Spectronaut outputs to PRIDE with complete metadata.

We thank the Reviewer for this constructive suggestion. We have comprehensively addressed the reviewer's concerns as follows:

1. **Site-localization cutoff**: We evaluated the distribution of localization probabilities based on the default Spectronaut export parameters (cutoff > 0.75, Class I sites; FDR < 0.01), and the results showed that the majority of the sites (90.6%) possess a localization probability greater than 0.9 (results shown below panel a). Following the Reviewer's suggestion, We re-analyzed the differentially phosphorylated sites and inferred kinase activity (KSEA, motif-centric and PTM-SEA) using only those sites with a localization probability > 0.9, FDR < 0.01. We have added the related results in the revised manuscript (Extended Data Fig. 1e, h).

2. **Statistics**: All analyses utilized the limma R package with empirical Bayes moderation, which incorporates all biological replicate measurements in linear models and stabilizes variance estimates across features. For changes in protein abundance and phosphorylated sites, p-values are adjusted using the Benjamini-Hochberg false discovery rate (FDR) method, with FDR < 0.05 as the significance threshold.

3. **Complementary kinase activity inference**: Following the Reviewer's suggestion, we performed both motif-centric (using PhosR, PMID: 33626354) and PTM-SEA (using ssGSEA2, PMID: 30563849) to complement kinase activity inference. Indeed, these orthogonal approaches successfully identified additional kinases that are commonly regulated across all three viral infections, including GRK6 (downregulated in motif-centric), DNAPK (upregulated in PTM-SEA), and CDK7 (downregulated in PTM-SEA). Notably, the downregulation of MELK activity in KSEA was largely consistent with these methods. Motif-centric analysis showed the significant downregulation in

HCoV-OC43 and HCoV-229E and a trend in SARS-CoV-2 (p=0.054). Consistently, PTM-SEA indicated that MELK activity was significantly downregulated in all three coronavirus). Additionally, the significant TBK1 activity upregulation was also detected in PTM-SEA (SARS-CoV-2, HCoV-OC43) and motif-centric (HCoV-OC43), partially corroborated the KSEA results (results shown below panel b, c). In contrast, the previously observed activation of TGFBR2 was no longer statistically significant when the analysis was restricted to phosphosites with a localization probability > 0.9. We have added these new results to the revised manuscript (Extended Data Fig. 2a, b) (Page 7, Lines 186-189, in red).

We regret that the PRIDE accession number, which was attached in the 'nr-reporting-summary' during the previous submission, was not accessible to the Reviewer. We have now re-submitted the PRIDE accessions, including raw DDA/DIA files, spectral library, and Spectronaut outputs to PRIDE. Global proteomics (Project accession: PXD057194, Token: fTZCUnjhIqio), global phosphoproteomics (Project accession: PXD057223,Token: pvDOiQfMjFMC), IP-MS (Project accession: PXD057095,Token: C8h3M1Lz9jA3), Cofilin-1 T70 phosphorylation (Project accession: PXD074164, Token: AjbSznUs6KfR).

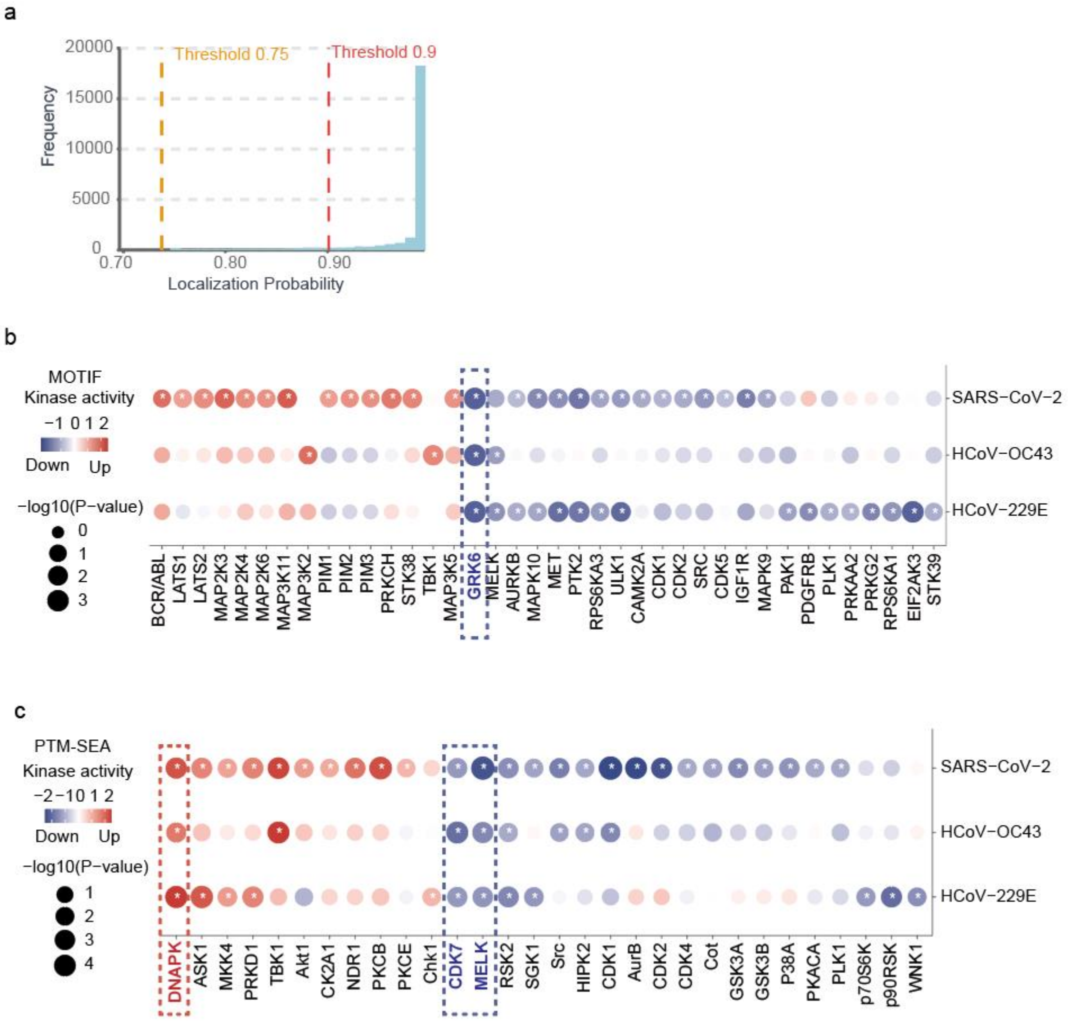

Figure legend:

a, Distribution of phosphosite localization probabilities. The dashed orange line indicates the default cutoff (0.75), and the dashed red line indicates the stricter cutoff (0.9) used for subsequent analyses. 90.6% of the identified sites exhibited a localization probability > 0.9.

b,c, Kinase activity inference across SARS-CoV-2, HCoV-OC43, and HCoV-229E infections using orthogonal approaches: (b) Motif-centric analysis performed with the PhosR package, and (c) PTM-SEA analysis performed with ssGSEA2. The color of the circles represents the normalized kinase activity (Red: upregulated; Blue: downregulated), and the size corresponds to the significance level (-log10 P-value). Dashed boxes highlight kinases with conserved regulatory patterns across the three coronaviruses, including the downregulation of MELK, GRK6, and CDK7, and the upregulation of DNAPK.

4. Cell-type & timing. Phosphoproteomics is only in Huh7 at a single 24h timepoint with mixed MOIs, which likely represents mid/late infection. A limited time-course (e.g., 1–6–24 h) and/or verification of MELK/cofilin sites in airway-relevant cells (Calu3/Calu-3, HBEC ALI, or your organoids) would help seperate entry vs post-entry signaling. The authors should compare or discuss

their data on the light of previously published SARS-CoV-2 phosphoproteomics articles

We sincerely thank the Reviewer for these constructive suggestions.We agree that the limitation regarding the respiratory physiological relevance of Huh7 cells.To exclude variations arising from different cellular backgrounds in phosphoproteomics, we aimed to identify a cell line susceptible to all three coronaviruses. However, currently available lung-derived cells do not simultaneously support the efficient replication of SARS-CoV-2, HCoV-229E, and HCoV-OC43.Therefore, we adopted the strategy from a pan-coronavirus genome-wide CRISPR screen (Wang et al., Cell 2021, PMID: 33333024) and selected Huh7 cells which are susceptible to all three coronaviruses. Additionally, considering the variability in replication kinetics and susceptibility, we utilized virus-specific MOIs as described in this study (Wang et al., Cell 2021 PMID: 33333024) to ensure comparable infection rates across the three coronaviruses. Furthermore, we validated the impact of MELK and its inhibition on viral replication using human airway organoids to ensure airway-relevance. Following the Reviewer's suggestions, we have performed experiments to examine the role of the MELK and Cofilin-1 in airway-relevant Calu-3 cells and comparative discussion with previously published SARS-CoV-2 phosphoproteomics articles.

1.Time-of-Addition Assay in Calu-3 Cells: In our original manuscript, we demonstrated that MELK knockdown inhibits viral infection in human airway organoids (Fig2, i-p of our original manuscript), and that MELK inhibition blocks viral entry in Huh7 cells (Fig7,a-e of our original manuscript). Consistent with our previous findings, a time-of-addition assay in Calu-3 cells confirmed that OTSSP167 exerts a significant inhibitory effect, particularly during the early phase of infection(results shown below panel a). We have added these new results to the revised manuscript (Extended Data Fig. 10a) (Page 17, Lines 471-475, in red).

1. Pseudovirus entry assays in Calu-3 cells revealed that viral entry was significantly impaired by OTSSP167 treatment, MELK knockdown, or the overexpression of Cofilin-1 mutants (S3A and T70A)(results shown below panel b-d). We have added these new results to the revised manuscript (Extended Data Fig. 10b, c, Extended Data Fig. 14c) (Page 17, Lines 480-484, Page 19, Lines 529-532, in red).

2. Discussion with published data: By employing three orthogonal kinase activity inference algorithms (KSEA, motif-centric, and PTM-SEA), we identified five conserved kinases (MELK, CDK7, GRK6, TBK1, and DNA-PK) that show consistent regulation across SARS-CoV-2, HCoV-OC43 and

HCoV-229E infections. Notably, our findings align well with previous reports. Our observation of MELK suppression is consistent with the global phosphorylation landscape of SARS-CoV-2 infection reported by Bouhaddou et al. (Cell, 2020; PMID: 32645325, Table S4), where MELK activity was downregulated at 8, 12, and 24 hours post-infection. Similarly, our finding regarding CDK7 is corroborated by Wu et al. (Cell, 2023; PMID: 36580912, Fig. S6A). Their dataset reveals a consistent trend of suppressed CDK7 activity at multiple time points (6, 24, 34, and 48 hours) following SARS-CoV-2 infection in human airway epithelial cells.We have added the discussion to the revised manuscript (Page 7, Lines 194-200, in red).

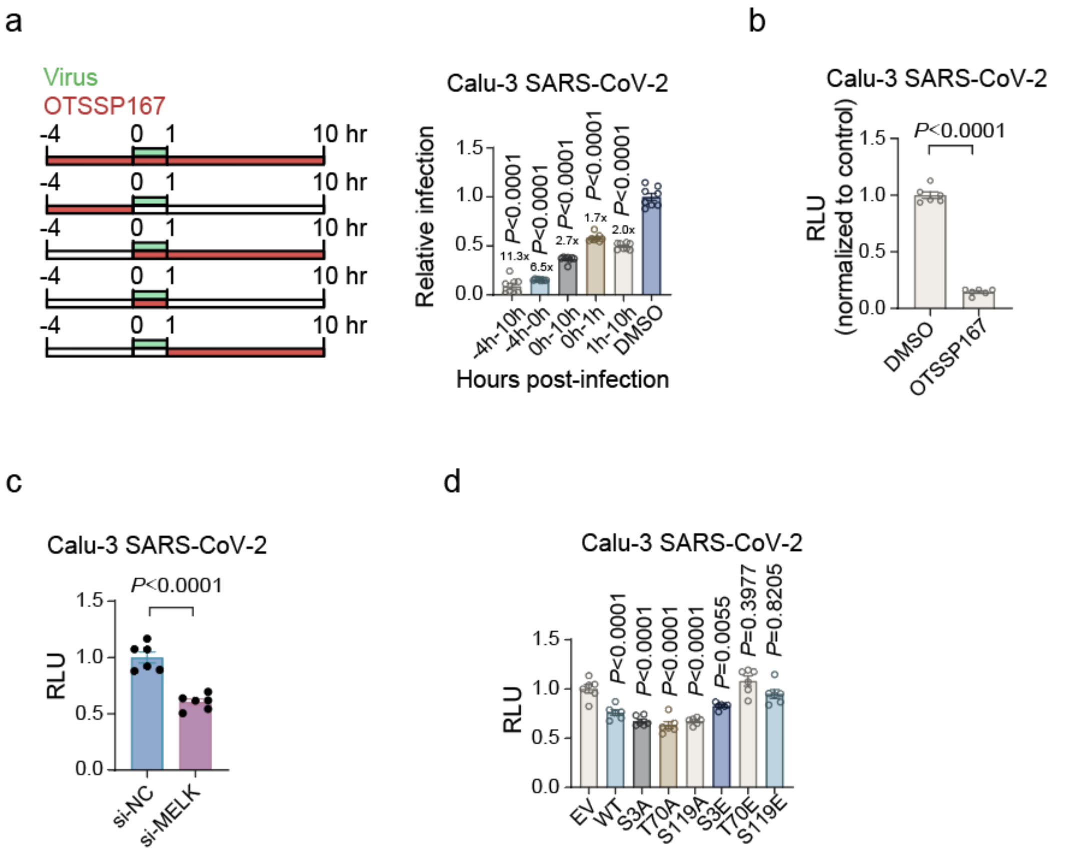

Figure legend:

a, Time-of-addition assay. Calu-3 cells were incubated with OTSSP167(0.2 μM) at the time points indicated. Infection efficiency at 24 hours post infection was quantified by immunostaining N protein and normalized to the DMSO-treated cells.

b, Quantification of viral entry efficiency in Calu-3 cells with or without OTSSP167 (0.2 μM) treatment inoculated with pseudoviruses bearing spike proteins from SARS-CoV-2. Viral entry efficiency was measured by normalized RLU.

c, Quantification of viral entry efficiency in siMELK-transfected Calu-3 cells inoculated with pseudoviruses bearing spike proteins from SARS-CoV-2. Viral entry efficiency was measured by normalized RLU.

d, Quantification of viral entry efficiency in cofilin-1 overexpression Calu-3 cells that were inoculated with pseudoviruses bearing spike proteins from SARS-CoV-2. Viral entry efficiency was measured by normalized RLU.

Data presented in (a) are mean ± SEM of n=8, and in (k) and (l) are mean ± SEM of n=6 independent biological replicates. Statistical significance was determined using one-way ANOVA followed by Dunnett's multiple comparisons test. Exact P values are indicated in the figure.

Data presented in (b) to (d) are mean ± SEM of n = 6 independent biological replicates. Statistical significance was determined using two-tailed Student's t-test. Exact P values are indicated in the figure.

5. Entry vs egress. Time-of-addition suggests both. The authors show reduced transferrin uptake, blocked fusion, fewer filopodia/microvilli, and less VLP release. Please quantify G/F-actin ratios, please analyse endocytic pit dynamics if feasible, and include sgMELK in transferrin/fusion assays to show phenotypes arise from MELK loss rather than just the compound.

We sincerely thank the Reviewer for this constructive suggestion. We followed the reviewer's suggestion and performed the following experiments.

1. **Quantification of G/F-actin ratios**: Using a biochemical fractionation assay of actin (PMID: 20181827), we quantified the G/F-actin ratios to assess the state of actin polymerization under OTSSP167 treatment. Consistent with our F-actin fluorescence imaging results, quantitative analysis of the fractionated lysates revealed that the F-actin pellet fraction was significantly reduced 4 hours after OTSSP167 treatment, then partial recovery after 12 hours (results shown below panel a, b). We have added these new results to the revised manuscript (Extended Data Fig. 7g, h) (Pages 12-13, Lines 347-352, in red).

2. **Endocytic pit dynamics**: We performed live-cell Total Internal Reflection Fluorescence (TIRF) Microscopy to track the lifetime distribution of GFP-clathrin-coated pits (CCPs), and we assessed cargo uptake efficiency using fluorescent transferrin.The lifecycle of a productive CCP, including initiation, maturation, and uncoating, is typically completed within 50–100 s (PMID: 19296720, PMID: 15339664). Consistent with this range, CCPs in our control cells exhibited a mean lifetime of 83.5 s. However, OTSSP167 caused an extension of the mean lifetime to 243.2 s, consistent with the formation of 'stalled' pits observed upon actin cytoskeleton inhibition (PMID: 21841790). Additionally, we used live-cell imaging to examine transferrin uptake dynamics with AF555-transferrin. The results revealed that both OTSSP167 and knockdown of MELK significantly impaired the uptake efficiency of transferrin, demonstrating a functional block in clathrin-mediated endocytosis (results shown below panel c-e). We have added these new results to the revised manuscript (Extended Data Fig. 12a-c) (Page 18, Lines 495-506, in red).

3. **Spike fusion assays in sgMELK cells**: Following the Reviewer's

suggestion, we examined the effect of MELK inhibition on Spike-mediated fusion in sgMELK Huh7 cells, as well as in Calu-3 cells transfected with siMELK or treated with OTSSP167. The results showed that, consistent with the OTSSP167 treatment, reducing MELK expression significantly inhibited Spike-mediated fusion (results shown below panel f-h). We have added these new results to the revised manuscript (Extended Data Fig.12d-f) (Page 18, Lines 506-510, in red).

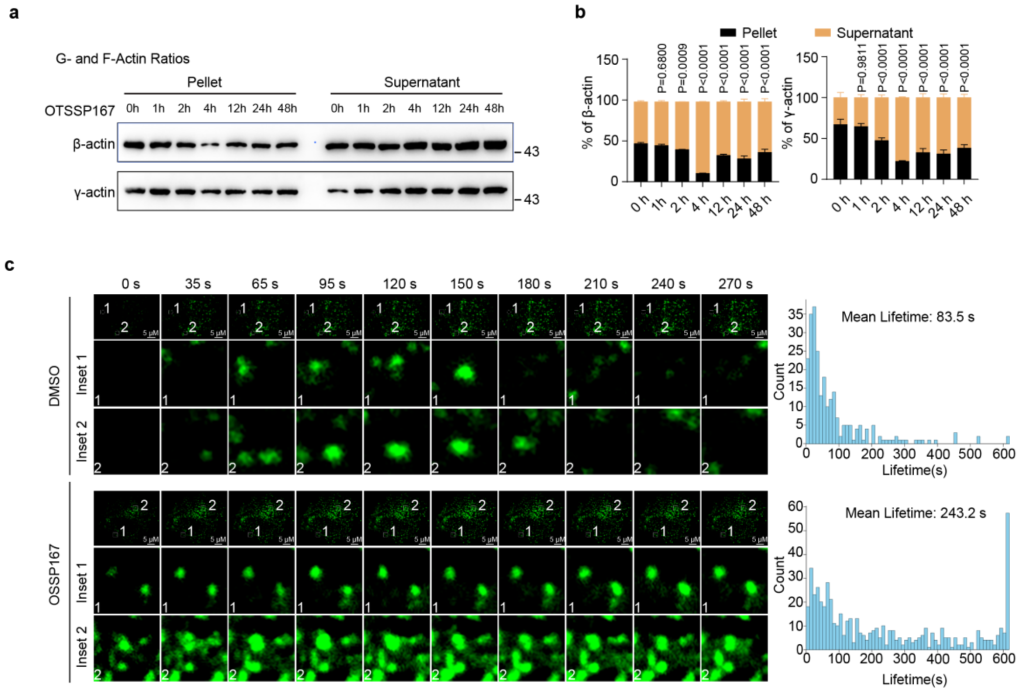

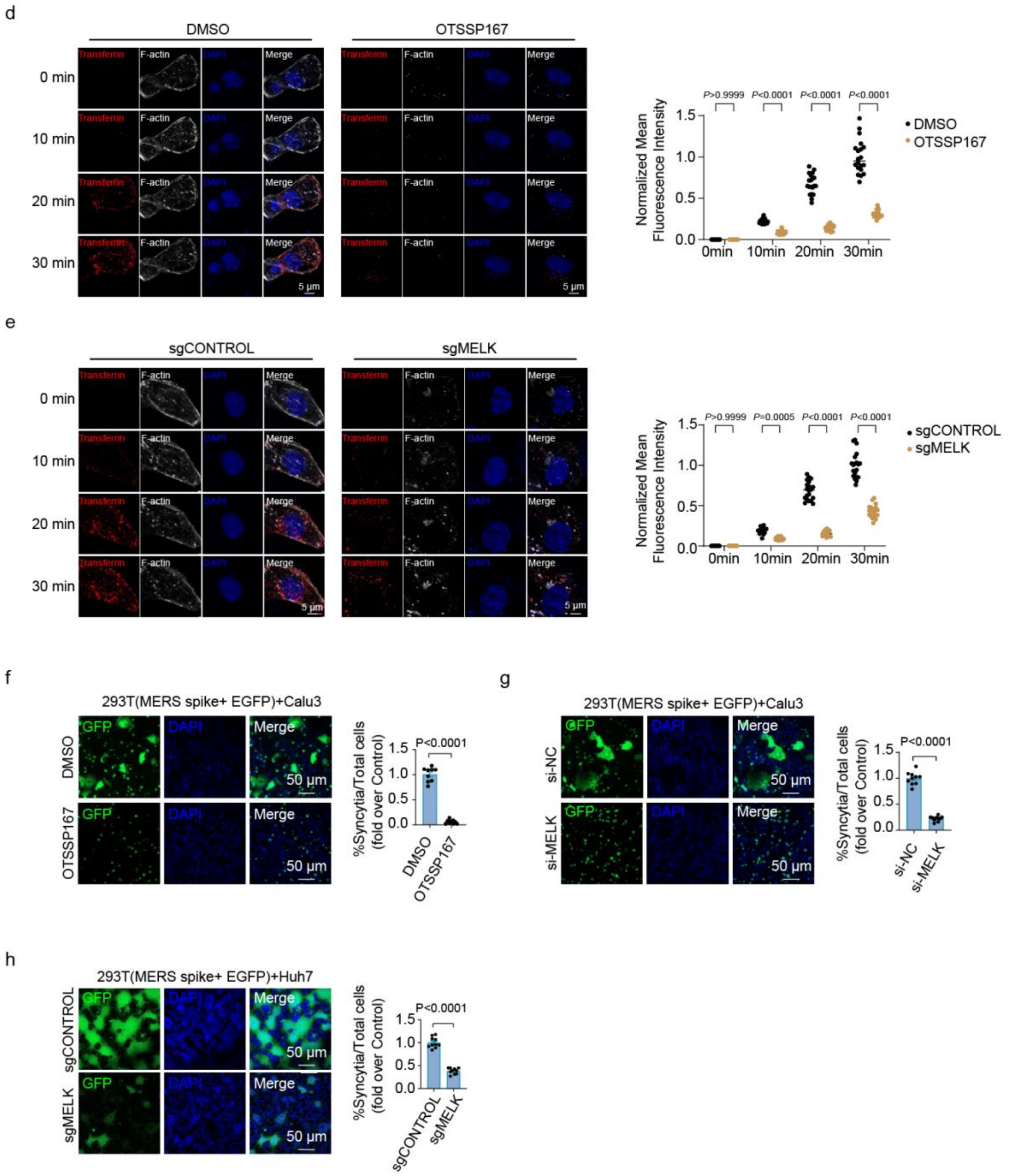

Figure legend:

a, Biochemical fractionation analysis of G-actin and F-actin ratios. Huh7 cells were treated with OTSSP167 for 0, 1, 2, 4, 12, 24, and 48 hours. F-actin (Pellet) and G-actin (Supernatant) fractions were separated by high-speed centrifugation (66000g, 1h), and the distribution of β-actin and γ-actin was detected by Western blot.

b, Quantitative analysis of the relative levels of β-actin and γ-actin with imageJ as shown in (a).

c, Analysis of clathrin-coated pit (CCP) dynamics using live-cell TIRF microscopy. Huh7 cells expressing GFP-Clathrin were treated with DMSO or OTSSP167(0.2 μM, 4 hours). Left: Time-lapse images of representative CCPs. Right: Histograms displaying the distribution of CCP lifetimes. The mean lifetime is indicated (83.5 s for DMSO vs 243.2 s for OTSSP167).

d, e, Live-cell imaging analysis of transferrin uptake dynamics. (d) Huh7 cells treated with OTSSP167(vs DMSO) or (e) transduced with sgMELK (vs sgCONTROL) were incubated with Transferrin-AF555. The dynamic accumulation of internalized transferrin was monitored by live-cell confocal microscopy over a

30-min time course. Transferrin (red), F-actin (Sir-actin, gray), Nucleus (Hoechst, blue) The fluorescence intensity of intracellular transferrin was quantified at indicated time points to evaluate the differences in uptake efficiency (right panels).

f–h, Spike-mediated membrane fusion assay. HEK293T cells were co-transfected with MERS-CoV S and pEGFP for 24 hours, then mixed with Calu-3 cells treated were co-cultured with target cells treated with (f) OTSSP167 (0.2 μM, 4 hours), (g) siMELK, or (h) mixed with sgMELK Huh7 cells. After 12 h, the formation of syncytium was observed using a confocal microscope. Syncytia formation (green) was quantified as the percentage of fused cells relative to control.

Data presented in (b) are mean ± SEM of n = 3 independent biological replicates. Statistical significance was determined using two-way ANOVA followed by Sidak's multiple comparisons test. Exact P values are indicated in the figure.

Data presented in (d) and (e) are mean ± SEM of n = 20 independent biological replicates. Statistical significance was determined using two-way ANOVA followed by Sidak's multiple comparisons test. Exact P values are indicated in the figure.

Data presented in (f) are mean ± SEM of n =9, in (g) and (h) are mean ± SEM of n =10 independent biological replicates. Statistical significance was determined using two-tailed Student’s t-test. Exact P values are indicated in the figure.

6. Cofilin epistasis in cells. In-vitro severing is convincing; mutants restrict pseudovirus entry. Test cellular epistasis: can cofilin-1 T70A or S3A (or double) rescue entry with MELK over-expression? Does a phospho-mimetic cofilin blunt OTSSP167’s antiviral effect? This would lock in the MELK→cofilin axis.

We thank the Reviewer for this constructive suggestion.Following the Reviewer's suggestion,we performed rescue experiments by co-expressing MELK with either wild-type (WT) Cofilin-1 or its mutants (S3A, T70A) and assessed viral entry using three pseudoviruses (SARS-CoV-2, HCoV-229E, and HCoV-OC43). The results demonstrated that the phosphodeficient mutants (S3A, T70A) effectively rescued the enhanced viral entry caused by MELK overexpression (results shown below panel a). While phospho-mimetic mutants are deficient in severing filament function, they lack the ability to promote filament assembly. Thus, introducing inactive phospho-mimetic mutants was insufficient to reverse the cytoskeletal disassembly and restore viral entry in the presence of OTSSP167 (results shown below panel b).

In summary, the observation that Cofilin-1 T70A or S3A mutants rescue viral entry in MELK-overexpressing cells provides evidence for MELK-Cofilin axis. We have added the new results to the revised manuscript (Extended Data Fig. 14d) (Page 19, Lines 533-537, in red).

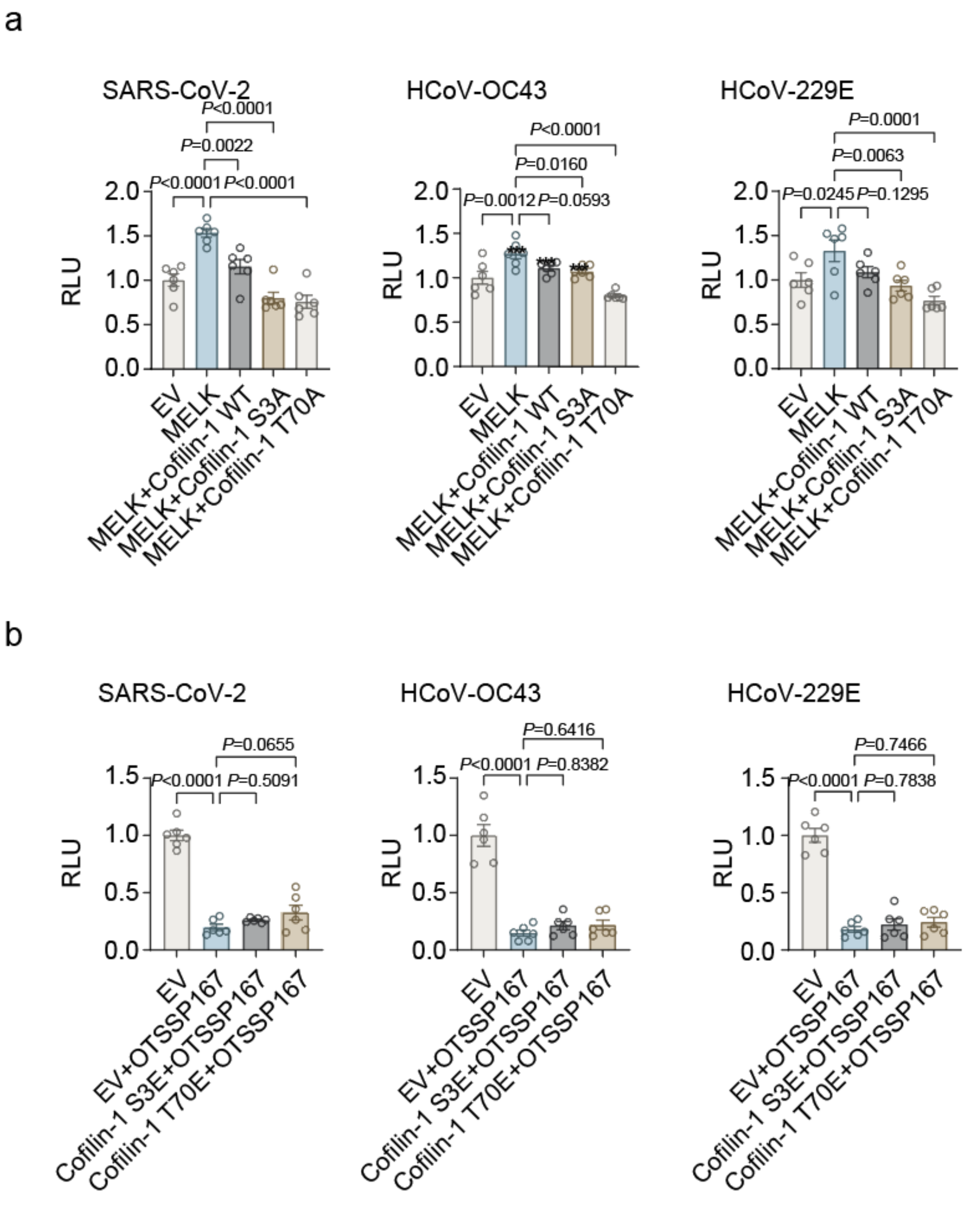

Figure legend:

a, Huh7 cells were co-transfected with MELK and Flag-tagged Cofilin-1 WT or phosphodeficient mutants (S3A, T70A). Cells were subsequently infected with pseudoviruses bearing spike proteins of SARS-CoV-2, HCoV-OC43, or HCoV-229E. Viral entry efficiency was measured by normalized RLU.

b, Huh7 cells expressing Cofilin-1 phosphomimetic mutants (S3E, T70E) were treated with OTSSP167 (0.2 μM) or DMSO, followed by infection with the indicated pseudoviruses. Viral entry efficiency was measured by normalized RLU.

Data presented in (a) means ± SEM of n = 3, and in (b) are mean ± SEM of n=6 independent biological replicates. Statistical significance was determined using one-way ANOVA followed by Dunnett's multiple comparisons test. Exact P values are indicated in the figure.

7. In vivo pharmacology. Mouse dosing is prophylactic (−1 dpi). Include a therapeutic arm (+12–24 hpi), basic tolerability/toxicity at 3.75 mg/kg intranasal in uninfected mice, and any PK/PD or target-engagement readout (even surrogate) to support translational relevance.

We thank the Reviewer for this constructive suggestion. We followed the reviewer's suggestion and examined therapeutic efficacy, tolerability and pharmacokinetics of OTSSP167 as follows.

1. **Therapeutic Efficacy**:To evaluate the therapeutic potential of OTSSP167, we initiated treatment at 12 or 24 hours post-infection (hpi).The results showed that post-infection treatment also significantly inhibited the replication of both SARS-CoV-2 BA.5 and HCoV-OC43 *in vivo* and improved survival rates to a certain extent. However, the therapeutic efficacy was less potent compared to prophylactic administration (results shown below panel a-f). This suggests that, similar to many anti-coronavirus agents (PMID: 31924756, PMID: 33561864), OTSSP167 exerts its optimal antiviral effects when administered prophylactically or early. We have added these new results to the revised manuscript (Extended Data Fig. 6 c-h) (Page 11, Lines 310-316, in red).

2. **Tolerability Assessment**:Following the Reviewer's suggestion, we treated uninfected mice with intranasal OTSSP167 (3.75 mg/kg) once daily for 5 days.No significant body weight loss or overt toxicity was observed (results shown below panel g, h). We have added these new results to the revised manuscript (Extended Data Fig. 6 a, b) (Page 11, Lines 293-294, in red).

3. **Pharmacokinetics (PK)**: To evaluate the translational potential, we further analyzed the pharmacokinetics of OTSSP167 in C57BL/6 mice serum following intranasal (i.n.) administration, using intravenous (i.v.) injection as a reference. The LC-MS analysis demonstrated that intranasal delivery resulted in excellent systemic absorption, with an absolute bioavailability of 97.0%. Notably, intranasal administration exhibited a sustained-release profile with a longer half-life (6.92 h) compared to the intravenous route (4.30 h) (results shown below panel i). These results demonstrated that intranasal administration of OTSSP167 effectively achieves robust and sustained systemic drug exposure. We have added these new results to the revised manuscript (Fig. 3m) (Page [11-12], Lines 318-325, in red).

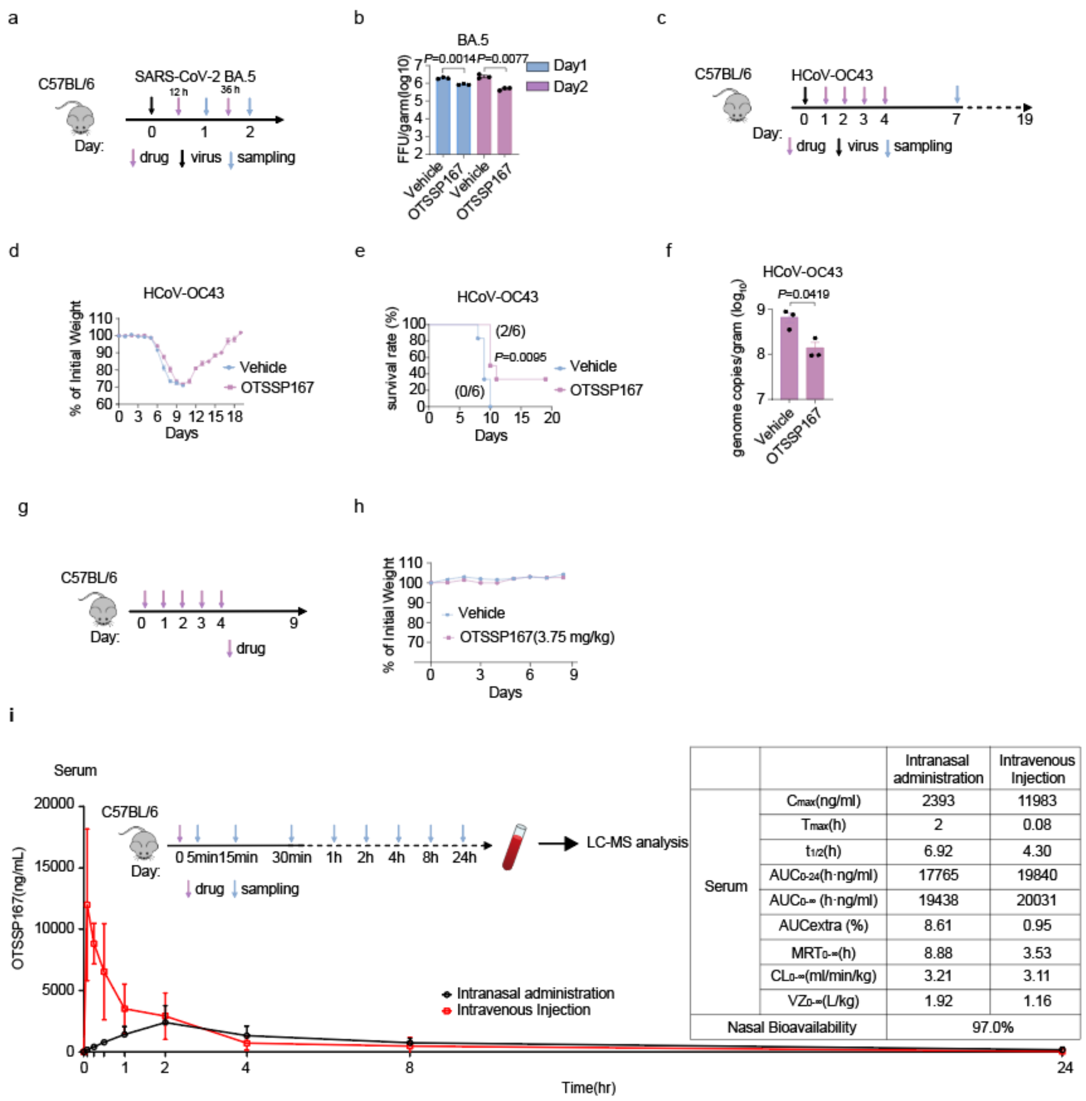

		Intranasal administration	Intravenous Injection
Serum	C_{max}(ng/ml)	2393	11983
	T_{max}(h)	2	0.08
	$t_{1/2}$(h)	6.92	4.30
	AUC_{0-24}(h·ng/ml)	17765	19840
	$AUC_{0-\infty}$ (h·ng/ml)	19438	20031
	AUCextra (%)	8.61	0.95
	$MRT_{0-\infty}$(h)	8.88	3.53
	$CL_{0-\infty}$(ml/min/kg)	3.21	3.11
	$VZ_{0-\infty}$(L/kg)	1.92	1.16
Nasal Bioavailability		97.0%	

Figure legend:

a,b, Six-week-old C57BL/6 mice were intranasally infected with 1×105 FFU SARS-CoV-2 BA.5 and treated with intranasal administration of DMSO or OTSSP167 (3.75 mg/kg/day) at 12 hpi and 36 hpi. Lungs of infected mice were collected on 1 dpi and 2 dpi. (a) Schematic of therapeutic administration in SARS-CoV-2 BA.5-infectedC57BL/6 mice. (b)The viral titers in lungs were determined by FFA.

c-f, Six-week-old C57BL/6 mice were intranasally infected with 2×104 FFU HCoV-OC43 and treated intranasally with DMSO or OTSSP167 (3.75 mg/kg/day) from 1 dpi for 4 days. Brains of infected mice were collected on 7 dpi. (c) Schematic of therapeutic administration in HCoV-OC43-infected C57BL/6 mice. (d) Body weight and (e) survival of the infected mice were monitored for 19 days. (f) Infected mice were sacrificed at 7 dpi, and viral gene copies in brain were quantified by qPCR.

g,h, Six-week-old C57BL/6 mice were used for an intranasal tolerability study of OTSSP167. OTSSP167 (3.75 mg/kg) was administered daily for 5 consecutive days. (g) Schematic of intranasal tolerability study in C57BL/6 mice. (h) Body weight of the mice was monitored for 8 days.

i, Pharmacokinetic profiles of OTSSP167 in mice. Serum concentration-time curves of OTSSP167 in C57BL/6 mice following intranasal (black line) or intravenous (red line) administration. Blood samples were collected at the indicated time points (5 min to 24 h) and analyzed by LC-MS. The table (right)

summarizes key pharmacokinetic parameters, maximum concentration (C_{max}), time to peak (T_{max}), elimination half-life ($t_{1/2}$), area under the curve (AUC_{0-24} and $AUC_{0-\infty}$), mean residence time ($MRT_{0-\infty}$), clearance ($CL_{0-\infty}$), and volume of distribution ($VZ_{0-\infty}$). The absolute bioavailability of intranasal administration was determined to be 97.0%. Data are presented as means ± SD.

Data presented in (b) are mean ± SEM of n = 3 independent biological replicates. Statistical significance was determined using two-tailed Student's t-test. Exact P values are indicated in the figure.

Data presented in (f) are mean ± SEM of n = 3 independent biological replicates. Statistical significance was determined using two-tailed Student's t-test. Exact P values are indicated in the figure.

Data presented in (i) are mean ± SD of n = 3 independent biological replicates.

8. Cell-cycle & innate immunity. MELK is cell-cycle regulated; both infection and OTSSP167 can shift cycling and secondarily impact actin. Control for cell-cycle distribution (PI/EdU/FUCCI) and discuss. Also check innate signaling (pTBK1, ISGs), as indirect antiviral effects are possible. It's important to rule out these confounders.

We thank the Reviewer for this thoughtful suggestion. Following the Reviewer's suggestion, we examined the time-course of cell cycle distribution and the innate immune response upon OTSSP167 treatment. Our data demonstrated that the antiviral activity of OTSSP167 precedes any significant cell cycle perturbation and occurs without triggering the innate immune response, indicating that its mechanism of action is independent of both cell cycle modulation and innate immunity.

1. **Cell Cycle**: We analyzed cell cycle distribution using PI staining. The results showed that OTSSP167 had no significant effect on the cell cycle at 6 h or 12 h post-treatment. Consistent with previous study (PMID: 33520717), G2/M arrest was observed after 24 h of treatment. Coronaviruses typically complete a replication cycle within 12 h. Our time-of-addition and pseudovirus entry assays demonstrated potent inhibition at the early entry stage, suggesting that the antiviral effect of OTSSP167 occurs before the cell cycle perturbation (results shown below panel a, b). We have added these new results to the revised manuscript (Extended Data Fig. 13 a, b) (Pages 18-19, Lines 518-524, in red).

2. **Innate Immunity**: We also examined the effect of OTSSP167 on innate immunity, including p-TBK1 levels and the transcription of IFNβ and ISG56. The results showed that OTSSP167 had no significant impact on these markers, indicating that its antiviral activity is independent of the innate immune signaling response (results shown below panel c-e). We have added these new results to the revised manuscript (Extended Data Fig. 13 c-e) (Page 19, Lines 524-528, in red).

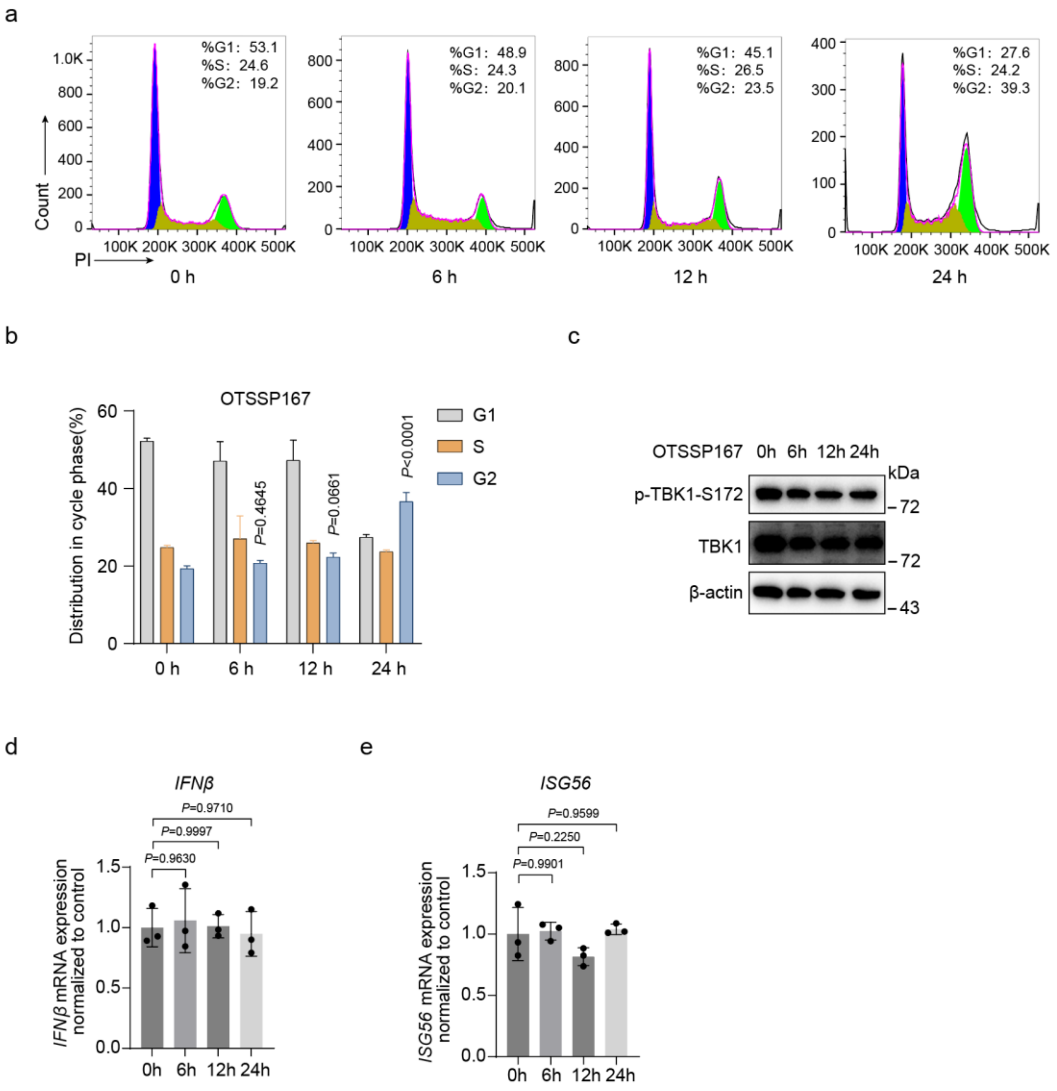

Figure legend:

a, Representative flow cytometry plots show cell cycle distribution (proportions of G1, S, and G2/M phases) at different time points (0, 6, 12, 24 h) following OTSSP167(0.2 μM) treatment for indicated time, as analyzed by PI staining.

b, Quantification of cell cycle distribution in (a).

c, Western blot of phosphorylated TBK1 (p-TBK1-S172) and total TBK1 protein levels at different time points following OTSSP167 treatment in Huh7 cells. β-actin served as the loading control.

d,e, (d) IFNβ and (e) ISG56 mRNA expression levels at different time points following OTSSP167 treatment in Huh7 cells, measured by qPCR and normalized to the 0 h control. Reference gene: GAPDH. Data presented in (b), (d) and (e) are mean ± SEM of n = 3 independent biological replicates. Statistical significance was determined using one-way ANOVA followed by Dunnett's multiple comparisons test. Exact P values are indicated in the figure.

9. Receptor-level controls. Flow analysis shows ACE2/hAPN/DPP4 unchanged and spike–ACE2 co-IP intact under drug. Please add gating strategies, replicate counts, and stats; test fusion/entry in TMPRSS2-high Calu-3 to broaden relevance.

We thank the Reviewer for this thoughtful suggestion. We have added the gating strategies, and performed statistical analysis of the mean fluorescence intensity (MFI) from four independent biological replicates (n=4) (results shown below panel a-f). Consistent with the results obtained in Huh7 cells, inhibition of MELK activity and expression in Calu-3 cells significantly suppressed spike-mediated cell fusion and pseudovirus entry (results shown in Panels b, c of Comment #4 of Reviewer #2, and Panels f, g of Comment #5 of Reviewer #2). We have added these new results to the revised manuscript (Extended Data Fig. 11a,b,d) (Page 17, Lines 487-489, in red).

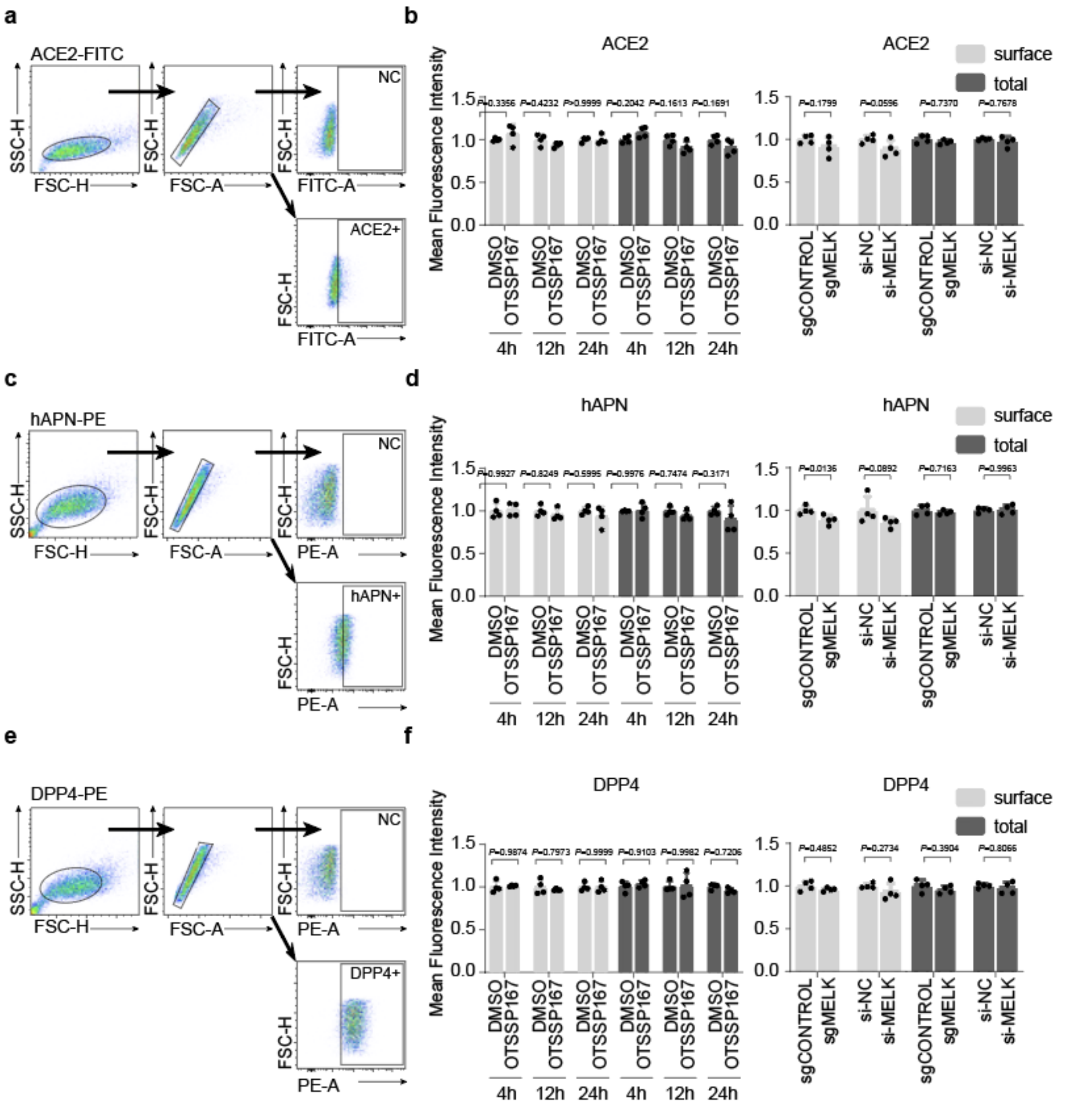

Figure legend:

a,c,e, Representative flow cytometry gating strategies. Single-cell populations were gated sequentially by forward scatter-height vs side scatter-height (FSC-H vs SSC-H) and forward scatter-height vs forward scatter-area (FSC-H vs FSC-A), followed by discrimination of negative (NC) and positive (ACE2+, hAPN+, DPP4+) populations for ACE2 (a), hAPN (c), and DPP4 (e).

b,d,f, Quantification of receptor expression. Mean fluorescence intensity (MFI) of surface and total ACE2 (b), hAPN (d), and DPP4 (f) at different time points (4, 12, 24 h) with OTSSP167 treated or in si/sgMELK transduced Huh7 Cells.

Data presented in (b) 、(d) and (f) are mean ± SEM of n = 4 independent biological replicates. Statistical significance was determined using two-way ANOVA followed by Sidak's multiple comparisons test. Exact P values are indicated in the figure.

10. Quantification & stats. For PLA counts, filament metrics, syncytia, etc., indicate blinding, normalise to cell area, and prefer ANOVA + post-hoc over

multiple t-tests. Report n, exact p, effect sizes, and define biological vs technical replicates clearly.

We thank the Reviewer for these crucial suggestions to enhance the rigor and reproducibility of our data. We have thoroughly checked the quantification and statistical analysis methods as follows.

1. **Quantification Standards**: For PLA signals, actin filament metrics, and syncytia formation, data were analyzed in a blinded manner to minimize bias, and values were normalized to cell area (or cell number) to ensure accuracy.

2. **Statistical Methods**: We have used One-way or Two-way ANOVA followed by appropriate post-hoc tests (e.g.,Tukey's or Sidak's) for multi-group comparisons.

We have updated the Figure Legends and Methods section to clearly define biological replicates (n),and exact p-values and effect sizes where applicable.

Minor comments

- AA residue naming. "Tyrosine 70 (T70)" should be threonine 70.

We apologize for this oversight and thank the Reviewer for the careful reading. We have corrected these mistakes in the revised manuscript (line 391, in red).

- MOI/timepoint heterogeneity. Please justify per-virus MOIs (e.g., 1.0 vs 0.1) and single 24h phospho timepoint; discuss impact on KSEA comparability.

We thank the Reviewer for this insightful comment. Considering the variability in replication kinetics and susceptibility among these coronaviruses, using a uniform MOI would result insignificant variations in the infection rates and viral loads of these viruses at 24 hours. We referred to a pan-coronavirus genome-wide CRISPR screen study (Wang et al., Cell 2021,PMID: 33333024, SARS-CoV-2 MOI=0.05-0.1, HCoV-229E MOI=0.1, HCoV-OC43 MOI=3, in Huh7.5.1 cells), and experimentally optimized the MOIs to normalize their infection levels at 24 h. It avoided the impact on KSEA comparability caused by the huge differences in infection rates among different viruses. Furthermore, the 24-hour timepoint encompasses viral entry, replication, and assembly. This allows for the identification of conserved kinase activities that are commonly regulated across multiple stages of the viral life cycle.

-EC50/CC50/SI. Provide selectivity indices across viruses and cell types, a compact table would do.

We thank the Reviewer for this suggestion. Accordingly, we have calculated and summarized the EC50, CC50, and Selectivity Indices (SI) across all tested viruses and cell types. These data are now presented in a compact table (Table S4).

- pT70 antibody. Add immunogen sequence/epitope context and validation panels to Supplement.

We thank the Reviewer for this suggestion. We have now included the detailed immunogen sequence (C-TVDDPYA(pT)FVKM) and the antibody generation protocol in the Methods section. Furthermore, we have added the antibody validation to Supplement (Extended Data Fig. 8a-d) (Pages 14-15, Lines 403-411, in red).

-Microscopy methods. State acquisition settings, randomisation/blinding, and segmentation parameters (e.g., arivis Vision 4D) to ensure reproducibility.

We thank the Reviewer for this suggestion.We have updated the Microscopy Methods section to include acquisition settings of LSM980 and Elyra 7, the randomisation/blinding, and segmentation parameters of arivis Vision 4D (Pages 31-32, Lines 883-906, in red).

-Figure typos. Clean minor labels (e.g., “OTSS167” missing “P”; repeated “BA.5”).

We apologize for these oversights.We have corrected these mistakes in the revised manuscript (lines 245, 513, in red).

-VLP interpretation. Also show cell-associated S/E/M/N to exclude expression/assembly artifacts; quantify intra- vs extracellular.

Following the Reviewer's advice,we examined S, E, M, and N protein levels in both whole cell lysates and supernatants. The results demonstrated that OTSSP167 treatment significantly reduced expression of S, E, M, and N proteins in the extracellular VLP fraction，but had no effect on their intracellular expression (results shown below panel).
We have added these new results to the revised manuscript (Extended Data Fig. 15e) (Page 89, Lines 1785-1791, in red).

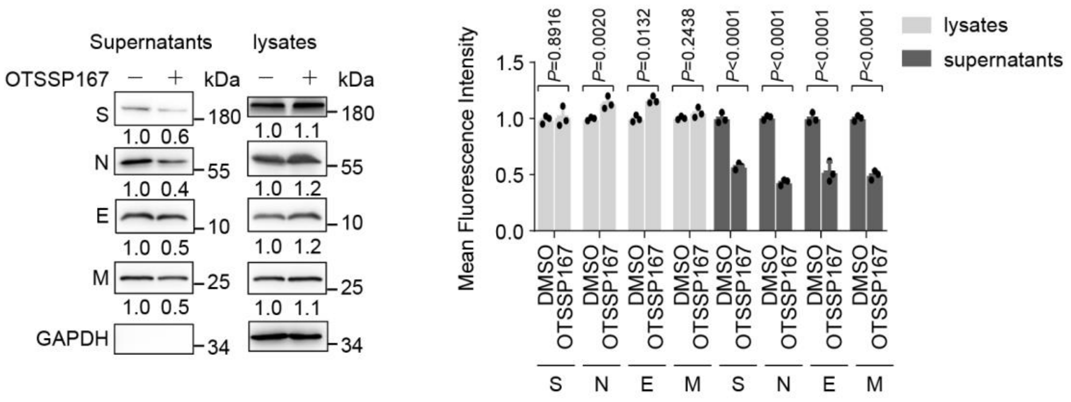

Figure legend:

Effects of OTSSP167 on intracellular and extracellular assembly of VLPs. Left, Western blot of S, N, E, and M protein levels in cell supernatants and lysates after DMSO (-) or OTSSP167 (+) treatment. GAPDH served as the loading control. The gray values were quantified using ImageJ and normalized to the DMSO group (set to 1.0). Right, Quantification of fluorescence intensity from the Western blot bands, comparing intracellular (lysates, gray bars) and extracellular (supernatants, dark gray bars) S, N, E, and M protein levels.

Data presented in right are mean ± SEM of n = 3 independent biological replicates. Statistical significance was determined using two-way ANOVA followed by Sidak's multiple comparisons test. Exact P values are indicated in the figure.

-Organoid health. Include viability/TEER or ciliation metrics under OTSSP167 (reduced microvilli may reflect general epithelium stress).

We thank the Reviewer for this suggestion. To assess organoid health, we measured intracellular ATP levels using the CellTiter-Glo assay. The results showed no significant difference in cell viability following OTSSP167 treatment. Furthermore, Scanning Electron Microscopy (SEM) images revealed that OTSSP167 treatment specifically reduced the number of microvilli without affecting the abundance of cilia, indicating its specific cytoskeletal disruption (results shown below panel a, b).

We have added these new results to the revised manuscript (Extended Data Fig. 4i, Extended Data Fig. 15d) (Page 10, Lines 265-268, Page 19, Lines 546-550, in red).

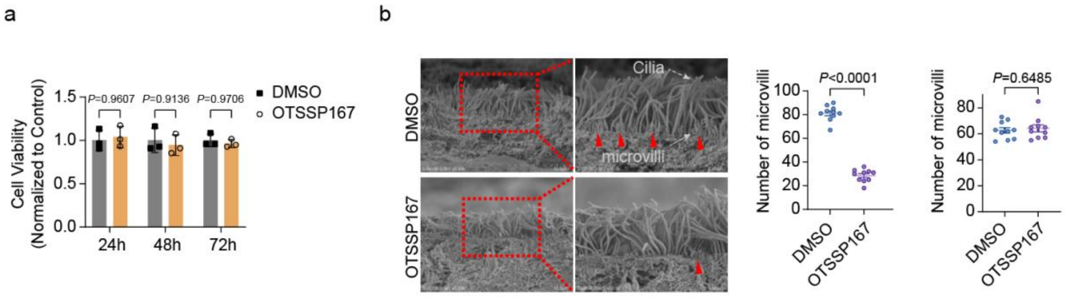

Figure legend:

a, Effect of OTSSP167 on the viability of human proximal airway organoids. Organoids were treated with OTSSP167 (0.2 μM) or DMSO for the indicated times. Intracellular ATP levels were measured using the CellTiter-Glo assay for cell viability evaluation. Values are normalized to the control.

b,Representative scanning electron microscopy (SEM) images of human proximal airway organoids (left) show the abundance of microvilli (red arrowheads) and motile cilia (white arrows). Quantification (right) reveals that OTSSP167 treatment significantly specifically reduces the number of microvilli, has no significant impact on the density of cilia.

Data presented in (a) are mean ± SEM of n = 3 independent biological replicates. Statistical significance was determined using two-way ANOVA followed by Sidak's multiple comparisons test. Exact P values are indicated in the figure.

Data presented in (b) are mean ± SD of n=10 from three independent biological replicates. Statistical significance was determined using two-tailed Student's t-test. Exact P values are indicated in the figure.

-Data & code. Please provide PRIDE accession(s) and share analysis outputs/code, include uncropped blots and imaging source data as per journal policy.

We thank the Reviewer for this important reminder. We regret that the PRIDE accession number, which was attached in the 'nr-reporting-summary' during the previous submission, was not accessible to the Reviewer. Following the Reviewer’s suggestion and journal policy, we have now re-submitted the PRIDE accessions (Global proteomics (Project accession: PXD057194, Token: fTZCUnjhIqio), global phosphoproteomics (Project accession: PXD057223,Token: pvDOiQfMjFMC), IP-MS (Project accession: PXD057095,Token: C8h3M1Lz9jA3), Cofilin-1 T70 phosphorylation (Project accession: PXD074164, Token: AjbSznUs6KfR)). We also updated the Methods section with the relevant analysis outputs and provided the full Source Data file (Page 33, Lines 948-955, in red).

Reviewer #3 (Remarks to the Author):

Summary:
The manuscript begins with a large-scale kinase inhibitor screen to identify compounds that perturb coronavirus infection, which showed that the MELK inhibitor OTSSP167 inhibits infection by the coronaviruses SARS-CoV-2, OC43, and 229E. The study also performs a complimentary phosphoproteomic analysis of Huh7 cells infected by SARS-CoV-2, OC43, or 229E, quantifying protein abundance and phosphoproteomic alterations following infection. By applying KSEA analysis to the phosphoproteomic data, they determine that MELK activity is reduced upon infection by all three coronaviruses. MELK small molecule inhibition, knockdown, and knockout are utilized to demonstrate broad requirement for MELK function for efficient replication of coronaviruses in cell culture, notably performing these experiments in both cancer-derived cells and primary human proximal airway organoids. Pretreatment with MELK inhibitor also reduces infection of mice with SARS-CoV-2 or OC43. The authors apply immunofluorescence microscopy to show co-localization of MELK with actin fibers, utilize AP-MS to identify an MELK interaction with cofilin, and demonstrate that MELK phosphorylates cofilin S3, T70 and S119. They demonstrate that cofilin T70A binds actin more strongly, and has augmented actin depolymerization activity. MELK inhibition appears to impact both viral entry and later stages of the virus lifecycle.

This study validates that MELK is required for optimal virus replication by multiple coronaviruses, including both low and high pathogenicity coronaviruses, in cell culture and in-vivo. It also establishes biochemical details of MELK-mediated cofilin phosphorylation, and studies the impact of MELK inhibition on the cytoskeleton. I predict these findings will have a moderate impact on the fields of virology and cell biology, and will likely stimulate interest in testing MELK inhibitors on other viruses.

We sincerely appreciate the Reviewer’s thoughtful summary and and constructive comments. We have followed the Reviewer’s suggestions, performed more experiments and revisions, carefully addressed the Reviewer’s concerns and prepared a point-by-point response as follows.

My enthusiasm is moderated by some issues with the manuscript:
1) The manuscript has numerous spelling and grammatical errors, and some of the supplemental data figures are not properly labeled.
2) The literature review in the introduction is insufficient.
3) Reporting of the proteomics data is insufficient, please see notes below.
4) The choice of using hepatocyte cells for drug screening and proteomic studies needs to be addressed in the text, since the liver is not the primary site of infection.
5) The materials and methods section is incomplete.

We sincerely appreciate the Reviewer's time and valuable comments. We apologize for the grammatical errors, as well as the insufficient literature review and method descriptions in the original manuscript. To address the Reviewer' s concerns, we have performed a comprehensive revision of the manuscript. The major updates are summarized below, with detailed point-by-point responses following thereafter:

1. **Manuscript Optimization**: We have thoroughly proofread the text to correct grammatical errors, expanded literature review to include more related phosphoproteomic studies, and supplemented missing methodological details (e.g., lentivirus and immunoprecipitation protocols).

2. **Model for Screening**: We clarified the rationale for using Huh7 cells for initial screening, citing the lack of available lung-derived cell lines that simultaneously support the efficient infection of all three coronaviruses (SARS-CoV-2, HCoV-OC43 and HCoV-229E). Following reported strategies pan-coronaviruses screening (Wang et al., Cell 2021, PMID: 33333024), we selected Huh7 cells and, importantly, validated our core findings in physiologically relevant human airway cells and organoids.

3. **Proteomics Data Reporting**: We have clarified the reporting of our proteomics data, including the identification depth, localization score cutoffs, and residue annotations. Furthermore, we have now re-submitted the PRIDE accessions, including all raw DDA/DIA files, spectral library, and Spectroaut outputs. We regret that the PRIDE accession number, which was attached in the 'nr-reporting-summary' during the previous submission, was not accessible to the Reviewer.

Comments on text:
-The introduction includes insufficient literature review regarding other phosphoproteomic analyses studying coronaviruses. Please add citations and a synopsis of other mass spectrometry-based global phosphoproteomic analyses on coronavirus infection published in peer reviewed journals.
101: "Currently the role of kinases in human coronavirus infections remains

largely unknown”. This seems overstated, please revise to be more precise.
145: “These findings unveil a previously undiscovered mechanism of virus-host interaction, potentially providing new avenues for antiviral strategy development.” Please revise to be more precise.

We thank the Reviewer for these constructive comments.We have revised the manuscript as follows:

1. **Literature Review**: We have revised the Introduction to include a more comprehensive synopsis of mass spectrometry-based phosphoproteomic analyses. Specifically, in addition to the previously cited Bouhaddou et al. (2020, Cell, PMID: 32645325) we added citations and summaries for studies by Klann et al. (2020,Molecular Cell, PMID: 32877642, ref12),Wu et al. (2023,Cell, PMID: 36580912, ref13) , and Stukalov et al. (2021, Nature, PMID: 33845483, ref14) (Pages 4-5, Lines 111-118, in red).

2. **Line 101**: We have revised the sentence to be more precise: “Currently, host kinases that play conserved regulatory roles across diverse human coronaviruses remain largely unidentified” (Page 4, Lines 97-99, in red) .

3. **Line 145**: We have revised the sentence to be more precise: “These findings unveil a common regulation of coronavirus infections by MELK through the modulation of actin cytoskeleton.Targeting MELK potentially provides new avenues for antiviral strategy development” (Page 5-6, Lines 144-147, in red).

-The study’s initial drug screening and proteomics datasets utilize Huh7 cells. Since the primary site of infection of these coronaviruses is the lungs, the authors should more clearly articulate why they chose to use Huh7 cultured hepatocytes for these initial studies.

We thank the Reviewer for this thoughtful comment, which is related to Comment #4 of Reviewer #2. To exclude variations arising from different cellular backgrounds in phosphoproteomics, we aimed to identify a cell line susceptible to all three coronaviruses. However, currently available lung-derived cells do not simultaneously support the efficient replication of SARS-CoV-2, HCoV-229E and HCoV-OC43. Therefore, we adopted the strategy from a pan-coronavirus genome-wide CRISPR screen (Wang et al., Cell 2021, PMID: 33333024) and selected Huh7 cells which are susceptible to all three coronaviruses. Subsequently, we validated the function of MELK and MELK inhibitors in regulating coronavirus infection in lung-derived Calu-3 cells and human airway organoids. We have also included this clarification in the revised manuscript (Page 6, Lines 159-164, in red).

-I have concerns regarding reporting of the proteomics data. The proteomics studies were only performed in duplicate – this is not a deal-breaker but is regrettable, since it limits the statistical power of the analysis. Furthermore, the number of phosphopeptides reported is quite high for a timsTofPro system (>27,000), and the materials and methods do not list the localization score cutoff specified in Spectronaut – the localization score cutoff should be specified, and if no filter is currently implemented, the authors should re-analyze the data and report the number of localized phosphosites. The annotations in the supplemental data file also includes residues such as M, Q, and N which are not canonically phosphorylated – clarity regarding this data is needed. In addition, I see no mention of upload of the raw data to a repository. It is convention in the proteomics community to upload raw data to a repository such as PRIDE, as this is required for proper review and consideration of proteomics data for publication. For the revision, the mass spectrometry data should be uploaded to a repository, and reviewer credentials should be shared for evaluation. Upon acceptance of the manuscript, my understanding of Nature policy is that this raw data should be made available for public access.

We thank the Reviewer for this thoughtful comment.

1. **Identification Depth**: We thank the Reviewer for noting the high coverage of our dataset. This identification depth was achieved by the DIA-PASEF on the timsTOF Pro combined with a comprehensive spectral library strategy. The reported depth is well within the capabilities of this technology (PMID: 35944843). To avoid confusion, we have clarified the DIA-PASEF details in the Materials and Methods section (Page 26, Lines 734-735, in red).

2. **Localization score** cutoff: In the original manuscript (Line 665-666), we stated that phosphorylation sites were exported using the default Spectronaut parameters (localization score cutoff > 0.75 and FDR < 0.01). Following the Reviewer's suggestion, we evaluated the distribution of localization probabilities based on these default parameters, and the results showed that the vast majority of the sites (90.6%) already possess a localization probability greater than 0.9 (results shown below).

3. **Clarification on other modifications**: We apologize for the confusion regarding the residues M, Q, and N. These annotations were generated by the default export settings of Spectronaut to denote Oxidation (M) and Deamidation (N/Q) modifications on the peptides, alongside phosphorylation. To avoid confusion, we have removed these non-phosphorylated modification annotations from the revised dataset. Furthermore, in accordance with Reviewer #2's suggestion (Comment #3), we applied a stringent localization cutoff (>0.9) to the dataset. Due to the removal of these non-phosphorylated modification annotations (which eliminates redundancy in site counting) and

the implementation of the stricter localization probability cutoff (>0.9), the final dataset was refined to 21,761 high-confidence phosphorylation sites. We have updated Supplementary Table S1.

Mass spectrometry raw data: We regret that the PRIDE accession number, which was attached in the 'nr-reporting-summary' during the previous submission, was not accessible to the Reviewer. Furthermore, we have now re-submitted the PRIDE accessions, including all raw DDA/DIA files, spectral library, and Spectroaut outputs. Global proteomics (Project accession: PXD057194, Token: fTZCUnjhIqio), global phosphoproteomics (Project accession: PXD057223,Token: pvDOiQfMjFMC), IP-MS (Project accession: PXD057095,Token: C8h3M1Lz9jA3), Cofilin-1 T70 phosphorylation (Project accession: PXD074164, Token: AjbSznUs6KfR).

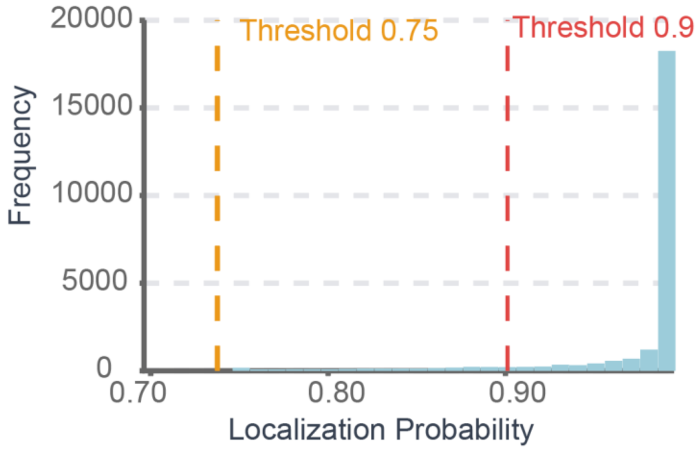

Figure legend:
Distribution of phosphosite localization probabilities. The dashed orange line indicates the default cutoff (0.75), and the dashed red line indicates the stricter cutoff (0.9) used for subsequent analyses. 90.6% of the identified sites exhibited a localization probability > 0.9.

-The authors should address in the text why the inhibition phenotype of MELK knockdown / knockout is frequently significantly smaller in magnitude than that of OTSSP167 treatment. Also, I was unable to find details of the knockout lentivirus in the methods section, this information needs to be included.

We thank the Reviewer for this insightful suggestion. The stronger phenotype observed with OTSSP167 treatment compared to MELK KD/KO is likely due to compensatory effects and delivery efficiency. OTSSP167 inhibits kinase activity upon binding and acts very rapidly (within minutes to tens of minutes). In contrast, siRNA usually needs 48-72 hours to take effect, and the continuous inhibition of MELK expression by CRISPR/Cas9 is prone to inducing cellular compensatory mechanisms. Additionally, the efficiency of

small molecules entering cells is typically higher than that of siRNA transfection. We have added this explanation to the revised manuscript (Page 22, Lines 612-618, in red).
We apologize for the oversight regarding the methods. The sequences of the two sgRNAs targeting human MELK were listed in Supplementary Table S3 of the original submission. We have now included full details of the knockout lentivirus in the Methods section (Page 28, Lines 784-793, in red).

-For the mouse work, I'm curious if the authors also attempted to treat with OTSSP167 after exposure of the animal to virus (this experiment is not necessary for publication).

We thank the Reviewer for raising this thoughtful point, which is related to Comment #7 of Reviewer #2. To evaluate the therapeutic potential of OTSSP167, we initiated treatment at 12 or 24 hours post-infection (hpi).The results showed that post-infection treatment also significantly inhibited the replication of both SARS-CoV-2 BA.5 and HCoV-OC43 *in vivo* and improved survival rates to a certain extent. However, the therapeutic efficacy was less potent compared to prophylactic administration (results shown below panel a-f). This suggests that, similar to many anti-coronavirus agents (PMID: 31924756, PMID: 33561864), OTSSP167 exerts its optimal antiviral effects when administered prophylactically or early. We have added these new results to the revised manuscript (Extended Data Fig. 6c-h) (Page 11, Lines 310-316, in red).

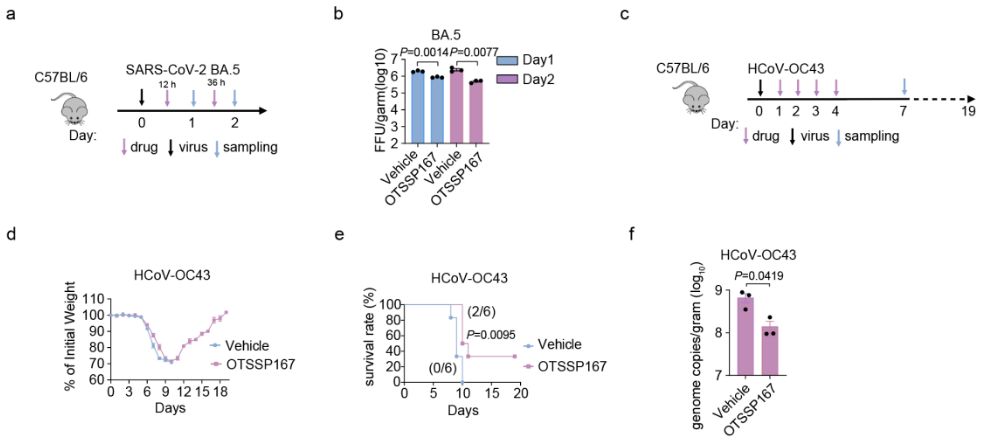

Figure legend:

a,b, Six-week-old C57BL/6 mice were intranasally infected with 1×10^5 FFU SARS-CoV-2 BA.5 and treated with intranasal administration of DMSO or OTSSP167 (3.75 mg/kg/day) at 12 hpi and 36 hpi. Lungs of infected mice were collected on 1 dpi and 2 dpi. (a) Schematic of therapeutic administration in SARS-CoV-2 BA.5-infectedC57BL/6 mice. (b)The viral titers in lungs were determined by FFA.

c-f, Six-week-old C57BL/6 mice were intranasally infected with 2×104 FFU HCoV-OC43 and treated

intranasally with DMSO or OTSSP167 (3.75 mg/kg/day) from 1 dpi for 4 days. Brains of infected mice were collected on 7 dpi. (c) Schematic of therapeutic administration in HCoV-OC43-infected C57BL/6 mice. (d) Body weight and (e) survival of the infected mice were monitored for 19 days. (f) Infected mice were sacrificed at 7 dpi, and viral gene copies in brain were quantified by qPCR.

g,h, Six-week-old C57BL/6 mice were used for an intranasal tolerability study of OTSSP167. OTSSP167 (3.75 mg/kg) was administered daily for 5 consecutive days. (g) Schematic of intranasal tolerability study in C57BL/6 mice. (h) Body weight of the mice was monitored for 8 days.

Data presented in (b) and (f) are mean ± SEM of n = 3 independent biological replicates. Statistical significance was determined using two-tailed Student's t-test. Exact P values are indicated in the figure.

Data presented in (e) are survival curves of n = 6 mice per group. Statistical significance was determined using Log-rank (Mantel-Cox) test. Exact P values are indicated in the figure.

- It would be helpful to address the apparent dichotomy that (1) reduced MELK activity is associated with coronavirus infection, yet (2) MELK inhibition suppresses viral replication. Addressing this point would help the broader readership reconcile these observations. For example, MELK regulation during infection may be localization-specific, or residual MELK function during infection might remain sufficient to support replication.

We appreciate the Reviewer for the accurate understanding and insightful comment. Indeed, our results showed that viral infection induces a reduction in MELK activity, and this reduction suppresses viral replication. Interestingly, we also found that infections upregulate TBK1, a canonical innate immune kinase that protects host cells from viral invasion by triggering the expression of interferons (IFNs) and interferon-stimulated genes (ISGs). In the original manuscript (Lines 534-536), we discussed this phenomenon. Similar to the TBK1 response, we propose that the regulation of MELK during infection represents a host defense strategy to resist viral invasion. Therefore, inhibition of MELK mimics and amplifies this defense mechanism. We hope this explanation clearly addresses the Reviewer's concerns, and we have updated the Discussion section to articulate this concept more clearly (Page 21, Lines 585-590, in red).

-Numerous grammatical and spelling errors were seen throughout the manuscript. The manuscript needs to be proof-read before resubmission. Also please add page numbers to the manuscript.

We sincerely apologize for the grammatical and spelling errors in the original manuscript. We have carefully revised and proof-read our manuscript, and have added page numbers as suggested.

Specific comments on figures:
-Figure 1G includes cytotoxicity data for OTSSP167, but other figures depicting similar experiments omit viability data. Cytotoxicity data should be included for small molecule inhibitor titration experiments on cultured cells.

We thank the Reviewer for this important suggestion.Since some experiments employed the same cell lines and drug concentrations as Figure 1G, we omitted viability data in subsequent figures. To avoid confusion and provide a comprehensive overview, we have summarized the EC50 and CC50 (viability data) values across all tested viruses and cell types in a compact table (Table S4). Additionally, following the Reviewer's suggestion, we measured the cell viability of both cultured cells and organoids at the specific time points corresponding to the inhibitor titration assays(results shown below panel a-d). The results showed that OTSSP167 had no significant effect on cell viability. We have added these new data to the revised manuscript (revised Extended Data Fig. 4f-i) (page 10, lines 267-268 , in red).

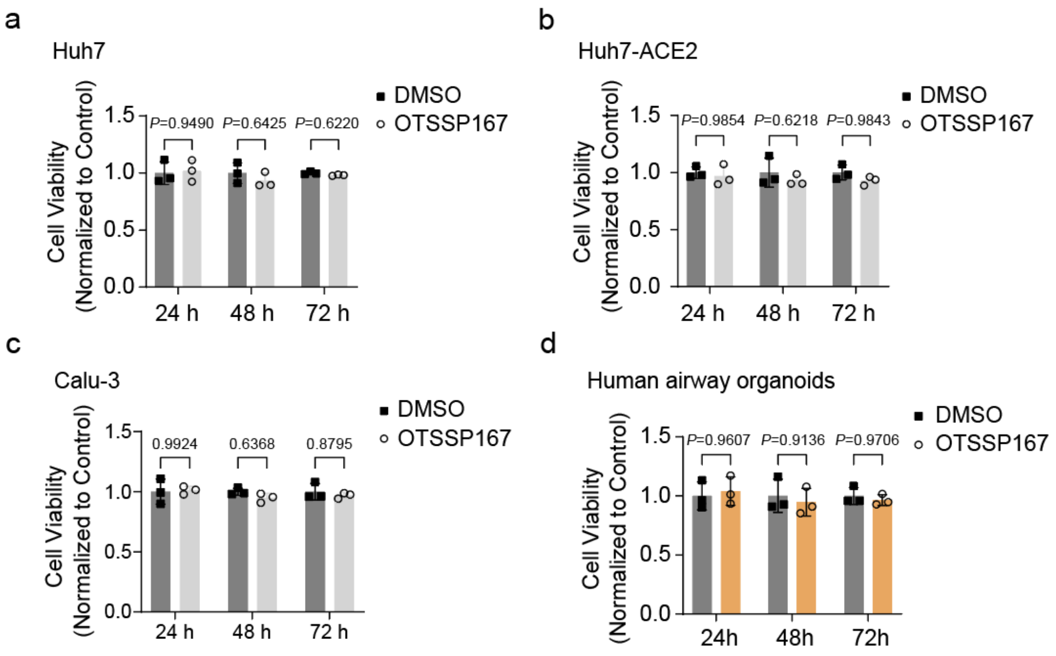

Figure legend:

a–d,Cell viability assays were performed to assess the potential cytotoxicity of OTSSP167 in (a) Huh7, (b) Huh7-ACE2, (c) Calu-3 cells, and (d) human airway organoids. Cells were treated with OTSSP167 or DMSO for the indicated time points (24, 48, and 72 h). Viability was quantified using the CCK-8 assay for cell lines (a–c) and the CellTiter-Glo ATP assay for organoids (d). Results are normalized to the DMSO-treated control at each time point.

Data presented in (a)to (d) are mean ± SEM of n = 3 independent biological replicates. Statistical significance was determined using two-way ANOVA followed by Sidak's multiple comparisons test. Exact P values are indicated in the figure.

-Figure 6b: When looking at a printout, it is difficult to tell the difference between the colors of each line in this plot.

We thank the Reviewer for this helpful comment. We have adjusted the colors to maximize contrast and distinguishability. We have updated the new data in the revised manuscript (revised Fig 6b, Extended Data Fig. 9a-d).

-Figure 7h includes a Strep-S immunoprecipitation, but I cannot find details of this construct or IP protocol in the methods section. I'd like to understand how the Strep-S fusion construct is designed, and how the IP experiment was performed.

We appreciate the Reviewer for noting this oversight. We have updated the comprehensive description of the immunoprecipitation protocol in the methods section (Pages 28-29, Lines 807-820, in red).

-Figure 7k and 7l - I would expect to see an OTSSP167-only treatment included in these experiments.

We thank the Reviewer for this thoughtful suggestion. We have performed the experiment including the OTSSP167-only treatment group. Given that OTSSP167 inhibits both the endocytosis and membrane fusion pathways, it demonstrated a stronger suppression of viral entry than the individual use of Chlorpromazine (CPZ) or Camostat Mesylate (CM). Notably, since OTSSP167 alone already suppresses viral entry to extremely low levels,the co-treatment with CPZ or CM showed no further additive effect. We have updated the new data in the revised manuscript (revised Fig 7k, l) (page 18, lines 514-515, in red).

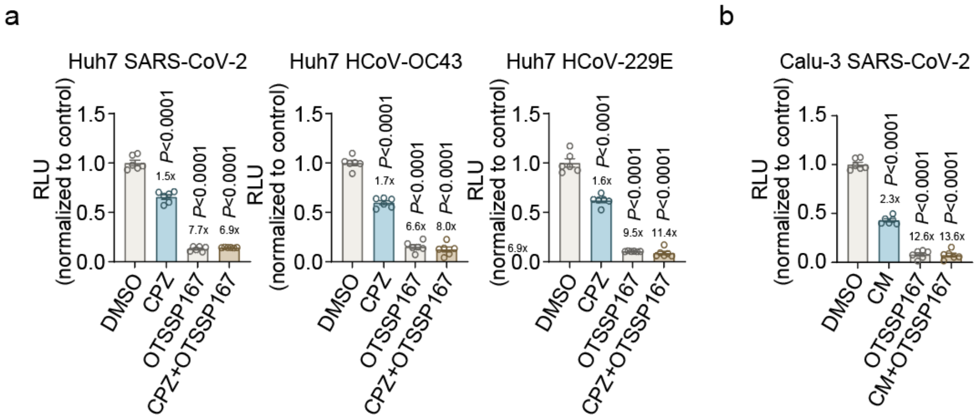

Figure legend:

a, Huh7 cells were pre-treated with the endocytosis inhibitor chlorpromazine (CPZ, 10 μM), OTSSP167 (0.2 μM), or their combination.

b, Calu-3 cells were pre-treated with the TMPRSS2 inhibitor camostat mesylate (CM, 25 μM),

OTSSP167 (0.2 μM), or their combination.

Cells were subsequently infected with the indicated pseudoviruses (SARS-CoV-2, HCoV-OC43, or HCoV-229E). Viral entry efficiency was quantified by luciferase activity (RLU) and normalized to the DMSO control.

Data presented in (a) and (b) are mean ± SEM of n=6 independent biological replicates. Statistical significance was determined using one-way ANOVA followed by Dunnett's multiple comparisons test. Exact P values are indicated in the figure.

Extended data figure 2c and 2d: The heat map colorbars require quantitative labels. “High” and “Low” are not appropriate labels.

We thank the Reviewer for pointing out this oversight. We have updated the labels with a quantitative scale representing the Log_2(Fold Changes) (revised Extended Data Fig. 1g, h).

-Extended data figure 11: Please correct the legend.

We apologize for this oversight. We have corrected these mistakes in the revised manuscript (page 87, line 1749, in red).

-Extended data figure 12e: Legend indicates that the inhibition of virus egress caused by OTSSP167 treatment was quantified, but it’s unclear where this quantification data is located.

We thank the Reviewer for pointing this out and apologize for the missing data. We have updated the revised Figure to include the quantification graphs from the Western blot analysis (supernatant and cellular fractions) with ImageJ (revised Extended Data Fig. 15e).

REVIEWERS' COMMENTS

Reviewer #2 (Remarks to the Author):

This revised manuscript integrates global phosphoproteomics across three human coronaviruses (SARS-CoV-2, HCoV-229E, HCoV-OC43) with high-content kinase inhibitor screening (1,805 compounds) to identify MELK as a common host kinase required for coronavirus infections. The authors demonstrate that the MELK inhibitor OTSSP167 exhibits broad-spectrum antiviral activity (EC50 values 0.045–0.143 μM) in cell lines, human airway organoids, and two mouse infection models. Mechanistically, they show MELK directly phosphorylates cofilin-1 at S3 and T70, suppressing its actin-severing activity; inhibition of MELK therefore activates cofilin-1, disrupts F-actin dynamics, and impairs both clathrin-mediated endocytosis and spike-mediated membrane fusion required for viral entry.
The authors have performed a substantial body of additional experiments in revision. The rebuttal is thorough and responsive, addressing all major concerns raised by both reviewers. The manuscript is considerably strengthened and represents an important contribution linking host kinase signaling, actin cytoskeleton regulation, and broad-spectrum antiviral biology.

Remaining concerns

The authors have addressed the majority of the original reviewers' concerns satisfactorily. However, several points merit further attention or should be explicitly acknowledged as limitations.
1. OTSSP167 selectivity and off-target considerations
While the authors have substantially strengthened the case that MELK is the relevant target (kinase-dead rescue, multiple inhibitors, CETSA), OTSSP167 is known to be a multi-kinase inhibitor with significant activity against FLT3, DYRK1A, Aurora B, and other kinases. The scaffold-distinct inhibitors MELK-8a and MELK-IN-1 are welcome additions, but their EC50 values against viral infection are notably higher than OTSSP167 (the data in the rebuttal suggests a meaningful gap). This differential potency could reflect either pharmacological differences or off-target contributions to OTSSP167's antiviral efficacy. The authors should explicitly discuss this discrepancy and acknowledge OTSSP167's known polypharmacology in the Discussion. They might also consider presenting MELK-8a and MELK-IN-1 EC50 curves side-by-side with OTSSP167 for direct comparison.

We thank the Reviewer for this constructive suggestion.
1. Discussion Updates: We have expanded the Discussion section to acknowledge the known polypharmacology of OTSSP167 and explicitly discussed the discrepancy in antiviral potency between OTSSP167 and the

other scaffold-distinct MELK inhibitors (Page 23, Lines 639-648, in red).
2. Figure Updates: Following the Reviewer’s suggestion, we have updated the figures to display the dose-response (EC50) curves of MELK-8a and MELK-IN-1 side-by-side with those of OTSSP167 (Supplementary Fig. 5a, b).

2. Phosphoproteomics limited to a single cell type and timepoint
The phosphoproteomics discovery was performed exclusively in Huh7 cells at 24 h post-infection. While the authors appropriately validated key functional findings in Calu-3 cells and airway organoids (as suggested by Reviewer #2), the kinase activity inference driving the entire study rests on a hepatocellular carcinoma cell line at a single late timepoint. The authors contextualize this well by referencing concordant MELK suppression in Bouhaddou et al. (Vero E6) and Wu et al. (HNE cells). Nonetheless, the absence of phosphoproteomic validation in a respiratory epithelial cell line remains a limitation that should be explicitly stated in the revised Discussion. Even showing that MELK phosphorylation status (e.g., p-MELK Western blot) changes upon infection in Calu-3 would help bridge this gap.

We thank the Reviewer for this insightful comment. Because of the requirement for simultaneous susceptibility to all three viruses and the lack of specific p-MELK antibodies, we are currently unable to conduct validation in a respiratory epithelial cell line. Following the Reviewer's suggestion, we have explicitly stated this limitation in the revised Discussion (Page 21, Line 610-612, in red).

3. In vivo dosing regimen and translational framing
The animal experiments are encouraging but employ a prophylactic dosing regimen (first dose at -1 dpi). For a paper presenting a potential therapeutic avenue, the absence of a therapeutic (post-infection) dosing arm is notable. Additionally, intranasal delivery of a kinase inhibitor is non-standard for clinical translation. Given that OTSSP167 has entered clinical trials for oncology via systemic administration, the authors should discuss whether systemic dosing could achieve therapeutic lung concentrations and what the expected toxicity profile might be at antiviral-effective doses. The current Discussion mentions clinical trials but could be more explicit about the translational gap.

We thank the Reviewer for this thoughtful suggestion.

1. Therapeutic Efficacy: In our previous revision, we evaluated a therapeutic dosing regimen (Extended Data Fig. 6c-h). While optimally effective when administered prophylactically, OTSSP167 treatment initiated at 12 or 24 hours post-infection also significantly inhibited the in vivo replication of SARS-CoV-2 and HCoV-OC43.

2. Translational framing: OTSSP167 and other clinical-stage kinase inhibitors were primarily developed for cancer therapies via systemic administration. However, since human coronaviruses primarily target the nose, airways, and lungs in vivo, intranasal delivery allows the drug to act directly on viral target cells, thereby minimizing potential side effects. Indeed, inhalation is already a clinically utilized route for several respiratory antivirals. Furthermore, our pharmacokinetic data demonstrate that intranasal delivery of OTSSP167 in mice achieves a high absolute bioavailability of 97.0%. Therefore, we postulate that systemic dosing would require significantly higher overall doses to reach effective antiviral concentrations in the lungs, thereby potentially increasing the risk of adverse effects. Addressing the translational gap requires optimizing precise delivery to target cells to boost efficacy while limiting adverse effects. We have explicitly discussed these translational considerations in the revised Discussion section (Page 22-23, Lines 620-645, in red).

4. Cofilin-1 T70 phosphorylation stoichiometry and functional contribution
The pT70 antibody validation is now thorough (dot blot, peptide competition, T70A mutant, LC-MS/MS). However, the relative functional contribution of T70 versus S3 phosphorylation to the overall antiviral phenotype is not clearly resolved. Both S3A and T70A cofilin-1 mutants show enhanced antiviral activity, but the magnitudes appear similar. Quantitative comparison (e.g., double mutant S3A/T70A versus each single mutant) would clarify whether these sites act additively or redundantly. Additionally, T70 was not detected in the global phosphoproteomics of infected cells (only in the overexpression LC-MS/MS experiments), which raises the question of its stoichiometry under physiological conditions. This should be discussed.

We thank the Reviewer for this insightful suggestion.

1. Functional Contribution: In our previous revision (Extended Data Fig. 14b, c), we utilized transient transfection to express WT and mutant Cofilin-1. Due to the limitations of transfection efficiency, the background viral infection in the untransfected cells likely partially masked the phenotypic differences between the T70A and S3A mutants. Importantly, our direct in vitro severing assay (Fig. 6b) reveals that the T70A mutant exhibits markedly stronger severing activity than the S3A mutant. This distinct biochemical difference implies that these dual sites function synergistically.

2. pT70 Stoichiometry and Physiological Relevance: Technically, global phosphoproteomic analysis is highly susceptible to ion suppression, where signals from low-abundance peptides are easily eclipsed by high-abundance peptides. This likely accounts for the masking of the T70 site in our initial global phosphoproteomics. Importantly, the PhosphoSitePlus database

documents several independent publications that have successfully identified endogenous CFL1 T70 phosphorylation, supporting the physiological relevance of T70 phosphorylation. We have added this to the Discussion section (Page 24, Lines 682-687, in red).

Minor Points

1. The kinase activity inference now uses three complementary approaches (KSEA, PhosR motif-centric, PTM-SEA), which is excellent. However, the authors should clarify how concordance between methods was defined and report the overlap statistics more explicitly. For instance, how many kinases were identified by all three methods versus only one?

We thank the Reviewer for this suggestion. To clearly define the concordance between the three methods and explicitly report the overlap statistics, we have updated both the main text and the figure legends (Page 7, Lines 188-199; Page 43, Lines 1268-1272; in red).

2. The increased phosphosite localization cutoff (>0.9 from the default >0.75) is appropriate and welcome. The authors should report how many phosphosites were retained after this stricter filtering and confirm that all downstream analyses (including kinase activity inference) use only Class I sites at the higher threshold.

We thank the reviewer for this comment. In our previous revision (Page6, Line169; Page26, Line726), we have documented the number of phosphosites retained after the stricter filtering. Following the reviewer's suggestion, we have added an explicit statement in the revised manuscript (Page 6, Line 171; Page 28, Lines 797-798; in red).

3. The cell cycle analysis (Extended Data Fig. 13a-b) showing no G2/M arrest at 6 h and 12 h but arrest at 24 h is important for interpreting the antiviral mechanism. However, the phosphoproteomics itself was performed at 24 h. The authors should acknowledge that some of the phosphorylation changes captured may reflect cell cycle perturbation in addition to, or downstream of, viral infection.

We thank the reviewer for this insightful comment. Following this suggestion, we have updated the manuscript to include this acknowledgment (Page 19, Line 541-544, in red).

4. The comparison with LIMK inhibitor R10015 is a valuable addition. The mechanistic distinction (LIMK phosphorylates only S3, MELK phosphorylates both S3 and T70) is compelling. Could the authors comment on whether other kinases reported to phosphorylate cofilin-1 (e.g., TESK1/2 at S3) might also contribute to the observed phenotype?

We thank the reviewer for this suggestion. We have updated the manuscript to include this comment about TESK1/2 (Page 24, Line 672-674, in red).

5. Throughout the manuscript, MOI values vary across virus types (SARS-CoV-2 MOI=0.5 or 1.0, HCoV-229E MOI=0.1, HCoV-OC43 MOI=0.5) without clear justification. While this likely reflects different viral replication kinetics, a brief explanation in the Methods would be helpful.

We thank the reviewer for this comment. We have added this explanation to the Methods section in the revised manuscript (Page 29, Line 822-823, in red).

6. The manuscript states that OTSSP167 has been tested in clinical trials for solid tumors and leukemia but does not mention its known dose-limiting hematologic toxicity (thrombocytopenia, neutropenia). Given that this is positioned as a potential antiviral therapeutic, acknowledging these toxicity signals would provide more balanced translational perspective.

We thank the reviewer for this comment. We have acknowledged the drug's toxicity in the revised Discussion (Page 22, Line 634-636, in red).

Reviewer #3 (Remarks to the Author):

The manuscript has been substantially improved, I compliment the authors on their hard work and comprehensive analyses. Most of my comments have been addressed.

The manuscript would benefit from additional proof reading for spelling and grammar, and I have highlighted some typos. Also, in the text several sentences are unclear or overstated. Cloning of the Strep-S construct is still ambiguous and this needs to be clarified.

My comments are outlined below, designated by line number or subfigure:

Line 60: “Protein kinases control nearly all biological processes in eukaryotic cells”. This overreaches a bit, please temper this statement.

We thank the reviewer for pointing this out. The sentence has been modified to: "Protein kinases regulate numerous critical biological processes in eukaryotic cells" (Page 3, Line 60, in red).

66: “Inhibition of MELK activity by OTSSP167 or it’s expression…” Please rephrase for clarity.

We appreciate the reviewer's careful reading.The sentence has been modified to: "Inhibition of MELK activity by OTSSP167, or genetic depletion of its expression" (Page 3, Line 66-67, in red).

93-94: “almost all aspects of cell life.” I suggest rephrasing.

The sentence has been modified to: " numerous critical cellular processes" (Page 4, Line 93-94, in red).

251: “we employed siRNA or sgRNA” – I suggest rephrasing to “we employed siRNA knockdown or Cas9 knockout“

The sentence has been modified to: "we employed siRNA knockdown or Cas9 knockout" (Page 9, Line 258-259, in red).

298-300: “We challenged mice with...OC43…followed by intranasal delivery of OTSSP167 at -1 dpi”. According to the figure and ‘-1 dpi’, the OTSSP167 was added before OC43 in this experiment.

The sentence has been modified to: "OTSSP167 (3.75 mg/kg, i.n.) was administered daily starting from -1 dpi (one day prior to viral challenge) through 2 dpi, with mice being challenged with 2×10^4 FFU of HCoV-OC43 (neurotropic) at 0 dpi" (Page 11, Line 308-311, in red).

305-306: “The attenuated virus replication and expression of pro-inflammatory markers resulted in less histological changes.” More conservative phrasing might use the phrase ‘…was accompanied by…’

The sentence has been modified to: "The attenuated virus replication and expression of pro-inflammatory markers were accompanied by fewer histological changes." (Page 11, Line 317, in red).

563: “mediate most of the signal transduction in eukaryotic cells through phosphorylation”. I’d soften this statement – there are many mechanisms of signal transduction.

The sentence has been modified to: "serve as central regulators of signal transduction in eukaryotic cells through protein phosphorylation." (Page 20, Line 570-571, in red).

597: “surfing” is an unusual way to phrase this.

The word has been modified to: "translocation" (Page 22, Line 617, in red).

785: Please specify the online design tool used to design the sgRNAs.

We followed the Reviewer's suggestion and have specified the design tool (CRISPick) in the revised manuscript (Page 30, Line 857, in red).

807-820: The cloning of the Strep-S construct is still unclear to me. Does the naming order “Strep-S” indicate that the strep tag is located at the N-terminus of Spike? If this is correct, please explain how it was cloned to avoid perturbing processing of the signal peptide at the N-terminus of Spike.

We thank the Reviewer for the careful reading, and sincerely apologize for this oversight in our writing. As the reviewer correctly pointed out, to avoid interfering with the signal peptide processing, the Strep tag was actually cloned at the C-terminus of the Spike protein.Thus, the correct name is "S-Strep". We have updated the figures and the Methods section in the revised manuscript (Page 31, Line 882-889, in red).

Extended Data Fig. 1 g and h – please describe y-axis for the reader.

We thank the reviewer for pointing this out. We have updated the figure legend of Supplementary Fig. 1g and h in the revised manuscript.

1452: Number 11 seems out of place.

We apologize for this oversight and thank the reviewer for pointing it out. It has been deleted in the revised manuscript.

Extended Data Fig 4 f-i – please state the concentration tested.

We thank the reviewer for pointing this out. We have added the concentration of OTSSP167 (0.2 μM) to the figure legend in the revised manuscript.

Extended Data Fig 6d: “FFU/garm”. I think this is a typo.

We apologize for the oversight. We have corrected the mistake in the revised manuscript.

Extended data Fig 15 d: It is somewhat unclear what data are represented by the plots to the right of the SEM images.

We thank the reviewer for pointing this out. We have updated the figure legend to specify that the red dashed boxes represent the microvilli regions and the red arrows indicate the microvilli in the magnified SEM images.
